# Expression of leukemia inhibitory factor in Müller glia cells is regulated by a redox-dependent mRNA stability mechanism

**DOI:** 10.1186/s12915-015-0137-1

**Published:** 2015-04-25

**Authors:** Cavit Agca, Karsten Boldt, Andrea Gubler, Isabelle Meneau, Armelle Corpet, Marijana Samardzija, Manuel Stucki, Marius Ueffing, Christian Grimm

**Affiliations:** Department of Ophthalmology, Lab for Retinal Cell Biology, University of Zurich, Wagistrasse 14, Zurich, 8091 Switzerland; Division of Experimental Ophthalmology and Medical Proteome Center, Centre for Ophthalmology, University of Tübingen, 72076 Tübingen, Germany; Department of Gynecology, University of Zurich, Zurich, 8091 Switzerland; Zurich Center for Integrative Human Physiology (ZIHP), University of Zurich, Zurich, 8091 Switzerland; Neuroscience Center (ZNZ), University of Zurich, Zurich, 8091 Switzerland; Present address: Department of Biomedicine, University Hospital Basel, Basel, 4031 Switzerland; Present address: Center for Molecular and Cellular Physiology and Genetics, University Lyon I, Villeurbanne, France

**Keywords:** LIF, Redox signaling, mRNA stability, Retina, Müller glial cells, ILF3, KHSRP, p38 MAPK, Neuroprotection

## Abstract

**Background:**

Photoreceptor degeneration is a main hallmark of many blinding diseases making protection of photoreceptors crucial to prevent vision loss. Thus, regulation of endogenous neuroprotective factors may be key for cell survival and attenuation of disease progression. Important neuroprotective factors in the retina include H_2_O_2_ generated by injured photoreceptors, and leukemia inhibitory factor (LIF) expressed in Müller glia cells in response to photoreceptor damage.

**Results:**

We present evidence that H_2_O_2_ connects to the LIF response by inducing stabilization of *Lif* transcripts in Müller cells. This process was independent of active gene transcription and p38 MAPK, but relied on AU-rich elements (AREs), which we identified within the highly conserved *Lif* 3′UTR. Affinity purification combined with quantitative mass spectrometry identified several proteins that bound to these AREs. Among those, interleukin enhancer binding factor 3 (ILF3) was confirmed to participate in the redox-dependent *Lif* mRNA stabilization. Additionally we show that KH-type splicing regulatory protein (KHSRP) was crucial for maintaining basal *Lif* expression levels in non-stressed Müller cells.

**Conclusions:**

Our results suggest that H_2_O_2_-induced redox signaling increases *Lif* transcript levels through ILF3 mediated mRNA stabilization. Generation of H_2_O_2_ by injured photoreceptors may thus enhance stability of *Lif* mRNA and therefore augment neuroprotective LIF signaling during degenerative conditions *in vivo*.

**Electronic supplementary material:**

The online version of this article (doi:10.1186/s12915-015-0137-1) contains supplementary material, which is available to authorized users.

## Background

Lack of detailed knowledge about molecular disease mechanisms poses a primary challenge for the development of new therapeutic strategies for blinding diseases. One approach to prevent blindness is to stimulate endogenous neuroprotective pathways to support survival of stressed or injured retinal cells. Whereas overexpression of neurotrophic factors delays or inhibits retinal degeneration [1-7], inhibition of neuroprotective signaling or absence of neuroprotective factors, such as leukemia inhibitory factor (LIF), brain derived neurotrophic factor (BDNF) and fibroblast growth factor 2 (FGF2) accelerates retinal degeneration in disease models, or in the aging retina [2,8,9]. Although increased levels of neurotrophic factors are beneficial for injured retinal cells, a balanced expression is required in the normal retina since exaggerated doses may have detrimental side effects [3,10,11].

One of the neuroprotective factors that is tightly regulated in the neuronal retina is LIF. *Lif* is expressed in a small and dispersed subpopulation of Müller glial cells in response to photoreceptor injury [2] and signals through the LIFR/gp130 receptor complex activating the Janus kinase (JAK)/signal transducer and activator of transcription 3 (STAT3) signaling pathway [2,3,12,13]. Activation of this pathway leads to increased expression of endothelin-2 (*Edn2*), *Fgf2*, *Stat3, Jak3,* suppressor of cytokine signaling 3 *(Socs3)* and glial fibrillary acidic protein, (*Gfap*) [2,12]. Elimination of LIF impairs expression of these factors in the retina and results in a more severe disease progression [2,12]. Thus, LIF induces complicated intercellular signaling events between degenerating photoreceptors and Müller cells that are crucial for photoreceptor survival [2,12-14].

Upregulation of LIF signaling has been observed in induced and inherited photoreceptor degeneration models [2,13,15,16], as well as in models of ganglion cell death [17-19]. Therefore, induction of *Lif* expression may be a common mechanism in the injured retina to support neuronal survival and may be one of the main tasks of Müller cells in their attempt to protect retinal cells against degeneration. Despite its important role in neuronal survival and its unique expression profile in the injured retina, the molecular mechanisms that regulate *Lif* expression in Müller cells are only poorly understood. Recently, we showed that activation of *Lif* gene transcription in the injured retina involves p38 MAPK signaling [20], but additional regulatory mechanisms are likely to exist.

Previous reports have shown that injured photoreceptors generate H_2_O_2_ through nicotinamide adenine dinucleotide phosphate-oxidase (NOX) enzyme complexes [21-23]. In the presence of NOX inhibitors, generation of H_2_O_2_ is impaired and photoreceptor apoptosis is increased in the presence of toxic stress [21-23]. Moreover, increased levels of reactive oxygen species (ROS) upregulate extracellular signal regulated kinase (ERK) and v-akt murine thymoma viral oncogene homolog kinase (AKT) dependent pathways and inhibit the activity of protein phosphatase 2 (PP2A), all of which critically affect photoreceptor survival [24,25]. This seems controversial since H_2_O_2_ and other ROS are well known to have detrimental effects on cell function and viability, and many reports show that oxidative stress contributes to retinal degenerative diseases [26-29]. However, it is now clear that subtoxic levels of H_2_O_2_ have important roles in signal transduction and are involved in many biological pathways [30,31]. Low levels of H_2_O_2_ can reversibly oxidize selective amino acids, such as cysteine, histidine, methionine and selenocysteine, and thus modulate molecular pathways associated with such modified proteins [32-38]. Subtoxic doses of H_2_O_2_ were also shown to participate in neuroprotection by ischemic preconditioning [39] and to induce axonal regeneration in zebrafish [40], supporting the concept that generation of H_2_O_2_ has neuroprotective consequences during stress conditions. Therefore, an intriguing hypothesis suggests that H_2_O_2_ generated by NOX enzymes or released from mitochondria in stressed cells may act as a physiological messenger to regulate expression of neuroprotective factors in Müller cells. This hypothesis is supported by the previously reported regulation of *Lif* expression by p38 MAPK [21], since p38 MAPK signaling can be activated by H_2_O_2_ and may interfere with mRNA stability of target genes. This level of gene regulation involves several RNA binding proteins including tristetraprolin (TTP), which is known to be regulated by p38 MAPK itself [41-43].

Here, we show that H_2_O_2_ enhanced mRNA stability of *Lif* during stress in a Müller cell line and in primary mouse Müller cells. Highly conserved AU-rich elements (AREs) in the *Lif* 3′UTR were important for this regulation and provided target sequences for several RNA binding proteins. Of those, interleukin enhancer binding factor 3 (ILF3) was identified to be critically involved in the regulation of the H_2_O_2_-dependent increase of *Lif* mRNA stability, and KH-type splicing regulatory protein (KHSRP) was identified to be a general regulator of *Lif* mRNA levels independent of redox signaling_._ Our results highlight the complex regulation of *Lif* expression, and provide a mechanism for the puzzling connection between redox signaling and expression of survival factors such as LIF in Müller glia cells.

## Results

### H_2_O_2_ stabilizes *Lif* mRNA in Müller glia cells

Signaling between degenerating photoreceptors and Müller glia cells induces expression of several neuroprotective factors for photoreceptor survival [2,13-15]. Recent evidence suggests that redox mechanisms may be involved in this intercellular communication, and it was proposed that H_2_O_2_, which is produced by stressed photoreceptors, might be a molecule responsible for the induction of retinal survival pathways [22-24].

**Figure 1:**
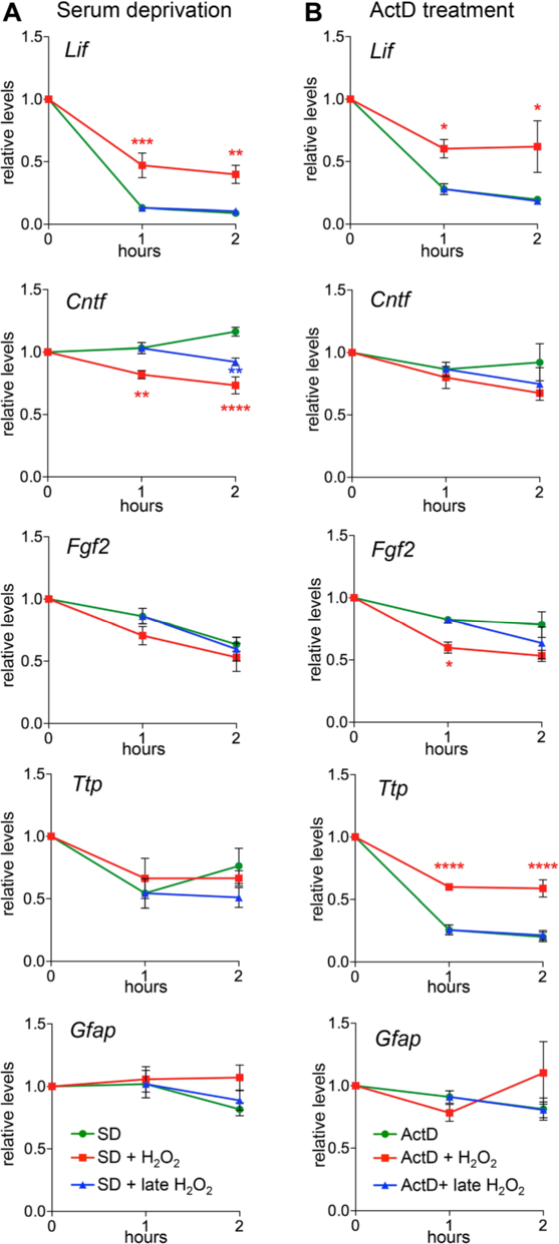
H_2_O_2_ stabilizes Lif transcripts in rMC-1 Müller cells. **A)** rMC-1 cells were serum deprived (SD, circle) for two hours. H_2_O_2_ (50 μM) was added immediately (SD + H_2_O_2_, square) or at one hour during SD (SD + late H_2_O_2_, triangle). **B)** rMC-1 cells were treated with actinomycin D (ActD, circle) for two hours. H_2_O_2_ was added immediately (ActD + H_2_O_2_, square) or at one hour during ActD treatment (ActD + late H_2_O_2_, triangle). RNA levels of the indicated genes were determined by real-time PCR at 0 hour, 1 hour and 2 hours of treatment and expressed relative to levels at 0 hour. Shown are means ± SEM of *N* = 4 to 5. One-way ANOVA with Sidak’s posttests were used to compare RNA levels against levels after SD (A) or ActD (B) treatments at one hour and two hours. (*) *P* <0.05, (**) *P* <0.01, (***) *P* <0.001 and (****) *P* <0.0001. ANOVA, analysis of variance; SEM, standard error of the mean.

Since LIF is one of the critical factors for photoreceptor survival and is expressed in Müller cells in response to photoreceptor injury [3,13,16,44], we tested the effect of subtoxic doses of H_2_O_2_ [45-47] on *Lif* mRNA expression in rMC-1 Müller cells [48]. Since rMC-1 cells expressed high levels of *Lif* mRNA under normal (optimal) culture conditions, stress-related signaling molecules like H_2_O_2_ were without effect and failed to super induce *Lif* expression (Additional file 1: Figure S1). As Müller glia may experience suboptimal (stressed) conditions in an injured retina, we serum deprived (SD) cells to impose a mild stress during the period of H_2_O_2_ signaling *in vitro*. This caused a rapid and strong reduction of *Lif* mRNA levels in Müller cells by 87% within one and 91% within two hours (Figure 1A). Whether this was due to the suboptimal conditions, or to the absence of *Lif*-inducing factors potentially present in serum has not been addressed. Importantly, SD allowed us to investigate the effect of H_2_O_2_ on *Lif* levels. Addition of H_2_O_2_ partially prevented this downregulation and resulted in *Lif* transcript levels that were 3.6-fold (one hour, *P* <0.001) and 4.5-fold (two hours, *P* <0.01) higher than in cells treated with SD alone (Figure 1A). H_2_O_2_ needed to be present from the beginning of SD treatment since its late addition (after one hour of SD treatment) did not affect *Lif* levels (Figure 1A). Similarly, H_2_O_2_ had no effect on *Lif* expression when added to rMC-1 cells under normal cell culture conditions (Additional file 1: Figure S1). The effect of H_2_O_2_ was specific for *Lif* since the mRNA levels of other genes known to be expressed in Müller cells, such as ciliary neurotrophic factor (*Cntf*), *Fgf2* and *Gfap* [2,13,49], or known to be postranscriptionally regulated at the level of mRNA stability, such as *Ttp* [42,50], were not increased by the addition of H_2_O_2_. Rather, *Cntf* was downregulated by 18% (one hour, *P* <0.01) and 27% (two hours, *P* <0.0001) in SD cells treated with H_2_O_2_ (Figure 1A).

To test whether a transcriptional or posttranscriptional mechanism was responsible for the H_2_O_2_-induced increase of *Lif* mRNA levels, we used actinomycin D (ActD) to block RNA polymerase II dependent transcription. Like SD, ActD treatment caused a rapid decrease of *Lif* mRNA levels, whereas other transcripts (except for *Ttp*) were not strongly affected. Importantly, H_2_O_2_ increased *Lif* transcripts by 2.2 fold (one hour, *P* <0.05) and 3.2 fold (two hours, *P* <0.05) also in the presence of ActD (Figure 1B) suggesting a posttranscriptional mechanism for the control of *Lif* mRNA levels. Since H_2_O_2_ is a reactive molecule that may potentially reduce the activity of ActD, we confirmed that ActD was fully active in the presence of H_2_O_2_ and completely blocked tumor necrosis factor-alpha (TNF)-induced *Lif* transcription (Additional file 1: Figure S1). H_2_O_2_ also significantly increased *Ttp* mRNA levels in the presence of ActD (Figure 1B) showing that *Ttp* mRNA levels can also be regulated on a posttranscriptional level in rMC-1 cells.

**Figure 2:**
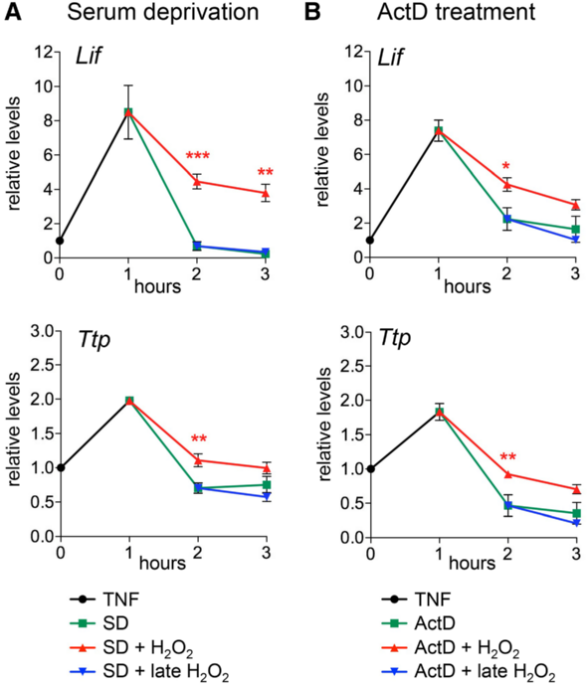
H_2_O_2_ stabilizes Lif transcripts after TNF induced gene expression in rMC-1 Müller cells. rMC-1 cells were treated with tumor necrosis factor-alpha (TNF, circle) for one hour to induce *Lif* gene expression. Following TNF treatment cells were either **(A)** serum deprived (SD, square) or **(B)** incubated with ActD (square). H_2_O_2_ (50 μM) was added either at the one hour (+ H_2_O_2_, triangle) or the two hour time-point (+ late H_2_O_2_, del). RNA levels of *Lif* and *Ttp* were determined by real-time PCR at the indicated time-points and expressed relative to levels at 0 hour. Shown are means ± SEM of *N* = 4. One-way ANOVA with Sidak’s posttests were used to compare RNA levels against levels after SD or ActD treatments at two hours and three hours. (*) *P* <0.05, (**) *P* <0.01, (***) *P* <0.001. ActD, actinomycin D. ANOVA, analysis of variance; SEM, standard error of the mean.

Since it was shown that retinal degenerations activate *Lif* mRNA transcription [21,50] and H_2_O_2_ clearly affected a posttranscriptional process, we tested whether H_2_O_2_ can increase longevity of *Lif* mRNA after an initial boost of mRNA production. We treated rMC-1 cells with TNF, which was previously shown to transiently upregulate both *Lif* and *Ttp* transcription [21,50]. When applied at one hour of TNF-induced upregulation of gene transcription, SD and ActD treatment caused a rapid decline of *Lif* and *Ttp* mRNA levels. This decline was partially prevented by the addition of H_2_O_2_ suggesting that redox signaling can also stabilize *Lif* and *Ttp* mRNAs under conditions of increased gene activity (Figure 2A, B). The data argue that H_2_O_2_ may function as a signaling molecule to overcome the unstable nature of *Lif* and *Ttp* mRNAs resulting in a more sustained expression of these genes when needed.

**Figure 3:**
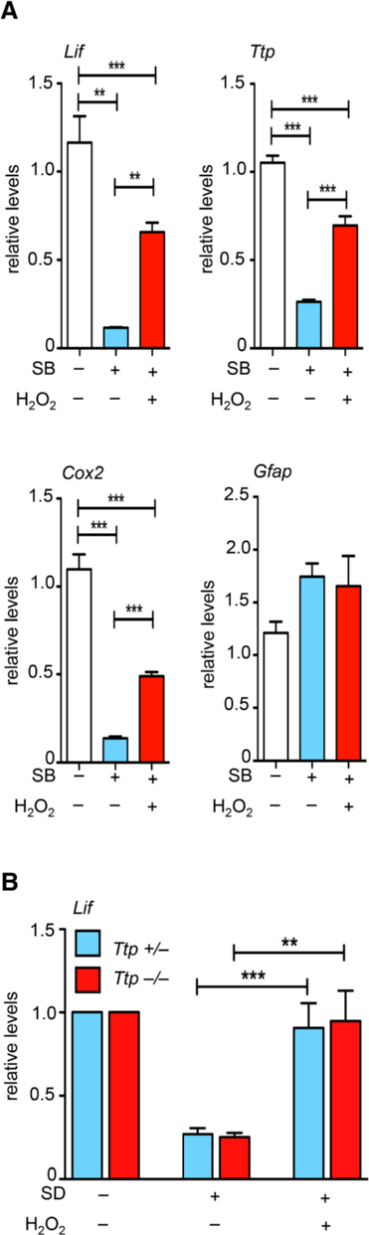
H_2_O_2_-induced stabilization of Lif in rMC-1 and primary Müller cells is independent of p38 MAPK. **A)** rMC-1 cells were (+) or were not (−) treated with p38 MAPK inhibitor SB202190 (SB) in the presence (+) or absence (−) of H_2_O_2_ (50 μM) for one hour. RNA levels were determined by real-time PCR and expressed relative to the levels in untreated cells. Shown are means ± SEM of *N* = 6 (five independent experiments). One-way ANOVA with Tukey’s posttests were used to compare all pairs of columns. **B)** Primary mouse Müller cells isolated from *Rlbp-GFP* mice heterozygous (*Ttp* ^*+/−*^) or homozygous (*Ttp* ^*−/−*^) for the *Ttp* knockout allele were (+) or were not (−) serum deprived (SD) for two hours in the presence (+) or absence (−) of H_2_O_2_ (50 μM). *Lif* mRNA levels were determined by real-time PCR at two hours of treatment and expressed relative to the levels of untreated cells. Shown are means ± SEM of *N* = 3 to 4. One-way ANOVA with Sidak’s posttests were used to compare the effect of treatment for both *Ttp* ^*+/−*^ and *Ttp* ^*−/−*^ Müller cells. (**) *P* <0.01 and (***) *P* <0.001. ANOVA, analysis of variance; SEM, standard error of the mean.

As already reported earlier, inhibition of p38 MAPK by SB202190 (SB) strongly decreased *Lif* mRNA levels in rMC-1 cells (Figure 3A, [21]). Similar to the effect after SD or ActD treatment, addition of H_2_O_2_ to SB-treated cells increased *Lif* mRNA levels. This argues that redox dependent *Lif* mRNA stabilization may not depend on p38 MAPK, even though several components of its signaling system are activated by H_2_O_2_ (Additional file 2: Figure S2A, B) [51-53]. To strengthen this conclusion, we tested whether H_2_O_2_ might be able to increase *Lif* mRNA levels in the absence of TTP, an important component of the p38 MAPK-dependent mRNA stability pathway [41-43]. Indeed, H_2_O_2_ rescued the SD-induced downregulation of *Lif* mRNA levels also in primary Müller cells isolated from *Ttp*^−/−^ mice (Figure 3B). Importantly, these experiments also showed that redox regulation of *Lif* was not specific for rMC-1 cells, but was also a feature of primary Müller cells making it likely that this mechanism is relevant for the regulation of *Lif* expression *in vivo*.

Inhibition of p38 MAPK signaling and addition of H_2_O_2_ also affected mRNA levels of cyclooxygenase 2 (*Cox2*) and *Ttp* comparably to *Lif* (Figure 3A). *Gfap* levels, however, were not significantly altered by the treatments. Interestingly, mRNAs of *Cox2* and *Ttp*, but not of *Gfap*, contain several AREs that regulate their stability [42,54].

### *Cis*-regulation of *Lif* mRNA stability

**Figure 4:**
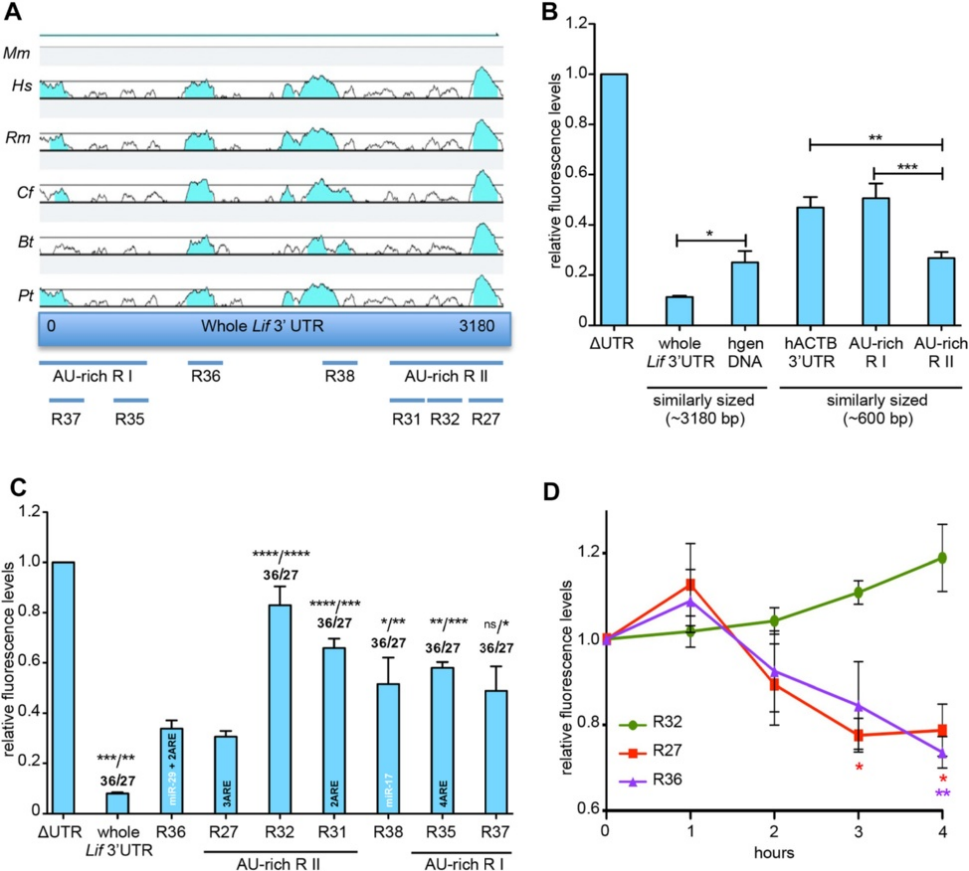
The Lif 3′UTR contains conserved regions with AREs that influence RNA levels in rMC-1 cells. **A)** Sequence alignment of the 3,180 bp long *Lif* 3′UTR sequences from *Mus musculus* (*Mm)*, *Homo sapiens* (*Hs*)*, Rhesus monkey* (*Rm*)*, Canis lupus familiaris* (*Cf*)*, Bos taurus* (*Bt*) and *Pan troglodytes* (*Pt*) revealed two highly conserved AU-rich regions (R I and R II) and seven smaller regions containing AREs (R27, R31, R32, R35, R37), a miRNA binding site (R38) or both (R36). **B, C)** rMC-1 cells were transfected with luciferase-reporter constructs, as indicated. Luminescence levels were determined after 24 hours and expressed relative to a construct with a minimal 3′UTR (ΔUTR). **D)** rMC-1 cells were transfected with luciferase-reporter constructs carrying R36, R27, R32 or ΔUTR sequences. Gene expression was blocked with ActD 24 hours after transfection. Luminescence levels were determined at 0 to 4 hours after ActD addition and shown relative to the levels of the ΔUTR control construct that were set to 1 for each time point. Shown are means ± SEM of *N* = 4 to 5. One-way ANOVA (B, C) or two-way ANOVA (D) with Dunnett’s posttests were used to compare constructs to the levels of the ΔUTR construct. (*) *P* <0.05, (**) *P* <0.01, (***) *P* <0.001 and (****) *P* <0.0001. ANOVA, analysis of variance; AREs, AU-rich elements; SEM, standard error of the mean.

Since *Lif* encodes a highly unstable mRNA (Figures 1 and 2) and stability of particular RNAs like *Cox2* can be regulated through proteins that bind to AREs within their 3′UTRs [54,55], we analyzed the sequence of the 3,180 bp long *Lif* 3′UTR for the presence of AREs. Alignment of the 3′UTRs from various mammalian *Lif* genes [56,57] showed that several regions were strongly conserved (Figure 4A). Two large regions (termed AU-rich R I and AU-rich R II) were identified that were particularly rich in adenosine and uracil (Figure 4A). These two regions contained several smaller regions with AUUU sequence elements and AUUUA core motifs (regions 27, 31, 36). Region 35 was an exception in that it contained only a string of 7 AUUU sequences without core motif. In addition, two conserved putative binding sites for miR-29 (region 36) and miR-17 (region 38) were identified using TargetScan software [58]. No AREs or miRNA binding sites were detected in regions 32 and 37 (Figure 4A, Additional file 3: Figure S3A).

To evaluate the potential importance of these conserved elements for the regulation of *Lif* RNA stability, we fused fragments of the mouse 3′UTR to a luciferase gene driven by the SV40 promoter and enhancer (Table 1, Additional file 3: Figure S3B). The incorporation of CL1 [59] and PEST [60] sequences led to the production of an unstable luciferase protein (Luc2CP) suitable to monitor changes in mRNA levels by measuring luminescence in cell culture homogenates.

**Table 1:** **Vector list of cloned regions used in constructs**

**Vector name**	**Lif 3′UTR position (1 to 3180)**	**Length of 3′UTR (bp)**	**AREs**	**Sequence source**	**Cloning method**
∆UTR	0	0	0	NA	RE
Whole Lif 3′UTR	1 to 3180	3180	11	mus musculus	PCR/RE
hgen DNA	NA	3142	2	homo sapiens	PCR/RE
AU-rich R I	1 to 570	570	4	mus musculus	PCR/RE
AU-rich R II	2556 to 3180	625	5	mus musculus	RE
hACTB 3′UTR	NA	600	3	homo sapiens	PCR/RE
R27 (wt)	2999 to 3180	182	3	mus musculus	RE
R32	2805 to 2998	194	0	mus musculus	PCR/RE
R31	2541 to 2812	272	2	mus musculus	PCR/RE
R38	2079 to 2245	167	0	mus musculus	PCR/RE
R36 (wt)	1062 to 1231	170	2	mus musculus	PCR/RE
R35	398 to 570	173	4	mus musculus	PCR/RE
R37	62 to 229	168	0	mus musculus	PCR/RE
R27-A	2999 to 3180	182	1	mus musculus	SD
R27-B	2999 to 3180	182	1	mus musculus	RE/AN
R27-C	2999 to 3180	182	1	mus musculus	SD
R27-No	2999 to 3180	182	0	mus musculus	SD
R36-DE	1062 to 1231	170	2	mus musculus	SD
R36-D	1062 to 1231	170	1	mus musculus	SD
R36-E	1062 to 1231	170	1	mus musculus	SD
R36-No	1062 to 1231	170	0	mus musculus	SD
R27 2 ARE	2999 to 3180	182	2	mus musculus	SD
R27 4 ARE	2999 to 3180	237	4	mus musculus	SD
R27 9 ARE	2999 to 3180	351	9	mus musculus	SD

Name and details of position, length, number of putative AREs, and cloning strategies are listed for individual 3′UTR sequences used in the luciferase experiments. AREs, AU-rich elements; hgen, human genomic DNA; NA, not applicable; PCR, polymerase chain reaction; RE, restriction digestion; RE/AN, restriction digestion/primer annealing; SD, site-directed mutagenesis.

Fusion of the whole *Lif* 3′UTR (3,180 bp) to the reporter gene reduced expression of Luc2CP by 89% compared to a construct with a minimal 3′UTR (ΔUTR) (Figure 4B). When the full length *Lif* 3′UTR was compared to a similar-sized human genomic sequence without conserved, genic or repetitive sequences this reduction was 55% (Figure 4B), excluding that *Lif* mRNA destabilization was mediated non-specifically by its long 3′UTR sequence. This suggested that the *Lif* 3′UTR contained *cis* regulatory elements leading to reduced levels of the reporter protein. Test of the individual AU-rich regions I and II (570 bp and 625 bp, respectively; Table 1) showed that region II strongly reduced luciferase levels by 43%, whereas region I had no effect when compared to a similar sized region from the human *ACTB* 3′UTR (Figure 4B). This suggested that region II may contain elements for the *cis*-regulation of *Lif* mRNA stability. To identify the elements responsible for the effect, we tested shorter sequences within both regions. In addition, short sequence elements that contained a putative binding site for miR-29 in connection with two AREs (region 36) or a putative binding site for miR-17 (region 38) were also tested (Figure 4C, Table 1). Whereas several of these sequence elements decreased luciferase levels only mildly, regions 36 and 27 had strong and significant effects (Figure 4C). These data identified two small regions important for the regulation of *Lif* mRNA stability and suggested that region 27 may be responsible for the destabilizing effect observed with the larger region II (Figure 4B). To confirm that regions 27 and 36 regulate *Lif* mRNA levels through RNA stability, we transfected rMC-1 cells with the respective reporter constructs and followed Luc2PC luminescence in the presence of ActD. Cells expressing the reporter gene fused to regions 27 or 36 lost Luc2PC luminescence significantly faster than cells expressing the reporter containing region 32 (no ARE) (Figure 4D). Thus, regions 27 and 36 influenced RNA levels, indeed, through regulation of RNA stability.

**Figure 5:**
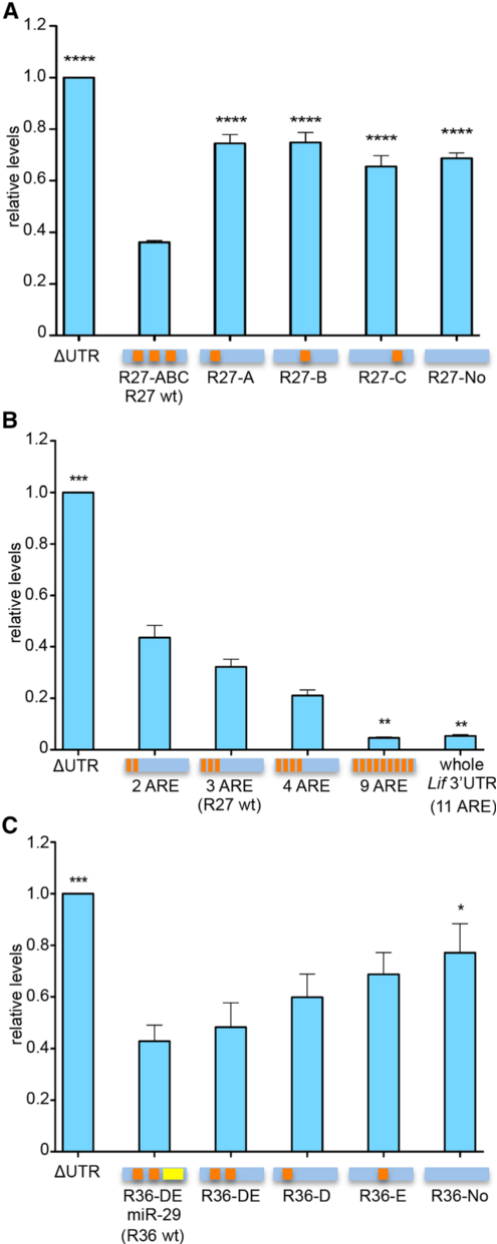
AREs are involved in cis-regulation of Lif mRNA stability in rMC-1 Müller cells. **A)** rMC-1 cells were transfected with reporter constructs fused to wild type or mutated R27 sequences (see Additional file 4: Figure S4A), as indicated. **B)** rMC-1 cells were transfected with reporter constructs fused to R27 sequences containing increasing numbers of AREs (see Additional file 4: Figure S4B), as indicated. **C)** rMC-1 cells were transfected with reporter constructs fused to indicated wild type or mutated R36 sequences (see Additional file 4: Figure S4C), as indicated. Luminescence levels were always determined after 24 hours and shown relative to the level generated by the ΔUTR control construct. Shown are means ± SEM of *N* = 3 to 4. Squares in horizontal bars indicate AREs or an miR-29 binding site, respectively, in the analyzed sequences. One-way ANOVA with Dunnett’s posttests were used to compare expression levels to constructs containing the respective wild type sequence **(A, C)** or to ‘2 ARE’ **(B)**. (*) *P* <0.05, (**) *P* <0.01, (***) *P* <0.001 and (****) *P* <0.0001. ANOVA, analysis of variance; AREs, AU-rich elements; SEM, standard error of the mean.

In order to identify the functional elements within regions 27 and 36, we mutated individual AUUU sequences (Figure 5, Table 1, Additional file 4: Figure S4). Region 27 had three predicted AREs (A, B and C) that, when mutated, significantly increased reporter expression (Figure 5A). Mutating two elements in either combination resulted in Luc2PC levels similar to the triple mutant suggesting that none of the individual AREs was sufficient to significantly reduce Luc2PC expression but that the elements may need to interact or to act synergistically to regulate mRNA stability. Consistent with a synergistic effect, increasing the number of AREs within the same reporter construct gradually decreased reporter expression to levels found for the whole *Lif* 3′UTR (Figure 5B, Additional file 4: Figure S4B). Region 36 contained two predicted AREs (D and E) and a conserved miR-29 binding site (Additional file 3: Figure S3A). Mutating the miR-29 binding sequence alone or in combination with either ARE did not significantly alter Luc2CP levels. In contrast, simultaneous elimination of all three elements significantly increased Luc2CP levels (Figure 5C).

Overall, these experiments identified ARE elements within regions 27 and 36 that are involved in the regulation of *Lif* mRNA stability in Müller cells.

### ILF3 influences redox-dependent *Lif* mRNA stability in Müller glia

To identify proteins that specifically bind to AREs in regions 27 and 36 we incubated the respective RNA sequences or their ARE-null counterparts (Additional file 4: Figure S4A, C) with protein extracts from rMC-1 cells. Mass spectrometry (MS)-based quantitative proteomics identified those proteins that preferentially bound to the wild type sequences of regions 27 or 36 (Tables 2 and 3; the mass spectrometry proteomics data have been deposited to the ProteomeXchange Consortium [61] via the PRIDE partner repository with the dataset identifier PXD001463). Gene Ontology (GO)-pathway analysis on identified proteins showed that pools were enriched in RNA binding proteins and contained 11 (region 27) and 14 (region 36) such proteins (Additional file 5: Figure S5A, B; blue circles, Additional file 6: File S1, Additional file 7: File S2). Whereas RNA binding proteins identified with region 36 were mainly linked to translational initiation and elongation as well as RNA catabolic processes (Table 3, stars, Additional file 7: File S2), region 27 RNA binding proteins were primarily associated with double-stranded RNA binding and mRNA stability (Table 2, stars, Additional file 6: File S1). Interestingly, the protein pool identified with region 27 was also enriched with cell redox homeostasis proteins (Table 2, squares; Additional file 5: Figure S5A, blue square, Additional file 6: File S1).

**Table 2:** **List of RNA binding proteins associated with region 27 of the Lif 3′UTR**

**Protein ID**		**Protein name**	**Gene name**	**27 wt versus 27-No Significance**	**27 wt versus 27-No Rel. levels**
Q15717	✩	ELAV-like protein 1	ELAVL1	1.88E-08	8.50
P13693		Translationally-controlled tumor protein	TPT1	0.0005	3.69
Q01469		Fatty acid-binding protein, epidermal	FABP5	0.0007	3.58
Q12906	✩	Interleukin enhancer-binding factor 3	ILF3	0.0023	3.11
P07741		Adenine phosphoribosyltransferase	APRT	0.0065	2.74
O75390		Citrate synthase, mitochondrial	CS	0.0069	2.72
Q9Y281		Cofilin-2	CFL2	0.0088	2.62
Q15185		Prostaglandin E synthase 3	PTGES3	0.0091	2.62
Q04760		Lactoylglutathione lyase	GLO1	0.0095	2.60
A6NL28		Putative tropomyosin alpha-3 chain-like protein	PTA3CLP	0.0103	2.57
Q12905	✩	Interleukin enhancer-binding factor 2	ILF2	0.0119	2.52
P61956		Small ubiquitin-related modifier 2-3-4	SUMO2-3-4	0.0131	2.48
P48735		Isocitrate dehydrogenase [NADP], mitochondrial	IDH2	0.0150	2.43
Q96SI9	✩	Spermatid perinuclear RNA-binding protein	STRBP	0.0155	2.42
P62633		Cellular nucleic acid-binding protein	CNBP	0.0173	2.38
P09104		Gamma-enolase	ENO2	0.0200	2.33
P30086		Phosphatidylethanolamine-binding protein 1	PEBP1	0.0214	2.31
P20962		Parathymosin	PTMS	0.0219	2.30
P61326	✩	Protein mago nashi homolog-B	MAGOH-B	0.0225	2.29
P50395		Rab GDP dissociation inhibitor beta	GDI2	0.0233	2.28
Q14019		Coactosin-like protein	COTL1	0.0234	2.27
P07737		Profilin-1	PFN1	0.0242	2.26
P00505		Aspartate aminotransferase, mitochondrial	GOT2	0.0243	2.26
Q9BTT0		Acidic leucine-rich nuclear phosphoprotein 32 family member E	ANP32E	0.0248	2.25
O00299		Chloride intracellular channel protein 1	CLIC1	0.0268	2.22
Q9NR31		GTP-binding protein SAR1a	SAR1A	0.0280	2.21
Q8NBS9	□	Thioredoxin domain-containing protein 5	TXNDC5	0.0289	2.20
P29558	✩	RNA-binding motif, single-stranded-interacting protein 1	RBMS1	0.0311	2.17
Q99471		Prefoldin subunit 5	PFDN5	0.0330	2.15
P13667	□	Protein disulfide-isomerase A4	PDIA4	0.0336	2.14
P60903		Protein S100-A10	S100A10	0.0365	2.11
P61088		Ubiquitin-conjugating enzyme E2 N	UBE2N	0.0381	2.10
P09382		Galectin-1	LGALS1	0.0410	2.07
P62834		Ras-related protein Rap-1A	RAP1A	0.0428	2.05
P09972		Fructose-bisphosphate aldolase C	ALDOC	0.0435	2.05
P30040		Endoplasmic reticulum resident protein 29	ERP29	0.0440	2.04
O60493		Sorting nexin-3	SNX3	0.0451	2.04
P22626	✩	Heterogeneous nuclear ribonucleoproteins A2/B1	HNRNPA2B1	0.0456	2.03
Q15843		NEDD8	NEDD8	0.0464	2.03
P30101	□	Protein disulfide-isomerase A3	PDIA3	0.0464	2.03
P19338	✩	Nucleolin	NCL	0.0470	2.02
O15511		Actin-related protein 2/3 complex subunit 5	ARPC5	0.0481	2.01
P00338		L-lactate dehydrogenase A chain	LDHA	0.0501	1.20
Q15121		Astrocytic phosphoprotein PEA-15	PEA15	0.0517	1.99
P62937		Peptidyl-prolyl cis-trans isomerase A	PPIA	0.0556	1.96
P14174		Macrophage migration inhibitory factor	MIF	0.0559	1.96
Q14103	✩	Heterogeneous nuclear ribonucleoprotein D0	HNRNPD	0.0559	1.96
Q99729	✩	Heterogeneous nuclear ribonucleoprotein A/B	HNRNPAB	0.0587	1.94
O14979	✩	Heterogeneous nuclear ribonucleoprotein D-like	HNRPDL	0.0602	1.93
P10599	□	Thioredoxin	TXN	0.0602	1.93

RNAs of region 27 (‘R27 wt’) and its counterpart ARE-null region 27 (‘R27-No’) were used for RNA binding assays. Bound proteins were identified by quantitative mass spectrometry analysis and relative ratios were determined using total sequence reads for region ‘R27 wt’ and region ‘R27-No’. RNA binding proteins, (stars) and cell redox homeostasis proteins (squares) are indicated as identified by GO pathway analysis,(Additional file 6: File S1). The significance is the significance A *P*-value as determined by the Perseus software, defining the likelihood of a protein being enriched compared to the background distribution [112].

**Table 3:** **List of RNA binding proteins associated with region 36 of the Lif 3′UTR**

**Protein ID**		**Protein name**	**Gene name**	**36 wt versus 36-No Significance**	**36 wt versus 36-No Rel. levels**
Q13126		S-methyl-5-thioadenosine phosphorylase	MTAP	6.70E-06	2.11
Q9Y3U8		60S ribosomal protein L36	RPL36	1.43E-05	2.05
P30040		Endoplasmic reticulum resident protein 29	ERP29	8.03E-05	1.91
P62942		Peptidyl-prolyl cis-trans isomerase FKBP1A	FKBP1A	0.0003	1.80
Q14019		Coactosin-like protein	COTL1	0.0005	1.76
O75390		Citrate synthase, mitochondrial	CS	0.0012	1.68
P15121		Aldose reductase	AKR1B1	0.0015	1.66
Q9Y281		Cofilin-2	CFL2	0.0019	1.64
Q02543	✩	60S ribosomal protein L18a	RPL18A	0.0020	1.64
P30041		Peroxiredoxin-6	PRDX6	0.0022	1.63
P42766	✩	60S ribosomal protein L35	RPL35	0.0024	1.62
Q96AG4		Leucine-rich repeat-containing protein 59	LRRC59	0.0041	1.57
Q01130	✩	Serine/arginine-rich splicing factor 2	SRSF2	0.0051	1.55
P26447		Protein S100-A4	S100A4	0.0080	1.51
P60059		Protein transport protein Sec61 subunit gamma	SEC61G	0.0089	1.50
Q96AE4	✩	Far upstream element-binding protein 1	FUBP1	0.0090	1.50
P09493		Tropomyosin alpha-1 chain	TPM1	0.0099	1.49
P40616		ADP-ribosylation factor-like protein 1	ARL1	0.0104	1.48
Q99536		Synaptic vesicle membrane protein VAT-1 homolog	VAT1	0.0115	1.47
P84098	✩	60S ribosomal protein L19	RPL19	0.0120	1.47
P61019		Ras-related protein Rab-2A	RAB2A	0.0127	1.47
Q9HAV7		GrpE protein homolog 1, mitochondrial	GRPEL1	0.0127	1.47
O43396		Thioredoxin-like protein 1	TXNL1	0.0136	1.46
P26639		Threonine--tRNA ligase, cytoplasmic	TARS	0.0137	1.46
P09972		Fructose-bisphosphate aldolase C	ALDOC	0.0143	1.45
Q01581		Hydroxymethylglutaryl-CoA synthase, cytoplasmic	HMGCS1	0.0144	1.45
P63261		Actin, cytoplasmic 2; Actin, cytoplasmic 2, N-terminally processed	ACTG1	0.0152	1.45
Q99497	✩	Protein DJ-1	PARK7	0.0180	1.43
P16989	✩	DNA-binding protein A	CSDA	0.0192	1.42
P06748	✩	Nucleophosmin	NPM1	0.0212	1.41
Q9BTT0		Acidic leucine-rich nuclear phosphoprotein 32 family member E	ANP32E	0.0221	1.41
P39023	✩	60S ribosomal protein L3	RPL3	0.0227	1.41
P13693		Translationally-controlled tumor protein	TPT1	0.0267	1.39
P41567	✩	Eukaryotic translation initiation factor 1	EIF1	0.0270	1.39
P49207	✩	60S ribosomal protein L34	RPL34	0.0290	1.38
Q9Y266		Nuclear migration protein nudC	NUDC	0.0305	1.38
Q15020	✩	Squamous cell carcinoma antigen recognized by T-cells 3	SART3	0.0331	1.37
P09429		High mobility group protein B1; Putative high mobility group protein B1-like 1	HMGB1	0.0364	1.36
P40429		60S ribosomal protein L13a; Putative 60S ribosomal protein L13a-like	RPL13A	0.0365	1.36
O43747		AP-1 complex subunit gamma-1	AP1G1	0.0400	1.35
Q00577		Transcriptional activator protein Pur-alpha	PURA	0.0410	1.35
P27635		60S ribosomal protein L10	RPL10	0.0414	1.34
P00558		Phosphoglycerate kinase 1	PGK1	0.0462	1.33
P22314		Ubiquitin-like modifier-activating enzyme 1	UBA1	0.0473	1.33
Q9H0U4		Ras-related protein Rab-1B	RAB1B	0.0475	1.33
P61106	✩	Ras-related protein Rab-14	RAB14	0.0495	1.32
P49588		Alanine--tRNA ligase, cytoplasmic	AARS	0.0503	1.32
O75915	✩	PRA1 family protein 3	ARL6IP5	0.0505	1.32
P55795		Heterogeneous nuclear ribonucleoprotein H2	HNRNPH2	0.0523	1.32
P30086		Phosphatidylethanolamine-binding protein 1; Hippocampal cholinergic neurostimulating peptide	PEBP1	0.0533	1.32
P68036		Ubiquitin-conjugating enzyme E2 L3	UBE2L3	0.0580	1.31
Q9UQE7		Structural maintenance of chromosomes protein 3	SMC3	0.0583	1.31

RNAs of region 36 (‘R36 wt’) and its counterpart ARE-null region 36 (‘R36-No’) were used for RNA binding assays. Bound proteins were identified by quantitative mass spectrometry analysis and relative ratios were determined using total sequence reads for region ‘R36 wt’ and region ‘R36-No’. RNA binding proteins (stars) are indicated as identified by GO pathway analysis (see Additional file 7: File S2). The significance is the significance A *P*-value as determined by the Perseus software, defining the likelihood of a protein being enriched compared to the background distribution.

ELAV Like RNA Binding Protein 1 (ELAVL1) and ILF3, two well-known ARE-binding proteins [62-65], were identified as primary candidates for targeting AREs in region 27 of the *Lif* 3′UTR (Table 2). Interestingly, ILF3 together with ELAVL1 was previously shown to regulate gene expression of mitogen-activated protein kinase phosphatase 1 (*Mkp1*) through an H_2_O_2_-dependent mechanism [64]. ILF3 was additionally shown to interact with several RNA binding proteins including heterogeneous nuclear ribonucleoprotein (HNRNP) D, HNRNPA_2_/B_1_ and HNRNPA/B [66]. All of these HNRNPs were identified by our MS analysis and shown to preferentially bind the 27 wild type over the respective ARE-null sequence (Table 2); but none reached statistical significance (*P* >0.05). Since HNRNPD not only is an interacting partner of ILF3 but also a known ARE binding protein, which potentially directly influences stability of target mRNAs [67-69], we included it in our further analyses.

**Figure 6:**
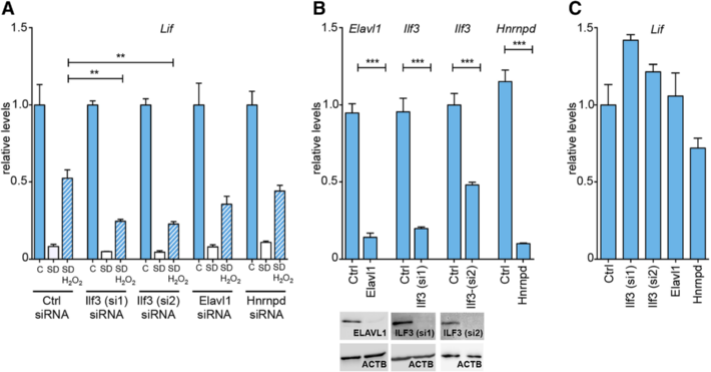
ILF3 regulates redox dependent mRNA stabilization in rMC-1 Müller cells. **A)** rMC-1 cells were transfected with siRNAs directed against *Ilf3*, *Elavl1* or *Hnrnpd*, as indicated. Scrambled siRNA was used as control (Ctrl). Medium was changed 48 hours after transfection and then cells were (SD) or were not **(C)** serum deprived. H_2_O_2_ (50 μM) was added to the indicated samples. RNA levels were determined by real-time PCR two hours after treatment and expressed relative to untreated cells **(C)**, which were set to 1 for each knockdown series. *N* = 4 to 8. **B)** Top panel: rMC-1 cells were transfected with siRNAs directed against *Ilf3*, *Elavl1* or *Hnrnpd*, as indicated. Scrambled siRNA was used as control (Ctrl). Medium was changed 48 hours after transfection and respective target RNA levels were determined after two hours by real-time PCR. Bottom panels show protein levels at 48 hours after siRNA transfection. *N* = 4. **C)** rMC-1 cells were transfected with siRNAs directed against *Ilf3*, *Elavl1* or *Hnrnpd*, as indicated. Scrambled siRNA was used as control (Ctrl). Medium was changed 48 hours after transfection and *Lif* RNA levels were determined after two hours by real-time PCR. To knockdown *Ilf3* expression, two different siRNAs (*Ilf3 (si1)*, *Ilf3 (si2)*) were used to exclude off-target effects. Shown are means ± SEM of *N* = 4. One-way ANOVA with Dunnett’s posttests was used to compare *Lif* levels after SD + H_2_O_2_ treatments (A) and in untreated knockdowns against control **(C)**. Student’s t-test was used to compare downregulation of target genes **(B)**. (**) *P* <0.01, (***) P <0.001. ANOVA, analysis of variance; ILF3, interleukin enhancer binding factor 3; SEM, standard error of the mean.

To test whether the identified proteins are indeed involved in redox regulation of *Lif* mRNA stability we silenced their expression in rMC-1 cells and analyzed the effect of H_2_O_2_ on *Lif* mRNA levels. *Ilf3* silencing by two different siRNAs significantly impaired *Lif* mRNA stabilization in H_2_O_2_-treated serum-deprived cells by about 50%. Knockdown of *Elavl1* or *Hnrnpd*, however, had no significant effect (Figure 6A). Controls showed that transfection of siRNA decreased expression levels of target mRNAs by 86% (*Elavl1*), 79% (*Ilf3*; si1), 52% (*Ilf3*; si2) and 91% (*Hnrnpd*), as compared to cells transfected with scrambled siRNA (Ctrl, Figure 6B). Similarly, protein levels of ELAVL1 and ILF3 (even in the case of *Ilf3* siRNA2) were severely reduced in cells treated with the respective siRNAs (Figure 6B, lower panels). Silencing of either gene did not significantly alter *Lif* expression in untreated Müller cells (Figure 6C).

This argued that redox regulated *Lif* mRNA stability may involve ILF3 but not ELAVL1 or HNRNPD.

### KHSRP is an important general regulator of *Lif* expression

**Figure 7:**
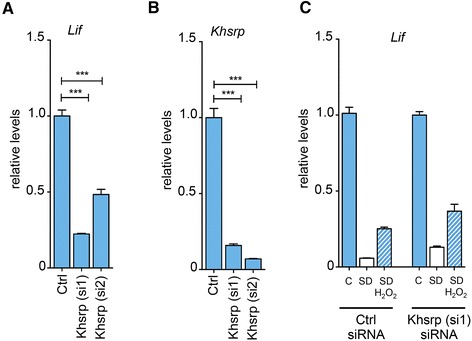
Silencing of Khsrp reduces Lif mRNA levels in non-stressed rMC-1 Müller cells. **A, B)** rMC-1 cells were transfected with siRNAs directed against *Khsrp.* Scrambled siRNA was used as control (Ctrl). Medium was changed 48 hours after transfection and RNA levels for *Khsrp* (A) or *Lif* **(B)** were determined after two hours by real-time PCR. **C)** rMC-1 cells were transfected with siRNAs directed against *Khsrp*. Scrambled siRNA was used as control (Ctrl). Medium was changed 48 hours after transfection and cells were (SD) or were not **(C)** serum deprived. H_2_O_2_ (50 μM) was added to samples as indicated. RNA levels were determined by real-time PCR two hours after treatment and expressed relative to untreated cells **(C)**, which were set to 1 for each knockdown series. Two different siRNAs (*Khsrp (si1)*, *Khsrp (si2)*) were used to exclude off-target effects. Shown are means ± SEM of *N* = 3 to 4. Student’s t-test was used to compare downregulation of *Khsrp* and *Lif* in untreated cells **(A, B)**. One-way ANOVA with Dunnett’s posttests were used to compare *Lif* or *Khsrp* levels to controls **(A, B)** or *Lif* levels after SD + H_2_O_2_ treatments **(C)**. (***) *P* <0.001. ANOVA, analysis of variance; SEM, standard error of the mean.

**Figure 8:**
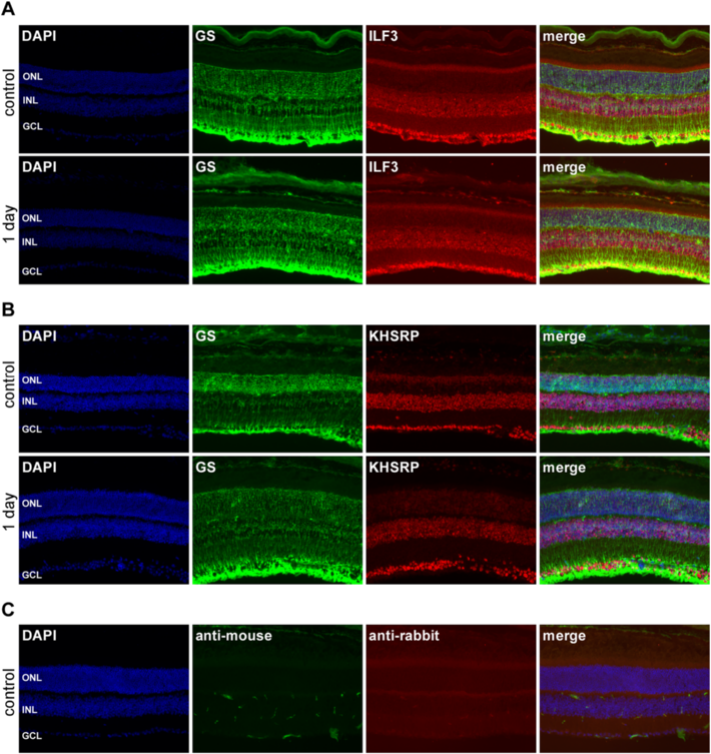
Immunofluorescence of ILF3 and KHSRP in the retina. Retinal sections were prepared from control mice and from mice at one day after light exposure and stained **(A)** for glutamine synthetase (GS, green) and ILF3 (red) or **(B)** for GS (green) and KHSRP (red). **(C)** Control stainings with secondary antibodies only (as indicated). DAPI was used to visualize nuclei. DAPI, 4',6-diamidino-2-phenylindole.

We identified ILF3 to be of importance for regulating *Lif* mRNA levels in redox signaling, probably through its interaction with AREs in the 3′UTR of the gene transcript. However, ILF3 did not affect *Lif* mRNA levels under normal growth conditions. We also showed that TTP, known to be capable of regulating the stability of ARE-containing mRNAs in various systems, was not involved in controlling *Lif* mRNA levels under normal conditions (Figure 3B). Thus, we asked whether other RNA binding proteins may affect *Lif* mRNA levels under normal non-stressed growth conditions. KHSRP was an obvious candidate as it is a multifunctional RNA binding protein involved in both transcriptional and posttranscriptional gene regulation as well as in miRNA biogenesis [70-73]. Furthermore, KHSRP is regulated through p38 MAPK signaling [71] that was shown to be important for *Lif* mRNA transcription [21]. Silencing *Khsrp* to 15% (si1) or 8% (si2) of control levels resulted in 78% (si1) and 52% (si2), respectively, lower *Lif* mRNA levels under normal conditions (Figure 7A, B) suggesting that KHSRP is essential to maintain normal *Lif* mRNA levels in non-stressed cells. However, lack of KHSRP did not affect redox-mediated stabilization of *Lif* transcripts. Note that expression levels of *Lif* in Figure 7C have been normalized to the respective controls (set to 1) to better visualize the effect of H_2_O_2_ in *Khsrp* knockdown cells. These results argue that KHSRP is involved in *Lif* expression independently of redox regulated mRNA stabilization and suggest that mechanisms controlling *Lif* mRNA levels under normal conditions differ from those under stress conditions. KHSRP may be involved in the first, ILF3 in the second, situation. Both proteins showed widespread expression in the normal mouse retina. Cells of the inner nuclear layer and of the ganglion cell layer were intensely stained whereas photoreceptor cells appeared to contain lower levels of the two proteins. Light-induced injury did not affect the expression pattern. Co-staining for glutamine synthetase confirmed expression of ILF3 and KHSRP in Müller glia supporting a role of ILF3 and KHSRP in Müller cells *in vivo* (Figure 8).

## Discussion

### Regulation of Lif expression in Müller cells

Our results show that *Lif* mRNA levels are regulated in stressed Müller cells by a redox controlled stability mechanism that involves AREs in the *Lif* 3′UTR. This mechanism may thus constitute a significant part of the neuroprotective activity of Müller glia cells that critically depends on signaling between injured photoreceptors and Müller cells [2,13-15]. Several reports argue that H_2_O_2_ may act as a signaling molecule in various biological systems [31,32]. Our results support this notion and suggest that the reported neuroprotective effect of H_2_O_2_ in the retina [22-24] may be through its signaling to Müller cells leading to increased expression of *Lif*. H_2_O_2_ may be generated by NADPH oxidases or be released from outer segments in case of photoreceptor injury [74]. Generation or release may occur as long as stress conditions exist and, thus, H_2_O_2_ may signal for a prolonged period of time to ensure a lasting increase of *Lif* mRNA levels in the damaged retina as observed in both the VPP and rd10 models of retinal degeneration [2,16].

In the mammalian retina, basal expression of *Lif* is barely detectable but expression increases after photoreceptor or ganglion cell injury [2,17,19,75]. However, in both primary Müller cells and rMC-1 cells basal *Lif* expression was relatively high, a phenomenon which may be the result of removing Müller cells from their tissue environment disrupting cell-cell interactions and/or of *in vitro* culture conditions. Still, *Lif* expression responded to TNF treatment in rMC-1 cells *in vitro* leading to transiently increased levels similar to the *in vivo* situation [21]. Importantly, H_2_O_2_ also enhanced *Lif* mRNA stability after such a transient upregulation of *Lif* transcription (Figure 2) highlighting the importance of H_2_O_2_ signaling for the prolonged maintenance of increased *Lif* expression during stress conditions. Under normal non-stress conditions, however, H_2_O_2_ did not affect *Lif* levels (Additional file 1: Figure S1), in contrast to mitogen-activated protein kinase phosphatase 1 (*Mkp1*) and placenta growth factor (*Plgf*) that were induced under such conditions [46,64]. Because of its reported side effects in the retina [3,11,12,76], *Lif* expression might be needed to be tightly regulated by a negative feedback system to avoid hazardous high LIF levels under normal conditions. Thus, H_2_O_2_ may be required for *Lif* to overcome this negative feedback and to sustain *Lif* RNA stability ultimately leading to an increased cell survival during degenerative conditions in the retina.

An additional level of control may be the restriction of *Lif* upregulation to a small subpopulation of Müller cells in the injured retina [2]. This may additionally ensure that overall levels of LIF may not exceed a certain threshold, thus safeguarding the retina. Although the molecular mechanism of this regulation is not known, an intriguing possibility is the existence of programmed Müller cells that have the unique capability to respond to H_2_O_2_ as a physiological messenger. Recently, aquaporins 3 and 8 have been identified as channels for H_2_O_2_, a molecule once believed to diffuse freely through cell membranes [47,53,77-80]. Although all known aquaporins have been identified in ocular tissues [81] and aquaporin-4 has been shown to alter its localization in Müller glia cells upon photoreceptor injury [82], it will be important to study the aquaporin expression profiles and localization with respect to H_2_O_2_ signaling and the ensuing Müller cell response.

Even though H_2_O_2_ clearly functioned through the regulation of *Lif* mRNA stability without affecting *Lif* gene transcription, H_2_O_2_ may nevertheless act on other levels as well. It has been shown that H_2_O_2_ activates expression of nuclear factor erythroid 2-related factor (NFE2L2) target genes such as sulfiredoxin and heme-oxygenase 1 [8]. NFE2L2 is a redox regulated transcription factor prominently expressed in Müller cells and astrocytes in the retina [8,83] and binds to specific sequence elements in the 5′UTR of target genes. Interestingly, such elements were not only identified in the *Lif* 5′UTR, but they also had the highest cross-species conservation among other transcription factor binding sites [84]. This raises the possibility that a connection might exist between H_2_O_2_ levels, NFE2L2 activation and initial *Lif* expression. However, we have not addressed the initiation of *Lif* transcription in this study as *Lif* expression was already high in cultured Müller cells. Therefore, *in vivo* studies are required to study such a potential interaction and to better understand redox regulation of *Lif* expression during retinal disease conditions.

### Cis-acting elements for Lif regulation

Various studies have shown that regulation of mRNA stability and, hence, gene expression is closely linked to regulatory sequence elements within the 3′UTR of the target gene. Among those, AREs regulate stability of the associated gene transcript by directing the binding of regulatory proteins. Hao *et al*. have shown that the number of AREs is closely associated with the timing of events during an inflammatory response. Early response genes have the highest number of AREs, which may support fast turnover of transcripts after the initial activation to strictly regulate gene expression [50]. *Lif* mRNA is also associated with a high number of AREs in its 3′UTR (Additional file 3: Figure S3A) and has been shown by us and others to be expressed early and transiently in response to injury [17,21,85]. AREs that were associated with the regulation of *Lif* mRNA stability resided mainly in highly conserved regions in mammals including humans (Figure 4A). This suggests that mechanisms for the regulation of *Lif* expression may be conserved among mammals.

Since H_2_O_2_ increased mRNA levels not only of *Lif* but also of other ARE containing transcripts such as *Ttp* and *Cox2* (Figure 3A), our data support the concept that H_2_O_2_ signaling may control a general pathway for the regulation of ARE-mediated mRNA stability during oxidative stress. Additionally, H_2_O_2_ signaling may also be linked to inflammation since (1) TTP can regulate expression of critical inflammatory response genes such as *Tnf* [41,43], (2) COX2 is an important mediator of the inflammatory response [86]*,* and (3) *Lif* signaling has both pro- and anti-inflammatory properties [87-90]. Since inflammatory events have been implicated in retinal pathologies including age related macular degeneration (AMD) [91], H_2_O_2_ signaling may not only be neuroprotective via regulation of *Lif* expression but may affect the outcome of retinal degenerative diseases on several levels.

### Trans-acting factors for Lif regulation

MS analysis has identified several RNA binding proteins that targeted *Lif* ARE sequences. ELAVL1 and ILF3 were of significant interest among the proteins that bound to AREs of region 27, as both proteins were previously shown to be involved in H_2_O_2_ dependent mRNA stabilization and/or translation [64]. Here, ILF3 but not ELAVL1 was involved in redox dependent regulation of *Lif* mRNA stability in Müller cells (Figure 6). This suggests that ELAVL1 either may not be involved in the regulation of *Lif* mRNA stability or may influence *Lif* expression by other means such as through regulation of RNA splicing [92,93] and/or translation [64].

Interestingly, neither *Lif* nor *Mkp1* mRNAs were identified as ILF3 target RNAs by a high-throughput ribonucleoprotein immunoprecipitation assay [94] even though both *Lif* (this study) and *Mkp1* (Kuwano *et al*. [64]) were shown to be regulated by ILF3 during oxidative stress. Since non-treated HeLa cell lysates were used for those experiments [95], this may indicate that ILF3 may have different binding affinities for target sequences under normal and stress conditions. Here, however, ILF3 was identified as a binding protein for AREs in the *Lif* 3′UTR using extracts from untreated Müller cells (Table 2) and shown to function under redox conditions. Although experimental approaches and binding conditions differed from the study of Kuwano [94], this indicates that the binding affinity of ILF3 to target AREs may be influenced by cell-type and/or species specificities.

In contrast to ILF3, the contribution of KHSRP to the regulation of *Lif* mRNA expression in Müller cells may not depend on redox signaling (Figure 7). Similar to the inhibition of p38 MAPK, siRNA-mediated knockdown of *Khsrp* severely reduced *Lif* mRNA levels suggesting that KHSRP is required to stabilize *Lif* transcripts under normal conditions. This result was unexpected, since KHSRP has been shown to negatively regulate stability of its target mRNAs [71,96]. Although KHSRP was detected in our MS analysis, it did not show preferential binding to either wild type or mutant sequences (dataset PXD001463 on the ProteomeXchange Consortium platform [61]). Thus, it is possible that the reduction of *Lif* mRNA levels in the absence of KHSRP may be independent of AREs in regions 27 and/or 36 of the 3′UTR. Since KHSRP has been shown to promote maturation of miRNAs [73], KHRSP may affect *Lif* mRNA levels indirectly, potentially by modulating non-coding RNAs involved in *Lif* regulation. Although miRNAs are generally believed to destabilize target mRNAs, evidence has been presented that individual miRNAs may also increase the stability of targets [97]. Thus, absence of KHSRP may prevent maturation of specific non-coding RNAs required for *Lif* mRNA stabilization under normal conditions. Alternatively, KHSRP may act through an interaction with the p38 MAPK pathway [71] since both inactivation of KHSRP and inhibition of p38 MAPK had similar consequences for *Lif* mRNA levels under normal conditions.

Our results highlight important aspects of *Lif* gene expression, which may impact on retinal physiology and pathophysiology. Specifically, regulatory proteins identified here may provide attractive targets for the modulation of LIF synthesis in retinal degenerative diseases since a moderate elevation of endogenous *Lif* expression may be neuroprotective and support photoreceptor survival (CA, unpublished data). Our data may also impact on stem cell biology since *Lif* is a pleiotropic factor that contributes to stem cell renewal *in vivo* and maintenance of pluripotency of mouse stem cells in culture [98,99]. Interestingly, Müller cells act as stem cells to regenerate retinal neurons in fish [100] and attempt (but fail) to do likewise in mouse [101-103]. Thus, a proper adjustment of *Lif* signaling *in vivo* may influence the capability of mammalian Müller cells to act as progenitors in retinal disease. Clearly, *in vivo* experiments are warranted to test whether modulating the components of LIF regulation may affect neuroprotection and/or impact on the stem cell potential of Müller glia cells in the mouse retina and may thus improve disease outcome and vision.

## Conclusions

Neuroprotection through the generation of ROS by injured neurons has long been a controversial issue in neurodegenerative diseases. Here, we show that H_2_O_2_ can act as a messenger to regulate expression of the neuroprotective gene *Lif* in stressed Müller glia. Redox dependent increase in expression is achieved by the modulation of *Lif* mRNA stability through an *Ilf3* dependent pathway and conserved AREs in the 3′UTR. Therefore, injury-induced production of ROS by retinal neurons leads to stabilization of *Lif* mRNA that may result in a more sustained expression when LIF signaling is necessary to preserve neurons.

## Methods

### Cell culture, serum deprivation, H_2_O_2_, ActD, TNF and p38 MAPK inhibitor treatment

The rat Müller glia cell line rMC-1 [48] was obtained from Dr. Sarthy (Northwestern University, Chicago, USA, IL). Cells were cultured in Dulbecco’s modified Eagle’s medium (DMEM; Life Technologies, Grand Island, NY, USA) supplemented with 10% fetal bovine serum (FBS; Life Technologies), 100 U/ml penicillin and 100 μg/ml streptomycin (Life Technologies), and grown in a humidified 5% CO_2_ incubator as described [21]. A total of 300,000 cells in 2 ml growth media were seeded on a six-well plate and cultured overnight. For serum deprivation, rMC-1 cells were washed once with warm PBS and growth media without serum was added. Then, 30% H_2_O_2_ (Sigma Aldrich, St. Louis, MO, USA) was diluted in DMEM with or without FBS to a final concentration of 50 μM and added to PBS-washed cells. ActD (Sigma Aldrich) was diluted in DMEM with or without FBS/H_2_O_2_ to a final concentration of 10 μg/ml. Rat recombinant TNF (R&D systems, Minneapolis, MN, USA) and the p38 MAPK inhibitor SB202190 (Sigma Aldrich) [104] were dissolved in 0.1% bovine serum albumin or in dimethyl sulfoxide (DMSO), respectively. TNF and SB202190 were added directly to growth media to reach final concentrations of 10 ng/μl (TNF) and 100 μM (SB202190). Cells were collected at time points indicated in the results.

### RT-PCR analysis

Total RNA was extracted from rMC-1 or primary Müller cells using the Megamax RNA isolation kit (Life Technologies) according to the manufacturer’s instructions. cDNA was prepared using the high capacity cDNA reverse transcription kit (Life Technologies). RT-PCR reactions were conducted using appropriate primer pairs (Additional file 8: Table S1). All primer pairs were checked for their amplification efficiency using serial dilutions of template and for the generation of a single amplicon of the correct size. *Actb* was used as internal control. Additional internal controls, *Gapdh* and *Rpl32*, were used for each new treatment. RT-PCR reactions were performed in a StepOne Real-Time PCR system with Fast SybrGreen master mix (Life Technologies) or a LightCycler 480 instrument with SybrGreen I Master mix (Roche, Basel, Switzerland). The comparative cycle threshold method was used to calculate relative transcript levels. Raw PCR data are presented in Additional file 9: File S3. N-values reflect independent experiments.

### Primary mouse Müller cells (Müller cell enriched primary retinal cell culture)

Animal experimentation protocols were accepted by the Veterinary Authorities of Zurich and experiments adhered to the statement of ‘The Association for Research in Vision and Ophthalmology’ for the use of animals in research. *Ttp*^*−/−*^ mice [105] and *Rlbp-GFP* transgenic mice [106] expressing GFP specifically in Müller glia cells were generously provided by Dr. Thomas Rülicke (University of Veterinary Medicine Vienna, Austria) and Dr. Edward M. Levine (University of Utah, Salt Lake City, UT, USA), respectively. *Rlbp-GFP;Ttp*^*−/−*^ and *Rlbp-GFP;Ttp*^*+/−*^ pups were euthanized by a CO_2_ overdose and decapitated between P8 and P12, and retinas were isolated. Retinal cells were dissociated according to the protocol by Siegert and colleagues [107]. Dissociated cells were centrifuged for five minutes at 50 × g to increase the relative ratio of large GFP-positive cells. Cells from two retinas of individual mice were subdivided into eight wells for further treatments. Retinal cells in each well were cultured in 2 ml media for two weeks with a media change every two days using the same conditions as for the rMC-1 cells except that 25 mM D-sorbitol (Sigma-Aldrich) instead of glucose was used for Müller cell enrichment [108]. Unlike adult Müller cells, GFP expression in P8-12 Müller cells was weak and upon attachment, GFP expression declined further, a phenomenon possibly due to proliferation of primary Müller cells or loss of cellular connections and increased surface area in culture. Müller cells proliferated and reached confluence generally after about two weeks.

### Sequence alignment and ARE identification

Alignment of 3’UTR sequences from *Lif* genes of various mammals and identified highly conserved regions were retrieved from Vista pre-computed whole-genome alignments [56,57]. The mouse *Lif* 3′UTR sequence (NM_008501.2) was manually scanned for AU-rich regions according to Hao *et al.* and Caput *et al.* [50,109]. To qualify as an ARE, AUUU sequences needed to be accompanied by at least three additional A or U residues. In cases where several AUUU sequences were spaced by less than 3 bps, we checked for the presence of an AUUUA core motif. Putative micro RNA binding sites were identified using Targetscan software [58].

### Cloning and site directed mutagenesis

PGL4.12[luc2CP] vector (Promega, Madison, WI, USA) was restriction digested with HindIII and XbaI to isolate *luc2CP*. The isolated *luc2CP* fragment (containing hCP1 and hPEST) was cloned into pGL3 control vector (Promega,) containing SV40 promoter and enhancer for robust expression, as Luc2CP luminescence was barely detectable in normal expression vectors due to the highly unstable nature of the protein. The resulting vector (ΔUTR) had a minimal 3′UTR and was used for further cloning. Mouse whole *Lif* 3′UTR (source: BAC clone RP23-451O6 (Children’s Hospital Oakland Research Institute, Oakland, CA, USA)), AU-rich region I, human β-Actin (*ACTB*) 3′UTR and human genomic sequences (alternate assembly CHM1_1.1, chr2:122988136–122991277) were PCR amplified and cloned into ΔUTR vector using the XbaI restriction site. Similarly, individual mouse *Lif* 3′UTR fragments were PCR amplified and cloned into the ΔUTR vector using XbaI and PstI restriction sites with the exception of AU-rich region II and region 27. These two regions were generated by restriction digestion of the plasmid containing the whole *Lif* 3′UTR using PstI-XcmI and PstI-EcoNI enzyme combinations for AU-rich region II and region 27, respectively. Overhanging ends of digested plasmids were blunted using Klenow enzyme and religated.

Site-directed mutagenesis was done according to instructions provided by the QuikChange Lightning Multi Site-Directed Mutagenesis kit (Agilent, Santa Clara, CA, USA). ARE core sequences were replaced by restriction digestion sites for identification of clones. During the mutation of element B (plasmid 27 ‘2 ARE’) within region 27, multiple ARE containing plasmids were generated as byproducts and used to test for the effects of increased numbers of ARE elements. Plasmid ‘27-B’ was generated by restriction digestion of plasmid ‘27-No’ with SacII and SpeI that was generated during mutation of elements A and C. Element B was introduced back to digested plasmid ‘27-No’ by annealed primers that contained element B and appropriate overhangs. Primers used for cloning are listed in Additional file 10: Table S2.

### Luciferase assay

rMC-1 cells were transfected with constructs containing 3′UTR sequences fused to *Luc2CP* firefly luciferase as described previously [21]. Renilla luciferase expressing vector, pRL-CMV (Promega), was used as internal control. Transfected rMC-1 cells were cultured for 24 hours and luciferase levels were measured using the Dual-Luciferase kit (Promega). Firefly/renilla luciferase ratios were calculated and expressed relative to the respective control. Each construct was tested in triplicate in three to four independent experiments.

### *In vitro* transcription and 3′ biotin labeling

RNAs used for capturing RNA binding proteins from cell extracts were generated by *in vitro* transcription (Maxiscript kit; Life Technologies) using PCR amplified templates from region 27, region 36 and their respective ARE-null counterparts. Primers used for amplification of templates are listed in Additional file 10: Table S2. RNA probes were labeled with biotin at the 3′ end using the RNA 3′ End Biotinylation Kit (Pierce, Rockford, IL, USA). All procedures were followed according to the instructions from the manufacturers.

### Quantitative mass spectrometry analysis and GO-pathway analysis

rMC-1 cells were grown in SILAC DMEM (GE Healthcare Life Sciences, Pittsburgh, PA, USA) supplemented with 3 mM L-glutamine (GE Healthcare Life Sciences), 10% dialyzed fetal bovine serum (GE Healthcare Life Sciences) and 0.55 mM lysine, 0.4 mM arginine. Light SILAC medium was supplemented with ^12^C6, ^14^N2 lysine and ^12^C6, ^14^N4 arginine. Heavy SILAC medium was supplemented with either ^13^C6 lysine and ^13^C6, ^15^N4 arginine or ^13^C6, ^15^N2 lysine and ^13^C6, ^15^N4 arginine. A total of 0.5mM proline was added to all SILAC media to prevent arginine to proline conversion. All amino acids were purchased from Silantes (Munich, Germany).

Biotin-labeled RNA (2 μg) was bound to Strep-tactin beads (IBA, Goettingen, Germany) in RNA binding buffer (150 mM NaCl, 50 mM Hepes-HCl pH 7.5, 0.5% NP40 (v/v), 10 mM MgCl_2_, Phosphatase Inhibitor Cocktail 2 and 3 (Sigma-Aldrich)) and incubated on a rotation wheel at 4°C. Beads were washed three times with RNA wash buffer containing 150 mM NaCl, 50 mM Hepes-HCl pH 7.5, 0.1% NP40 and 10 mM MgCl_2_ and Phosphatase Inhibitor Cocktail 2 and 3 (Sigma-Aldrich) before incubation at 4°C for 30 minutes with 2 mg of cytoplasmic extract, 200 units RNase inhibitor (Fermentas, Schwerte, Germany) and 20 μg yeast tRNA. After incubation, the corresponding samples were combined and the beads were washed another three times with RNA wash buffer before the protein/RNA complexes were eluted from the beads with Laemmli buffer. The eluted proteins were subjected to gel-based pre-fractionation and tryptic cleavage as described elsewhere [110,111].

Liquid chromatography-tandem mass spectrometry (LC-MS/MS) analysis was performed on an Ultimate3000 nano RSLC system (Thermo Fisher Scientific, Waltham, MA USA) coupled to a LTQ Orbitrap Velos mass spectrometer (Thermo Fisher Scientific) by a nano spray ion source. Tryptic peptide mixtures were automatically injected and separated by a linear gradient from 5% to 40% of buffer B (2% acetonitrile, 0.1% formic acid in HPLC grade water) in buffer A (0.1% formic acid in HPLC grade water) at a flow rate of 300 nl/minute over 90 minutes. Remaining peptides were eluted by a short gradient from 40% to 100% buffer B in five minutes. The eluted peptides were analyzed by the LTQ Orbitrap Velos mass spectrometer. From the high resolution MS pre-scan with a mass range of 300 to 1,500, the ten most intense peptide ions were selected for fragment analysis in the linear ion trap if they exceeded an intensity of at least 500 counts and if they were at least doubly charged. The normalized collision energy for CID was set to a value of 35 and the resulting fragments were detected with normal resolution in the linear ion trap. The lock mass option was activated, the background signal with a mass of 445.12002 was used as lock mass 5. Every ion selected for fragmentation was excluded for 20 seconds by dynamic exclusion.

All acquired spectra were processed and analyzed using the MaxQuant software 6 (version 1.3.0.5) and the human specific IPI database version 3.52 [112] in combination with Mascot (Matrix Science, version 2.2). Cysteine carbamidomethylation was selected as fixed modification, and methionine oxidation and protein acetylation were allowed as variable modifications. The peptide and protein false discovery rates were set to 1%. Contaminants, such as keratins, were removed. Proteins, identified and quantified by at least two unique peptides were considered for further analysis. The significance values were determined by Perseus tool (part of MaxQuant) using significance A [112]. GO pathway analyses were done using the web-based Gene Set Analysis Toolkit [113,114].

### siRNA transfection

rMC-1 cells were seeded on six-well plates (50,000 cells per well, 2 ml growth medium). After 24 hours at 37°C and 5% CO_2_, rMC-1 cells were transfected with siRNA using RNAiMax (Life Technologies) and 80 pmol of specific siRNA oligonucleotides (Additional file 11: Table S3; Qiagen, Hilden, Germany) or AllStars Negative Control siRNA (Qiagen) according to the manufacturer’s instructions. Cells were used for experiments 48 hours after transfection.

### Western blotting

rMC-1 cells were lysed in 200 μl 2 × Laemmli sample buffer. A total of 30 μl of the homogenate was separated on 10% SDS-polyacrylamide gels, blotted and probed as described previously [13]. Primary antibodies for ELAVL1 (1:2,000, cat# sc-5261, Santa Cruz, Dallas, TX, USA) and ILF3 (1:1,000, cat# 19887-1-AP, Proteintech, Manchester, UK) were applied over night at 4°C. The secondary antibody (1:10,000, peroxidase-linked anti-rabbit immunoglobulin G (IgG), cat# NA934; GE Healthcare) was applied for one hour at room temperature. We have used WesternBright Sirius horseradish peroxidase (HRP) substrate (Advansta, Menlo Park, CA, USA) for chemiluminescence reaction. Fusion FX7 Advance imaging system (Vilber Lourmat, Torcy, France) with a CCD camera was used for digital signal detection. Recordings were taken at the dynamic range of exposure without binning. Calculations for expression levels were performed using BioD1 software (Vilber Lourmat) without background subtractions. Signals for ACTB served as controls.

For Western blotting experiments on SD, H_2_O_2_, SD + H_2_O_2_, TNF or SB treated rMC-1 cells (Additional file 2: Figure S2), the same procedures were applied except for the detection system and quantification method. Briefly, X-ray film-based detection was followed by Image J quantification relative to ACTB or unphosphorylated p38 MAPK levels. The following primary antibodies and dilutions were used: p38 MAPK (cat# 9212, 1:1,000, Cell Signaling, Danvers, MA, USA), phospho-p38 MAPK (cat# 9211, 1:1,000, Cell Signaling); phospho-HSP27 (cat# 2401P, 1:1,000, Cell Signaling); phospho-MKK3/6 (cat# 9231S, 1:1,000, Cell Signaling); ACTB (cat# A5441, 1:5,000, Sigma-Aldrich). Peroxidase-linked anti-mouse IgG (cat# sc-2031, Santa Cruz) was used at a dilution of 1:10,000 as secondary antibody.

### Immunofluorescence

129S6 wild type mice were or were not exposed to two hours of 15,000 lux of white light as described [115]. Twenty-four hours after light exposure, mice were euthanized, eyes enucleated and fixed in 4% paraformaldehyde (PFA) prepared in phosphate buffered saline (PBS; pH 7.4), as described previously [116]. Cryosections (12 μm) were blocked for one hour with 3% normal goat serum (containing 0.3% Triton X-100 in PBS), and incubated overnight at 4°C with rabbit anti-ILF3 (1:100; cat# 19887-1-AP, Proteintech, Manchester, UK), rabbit anti-KHSRP (1:250; cat# NBP1-18910, Novus Biologicals, Cambridge, UK) or mouse anti-glutamine synthetase (1:500; cat# MAB302, Millipore, Darmstadt, Germany) primary antibodies. Slides were washed three times with PBS and incubated with Cy2-labeled secondary anti-rabbit or Cy3-labeled secondary anti-mouse antibodies (Jackson ImmunoResearch Laboratories, Soham, UK), counterstained with 4',6-diamidino-2-phenylindole (DAPI) and analyzed by fluorescence microscopy (Axioplan 2; Carl Zeiss AG, Feldbach, Switzerland).

### Data analysis

Statistical analysis was performed using ANOVA with appropriate posttests (see Figure legends) for multiple comparisons. Student's *t*-tests were used for individual pairwise comparisons. *P* values less than 0.05 were considered to indicate significant differences. Error bars represent the standard error of the mean (SEM). Graph Pad 6 software (GraphPad Inc., San Diego, USA) was used for all statistical analyses.

## Additional files

Additional file 1: Figure S1. rMC-1 cells were (+) or were not (−) treated with H_2_O_2_, ActD and/or TNF for one hour as indicated. *Lif* RNA levels were determined by real-time PCR and expressed relative to untreated controls. Shown are means ± SEM of *N* = 3. One-way ANOVA with Sidak’s posttests were used to compare identical treatments in the absence or presence of H_2_O_2_. (*) *P* <0.05 and (****) *P* < 0.0001. H_2_O_2_ did not increase *Lif* mRNA levels in rMC-1 cells under normal, non-stressing conditions. Addition of TNF to non-stressed cells strongly increased *Lif* levels as published before [21]. This increase depended on active gene transcription since addition of ActD prevented TNF-induced elevation of *Lif* mRNA levels. Since ActD still repressed TNF-induced *Lif* expression after addition of H_2_O_2_, ActD remained active in the presence of H_2_O_2_. Additional file 2: Figure S2.
**A**) rMC-1 cells were not (Ctrl) or were treated with H_2_O_2_, SD, SD + H_2_O_2_, TNF or SB202190 (SB) for one hour as indicated. Protein homogenates were prepared and used in Western blotting experiments to test protein levels as indicated. Representative blots are shown. **B**) Quantification of signals detected by Western blotting. Shown are means ± SEM of *N* = 3. One-way ANOVA with Dunnett’s posttests were used to compare no treatment with other treatments for each protein. (**) *P* <0.01. Additional file 3: Figure S3.
**A**) Sequence analysis of the 3,180 bp long *Lif* 3’UTR predicted 11 potential AU-rich elements (AREs; A-K) and highly conserved binding sites for miRNA-29 and miRNA-17 (purple lines). AREs indicated by red and AUUU sequence elements by green lines. Individual AU-rich regions (indicated on the right) are color coded and their boundaries marked with brackets in the sequence. **B**) Experimental design of luciferase reporter constructs to test the effect of potential AREs on reporter expression. Top: schematic representation of the endogenous *Lif* gene. Bottom: schematic representation of the reporter construct containing: SV40-driven *Luciferase* reporter gene fused to hCL1 and hPEST sequences for protein destabilization and various *Lif* 3′UTR sequences. Additional file 4: Figure S4.
**A-C**) Sequences of the constructs used in the respective experiments shown in Figure 5A-C. AREs (A, B, C, D, E) are indicated with red lines and the putative miR-29 binding site is marked with a green line. Mutated sequences are indicated in blue. Additional file 5: Figure S5.
**A**) Schematic diagram of enriched pathways determined by GO pathway analysis of proteins identified with region 27. The blue square marks proteins involved in cell redox homeostasis. The blue circle marks RNA binding proteins. **B**) Schematic diagram of enriched pathways determined by GO pathway analysis of proteins identified with region 36. The blue circle marks RNA binding proteins. Individual proteins targeting regions 27 or 36, and their respective pathways are listed in Additional file 6: File 1 and Additional file 7: File 2, respectively. Hypergeometric test with BH posttest was performed to test for significance. Significance values are listed in the schematic diagram for each pathway. Additional file 6: File S1 Analysis of gene sets using the WEB-based Gene Set Analysis Toolkit. Genes encoding for proteins identified using R27 are listed according to their biological processes, molecular function, and cellular component. Links are provided to EntrezGene and Ensembl data bases for each gene. (PDF 674 kb) Additional file 7: File S2. Analysis of gene sets using the WEB-based Gene Set Analysis Toolkit. Genes encoding for proteins identified using R36 are listed according to their biological processes, molecular function, and cellular component. Links are provided to EntrezGene and Ensembl data bases for each gene. Additional file 8: Table S1. Real-time PCR primer sequences. Sequences of primers used for real-time PCR. Primers used for both rat and mouse samples are marked (Rn/Mm). *Rn*, *Rattus norvegicus*; *Mm*, *Mus musculus.*
Additional file 9: File S3. Raw real-time PCR data. Additional file 10: Table S2 Primer sequences used for cloning and site-directed mutagenesis. Sequences of primers used for cloning, site-directed mutagenesis and templates for *in vitro* transcription. Additional file 11: Table S3. siRNA sequences. Target sequences of siRNAs used to knockdown respective genes in rMC-1 cells.
