# Supplementary figures and images for: Expression of leukemia inhibitory factor in Müller glia cells is regulated by a redox-dependent mRNA stability mechanism
Source: BMC Biol. 2015 Apr 25;13:30. doi: 10.1186/s12915-015-0137-1 (PMC4462110; doi:10.1186/s12915-015-0137-1)

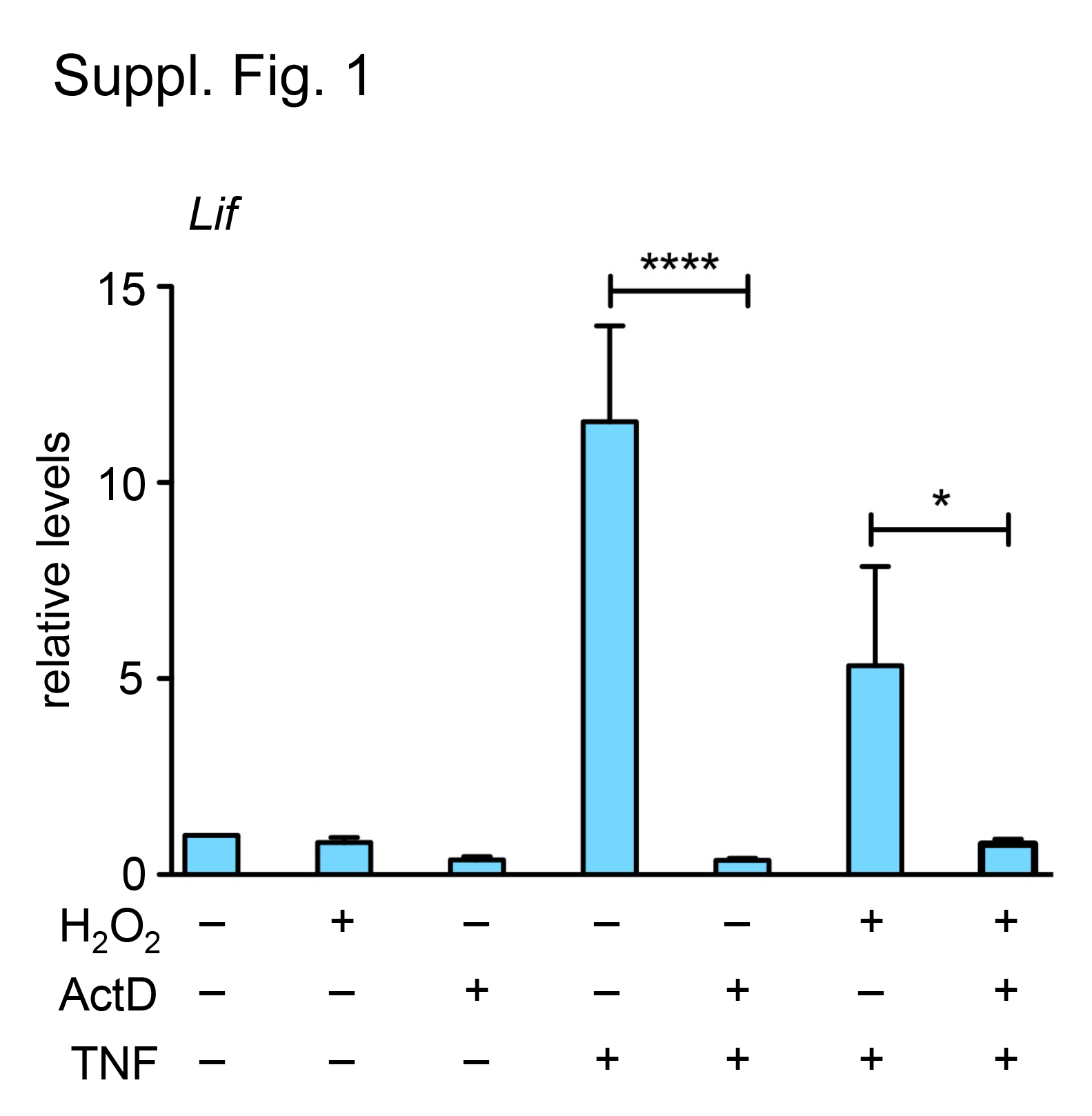

Supplement: Additional file 1: Figure S1. — rMC-1 cells were (+) or were not (−) treated with H2O2, ActD and/or TNF for one hour as indicated. Lif RNA levels were determined by real-time PCR and expressed relative to untreated controls. Shown are means ± SEM of N = 3. One-way ANOVA with Sidak’s posttests were used to compare identical treatments in the absence or presence of H2O2. (*) P <0.05 and (****) P < 0.0001. H2O2 did not increase Lif mRNA levels in rMC-1 cells under normal, non-stressing conditions. Addition of TNF to non-stressed cells strongly increased Lif levels as published before [21]. This increase depended on active gene transcription since addition of ActD prevented TNF-induced elevation of Lif mRNA levels. Since ActD still repressed TNF-induced Lif expression after addition of H2O2, ActD remained active in the presence of H2O2. [file 12915_2015_137_MOESM1_ESM.tiff]

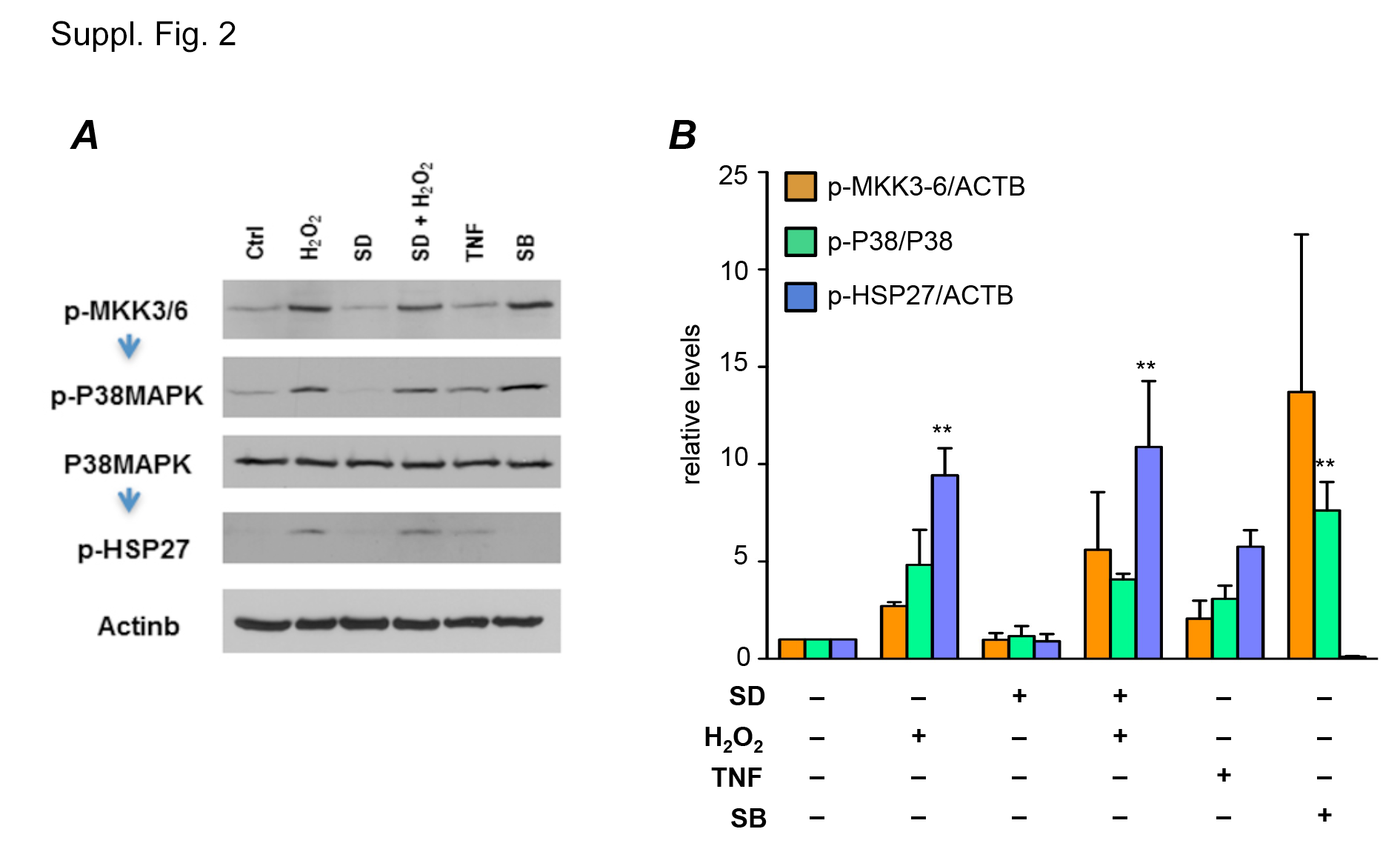

Supplement: Additional file 2: Figure S2. — A) rMC-1 cells were not (Ctrl) or were treated with H2O2, SD, SD + H2O2, TNF or SB202190 (SB) for one hour as indicated. Protein homogenates were prepared and used in Western blotting experiments to test protein levels as indicated. Representative blots are shown. B) Quantification of signals detected by Western blotting. Shown are means ± SEM of N = 3. One-way ANOVA with Dunnett’s posttests were used to compare no treatment with other treatments for each protein. (**) P <0.01. [file 12915_2015_137_MOESM2_ESM.tiff]

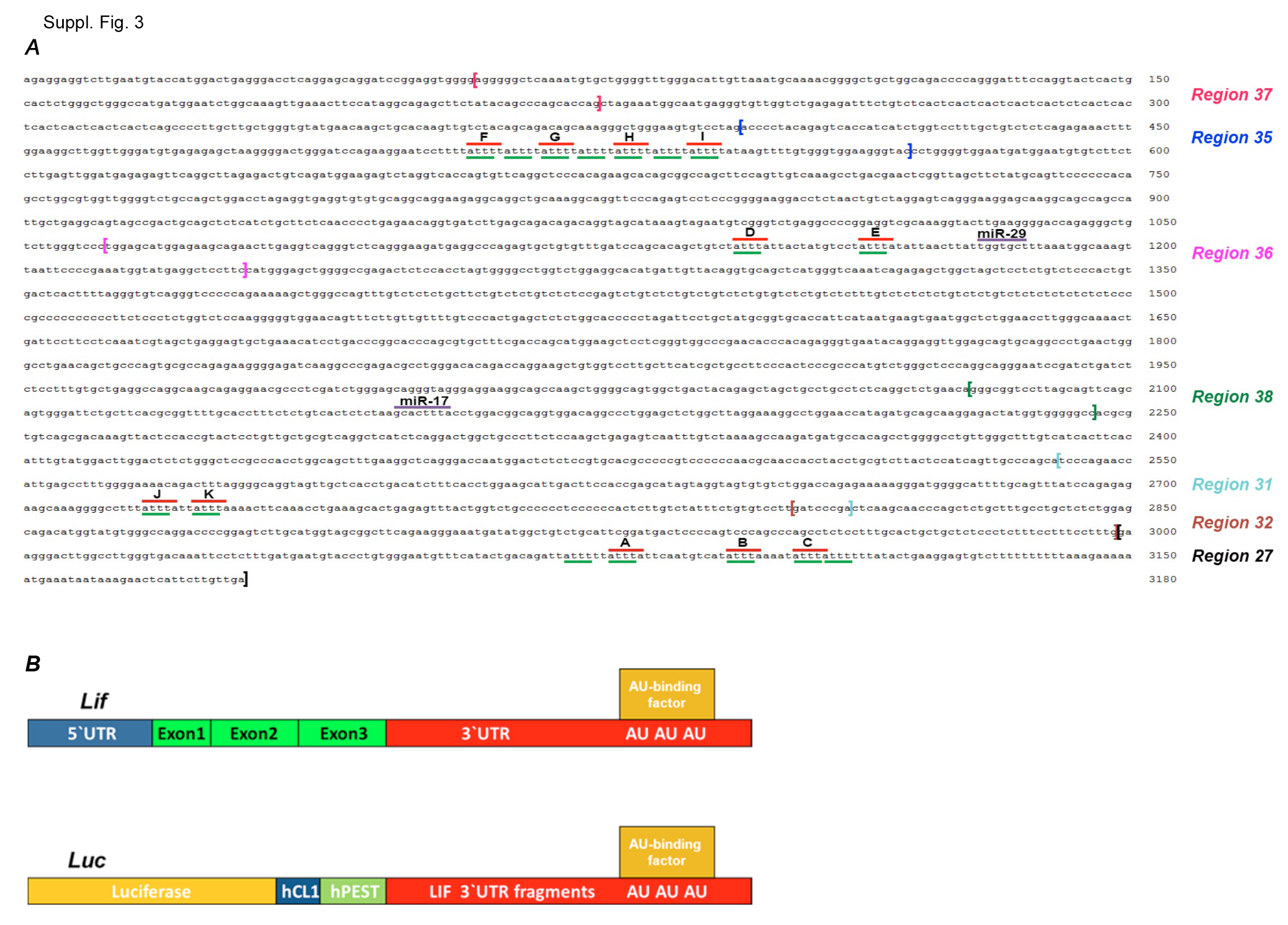

Supplement: Additional file 3: Figure S3. — A) Sequence analysis of the 3,180 bp long Lif 3’UTR predicted 11 potential AU-rich elements (AREs; A-K) and highly conserved binding sites for miRNA-29 and miRNA-17 (purple lines). AREs indicated by red and AUUU sequence elements by green lines. Individual AU-rich regions (indicated on the right) are color coded and their boundaries marked with brackets in the sequence. B) Experimental design of luciferase reporter constructs to test the effect of potential AREs on reporter expression. Top: schematic representation of the endogenous Lif gene. Bottom: schematic representation of the reporter construct containing: SV40-driven Luciferase reporter gene fused to hCL1 and hPEST sequences for protein destabilization and various Lif 3′UTR sequences. [file 12915_2015_137_MOESM3_ESM.tiff]

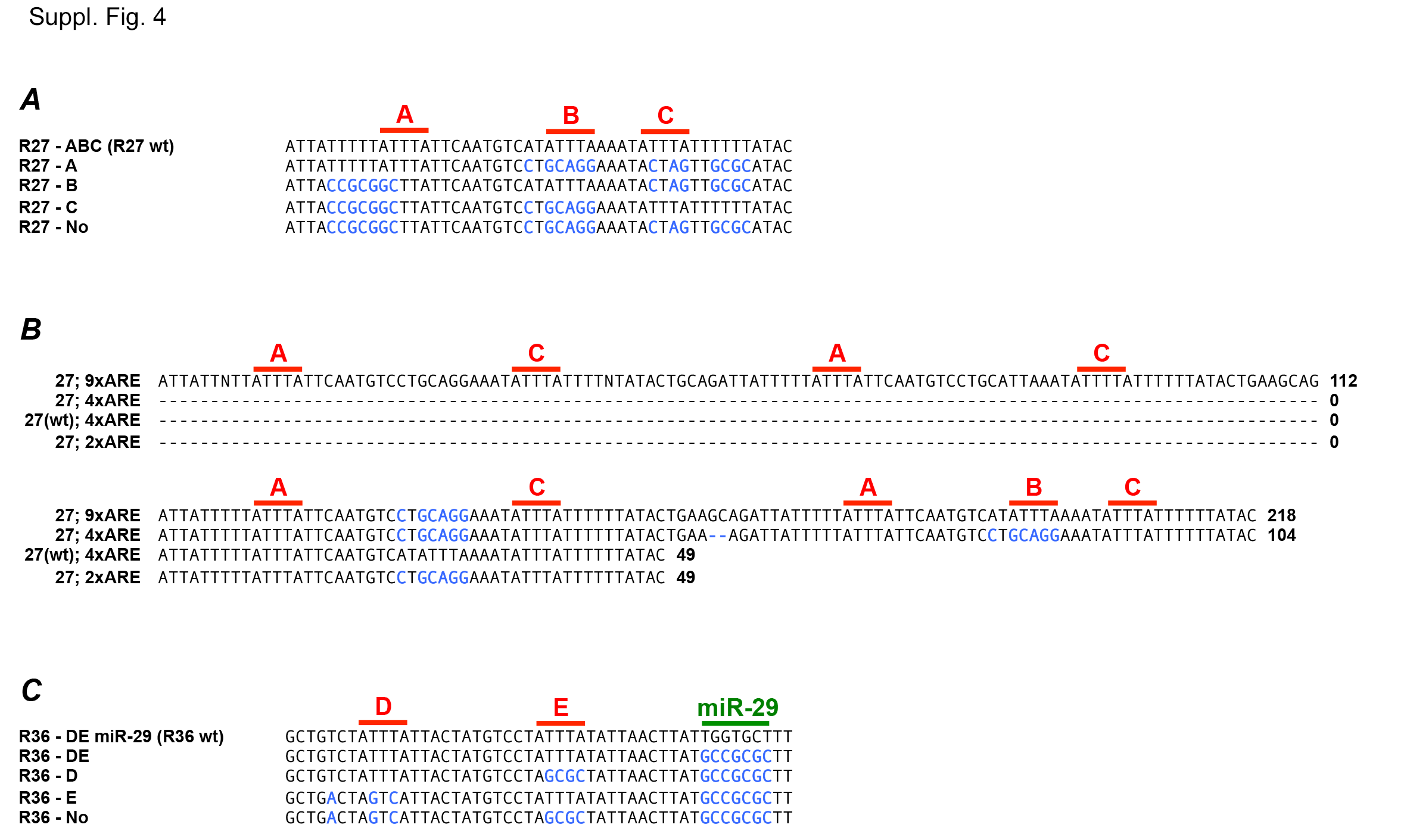

Supplement: Additional file 4: Figure S4. — A-C) Sequences of the constructs used in the respective experiments shown in Figure 5A-C. AREs (A, B, C, D, E) are indicated with red lines and the putative miR-29 binding site is marked with a green line. Mutated sequences are indicated in blue. [file 12915_2015_137_MOESM4_ESM.tiff]

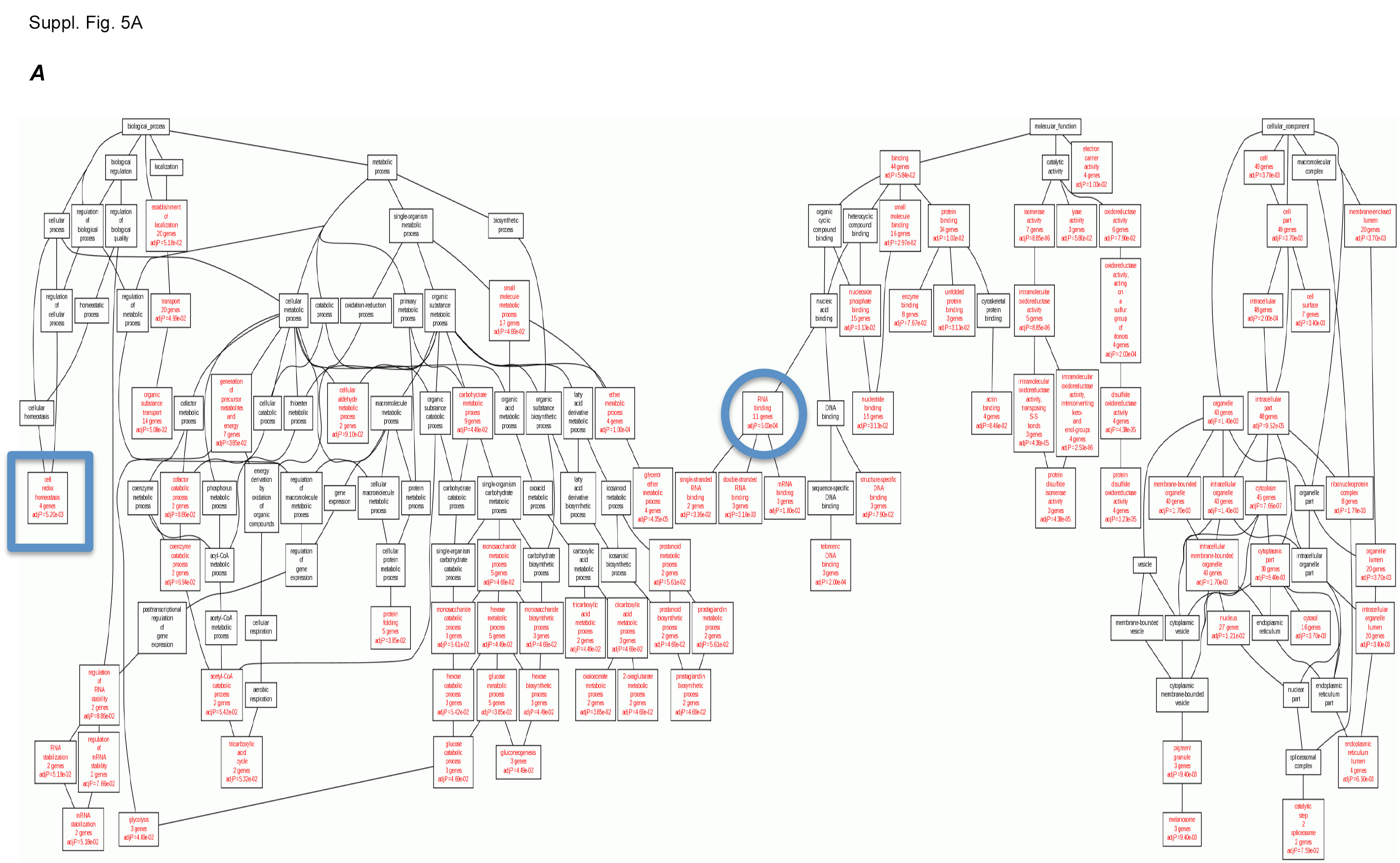

Supplement: Additional file 5: Figure S5. — A) Schematic diagram of enriched pathways determined by GO pathway analysis of proteins identified with region 27. The blue square marks proteins involved in cell redox homeostasis. The blue circle marks RNA binding proteins. B) Schematic diagram of enriched pathways determined by GO pathway analysis of proteins identified with region 36. The blue circle marks RNA binding proteins. Individual proteins targeting regions 27 or 36, and their respective pathways are listed in Additional file 6: File 1 and Additional file 7: File 2, respectively. Hypergeometric test with BH posttest was performed to test for significance. Significance values are listed in the schematic diagram for each pathway. [file 12915_2015_137_MOESM5_ESM.zip › 6376898291560173_add5.tiff]

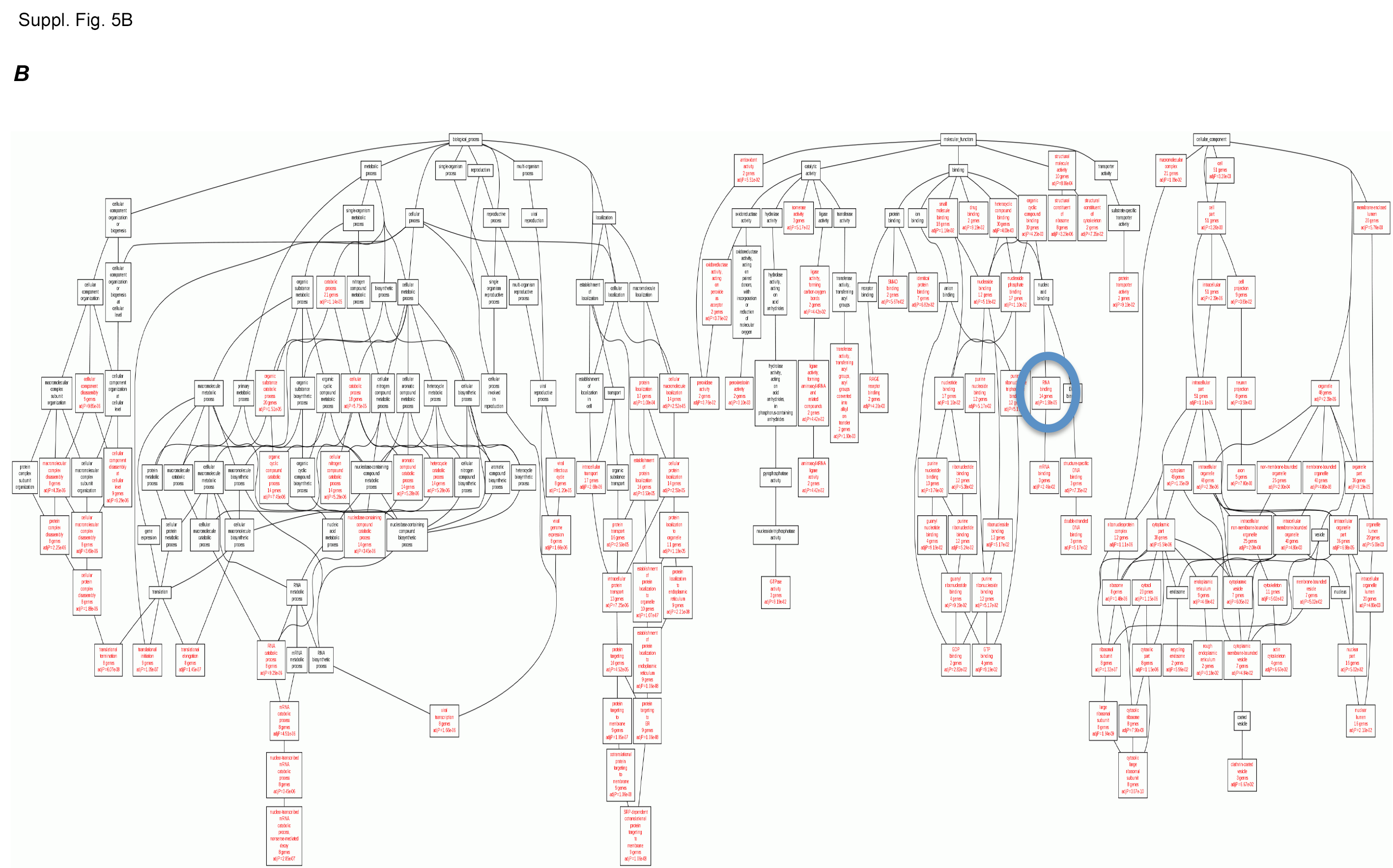

Supplement: Additional file 5: Figure S5. — A) Schematic diagram of enriched pathways determined by GO pathway analysis of proteins identified with region 27. The blue square marks proteins involved in cell redox homeostasis. The blue circle marks RNA binding proteins. B) Schematic diagram of enriched pathways determined by GO pathway analysis of proteins identified with region 36. The blue circle marks RNA binding proteins. Individual proteins targeting regions 27 or 36, and their respective pathways are listed in Additional file 6: File 1 and Additional file 7: File 2, respectively. Hypergeometric test with BH posttest was performed to test for significance. Significance values are listed in the schematic diagram for each pathway. [file 12915_2015_137_MOESM5_ESM.zip › 6376898291560173_add6.tiff]
