# Supplementary material for: Expression of leukemia inhibitory factor in Müller glia cells is regulated by a redox-dependent mRNA stability mechanism
Source: BMC Biol. 2015 Apr 25;13:30. doi: 10.1186/s12915-015-0137-1 (PMC4462110; doi:10.1186/s12915-015-0137-1)
Supplement: Additional file 6: File S1 — Analysis of gene sets using the WEB-based Gene Set Analysis Toolkit. Genes encoding for proteins identified using R27 are listed according to their biological processes, molecular function, and cellular component. Links are provided to EntrezGene and Ensembl data bases for each gene. (PDF 674 kb) [file 12915_2015_137_MOESM6_ESM.pdf]

WEB-based GEne SeT AnaLysis Toolkit

WebGestalt *Translating gene lists into biological insights...*

User data and parameters: User data: textAreaUpload.txt, Organism: hsapiens, Id Type: uniprot_swissprot_accession, Ref Set: entrezgene_protein-coding, Significance Level: .1, Statistics Test: Hypergeometric, MTC: BH, Minimum: 2

The results for the enriched GO category are listed in this table. For each GO category, the first row lists its sub-root (biological process, molecular function, or cellular component), category name, and corresponding GO ID.
The second row lists the following statistics:

- C: the number of reference genes in the category
- O: the number of genes in the gene set and also in the category
- E: the expected number in the category
- R: ratio of enrichment
- rawP: p value from hypergeometric test
- adjP: p value adjusted by the multiple test adjustment

Finally, genes in the category are listed. For each gene, the table lists the user uploaded ID and value (optional), Entrez ID, Ensembl Gene Stable ID, Gene symbol, and description. Ensembl Gene Stable ID and Entrez Gene ID are linked to the Ensembl and Entrez Gene databases, respectively.

**Database:biological process Name:glycerol ether metabolic process ID:GO:0006662**						
C=14; O=4; E=0.05; R=87.67; rawP=9.68e-08; adjP=4.35e-05						
Index	UserID	Value	Gene Symbol	Gene Name	EntrezGene	Ensembl
1 ☐	P30101	NA	PDIA3	protein disulfide isomerase family A, member 3	2923	ENSG00000167004
2 ☐	P10599	NA	TXN	thioredoxin	7295	ENSG00000136810
3 ☐	Q8NBS9	NA	TXNDC5	thioredoxin domain containing 5 (endoplasmic reticulum)	81567	ENSG00000239264
4 ☐	P13667	NA	PDIA4	protein disulfide isomerase family A, member 4	9601	ENSG00000155660

**Database:biological process Name:ether metabolic process ID:GO:0018904**						
C=21; O=4; E=0.07; R=58.45; rawP=5.69e-07; adjP=0.0001						
Index	UserID	Value	Gene Symbol	Gene Name	EntrezGene	Ensembl
1 ☐	P30101	NA	PDIA3	protein disulfide isomerase family A, member 3	2923	ENSG00000167004
2 ☐	P10599	NA	TXN	thioredoxin	7295	ENSG00000136810
3 ☐	Q8NBS9	NA	TXNDC5	thioredoxin domain containing 5 (endoplasmic reticulum)	81567	ENSG00000239264
4 ☐	P13667	NA	PDIA4	protein disulfide isomerase family A, member 4	9601	ENSG00000155660

Database:biological process Name:ether metabolic process ID:GO:0018904						
C=21; O=4; E=0.07; R=58.45; rawP=5.69e-07; adjP=0.0001						
Index	UserID	Value	Gene Symbol	Gene Name	EntrezGene	Ensembl

Database:biological process Name:cell redox homeostasis ID:GO:0045454						
C=57; O=4; E=0.19; R=21.53; rawP=3.45e-05; adjP=0.0052						
Index	UserID	Value	Gene Symbol	Gene Name	EntrezGene	Ensembl
1 ☐	P30101	NA	PDIA3	protein disulfide isomerase family A, member 3	2923	ENSG00000167004
2 ☐	P10599	NA	TXN	thioredoxin	7295	ENSG00000136810
3 ☐	Q8NBS9	NA	TXNDC5	thioredoxin domain containing 5 (endoplasmic reticulum)	81567	ENSG00000239264
4 ☐	P13667	NA	PDIA4	protein disulfide isomerase family A, member 4	9601	ENSG00000155660

Database:biological process Name:oxaloacetate metabolic process ID:GO:0006107						
C=10; O=2; E=0.03; R=61.37; rawP=0.0005; adjP=0.0385						
Index	UserID	Value	Gene Symbol	Gene Name	EntrezGene	Ensembl
1 ☐	P00505	NA	GOT2	glutamic-oxaloacetic transaminase 2, mitochondrial (aspartate aminotransferase 2)	2806	ENSG00000125166
2 ☐	O75390	NA	CS	citrate synthase	1431	ENSG00000062485

Database:biological process Name:protein folding ID:GO:0006457						
C=211; O=5; E=0.69; R=7.27; rawP=0.0006; adjP=0.0385						
Index	UserID	Value	Gene Symbol	Gene Name	EntrezGene	Ensembl
1 ☐	P30101	NA	PDIA3	protein disulfide isomerase family A, member 3	2923	ENSG00000167004
2 ☐	Q99471	NA	PFDN5	prefoldin subunit 5	5204	ENSG00000123349
3 ☐	Q15185	NA	PTGES3	prostaglandin E synthase 3 (cytosolic)	10728	ENSG00000110958
4 ☐	P30040	NA	ERP29	endoplasmic reticulum protein 29	10961	ENSG00000089248

Database:biological process Name:protein folding ID:GO:0006457						
C=211; O=5; E=0.69; R=7.27; rawP=0.0006; adjP=0.0385						
Index	UserID	Value	Gene Symbol	Gene Name	EntrezGene	Ensembl
5 ☐	P62937	NA	PPIA	peptidylprolyl isomerase A (cyclophilin A)	5478	ENSG00000196262

Database:biological process Name:generation of precursor metabolites and energy ID:GO:0006091						
C=447; O=7; E=1.46; R=4.81; rawP=0.0006; adjP=0.0385						
Index	UserID	Value	Gene Symbol	Gene Name	EntrezGene	Ensembl
1 ☐	P48735	NA	IDH2	isocitrate dehydrogenase 2 (NADP+), mitochondrial	3418	ENSG00000182054
2 ☐	P62834	NA	RAP1A	RAP1A, member of RAS oncogene family	5906	ENSG00000116473
3 ☐	P10599	NA	TXN	thioredoxin	7295	ENSG00000136810
4 ☐	P09104	NA	ENO2	enolase 2 (gamma, neuronal)	2026	ENSG00000111674
5 ☐	P09972	NA	ALDOC	aldolase C, fructose-bisphosphate	230	ENSG00000109107
6 ☐	P00338	NA	LDHA	lactate dehydrogenase A	3939	ENSG00000134333
7 ☐	O75390	NA	CS	citrate synthase	1431	ENSG00000062485

Database:biological process Name:glucose metabolic process ID:GO:0006006						
C=213; O=5; E=0.69; R=7.20; rawP=0.0006; adjP=0.0385						
Index	UserID	Value	Gene Symbol	Gene Name	EntrezGene	Ensembl
1 ☐	Q01469	NA	FABP5	fatty acid binding protein 5 (psoriasis-associated)	2171	ENSG00000164687
2 ☐	P09104	NA	ENO2	enolase 2 (gamma, neuronal)	2026	ENSG00000111674
3 ☐	P00505	NA	GOT2	glutamic-oxaloacetic transaminase 2, mitochondrial (aspartate aminotransferase 2)	2806	ENSG00000125166
4 ☐	P09972	NA	ALDOC	aldolase C, fructose-bisphosphate	230	ENSG00000109107

Database:biological process Name:glucose metabolic process ID:GO:0006006						
C=213; O=5; E=0.69; R=7.20; rawP=0.0006; adjP=0.0385						
Index	UserID	Value	Gene Symbol	Gene Name	EntrezGene	Ensembl
5 ☐	P00338	NA	LDHA	lactate dehydrogenase A	3939	ENSG00000134333

Database:biological process Name:tricarboxylic acid metabolic process ID:GO:0072350						
C=13; O=2; E=0.04; R=47.21; rawP=0.0008; adjP=0.0449						
Index	UserID	Value	Gene Symbol	Gene Name	EntrezGene	Ensembl
1 ☐	P48735	NA	IDH2	isocitrate dehydrogenase 2 (NADP+), mitochondrial	3418	ENSG00000182054
2 ☐	O75390	NA	CS	citrate synthase	1431	ENSG00000062485

Database:biological process Name:hexose biosynthetic process ID:GO:0019319						
C=64; O=3; E=0.21; R=14.38; rawP=0.0012; adjP=0.0449						
Index	UserID	Value	Gene Symbol	Gene Name	EntrezGene	Ensembl
1 ☐	P09104	NA	ENO2	enolase 2 (gamma, neuronal)	2026	ENSG00000111674
2 ☐	P00505	NA	GOT2	glutamic-oxaloacetic transaminase 2, mitochondrial (aspartate aminotransferase 2)	2806	ENSG00000125166
3 ☐	P09972	NA	ALDOC	aldolase C, fructose-bisphosphate	230	ENSG00000109107

Database:biological process Name:carbohydrate metabolic process ID:GO:0005975						
C=786; O=9; E=2.56; R=3.51; rawP=0.0009; adjP=0.0449						
Index	UserID	Value	Gene Symbol	Gene Name	EntrezGene	Ensembl
1 ☐	P09104	NA	ENO2	enolase 2 (gamma, neuronal)	2026	ENSG00000111674
2 ☐	P00505	NA	GOT2	glutamic-oxaloacetic transaminase 2, mitochondrial (aspartate aminotransferase 2)	2806	ENSG00000125166
3 ☐	P09972	NA	ALDOC		230	ENSG00000109107

Database:biological process Name:carbohydrate metabolic process ID:GO:0005975						
C=786; O=9; E=2.56; R=3.51; rawP=0.0009; adjP=0.0449						
Index	UserID	Value	Gene Symbol	Gene Name	EntrezGene	Ensembl
				aldolase C, fructose-bisphosphate		
4 ☐	P00338	NA	LDHA	lactate dehydrogenase A	3939	ENSG00000134333
5 ☐	O75390	NA	CS	citrate synthase	1431	ENSG00000062485
6 ☐	P30101	NA	PDIA3	protein disulfide isomerase family A, member 3	2923	ENSG00000167004
7 ☐	Q01469	NA	FABP5	fatty acid binding protein 5 (psoriasis-associated)	2171	ENSG00000164687
8 ☐	P48735	NA	IDH2	isocitrate dehydrogenase 2 (NADP+), mitochondrial	3418	ENSG00000182054
9 ☐	Q04760	NA	GLO1	glyoxalase I	2739	ENSG00000124767

Database:biological process Name:gluconeogenesis ID:GO:0006094						
C=60; O=3; E=0.20; R=15.34; rawP=0.0010; adjP=0.0449						
Index	UserID	Value	Gene Symbol	Gene Name	EntrezGene	Ensembl
1 ☐	P09104	NA	ENO2	enolase 2 (gamma, neuronal)	2026	ENSG00000111674
2 ☐	P00505	NA	GOT2	glutamic-oxaloacetic transaminase 2, mitochondrial (aspartate aminotransferase 2)	2806	ENSG00000125166
3 ☐	P09972	NA	ALDOC	aldolase C, fructose-bisphosphate	230	ENSG00000109107

Database:biological process Name:hexose metabolic process ID:GO:0019318						
C=249; O=5; E=0.81; R=6.16; rawP=0.0013; adjP=0.0449						
Index	UserID	Value	Gene Symbol	Gene Name	EntrezGene	Ensembl
1 ☐	Q01469	NA	FABP5	fatty acid binding protein 5 (psoriasis-associated)	2171	ENSG00000164687
2 ☐	P09104	NA	ENO2		2026	ENSG00000111674

Database:biological process Name:hexose metabolic process ID:GO:0019318						
C=249; O=5; E=0.81; R=6.16; rawP=0.0013; adjP=0.0449						
Index	UserID	Value	Gene Symbol	Gene Name	EntrezGene	Ensembl
				enolase 2 (gamma, neuronal)		
3 ☐	P00505	NA	GOT2	glutamic-oxaloacetic transaminase 2, mitochondrial (aspartate aminotransferase 2)	2806	ENSG00000125166
4 ☐	P09972	NA	ALDOC	aldolase C, fructose-bisphosphate	230	ENSG00000109107
5 ☐	P00338	NA	LDHA	lactate dehydrogenase A	3939	ENSG00000134333

Database:biological process Name:glycolysis ID:GO:0006096						
C=63; O=3; E=0.21; R=14.61; rawP=0.0011; adjP=0.0449						
Index	UserID	Value	Gene Symbol	Gene Name	EntrezGene	Ensembl
1 ☐	P09104	NA	ENO2	enolase 2 (gamma, neuronal)	2026	ENSG00000111674
2 ☐	P09972	NA	ALDOC	aldolase C, fructose-bisphosphate	230	ENSG00000109107
3 ☐	P00338	NA	LDHA	lactate dehydrogenase A	3939	ENSG00000134333

Database:biological process Name:prostanoid biosynthetic process ID:GO:0046457						
C=21; O=2; E=0.07; R=29.22; rawP=0.0021; adjP=0.0469						
Index	UserID	Value	Gene Symbol	Gene Name	EntrezGene	Ensembl
1 ☐	P14174	NA	MIF	macrophage migration inhibitory factor (glycosylation-inhibiting factor)	4282	ENSG00000240972
2 ☐	Q15185	NA	PTGES3	prostaglandin E synthase 3 (cytosolic)	10728	ENSG00000110958

Database:biological process Name:glucose catabolic process ID:GO:0006007						
C=77; O=3; E=0.25; R=11.96; rawP=0.0020; adjP=0.0469						
Index	UserID	Value	Gene Symbol	Gene Name	EntrezGene	Ensembl
1 ☐	P09104	NA	ENO2		2026	ENSG00000111674

Database:biological process Name:glucose catabolic process ID:GO:0006007						
C=77; O=3; E=0.25; R=11.96; rawP=0.0020; adjP=0.0469						
Index	UserID	Value	Gene Symbol	Gene Name	EntrezGene	Ensembl
				enolase 2 (gamma, neuronal)		
2 ☐	P09972	NA	ALDOC	aldolase C, fructose-bisphosphate	230	ENSG00000109107
3 ☐	P00338	NA	LDHA	lactate dehydrogenase A	3939	ENSG00000134333

Database:biological process Name:monosaccharide metabolic process ID:GO:0005996						
C=279; O=5; E=0.91; R=5.50; rawP=0.0021; adjP=0.0469						
Index	UserID	Value	Gene Symbol	Gene Name	EntrezGene	Ensembl
1 ☐	Q01469	NA	FABP5	fatty acid binding protein 5 (psoriasis-associated)	2171	ENSG00000164687
2 ☐	P09104	NA	ENO2	enolase 2 (gamma, neuronal)	2026	ENSG00000111674
3 ☐	P00505	NA	GOT2	glutamic-oxaloacetic transaminase 2, mitochondrial (aspartate aminotransferase 2)	2806	ENSG00000125166
4 ☐	P09972	NA	ALDOC	aldolase C, fructose-bisphosphate	230	ENSG00000109107
5 ☐	P00338	NA	LDHA	lactate dehydrogenase A	3939	ENSG00000134333

Database:biological process Name:2-oxoglutarate metabolic process ID:GO:0006103						
C=19; O=2; E=0.06; R=32.30; rawP=0.0017; adjP=0.0469						
Index	UserID	Value	Gene Symbol	Gene Name	EntrezGene	Ensembl
1 ☐	P48735	NA	IDH2	isocitrate dehydrogenase 2 (NADP+), mitochondrial	3418	ENSG00000182054
2 ☐	P00505	NA	GOT2	glutamic-oxaloacetic transaminase 2, mitochondrial (aspartate aminotransferase 2)	2806	ENSG00000125166

Database:biological process Name:small molecule metabolic process ID:GO:0044281						
C=2500; O=17; E=8.15; R=2.09; rawP=0.0015; adjP=0.0469						
Index	UserID	Value	Gene Symbol	Gene Name	EntrezGene	Ensembl
1 ☐	P14174	NA	MIF	macrophage migration inhibitory factor (glycosylation-inhibiting factor)	4282	ENSG00000240972
2 ☐	P10599	NA	TXN	thioredoxin	7295	ENSG00000136810
3 ☐	P09104	NA	ENO2	enolase 2 (gamma, neuronal)	2026	ENSG00000111674
4 ☐	P00338	NA	LDHA	lactate dehydrogenase A	3939	ENSG00000134333
5 ☐	P62633	NA	CNBP	CCHC-type zinc finger, nucleic acid binding protein	7555	ENSG00000169714
6 ☐	P30101	NA	PDIA3	protein disulfide isomerase family A, member 3	2923	ENSG00000167004
7 ☐	Q01469	NA	FABP5	fatty acid binding protein 5 (psoriasis-associated)	2171	ENSG00000164687
8 ☐	Q04760	NA	GLO1	glyoxalase I	2739	ENSG00000124767
9 ☐	P48735	NA	IDH2	isocitrate dehydrogenase 2 (NADP+), mitochondrial	3418	ENSG00000182054
10 ☐	P07741	NA	APRT	adenine phosphoribosyltransferase	353	ENSG00000198931
11 ☐	Q8NBS9	NA	TXNDC5	thioredoxin domain containing 5 (endoplasmic reticulum)	81567	ENSG00000239264
12 ☐	P13667	NA	PDIA4	protein disulfide isomerase family A, member 4	9601	ENSG00000155660
13 ☐	P62834	NA	RAP1A	RAP1A, member of RAS oncogene family	5906	ENSG00000116473
14 ☐	Q9NR31	NA	SAR1A	SAR1 homolog A (S. cerevisiae)	56681	ENSG00000079332
15 ☐	P00505	NA	GOT2	glutamic-oxaloacetic transaminase 2, mitochondrial (aspartate aminotransferase 2)	2806	ENSG00000125166
16 ☐	P09972	NA	ALDOC	aldolase C, fructose-bisphosphate	230	ENSG00000109107
17 ☐	O75390	NA	CS	citrate synthase	1431	ENSG00000062485

Database:biological process Name:transport ID:GO:0006810						
C=3323; O=20; E=10.83; R=1.85; rawP=0.0023; adjP=0.0469						
Index	UserID	Value	Gene Symbol	Gene Name	EntrezGene	Ensembl

Database:biological process Name:transport ID:GO:0006810						
C=3323; O=20; E=10.83; R=1.85; rawP=0.0023; adjP=0.0469						
Index	UserID	Value	Gene Symbol	Gene Name	EntrezGene	Ensembl
1 ☐	Q15121	NA	PEA15	phosphoprotein enriched in astrocytes 15	8682	ENSG00000162734
2 ☐	P14174	NA	MIF	macrophage migration inhibitory factor (glycosylation-inhibiting factor)	4282	ENSG00000240972
3 ☐	P10599	NA	TXN	thioredoxin	7295	ENSG00000136810
4 ☐	P61326	NA	MAGOH	mago-nashi homolog, proliferation-associated (Drosophila)	4116	ENSG00000162385
5 ☐	P30101	NA	PDIA3	protein disulfide isomerase family A, member 3	2923	ENSG00000167004
6 ☐	Q01469	NA	FABP5	fatty acid binding protein 5 (psoriasis-associated)	2171	ENSG00000164687
7 ☐	P07741	NA	APRT	adenine phosphoribosyltransferase	353	ENSG00000198931
8 ☐	P30040	NA	ERP29	endoplasmic reticulum protein 29	10961	ENSG00000089248
9 ☐	P62937	NA	PPIA	peptidylprolyl isomerase A (cyclophilin A)	5478	ENSG00000196262
10 ☐	P13667	NA	PDIA4	protein disulfide isomerase family A, member 4	9601	ENSG00000155660
11 ☐	Q8NBS9	NA	TXNDC5	thioredoxin domain containing 5 (endoplasmic reticulum)	81567	ENSG00000239264
12 ☐	P22626	NA	HNRNPA2B1	heterogeneous nuclear ribonucleoprotein A2/B1	3181	ENSG00000122566
13 ☐	P62834	NA	RAP1A	RAP1A, member of RAS oncogene family	5906	ENSG00000116473
14 ☐	Q9NR31	NA	SAR1A	SAR1 homolog A (S. cerevisiae)	56681	ENSG00000079332
15 ☐	P00505	NA	GOT2	glutamic-oxaloacetic transaminase 2, mitochondrial (aspartate aminotransferase 2)	2806	ENSG00000125166
16 ☐	P50395	NA	GDI2	GDP dissociation inhibitor 2	2665	ENSG00000057608
17 ☐	P07737	NA	PFN1	profilin 1	5216	ENSG00000108518
18 ☐	O60493	NA	SNX3	sorting nexin 3	8724	ENSG00000112335
19 ☐	O00299	NA	CLIC1	chloride intracellular channel 1	1192	ENSG00000213719
20 ☐	P13693	NA	TPT1	tumor protein, translationally-controlled 1	7178	ENSG00000133112

Database:biological process Name:transport ID:GO:0006810						
C=3323; O=20; E=10.83; R=1.85; rawP=0.0023; adjP=0.0469						
Index	UserID	Value	Gene Symbol	Gene Name	EntrezGene	Ensembl

Database:biological process Name:monosaccharide biosynthetic process ID:GO:0046364						
C=73; O=3; E=0.24; R=12.61; rawP=0.0017; adjP=0.0469						
Index	UserID	Value	Gene Symbol	Gene Name	EntrezGene	Ensembl
1 ☐	P09104	NA	ENO2	enolase 2 (gamma, neuronal)	2026	ENSG00000111674
2 ☐	P00505	NA	GOT2	glutamic-oxaloacetic transaminase 2, mitochondrial (aspartate aminotransferase 2)	2806	ENSG00000125166
3 ☐	P09972	NA	ALDOC	aldolase C, fructose-bisphosphate	230	ENSG00000109107

Database:biological process Name:prostaglandin biosynthetic process ID:GO:0001516						
C=21; O=2; E=0.07; R=29.22; rawP=0.0021; adjP=0.0469						
Index	UserID	Value	Gene Symbol	Gene Name	EntrezGene	Ensembl
1 ☐	P14174	NA	MIF	macrophage migration inhibitory factor (glycosylation-inhibiting factor)	4282	ENSG00000240972
2 ☐	Q15185	NA	PTGES3	prostaglandin E synthase 3 (cytosolic)	10728	ENSG00000110958

Database:biological process Name:dicarboxylic acid metabolic process ID:GO:0043648						
C=81; O=3; E=0.26; R=11.36; rawP=0.0023; adjP=0.0469						
Index	UserID	Value	Gene Symbol	Gene Name	EntrezGene	Ensembl
1 ☐	P48735	NA	IDH2	isocitrate dehydrogenase 2 (NADP+), mitochondrial	3418	ENSG00000182054
2 ☐	P00505	NA	GOT2	glutamic-oxaloacetic transaminase 2, mitochondrial (aspartate aminotransferase 2)	2806	ENSG00000125166

Database:biological process			Name:dicarboxylic acid metabolic process		ID:GO:0043648	
C=81; O=3; E=0.26; R=11.36; rawP=0.0023; adjP=0.0469						
Index	UserID	Value	Gene Symbol	Gene Name	EntrezGene	Ensembl
3 ☐	O75390	NA	CS	citrate synthase	1431	ENSG00000062485

Database:biological process			Name:organic substance transport		ID:GO:0071702	
C=1929; O=14; E=6.29; R=2.23; rawP=0.0026; adjP=0.0508						
Index	UserID	Value	Gene Symbol	Gene Name	EntrezGene	Ensembl
1 ☐	Q15121	NA	PEA15	phosphoprotein enriched in astrocytes 15	8682	ENSG00000162734
2 ☐	P22626	NA	HNRNPA2B1	heterogeneous nuclear ribonucleoprotein A2/B1	3181	ENSG00000122566
3 ☐	P14174	NA	MIF	macrophage migration inhibitory factor (glycosylation-inhibiting factor)	4282	ENSG00000240972
4 ☐	P62834	NA	RAP1A	RAP1A, member of RAS oncogene family	5906	ENSG00000116473
5 ☐	P10599	NA	TXN	thioredoxin	7295	ENSG00000136810
6 ☐	P50395	NA	GDI2	GDP dissociation inhibitor 2	2665	ENSG00000057608
7 ☐	P00505	NA	GOT2	glutamic-oxaloacetic transaminase 2, mitochondrial (aspartate aminotransferase 2)	2806	ENSG00000125166
8 ☐	Q9NR31	NA	SAR1A	SAR1 homolog A (S. cerevisiae)	56681	ENSG00000079332
9 ☐	O60493	NA	SNX3	sorting nexin 3	8724	ENSG00000112335
10 ☐	P61326	NA	MAGOH	mago-nashi homolog, proliferation-associated (Drosophila)	4116	ENSG00000162385
11 ☐	P30101	NA	PDIA3	protein disulfide isomerase family A, member 3	2923	ENSG00000167004
12 ☐	Q01469	NA	FABP5	fatty acid binding protein 5 (psoriasis-associated)	2171	ENSG00000164687
13 ☐	P30040	NA	ERP29		10961	ENSG00000089248

Database:biological process			Name:organic substance transport		ID:GO:0071702	
C=1929; O=14; E=6.29; R=2.23; rawP=0.0026; adjP=0.0508						
Index	UserID	Value	Gene Symbol	Gene Name	EntrezGene	Ensembl
				endoplasmic reticulum protein 29		
14 ☐	P13667	NA	PDIA4	protein disulfide isomerase family A, member 4	9601	ENSG00000155660

Database:biological process			Name:establishment of localization		ID:GO:0051234	
C=3377; O=20; E=11.01; R=1.82; rawP=0.0028; adjP=0.0518						
Index	UserID	Value	Gene Symbol	Gene Name	EntrezGene	Ensembl
1 ☐	Q15121	NA	PEA15	phosphoprotein enriched in astrocytes 15	8682	ENSG00000162734
2 ☐	P14174	NA	MIF	macrophage migration inhibitory factor (glycosylation-inhibiting factor)	4282	ENSG00000240972
3 ☐	P10599	NA	TXN	thioredoxin	7295	ENSG00000136810
4 ☐	P61326	NA	MAGOH	mago-nashi homolog, proliferation-associated (Drosophila)	4116	ENSG00000162385
5 ☐	P30101	NA	PDIA3	protein disulfide isomerase family A, member 3	2923	ENSG00000167004
6 ☐	Q01469	NA	FABP5	fatty acid binding protein 5 (psoriasis-associated)	2171	ENSG00000164687
7 ☐	P07741	NA	APRT	adenine phosphoribosyltransferase	353	ENSG00000198931
8 ☐	P30040	NA	ERP29	endoplasmic reticulum protein 29	10961	ENSG00000089248
9 ☐	P62937	NA	PPIA	peptidylprolyl isomerase A (cyclophilin A)	5478	ENSG00000196262
10 ☐	P13667	NA	PDIA4	protein disulfide isomerase family A, member 4	9601	ENSG00000155660
11 ☐	Q8NBS9	NA	TXNDC5	thioredoxin domain containing 5 (endoplasmic reticulum)	81567	ENSG00000239264
12 ☐	P22626	NA	HNRNPA2B1	heterogeneous nuclear ribonucleoprotein A2/B1	3181	ENSG00000122566
13 ☐	P62834	NA	RAP1A	RAP1A, member of RAS oncogene family	5906	ENSG00000116473
14 ☐	Q9NR31	NA	SAR1A	SAR1 homolog A (S. cerevisiae)	56681	ENSG00000079332
15 ☐	P00505	NA	GOT2	glutamic-oxaloacetic transaminase 2,	2806	ENSG00000125166

Database:biological process Name:establishment of localization ID:GO:0051234						
C=3377; O=20; E=11.01; R=1.82; rawP=0.0028; adjP=0.0518						
Index	UserID	Value	Gene Symbol	Gene Name	EntrezGene	Ensembl
				mitochondrial (aspartate aminotransferase 2)		
16 ☐	P50395	NA	GDI2	GDP dissociation inhibitor 2	2665	ENSG00000057608
17 ☐	P07737	NA	PFN1	profilin 1	5216	ENSG00000108518
18 ☐	O60493	NA	SNX3	sorting nexin 3	8724	ENSG00000112335
19 ☐	O00299	NA	CLIC1	chloride intracellular channel 1	1192	ENSG00000213719
20 ☐	P13693	NA	TPT1	tumor protein, translationally-controlled 1	7178	ENSG00000133112

Database:biological process Name:RNA stabilization ID:GO:0043489						
C=25; O=2; E=0.08; R=24.55; rawP=0.0030; adjP=0.0518						
Index	UserID	Value	Gene Symbol	Gene Name	EntrezGene	Ensembl
1 ☐	Q14103	NA	HNRNPD	heterogeneous nuclear ribonucleoprotein D (AU-rich element RNA binding protein 1, 37kDa)	3184	ENSG00000138668
2 ☐	Q15717	NA	ELAVL1	ELAV (embryonic lethal, abnormal vision, Drosophila)-like 1 (Hu antigen R)	1994	ENSG00000066044

Database:biological process Name:mRNA stabilization ID:GO:0048255						
C=25; O=2; E=0.08; R=24.55; rawP=0.0030; adjP=0.0518						
Index	UserID	Value	Gene Symbol	Gene Name	EntrezGene	Ensembl
1 ☐	Q14103	NA	HNRNPD	heterogeneous nuclear ribonucleoprotein D (AU-rich element RNA binding protein 1, 37kDa)	3184	ENSG00000138668
2 ☐	Q15717	NA	ELAVL1	ELAV (embryonic lethal, abnormal vision, Drosophila)-like 1 (Hu antigen R)	1994	ENSG00000066044

Database:biological process Name:tricarboxylic acid cycle ID:GO:0006099						
C=26; O=2; E=0.08; R=23.60; rawP=0.0032; adjP=0.0532						
Index	UserID	Value	Gene Symbol	Gene Name	EntrezGene	Ensembl
1 ☐	P48735	NA	IDH2	isocitrate dehydrogenase 2 (NADP+), mitochondrial	3418	ENSG00000182054
2 ☐	O75390	NA	CS	citrate synthase	1431	ENSG00000062485

Database:biological process Name:hexose catabolic process ID:GO:0019320						
C=93; O=3; E=0.30; R=9.90; rawP=0.0034; adjP=0.0542						
Index	UserID	Value	Gene Symbol	Gene Name	EntrezGene	Ensembl
1 ☐	P09104	NA	ENO2	enolase 2 (gamma, neuronal)	2026	ENSG00000111674
2 ☐	P09972	NA	ALDOC	aldolase C, fructose-bisphosphate	230	ENSG00000109107
3 ☐	P00338	NA	LDHA	lactate dehydrogenase A	3939	ENSG00000134333

Database:biological process Name:acetyl-CoA catabolic process ID:GO:0046356						
C=27; O=2; E=0.09; R=22.73; rawP=0.0035; adjP=0.0542						
Index	UserID	Value	Gene Symbol	Gene Name	EntrezGene	Ensembl
1 ☐	P48735	NA	IDH2	isocitrate dehydrogenase 2 (NADP+), mitochondrial	3418	ENSG00000182054
2 ☐	O75390	NA	CS	citrate synthase	1431	ENSG00000062485

Database:biological process Name:prostanoid metabolic process ID:GO:0006692						
C=29; O=2; E=0.09; R=21.16; rawP=0.0040; adjP=0.0561						
Index	UserID	Value	Gene Symbol	Gene Name	EntrezGene	Ensembl
1 ☐	P14174	NA	MIF	macrophage migration inhibitory factor (glycosylation-inhibiting factor)	4282	ENSG00000240972
2 ☐	Q15185	NA	PTGES3	prostaglandin E synthase 3 (cytosolic)	10728	ENSG00000110958

Database:biological process Name:monosaccharide catabolic process ID:GO:0046365						
C=98; O=3; E=0.32; R=9.39; rawP=0.0040; adjP=0.0561						
Index	UserID	Value	Gene Symbol	Gene Name	EntrezGene	Ensembl
1 ☐	P09104	NA	ENO2	enolase 2 (gamma, neuronal)	2026	ENSG00000111674
2 ☐	P09972	NA	ALDOC	aldolase C, fructose-bisphosphate	230	ENSG00000109107
3 ☐	P00338	NA	LDHA	lactate dehydrogenase A	3939	ENSG00000134333

Database:biological process Name:prostaglandin metabolic process ID:GO:0006693						
C=29; O=2; E=0.09; R=21.16; rawP=0.0040; adjP=0.0561						
Index	UserID	Value	Gene Symbol	Gene Name	EntrezGene	Ensembl
1 ☐	P14174	NA	MIF	macrophage migration inhibitory factor (glycosylation-inhibiting factor)	4282	ENSG00000240972
2 ☐	Q15185	NA	PTGES3	prostaglandin E synthase 3 (cytosolic)	10728	ENSG00000110958

Database:biological process Name:coenzyme catabolic process ID:GO:0009109						
C=33; O=2; E=0.11; R=18.60; rawP=0.0051; adjP=0.0694						
Index	UserID	Value	Gene Symbol	Gene Name	EntrezGene	Ensembl
1 ☐	P48735	NA	IDH2	isocitrate dehydrogenase 2 (NADP+), mitochondrial	3418	ENSG00000182054
2 ☐	O75390	NA	CS	citrate synthase	1431	ENSG00000062485

Database:biological process Name:regulation of mRNA stability ID:GO:0043488						
C=35; O=2; E=0.11; R=17.53; rawP=0.0058; adjP=0.0766						
Index	UserID	Value	Gene Symbol	Gene Name	EntrezGene	Ensembl
1 ☐	Q14103	NA	HNRNPD	heterogeneous nuclear ribonucleoprotein D (AU-rich element RNA binding protein 1, 37kDa)	3184	ENSG00000138668
2 ☐	Q15717	NA	ELAVL1	ELAV (embryonic lethal, abnormal vision,	1994	ENSG00000066044

Database:biological process Name:regulation of mRNA stability ID:GO:0043488						
C=35; O=2; E=0.11; R=17.53; rawP=0.0058; adjP=0.0766						
Index	UserID	Value	Gene Symbol	Gene Name	EntrezGene	Ensembl
				Drosophila)-like 1 (Hu antigen R)		

Database:biological process Name:cofactor catabolic process ID:GO:0051187						
C=39; O=2; E=0.13; R=15.74; rawP=0.0071; adjP=0.0886						
Index	UserID	Value	Gene Symbol	Gene Name	EntrezGene	Ensembl
1 ☐	P48735	NA	IDH2	isocitrate dehydrogenase 2 (NADP+), mitochondrial	3418	ENSG00000182054
2 ☐	O75390	NA	CS	citrate synthase	1431	ENSG00000062485

Database:biological process Name:regulation of RNA stability ID:GO:0043487						
C=39; O=2; E=0.13; R=15.74; rawP=0.0071; adjP=0.0886						
Index	UserID	Value	Gene Symbol	Gene Name	EntrezGene	Ensembl
1 ☐	Q14103	NA	HNRNPD	heterogeneous nuclear ribonucleoprotein D (AU-rich element RNA binding protein 1, 37kDa)	3184	ENSG00000138668
2 ☐	Q15717	NA	ELAVL1	ELAV (embryonic lethal, abnormal vision, Drosophila)-like 1 (Hu antigen R)	1994	ENSG00000066044

Database:biological process Name:cellular aldehyde metabolic process ID:GO:0006081						
C=40; O=2; E=0.13; R=15.34; rawP=0.0075; adjP=0.0910						
Index	UserID	Value	Gene Symbol	Gene Name	EntrezGene	Ensembl
1 ☐	Q04760	NA	GLO1	glyoxalase I	2739	ENSG00000124767
2 ☐	P48735	NA	IDH2	isocitrate dehydrogenase 2 (NADP+), mitochondrial	3418	ENSG00000182054

Database:molecular function Name:intramolecular oxidoreductase activity, interconverting keto- and enol-groups ID:GO:0016862						
C=11; O=4; E=0.04; R=113.46; rawP=3.01e-08; adjP=2.53e-06						
Index	UserID	Value	Gene Symbol	Gene Name	EntrezGene	Ensembl
1 ☐	P30101	NA	PDIA3		2923	ENSG00000167004

**Database:molecular function Name:intramolecular oxidoreductase activity, interconverting keto- and enol-groups ID:GO:0016862**						
C=11; O=4; E=0.04; R=113.46; rawP=3.01e-08; adjP=2.53e-06						
Index	UserID	Value	Gene Symbol	Gene Name	EntrezGene	Ensembl
				protein disulfide isomerase family A, member 3		
2 ☐	P14174	NA	MIF	macrophage migration inhibitory factor (glycosylation-inhibiting factor)	4282	ENSG00000240972
3 ☐	P30040	NA	ERP29	endoplasmic reticulum protein 29	10961	ENSG00000089248
4 ☐	P13667	NA	PDIA4	protein disulfide isomerase family A, member 4	9601	ENSG00000155660

**Database:molecular function activity Name:intramolecular oxidoreductase ID:GO:0016860**						
C=45; O=5; E=0.14; R=34.67; rawP=3.03e-07; adjP=8.85e-06						
Index	UserID	Value	Gene Symbol	Gene Name	EntrezGene	Ensembl
1 ☐	P30101	NA	PDIA3	protein disulfide isomerase family A, member 3	2923	ENSG00000167004
2 ☐	P14174	NA	MIF	macrophage migration inhibitory factor (glycosylation-inhibiting factor)	4282	ENSG00000240972
3 ☐	Q15185	NA	PTGES3	prostaglandin E synthase 3 (cytosolic)	10728	ENSG00000110958
4 ☐	P30040	NA	ERP29	endoplasmic reticulum protein 29	10961	ENSG00000089248
5 ☐	P13667	NA	PDIA4	protein disulfide isomerase family A, member 4	9601	ENSG00000155660

**Database:molecular function Name:isomerase activity ID:GO:0016853**						
C=142; O=7; E=0.46; R=15.38; rawP=3.16e-07; adjP=8.85e-06						
Index	UserID	Value	Gene Symbol	Gene Name	EntrezGene	Ensembl
1 ☐	P30101	NA	PDIA3	protein disulfide isomerase family A, member 3	2923	ENSG00000167004
2 ☐	P14174	NA	MIF	macrophage migration inhibitory factor	4282	ENSG00000240972

Database:molecular function Name:isomerase activity ID:GO:0016853						
C=142; O=7; E=0.46; R=15.38; rawP=3.16e-07; adjP=8.85e-06						
Index	UserID	Value	Gene Symbol	Gene Name	EntrezGene	Ensembl
				(glycosylation-inhibiting factor)		
3 ☐	Q15185	NA	PTGES3	prostaglandin E synthase 3 (cytosolic)	10728	ENSG00000110958
4 ☐	P30040	NA	ERP29	endoplasmic reticulum protein 29	10961	ENSG00000089248
5 ☐	P62937	NA	PPIA	peptidylprolyl isomerase A (cyclophilin A)	5478	ENSG00000196262
6 ☐	Q8NBS9	NA	TXNDC5	thioredoxin domain containing 5 (endoplasmic reticulum)	81567	ENSG00000239264
7 ☐	P13667	NA	PDIA4	protein disulfide isomerase family A, member 4	9601	ENSG00000155660

Database:molecular function Name:protein disulfide oxidoreductase activity ID:GO:0015035						
C=27; O=4; E=0.09; R=46.23; rawP=1.54e-06; adjP=3.23e-05						
Index	UserID	Value	Gene Symbol	Gene Name	EntrezGene	Ensembl
1 ☐	P30101	NA	PDIA3	protein disulfide isomerase family A, member 3	2923	ENSG00000167004
2 ☐	P10599	NA	TXN	thioredoxin	7295	ENSG00000136810
3 ☐	Q8NBS9	NA	TXNDC5	thioredoxin domain containing 5 (endoplasmic reticulum)	81567	ENSG00000239264
4 ☐	P13667	NA	PDIA4	protein disulfide isomerase family A, member 4	9601	ENSG00000155660

Database:molecular function Name:disulfide oxidoreductase activity ID:GO:0015036						
C=32; O=4; E=0.10; R=39.00; rawP=3.13e-06; adjP=4.38e-05						
Index	UserID	Value	Gene Symbol	Gene Name	EntrezGene	Ensembl
1 ☐	P30101	NA	PDIA3	protein disulfide isomerase family A, member 3	2923	ENSG00000167004
2 ☐	P10599	NA	TXN	thioredoxin	7295	ENSG00000136810

Database:molecular function Name:disulfide oxidoreductase activity ID:GO:0015036						
C=32; O=4; E=0.10; R=39.00; rawP=3.13e-06; adjP=4.38e-05						
Index	UserID	Value	Gene Symbol	Gene Name	EntrezGene	Ensembl
3 ☐	Q8NBS9	NA	TXNDC5	thioredoxin domain containing 5 (endoplasmic reticulum)	81567	ENSG00000239264
4 ☐	P13667	NA	PDIA4	protein disulfide isomerase family A, member 4	9601	ENSG00000155660

Database:molecular function Name:protein disulfide isomerase activity ID:GO:0003756						
C=10; O=3; E=0.03; R=93.61; rawP=3.65e-06; adjP=4.38e-05						
Index	UserID	Value	Gene Symbol	Gene Name	EntrezGene	Ensembl
1 ☐	P30101	NA	PDIA3	protein disulfide isomerase family A, member 3	2923	ENSG00000167004
2 ☐	P30040	NA	ERP29	endoplasmic reticulum protein 29	10961	ENSG00000089248
3 ☐	P13667	NA	PDIA4	protein disulfide isomerase family A, member 4	9601	ENSG00000155660

Database:molecular function Name:intramolecular oxidoreductase activity, transposing S-S bonds ID:GO:0016864						
C=10; O=3; E=0.03; R=93.61; rawP=3.65e-06; adjP=4.38e-05						
Index	UserID	Value	Gene Symbol	Gene Name	EntrezGene	Ensembl
1 ☐	P30101	NA	PDIA3	protein disulfide isomerase family A, member 3	2923	ENSG00000167004
2 ☐	P30040	NA	ERP29	endoplasmic reticulum protein 29	10961	ENSG00000089248
3 ☐	P13667	NA	PDIA4	protein disulfide isomerase family A, member 4	9601	ENSG00000155660

Database:molecular function Name:telomeric DNA binding ID:GO:0042162						
C=16; O=3; E=0.05; R=58.50; rawP=1.68e-05; adjP=0.0002						
Index	UserID	Value	Gene Symbol	Gene Name	EntrezGene	Ensembl
1 ☐	Q14103	NA	HNRNPD	heterogeneous nuclear ribonucleoprotein D (AU-rich element RNA	3184	ENSG00000138668

Database:molecular function Name:telomeric DNA binding ID:GO:0042162						
C=16; O=3; E=0.05; R=58.50; rawP=1.68e-05; adjP=0.0002						
Index	UserID	Value	Gene Symbol	Gene Name	EntrezGene	Ensembl
				binding protein 1, 37kDa)		
2 ☐	P22626	NA	HNRNPA2B1	heterogeneous nuclear ribonucleoprotein A2/B1	3181	ENSG00000122566
3 ☐	P19338	NA	NCL	nucleolin	4691	ENSG00000115053

Database:molecular function Name:oxidoreductase activity, acting on a sulfur group of donors ID:GO:0016667						
C=54; O=4; E=0.17; R=23.11; rawP=2.61e-05; adjP=0.0002						
Index	UserID	Value	Gene Symbol	Gene Name	EntrezGene	Ensembl
1 ☐	P30101	NA	PDIA3	protein disulfide isomerase family A, member 3	2923	ENSG00000167004
2 ☐	P10599	NA	TXN	thioredoxin	7295	ENSG00000136810
3 ☐	Q8NBS9	NA	TXNDC5	thioredoxin domain containing 5 (endoplasmic reticulum)	81567	ENSG00000239264
4 ☐	P13667	NA	PDIA4	protein disulfide isomerase family A, member 4	9601	ENSG00000155660

Database:molecular function Name:RNA binding ID:GO:0003723						
C=843; O=11; E=2.70; R=4.07; rawP=5.58e-05; adjP=0.0005						
Index	UserID	Value	Gene Symbol	Gene Name	EntrezGene	Ensembl
1 ☐	Q12905	NA	ILF2	interleukin enhancer binding factor 2, 45kDa	3608	ENSG00000143621
2 ☐	P22626	NA	HNRNPA2B1	heterogeneous nuclear ribonucleoprotein A2/B1	3181	ENSG00000122566
3 ☐	Q96SI9	NA	STRBP	spermatid perinuclear RNA binding protein	55342	ENSG00000165209
4 ☐	Q99729	NA	HNRNPAB	heterogeneous nuclear ribonucleoprotein A/B	3182	ENSG00000197451
5 ☐	Q15717	NA	ELAVL1	ELAV (embryonic lethal, abnormal vision,	1994	ENSG00000066044

Database:molecular function Name:RNA binding ID:GO:0003723						
C=843; O=11; E=2.70; R=4.07; rawP=5.58e-05; adjP=0.0005						
Index	UserID	Value	Gene Symbol	Gene Name	EntrezGene	Ensembl
				Drosophila)-like 1 (Hu antigen R)		
6 ☐	P61326	NA	MAGOH	mago-nashi homolog, proliferation-associated (Drosophila)	4116	ENSG00000162385
7 ☐	Q12906	NA	ILF3	interleukin enhancer binding factor 3, 90kDa	3609	ENSG00000129351
8 ☐	Q14103	NA	HNRNPD	heterogeneous nuclear ribonucleoprotein D (AU-rich element RNA binding protein 1, 37kDa)	3184	ENSG00000138668
9 ☐	P29558	NA	RBMS1	RNA binding motif, single stranded interacting protein 1	5937	ENSG00000153250
10 ☐	P19338	NA	NCL	nucleolin	4691	ENSG00000115053
11 ☐	O14979	NA	HNRPDL	heterogeneous nuclear ribonucleoprotein D-like	9987	ENSG00000152795

Database:molecular function Name:double-stranded RNA binding ID:GO:0003725						
C=44; O=3; E=0.14; R=21.27; rawP=0.0004; adjP=0.0031						
Index	UserID	Value	Gene Symbol	Gene Name	EntrezGene	Ensembl
1 ☐	Q12905	NA	ILF2	interleukin enhancer binding factor 2, 45kDa	3608	ENSG00000143621
2 ☐	Q96SI9	NA	STRBP	spermatid perinuclear RNA binding protein	55342	ENSG00000165209
3 ☐	Q12906	NA	ILF3	interleukin enhancer binding factor 3, 90kDa	3609	ENSG00000129351

Database:molecular function Name:electron carrier activity ID:GO:0009055						
C=154; O=4; E=0.49; R=8.10; rawP=0.0015; adjP=0.0103						
Index	UserID	Value	Gene Symbol	Gene Name	EntrezGene	Ensembl
1 ☐	P30101	NA	PDIA3		2923	ENSG00000167004

Database:molecular function			Name:electron carrier activity		ID:GO:0009055	
C=154; O=4; E=0.49; R=8.10; rawP=0.0015; adjP=0.0103						
Index	UserID	Value	Gene Symbol	Gene Name	EntrezGene	Ensembl
				protein disulfide isomerase family A, member 3		
2 ☐	P10599	NA	TXN	thioredoxin	7295	ENSG00000136810
3 ☐	Q8NBS9	NA	TXNDC5	thioredoxin domain containing 5 (endoplasmic reticulum)	81567	ENSG00000239264
4 ☐	P13667	NA	PDIA4	protein disulfide isomerase family A, member 4	9601	ENSG00000155660

Database:molecular function			Name:protein binding		ID:GO:0005515	
C=7301; O=34; E=23.40; R=1.45; rawP=0.0016; adjP=0.0103						
Index	UserID	Value	Gene Symbol	Gene Name	EntrezGene	Ensembl
1 ☐	P14174	NA	MIF	macrophage migration inhibitory factor (glycosylation-inhibiting factor)	4282	ENSG00000240972
2 ☐	P10599	NA	TXN	thioredoxin	7295	ENSG00000136810
3 ☐	P09104	NA	ENO2	enolase 2 (gamma, neuronal)	2026	ENSG00000111674
4 ☐	P61956	NA	SUMO2	SMT3 suppressor of mif two 3 homolog 2 (S. cerevisiae)	6613	ENSG00000188612
5 ☐	P30086	NA	PEBP1	phosphatidylethanolamine binding protein 1	5037	ENSG00000089220
6 ☐	P30101	NA	PDIA3	protein disulfide isomerase family A, member 3	2923	ENSG00000167004
7 ☐	P62937	NA	PPIA	peptidylprolyl isomerase A (cyclophilin A)	5478	ENSG00000196262
8 ☐	P13667	NA	PDIA4	protein disulfide isomerase family A, member 4	9601	ENSG00000155660
9 ☐	P22626	NA	HNRNPA2B1	heterogeneous nuclear ribonucleoprotein A2/B1	3181	ENSG00000122566
10 ☐	P62834	NA	RAP1A	RAP1A, member of RAS oncogene family	5906	ENSG00000116473
11 ☐	P60903	NA	S100A10	S100 calcium binding protein A10	6281	ENSG00000197747
12 ☐	O15511	NA	ARPC5	actin related protein 2/3 complex, subunit 5, 16kDa	10092	ENSG00000162704

Database:molecular function			Name:protein binding		ID:GO:0005515	
C=7301; O=34; E=23.40; R=1.45; rawP=0.0016; adjP=0.0103						
Index	UserID	Value	Gene Symbol	Gene Name	EntrezGene	Ensembl
13 ☐	P50395	NA	GDI2	GDP dissociation inhibitor 2	2665	ENSG00000057608
14 ☐	P09972	NA	ALDOC	aldolase C, fructose-bisphosphate	230	ENSG00000109107
15 ☐	P07737	NA	PFN1	profilin 1	5216	ENSG00000108518
16 ☐	O60493	NA	SNX3	sorting nexin 3	8724	ENSG00000112335
17 ☐	Q14103	NA	HNRNPD	heterogeneous nuclear ribonucleoprotein D (AU-rich element RNA binding protein 1, 37kDa)	3184	ENSG00000138668
18 ☐	O00299	NA	CLIC1	chloride intracellular channel 1	1192	ENSG00000213719
19 ☐	Q15185	NA	PTGES3	prostaglandin E synthase 3 (cytosolic)	10728	ENSG00000110958
20 ☐	Q15121	NA	PEA15	phosphoprotein enriched in astrocytes 15	8682	ENSG00000162734
21 ☐	Q12905	NA	ILF2	interleukin enhancer binding factor 2, 45kDa	3608	ENSG00000143621
22 ☐	Q12906	NA	ILF3	interleukin enhancer binding factor 3, 90kDa	3609	ENSG00000129351
23 ☐	P61326	NA	MAGOH	mago-nashi homolog, proliferation-associated (Drosophila)	4116	ENSG00000162385
24 ☐	P09382	NA	LGALS1	lectin, galactoside-binding, soluble, 1	3956	ENSG00000100097
25 ☐	P19338	NA	NCL	nucleolin	4691	ENSG00000115053
26 ☐	Q01469	NA	FABP5	fatty acid binding protein 5 (psoriasis-associated)	2171	ENSG00000164687
27 ☐	Q14019	NA	COTL1	coactosin-like 1 (Dictyostelium)	23406	ENSG00000103187
28 ☐	Q9Y281	NA	CFL2	cofilin 2 (muscle)	1073	ENSG00000165410
29 ☐	Q15843	NA	NEDD8	neural precursor cell expressed, developmentally down-regulated 8	4738	ENSG00000129559
30 ☐	Q99471	NA	PFDN5	prefoldin subunit 5	5204	ENSG00000123349
31 ☐	Q15717	NA	ELAVL1	ELAV (embryonic lethal, abnormal vision, Drosophila)-like 1 (Hu antigen R)	1994	ENSG00000066044
32 ☐	P29558	NA	RBMS1	RNA binding motif, single stranded interacting protein 1	5937	ENSG00000153250
33 ☐	P61088	NA	UBE2N	ubiquitin-conjugating enzyme E2N	7334	ENSG00000177889

Database:molecular function Name:protein binding ID:GO:0005515						
C=7301; O=34; E=23.40; R=1.45; rawP=0.0016; adjP=0.0103						
Index	UserID	Value	Gene Symbol	Gene Name	EntrezGene	Ensembl
34 ☐	P13693	NA	TPT1	tumor protein, translationally-controlled 1	7178	ENSG00000133112

Database:molecular function Name:mRNA binding ID:GO:0003729						
C=90; O=3; E=0.29; R=10.40; rawP=0.0030; adjP=0.0180						
Index	UserID	Value	Gene Symbol	Gene Name	EntrezGene	Ensembl
1 ☐	Q99729	NA	HNRNPAB	heterogeneous nuclear ribonucleoprotein A/B	3182	ENSG00000197451
2 ☐	O14979	NA	HNRPDL	heterogeneous nuclear ribonucleoprotein D-like	9987	ENSG00000152795
3 ☐	Q15717	NA	ELAVL1	ELAV (embryonic lethal, abnormal vision, Drosophila)-like 1 (Hu antigen R)	1994	ENSG00000066044

Database:molecular function Name:small molecule binding ID:GO:0036094						
C=2595; O=16; E=8.32; R=1.92; rawP=0.0053; adjP=0.0297						
Index	UserID	Value	Gene Symbol	Gene Name	EntrezGene	Ensembl
1 ☐	Q12905	NA	ILF2	interleukin enhancer binding factor 2, 45kDa	3608	ENSG00000143621
2 ☐	P22626	NA	HNRNPA2B1	heterogeneous nuclear ribonucleoprotein A2/B1	3181	ENSG00000122566
3 ☐	P62834	NA	RAP1A	RAP1A, member of RAS oncogene family	5906	ENSG00000116473
4 ☐	Q99729	NA	HNRNPAB	heterogeneous nuclear ribonucleoprotein A/B	3182	ENSG00000197451
5 ☐	Q15717	NA	ELAVL1	ELAV (embryonic lethal, abnormal vision, Drosophila)-like 1 (Hu antigen R)	1994	ENSG00000066044
6 ☐	P00505	NA	GOT2	glutamic-oxaloacetic transaminase 2, mitochondrial (aspartate aminotransferase 2)	2806	ENSG00000125166
7 ☐	Q9NR31	NA	SAR1A	SAR1 homolog A (S. cerevisiae)	56681	ENSG00000079332
8 ☐	P30086	NA	PEBP1	phosphatidylethanolamine binding protein 1	5037	ENSG00000089220
9 ☐	P00338	NA	LDHA	lactate dehydrogenase A	3939	ENSG00000134333

Database:molecular function Name:small molecule binding ID:GO:0036094						
C=2595; O=16; E=8.32; R=1.92; rawP=0.0053; adjP=0.0297						
Index	UserID	Value	Gene Symbol	Gene Name	EntrezGene	Ensembl
10 ☐	P29558	NA	RBMS1	RNA binding motif, single stranded interacting protein 1	5937	ENSG00000153250
11 ☐	Q14103	NA	HNRNPD	heterogeneous nuclear ribonucleoprotein D (AU-rich element RNA binding protein 1, 37kDa)	3184	ENSG00000138668
12 ☐	P19338	NA	NCL	nucleolin	4691	ENSG00000115053
13 ☐	P48735	NA	IDH2	isocitrate dehydrogenase 2 (NADP+), mitochondrial	3418	ENSG00000182054
14 ☐	P07741	NA	APRT	adenine phosphoribosyltransferase	353	ENSG00000198931
15 ☐	O14979	NA	HNRPDL	heterogeneous nuclear ribonucleoprotein D-like	9987	ENSG00000152795
16 ☐	P61088	NA	UBE2N	ubiquitin-conjugating enzyme E2N	7334	ENSG00000177889

Database:molecular function Name:nucleoside phosphate binding ID:GO:1901265						
C=2404; O=15; E=7.70; R=1.95; rawP=0.0065; adjP=0.0313						
Index	UserID	Value	Gene Symbol	Gene Name	EntrezGene	Ensembl
1 ☐	Q12905	NA	ILF2	interleukin enhancer binding factor 2, 45kDa	3608	ENSG00000143621
2 ☐	P22626	NA	HNRNPA2B1	heterogeneous nuclear ribonucleoprotein A2/B1	3181	ENSG00000122566
3 ☐	P62834	NA	RAP1A	RAP1A, member of RAS oncogene family	5906	ENSG00000116473
4 ☐	Q99729	NA	HNRNPAB	heterogeneous nuclear ribonucleoprotein A/B	3182	ENSG00000197451
5 ☐	Q15717	NA	ELAVL1	ELAV (embryonic lethal, abnormal vision, Drosophila)-like 1 (Hu antigen R)	1994	ENSG00000066044
6 ☐	Q9NR31	NA	SAR1A	SAR1 homolog A (S. cerevisiae)	56681	ENSG00000079332
7 ☐	P30086	NA	PEBP1	phosphatidylethanolamine binding protein 1	5037	ENSG00000089220
8 ☐	P00338	NA	LDHA	lactate dehydrogenase A	3939	ENSG00000134333
9 ☐	P29558	NA	RBMS1	RNA binding motif, single stranded interacting protein 1	5937	ENSG00000153250
10 ☐	Q14103	NA	HNRNPD	heterogeneous nuclear ribonucleoprotein D (AU-rich element RNA binding protein 1, 37kDa)	3184	ENSG00000138668

Database:molecular function			Name:nucleoside phosphate binding		ID:GO:1901265	
C=2404; O=15; E=7.70; R=1.95; rawP=0.0065; adjP=0.0313						
Index	UserID	Value	Gene Symbol	Gene Name	EntrezGene	Ensembl
11 ☐	P19338	NA	NCL	nucleolin	4691	ENSG00000115053
12 ☐	P48735	NA	IDH2	isocitrate dehydrogenase 2 (NADP+), mitochondrial	3418	ENSG00000182054
13 ☐	P07741	NA	APRT	adenine phosphoribosyltransferase	353	ENSG00000198931
14 ☐	O14979	NA	HNRPDL	heterogeneous nuclear ribonucleoprotein D-like	9987	ENSG00000152795
15 ☐	P61088	NA	UBE2N	ubiquitin-conjugating enzyme E2N	7334	ENSG00000177889

Database:molecular function			Name:nucleotide binding		ID:GO:0000166	
C=2403; O=15; E=7.70; R=1.95; rawP=0.0065; adjP=0.0313						
Index	UserID	Value	Gene Symbol	Gene Name	EntrezGene	Ensembl
1 ☐	Q12905	NA	ILF2	interleukin enhancer binding factor 2, 45kDa	3608	ENSG00000143621
2 ☐	P22626	NA	HNRNPA2B1	heterogeneous nuclear ribonucleoprotein A2/B1	3181	ENSG00000122566
3 ☐	P62834	NA	RAP1A	RAP1A, member of RAS oncogene family	5906	ENSG00000116473
4 ☐	Q99729	NA	HNRNPAB	heterogeneous nuclear ribonucleoprotein A/B	3182	ENSG00000197451
5 ☐	Q15717	NA	ELAVL1	ELAV (embryonic lethal, abnormal vision, Drosophila)-like 1 (Hu antigen R)	1994	ENSG00000066044
6 ☐	Q9NR31	NA	SAR1A	SAR1 homolog A (S. cerevisiae)	56681	ENSG00000079332
7 ☐	P30086	NA	PEBP1	phosphatidylethanolamine binding protein 1	5037	ENSG00000089220
8 ☐	P00338	NA	LDHA	lactate dehydrogenase A	3939	ENSG00000134333
9 ☐	P29558	NA	RBMS1	RNA binding motif, single stranded interacting protein 1	5937	ENSG00000153250
10 ☐	Q14103	NA	HNRNPD	heterogeneous nuclear ribonucleoprotein D (AU-rich element RNA binding protein 1, 37kDa)	3184	ENSG00000138668
11 ☐	P19338	NA	NCL	nucleolin	4691	ENSG00000115053
12 ☐	P48735	NA	IDH2	isocitrate dehydrogenase 2 (NADP+), mitochondrial	3418	ENSG00000182054
13 ☐	P07741	NA	APRT	adenine phosphoribosyltransferase	353	ENSG00000198931
14 ☐	O14979	NA	HNRPDL		9987	ENSG00000152795

Database:molecular function Name:nucleotide binding ID:GO:0000166						
C=2403; O=15; E=7.70; R=1.95; rawP=0.0065; adjP=0.0313						
Index	UserID	Value	Gene Symbol	Gene Name	EntrezGene	Ensembl
				heterogeneous nuclear ribonucleoprotein D-like		
15 ☐	P61088	NA	UBE2N	ubiquitin-conjugating enzyme E2N	7334	ENSG00000177889

Database:molecular function Name:unfolded protein binding ID:GO:0051082						
C=120; O=3; E=0.38; R=7.80; rawP=0.0067; adjP=0.0313						
Index	UserID	Value	Gene Symbol	Gene Name	EntrezGene	Ensembl
1 ☐	Q99471	NA	PFDN5	prefoldin subunit 5	5204	ENSG00000123349
2 ☐	Q15185	NA	PTGES3	prostaglandin E synthase 3 (cytosolic)	10728	ENSG00000110958
3 ☐	P62937	NA	PPIA	peptidylprolyl isomerase A (cyclophilin A)	5478	ENSG00000196262

Database:molecular function Name:single-stranded RNA binding ID:GO:0003727						
C=41; O=2; E=0.13; R=15.22; rawP=0.0076; adjP=0.0336						
Index	UserID	Value	Gene Symbol	Gene Name	EntrezGene	Ensembl
1 ☐	Q96SI9	NA	STRBP	spermatid perinuclear RNA binding protein	55342	ENSG00000165209
2 ☐	O14979	NA	HNRPDL	heterogeneous nuclear ribonucleoprotein D-like	9987	ENSG00000152795

Database:molecular function Name:lyase activity ID:GO:0016829						
C=157; O=3; E=0.50; R=5.96; rawP=0.0138; adjP=0.0580						
Index	UserID	Value	Gene Symbol	Gene Name	EntrezGene	Ensembl
1 ☐	Q04760	NA	GLO1	glyoxalase I	2739	ENSG00000124767
2 ☐	P09104	NA	ENO2	enolase 2 (gamma, neuronal)	2026	ENSG00000111674
3 ☐	P09972	NA	ALDOC	aldolase C, fructose-bisphosphate	230	ENSG00000109107

Database:molecular function Name:binding ID:GO:0005488						
C=11779; O=44; E=37.75; R=1.17; rawP=0.0146; adjP=0.0584						
Index	UserID	Value	Gene Symbol	Gene Name	EntrezGene	Ensembl
1 ☐	P14174	NA	MIF	macrophage migration inhibitory factor (glycosylation-inhibiting factor)	4282	ENSG00000240972
2 ☐	P10599	NA	TXN	thioredoxin	7295	ENSG00000136810
3 ☐	P09104	NA	ENO2	enolase 2 (gamma, neuronal)	2026	ENSG00000111674
4 ☐	Q99729	NA	HNRNPAB	heterogeneous nuclear ribonucleoprotein A/B	3182	ENSG00000197451
5 ☐	P61956	NA	SUMO2	SMT3 suppressor of mif two 3 homolog 2 (S. cerevisiae)	6613	ENSG00000188612
6 ☐	P30086	NA	PEBP1	phosphatidylethanolamine binding protein 1	5037	ENSG00000089220
7 ☐	P62633	NA	CNBP	CCHC-type zinc finger, nucleic acid binding protein	7555	ENSG00000169714
8 ☐	P30101	NA	PDIA3	protein disulfide isomerase family A, member 3	2923	ENSG00000167004
9 ☐	P07741	NA	APRT	adenine phosphoribosyltransferase	353	ENSG00000198931
10 ☐	P48735	NA	IDH2	isocitrate dehydrogenase 2 (NADP+), mitochondrial	3418	ENSG00000182054
11 ☐	P62937	NA	PPIA	peptidylprolyl isomerase A (cyclophilin A)	5478	ENSG00000196262
12 ☐	P13667	NA	PDIA4	protein disulfide isomerase family A, member 4	9601	ENSG00000155660
13 ☐	P22626	NA	HNRNPA2B1	heterogeneous nuclear ribonucleoprotein A2/B1	3181	ENSG00000122566
14 ☐	P62834	NA	RAP1A	RAP1A, member of RAS oncogene family	5906	ENSG00000116473
15 ☐	P60903	NA	S100A10	S100 calcium binding protein A10	6281	ENSG00000197747
16 ☐	Q96SI9	NA	STRBP	spermatid perinuclear RNA binding protein	55342	ENSG00000165209
17 ☐	P50395	NA	GDI2	GDP dissociation inhibitor 2	2665	ENSG00000057608
18 ☐	P00505	NA	GOT2	glutamic-oxaloacetic transaminase 2, mitochondrial (aspartate aminotransferase 2)	2806	ENSG00000125166
19 ☐	Q9NR31	NA	SAR1A	SAR1 homolog A (S. cerevisiae)	56681	ENSG00000079332

Database:molecular function Name:binding ID:GO:0005488						
C=11779; O=44; E=37.75; R=1.17; rawP=0.0146; adjP=0.0584						
Index	UserID	Value	Gene Symbol	Gene Name	EntrezGene	Ensembl
20 ☐	O15511	NA	ARPC5	actin related protein 2/3 complex, subunit 5, 16kDa	10092	ENSG00000162704
21 ☐	P07737	NA	PFN1	profilin 1	5216	ENSG00000108518
22 ☐	P09972	NA	ALDOC	aldolase C, fructose-bisphosphate	230	ENSG00000109107
23 ☐	O60493	NA	SNX3	sorting nexin 3	8724	ENSG00000112335
24 ☐	Q14103	NA	HNRNPD	heterogeneous nuclear ribonucleoprotein D (AU-rich element RNA binding protein 1, 37kDa)	3184	ENSG00000138668
25 ☐	O00299	NA	CLIC1	chloride intracellular channel 1	1192	ENSG00000213719
26 ☐	Q15185	NA	PTGES3	prostaglandin E synthase 3 (cytosolic)	10728	ENSG00000110958
27 ☐	Q15121	NA	PEA15	phosphoprotein enriched in astrocytes 15	8682	ENSG00000162734
28 ☐	Q12905	NA	ILF2	interleukin enhancer binding factor 2, 45kDa	3608	ENSG00000143621
29 ☐	P00338	NA	LDHA	lactate dehydrogenase A	3939	ENSG00000134333
30 ☐	Q12906	NA	ILF3	interleukin enhancer binding factor 3, 90kDa	3609	ENSG00000129351
31 ☐	P61326	NA	MAGOH	mago-nashi homolog, proliferation-associated (Drosophila)	4116	ENSG00000162385
32 ☐	P09382	NA	LGALS1	lectin, galactoside-binding, soluble, 1	3956	ENSG00000100097
33 ☐	Q01469	NA	FABP5	fatty acid binding protein 5 (psoriasis-associated)	2171	ENSG00000164687
34 ☐	Q04760	NA	GLO1	glyoxalase I	2739	ENSG00000124767
35 ☐	P19338	NA	NCL	nucleolin	4691	ENSG00000115053
36 ☐	Q14019	NA	COTL1	coactosin-like 1 (Dictyostelium)	23406	ENSG00000103187
37 ☐	Q9Y281	NA	CFL2	cofilin 2 (muscle)	1073	ENSG00000165410
38 ☐	Q15843	NA	NEDD8	neural precursor cell expressed, developmentally down-regulated 8	4738	ENSG00000129559
39 ☐	Q99471	NA	PFDN5	prefoldin subunit 5	5204	ENSG00000123349
40 ☐	Q15717	NA	ELAVL1	ELAV (embryonic lethal, abnormal vision, Drosophila)-like 1 (Hu antigen R)	1994	ENSG00000066044

Database:molecular function Name:binding ID:GO:0005488						
C=11779; O=44; E=37.75; R=1.17; rawP=0.0146; adjP=0.0584						
Index	UserID	Value	Gene Symbol	Gene Name	EntrezGene	Ensembl
41 ☐	P29558	NA	RBMS1	RNA binding motif, single stranded interacting protein 1	5937	ENSG00000153250
42 ☐	O14979	NA	HNRPDL	heterogeneous nuclear ribonucleoprotein D-like	9987	ENSG00000152795
43 ☐	P61088	NA	UBE2N	ubiquitin-conjugating enzyme E2N	7334	ENSG00000177889
44 ☐	P13693	NA	TPT1	tumor protein, translationally-controlled 1	7178	ENSG00000133112

Database:molecular function Name: ID:GO:0003697						
C=61; O=2; E=0.20; R=10.23; rawP=0.0163; adjP=0.0622						
Index	UserID	Value	Gene Symbol	Gene Name	EntrezGene	Ensembl
1 ☐	P29558	NA	RBMS1	RNA binding motif, single stranded interacting protein 1	5937	ENSG00000153250
2 ☐	O14979	NA	HNRPDL	heterogeneous nuclear ribonucleoprotein D-like	9987	ENSG00000152795

Database:molecular function Name:enzyme binding ID:GO:0019899						
C=1086; O=8; E=3.48; R=2.30; rawP=0.0210; adjP=0.0767						
Index	UserID	Value	Gene Symbol	Gene Name	EntrezGene	Ensembl
1 ☐	Q15843	NA	NEDD8	neural precursor cell expressed, developmentally down-regulated 8	4738	ENSG00000129559
2 ☐	Q14019	NA	COTL1	coactosin-like 1 (Dictyostelium)	23406	ENSG00000103187
3 ☐	P62834	NA	RAP1A	RAP1A, member of RAS oncogene family	5906	ENSG00000116473
4 ☐	Q15717	NA	ELAVL1	ELAV (embryonic lethal, abnormal vision, Drosophila)-like 1 (Hu antigen R)	1994	ENSG00000066044
5 ☐	P07737	NA	PFN1	profilin 1	5216	ENSG00000108518
6 ☐	O60493	NA	SNX3	sorting nexin 3	8724	ENSG00000112335
7 ☐	P61956	NA	SUMO2	SMT3 suppressor of mif two 3 homolog 2 (S. cerevisiae)	6613	ENSG00000188612

Database:molecular function Name:enzyme binding ID:GO:0019899						
C=1086; O=8; E=3.48; R=2.30; rawP=0.0210; adjP=0.0767						
Index	UserID	Value	Gene Symbol	Gene Name	EntrezGene	Ensembl
8 ☐	P30086	NA	PEBP1	phosphatidylethanolamine binding protein 1	5037	ENSG00000089220

Database:molecular function Name:structure-specific DNA binding ID:GO:0043566						
C=192; O=3; E=0.62; R=4.88; rawP=0.0235; adjP=0.0790						
Index	UserID	Value	Gene Symbol	Gene Name	EntrezGene	Ensembl
1 ☐	P29558	NA	RBMS1	RNA binding motif, single stranded interacting protein 1	5937	ENSG00000153250
2 ☐	P22626	NA	HNRNPA2B1	heterogeneous nuclear ribonucleoprotein A2/B1	3181	ENSG00000122566
3 ☐	O14979	NA	HNRPDL	heterogeneous nuclear ribonucleoprotein D-like	9987	ENSG00000152795

Database:molecular function Name:oxidoreductase activity ID:GO:0016491						
C=697; O=6; E=2.23; R=2.69; rawP=0.0231; adjP=0.0790						
Index	UserID	Value	Gene Symbol	Gene Name	EntrezGene	Ensembl
1 ☐	P30101	NA	PDIA3	protein disulfide isomerase family A, member 3	2923	ENSG00000167004
2 ☐	P48735	NA	IDH2	isocitrate dehydrogenase 2 (NADP+), mitochondrial	3418	ENSG00000182054
3 ☐	P10599	NA	TXN	thioredoxin	7295	ENSG00000136810
4 ☐	P00338	NA	LDHA	lactate dehydrogenase A	3939	ENSG00000134333
5 ☐	Q8NBS9	NA	TXNDC5	thioredoxin domain containing 5 (endoplasmic reticulum)	81567	ENSG00000239264
6 ☐	P13667	NA	PDIA4	protein disulfide isomerase family A, member 4	9601	ENSG00000155660

**Database:molecular function Name:actin binding ID:GO:0003779**						
C=353; O=4; E=1.13; R=3.54; rawP=0.0262; adjP=0.0846						
Index	UserID	Value	Gene Symbol	Gene Name	EntrezGene	Ensembl
1 ☐	Q14019	NA	COTL1	coactosin-like 1 (Dictyostelium)	23406	ENSG00000103187
2 ☐	O15511	NA	ARPC5	actin related protein 2/3 complex, subunit 5, 16kDa	10092	ENSG00000162704
3 ☐	P07737	NA	PFN1	profilin 1	5216	ENSG00000108518
4 ☐	Q9Y281	NA	CFL2	cofilin 2 (muscle)	1073	ENSG00000165410

**Database:cellular component Name:cytoplasm ID:GO:0005737**						
C=9051; O=45; E=26.59; R=1.69; rawP=1.11e-08; adjP=7.66e-07						
Index	UserID	Value	Gene Symbol	Gene Name	EntrezGene	Ensembl
1 ☐	P14174	NA	MIF	macrophage migration inhibitory factor (glycosylation-inhibiting factor)	4282	ENSG00000240972
2 ☐	P20962	NA	PTMS	parathymosin	5763	ENSG00000159335
3 ☐	P10599	NA	TXN	thioredoxin	7295	ENSG00000136810
4 ☐	P09104	NA	ENO2	enolase 2 (gamma, neuronal)	2026	ENSG00000111674
5 ☐	Q99729	NA	HNRNPAB	heterogeneous nuclear ribonucleoprotein A/B	3182	ENSG00000197451
6 ☐	P30086	NA	PEBP1	phosphatidylethanolamine binding protein 1	5037	ENSG00000089220
7 ☐	P62633	NA	CNBP	CCHC-type zinc finger, nucleic acid binding protein	7555	ENSG00000169714
8 ☐	P30101	NA	PDIA3	protein disulfide isomerase family A, member 3	2923	ENSG00000167004
9 ☐	P07741	NA	APRT	adenine phosphoribosyltransferase	353	ENSG00000198931
10 ☐	P48735	NA	IDH2	isocitrate dehydrogenase 2 (NADP+), mitochondrial	3418	ENSG00000182054
11 ☐	P30040	NA	ERP29	endoplasmic reticulum protein 29	10961	ENSG00000089248
12 ☐	P62937	NA	PPIA	peptidylprolyl isomerase A (cyclophilin A)	5478	ENSG00000196262
13 ☐	Q8NBS9	NA	TXNDC5	thioredoxin domain containing 5 (endoplasmic reticulum)	81567	ENSG00000239264
14 ☐	P13667	NA	PDIA4		9601	ENSG00000155660

Database:cellular component Name:cytoplasm ID:GO:0005737						
C=9051; O=45; E=26.59; R=1.69; rawP=1.11e-08; adjP=7.66e-07						
Index	UserID	Value	Gene Symbol	Gene Name	EntrezGene	Ensembl
				protein disulfide isomerase family A, member 4		
15 ☐	P22626	NA	HNRNPA2B1	heterogeneous nuclear ribonucleoprotein A2/B1	3181	ENSG00000122566
16 ☐	P62834	NA	RAP1A	RAP1A, member of RAS oncogene family	5906	ENSG00000116473
17 ☐	Q96SI9	NA	STRBP	spermatid perinuclear RNA binding protein	55342	ENSG00000165209
18 ☐	P50395	NA	GDI2	GDP dissociation inhibitor 2	2665	ENSG00000057608
19 ☐	P00505	NA	GOT2	glutamic-oxaloacetic transaminase 2, mitochondrial (aspartate aminotransferase 2)	2806	ENSG00000125166
20 ☐	Q9NR31	NA	SAR1A	SAR1 homolog A (S. cerevisiae)	56681	ENSG00000079332
21 ☐	O15511	NA	ARPC5	actin related protein 2/3 complex, subunit 5, 16kDa	10092	ENSG00000162704
22 ☐	P07737	NA	PFN1	profilin 1	5216	ENSG00000108518
23 ☐	P09972	NA	ALDOC	aldolase C, fructose-bisphosphate	230	ENSG00000109107
24 ☐	O60493	NA	SNX3	sorting nexin 3	8724	ENSG00000112335
25 ☐	O75390	NA	CS	citrate synthase	1431	ENSG00000062485
26 ☐	Q14103	NA	HNRNPD	heterogeneous nuclear ribonucleoprotein D (AU-rich element RNA binding protein 1, 37kDa)	3184	ENSG00000138668
27 ☐	O00299	NA	CLIC1	chloride intracellular channel 1	1192	ENSG00000213719
28 ☐	Q15185	NA	PTGES3	prostaglandin E synthase 3 (cytosolic)	10728	ENSG00000110958
29 ☐	Q15121	NA	PEA15	phosphoprotein enriched in astrocytes 15	8682	ENSG00000162734
30 ☐	Q12905	NA	ILF2	interleukin enhancer binding factor 2, 45kDa	3608	ENSG00000143621
31 ☐	P09382	NA	LGALS1	lectin, galactoside-binding, soluble, 1	3956	ENSG00000100097
32 ☐	P61326	NA	MAGOH	mago-nashi homolog, proliferation-associated (Drosophila)	4116	ENSG00000162385
33 ☐	P00338	NA	LDHA	lactate dehydrogenase A	3939	ENSG00000134333
34 ☐	Q12906	NA	ILF3	interleukin enhancer binding factor 3, 90kDa	3609	ENSG00000129351

Database:cellular component Name:cytoplasm ID:GO:0005737						
C=9051; O=45; E=26.59; R=1.69; rawP=1.11e-08; adjP=7.66e-07						
Index	UserID	Value	Gene Symbol	Gene Name	EntrezGene	Ensembl
35 ☐	Q01469	NA	FABP5	fatty acid binding protein 5 (psoriasis-associated)	2171	ENSG00000164687
36 ☐	Q04760	NA	GLO1	glyoxalase I	2739	ENSG00000124767
37 ☐	P19338	NA	NCL	nucleolin	4691	ENSG00000115053
38 ☐	Q14019	NA	COTL1	coactosin-like 1 (Dictyostelium)	23406	ENSG00000103187
39 ☐	Q9Y281	NA	CFL2	cofilin 2 (muscle)	1073	ENSG00000165410
40 ☐	Q99471	NA	PFDN5	prefoldin subunit 5	5204	ENSG00000123349
41 ☐	Q15717	NA	ELAVL1	ELAV (embryonic lethal, abnormal vision, Drosophila)-like 1 (Hu antigen R)	1994	ENSG00000066044
42 ☐	Q9BTT0	NA	ANP32E	acidic (leucine-rich) nuclear phosphoprotein 32 family, member E	81611	ENSG00000143401
43 ☐	O14979	NA	HNRPDL	heterogeneous nuclear ribonucleoprotein D-like	9987	ENSG00000152795
44 ☐	P61088	NA	UBE2N	ubiquitin-conjugating enzyme E2N	7334	ENSG00000177889
45 ☐	P13693	NA	TPT1	tumor protein, translationally-controlled 1	7178	ENSG00000133112

Database:cellular component Name:intracellular part ID:GO:0044424						
C=12096; O=48; E=35.53; R=1.35; rawP=2.76e-06; adjP=9.52e-05						
Index	UserID	Value	Gene Symbol	Gene Name	EntrezGene	Ensembl
1 ☐	P14174	NA	MIF	macrophage migration inhibitory factor (glycosylation-inhibiting factor)	4282	ENSG00000240972
2 ☐	P20962	NA	PTMS	parathymosin	5763	ENSG00000159335
3 ☐	P10599	NA	TXN	thioredoxin	7295	ENSG00000136810
4 ☐	P09104	NA	ENO2	enolase 2 (gamma, neuronal)	2026	ENSG00000111674
5 ☐	Q99729	NA	HNRNPAB	heterogeneous nuclear ribonucleoprotein A/B	3182	ENSG00000197451
6 ☐	P61956	NA	SUMO2	SMT3 suppressor of mif two 3 homolog 2 (S. cerevisiae)	6613	ENSG00000188612
7 ☐	P30086	NA	PEBP1	phosphatidylethanolamine binding protein 1	5037	ENSG00000089220
8 ☐	P62633	NA	CNBP		7555	ENSG00000169714

Database:cellular component Name:intracellular part ID:GO:0044424						
C=12096; O=48; E=35.53; R=1.35; rawP=2.76e-06; adjP=9.52e-05						
Index	UserID	Value	Gene Symbol	Gene Name	EntrezGene	Ensembl
				CCHC-type zinc finger, nucleic acid binding protein		
9 ☐	P30101	NA	PDIA3	protein disulfide isomerase family A, member 3	2923	ENSG00000167004
10 ☐	P07741	NA	APRT	adenine phosphoribosyltransferase	353	ENSG00000198931
11 ☐	P48735	NA	IDH2	isocitrate dehydrogenase 2 (NADP+), mitochondrial	3418	ENSG00000182054
12 ☐	P30040	NA	ERP29	endoplasmic reticulum protein 29	10961	ENSG00000089248
13 ☐	P62937	NA	PPIA	peptidylprolyl isomerase A (cyclophilin A)	5478	ENSG00000196262
14 ☐	Q8NBS9	NA	TXNDC5	thioredoxin domain containing 5 (endoplasmic reticulum)	81567	ENSG00000239264
15 ☐	P13667	NA	PDIA4	protein disulfide isomerase family A, member 4	9601	ENSG00000155660
16 ☐	P22626	NA	HNRNPA2B1	heterogeneous nuclear ribonucleoprotein A2/B1	3181	ENSG00000122566
17 ☐	P62834	NA	RAP1A	RAP1A, member of RAS oncogene family	5906	ENSG00000116473
18 ☐	Q96SI9	NA	STRBP	spermatid perinuclear RNA binding protein	55342	ENSG00000165209
19 ☐	P50395	NA	GDI2	GDP dissociation inhibitor 2	2665	ENSG00000057608
20 ☐	P00505	NA	GOT2	glutamic-oxaloacetic transaminase 2, mitochondrial (aspartate aminotransferase 2)	2806	ENSG00000125166
21 ☐	Q9NR31	NA	SAR1A	SAR1 homolog A (S. cerevisiae)	56681	ENSG00000079332
22 ☐	O15511	NA	ARPC5	actin related protein 2/3 complex, subunit 5, 16kDa	10092	ENSG00000162704
23 ☐	P07737	NA	PFN1	profilin 1	5216	ENSG00000108518
24 ☐	P09972	NA	ALDOC	aldolase C, fructose-bisphosphate	230	ENSG00000109107
25 ☐	O60493	NA	SNX3	sorting nexin 3	8724	ENSG00000112335
26 ☐	O75390	NA	CS	citrate synthase	1431	ENSG00000062485
27 ☐	Q14103	NA	HNRNPD	heterogeneous nuclear ribonucleoprotein D (AU-rich element RNA binding protein 1, 37kDa)	3184	ENSG00000138668

Database:cellular component Name:intracellular part ID:GO:0044424						
C=12096; O=48; E=35.53; R=1.35; rawP=2.76e-06; adjP=9.52e-05						
Index	UserID	Value	Gene Symbol	Gene Name	EntrezGene	Ensembl
28 ☐	O00299	NA	CLIC1	chloride intracellular channel 1	1192	ENSG00000213719
29 ☐	Q15185	NA	PTGES3	prostaglandin E synthase 3 (cytosolic)	10728	ENSG00000110958
30 ☐	Q15121	NA	PEA15	phosphoprotein enriched in astrocytes 15	8682	ENSG00000162734
31 ☐	Q12905	NA	ILF2	interleukin enhancer binding factor 2, 45kDa	3608	ENSG00000143621
32 ☐	P09382	NA	LGALS1	lectin, galactoside-binding, soluble, 1	3956	ENSG00000100097
33 ☐	P61326	NA	MAGOH	mago-nashi homolog, proliferation-associated (Drosophila)	4116	ENSG00000162385
34 ☐	Q12906	NA	ILF3	interleukin enhancer binding factor 3, 90kDa	3609	ENSG00000129351
35 ☐	P00338	NA	LDHA	lactate dehydrogenase A	3939	ENSG00000134333
36 ☐	Q01469	NA	FABP5	fatty acid binding protein 5 (psoriasis-associated)	2171	ENSG00000164687
37 ☐	Q04760	NA	GLO1	glyoxalase I	2739	ENSG00000124767
38 ☐	P19338	NA	NCL	nucleolin	4691	ENSG00000115053
39 ☐	Q14019	NA	COTL1	coactosin-like 1 (Dictyostelium)	23406	ENSG00000103187
40 ☐	Q9Y281	NA	CFL2	cofilin 2 (muscle)	1073	ENSG00000165410
41 ☐	Q15843	NA	NEDD8	neural precursor cell expressed, developmentally down-regulated 8	4738	ENSG00000129559
42 ☐	Q99471	NA	PFDN5	prefoldin subunit 5	5204	ENSG00000123349
43 ☐	Q15717	NA	ELAVL1	ELAV (embryonic lethal, abnormal vision, Drosophila)-like 1 (Hu antigen R)	1994	ENSG00000066044
44 ☐	P29558	NA	RBMS1	RNA binding motif, single stranded interacting protein 1	5937	ENSG00000153250
45 ☐	Q9BTT0	NA	ANP32E	acidic (leucine-rich) nuclear phosphoprotein 32 family, member E	81611	ENSG00000143401
46 ☐	O14979	NA	HNRPDL	heterogeneous nuclear ribonucleoprotein D-like	9987	ENSG00000152795
47 ☐	P61088	NA	UBE2N	ubiquitin-conjugating enzyme E2N	7334	ENSG00000177889
48 ☐	P13693	NA	TPT1		7178	ENSG00000133112

Database:cellular component Name:intracellular part ID:GO:0044424						
C=12096; O=48; E=35.53; R=1.35; rawP=2.76e-06; adjP=9.52e-05						
Index	UserID	Value	Gene Symbol	Gene Name	EntrezGene	Ensembl
				tumor protein, translationally-controlled 1		

Database:cellular component Name:intracellular ID:GO:0005622						
C=12412; O=48; E=36.46; R=1.32; rawP=8.93e-06; adjP=0.0002						
Index	UserID	Value	Gene Symbol	Gene Name	EntrezGene	Ensembl
1 ☐	P14174	NA	MIF	macrophage migration inhibitory factor (glycosylation-inhibiting factor)	4282	ENSG00000240972
2 ☐	P20962	NA	PTMS	parathymosin	5763	ENSG00000159335
3 ☐	P10599	NA	TXN	thioredoxin	7295	ENSG00000136810
4 ☐	P09104	NA	ENO2	enolase 2 (gamma, neuronal)	2026	ENSG00000111674
5 ☐	Q99729	NA	HNRNPAB	heterogeneous nuclear ribonucleoprotein A/B	3182	ENSG00000197451
6 ☐	P61956	NA	SUMO2	SMT3 suppressor of mif two 3 homolog 2 (S. cerevisiae)	6613	ENSG00000188612
7 ☐	P30086	NA	PEBP1	phosphatidylethanolamine binding protein 1	5037	ENSG00000089220
8 ☐	P62633	NA	CNBP	CCHC-type zinc finger, nucleic acid binding protein	7555	ENSG00000169714
9 ☐	P30101	NA	PDIA3	protein disulfide isomerase family A, member 3	2923	ENSG00000167004
10 ☐	P07741	NA	APRT	adenine phosphoribosyltransferase	353	ENSG00000198931
11 ☐	P48735	NA	IDH2	isocitrate dehydrogenase 2 (NADP+), mitochondrial	3418	ENSG00000182054
12 ☐	P30040	NA	ERP29	endoplasmic reticulum protein 29	10961	ENSG00000089248
13 ☐	P62937	NA	PPIA	peptidylprolyl isomerase A (cyclophilin A)	5478	ENSG00000196262
14 ☐	Q8NBS9	NA	TXNDC5	thioredoxin domain containing 5 (endoplasmic reticulum)	81567	ENSG00000239264
15 ☐	P13667	NA	PDIA4	protein disulfide isomerase family A, member 4	9601	ENSG00000155660
16 ☐	P22626	NA	HNRNPA2B1	heterogeneous nuclear ribonucleoprotein A2/B1	3181	ENSG00000122566

Database:cellular component Name:intracellular ID:GO:0005622						
C=12412; O=48; E=36.46; R=1.32; rawP=8.93e-06; adjP=0.0002						
Index	UserID	Value	Gene Symbol	Gene Name	EntrezGene	Ensembl
17 ☐	P62834	NA	RAP1A	RAP1A, member of RAS oncogene family	5906	ENSG00000116473
18 ☐	Q96SI9	NA	STRBP	spermatid perinuclear RNA binding protein	55342	ENSG00000165209
19 ☐	P50395	NA	GDI2	GDP dissociation inhibitor 2	2665	ENSG00000057608
20 ☐	P00505	NA	GOT2	glutamic-oxaloacetic transaminase 2, mitochondrial (aspartate aminotransferase 2)	2806	ENSG00000125166
21 ☐	Q9NR31	NA	SAR1A	SAR1 homolog A (S. cerevisiae)	56681	ENSG00000079332
22 ☐	O15511	NA	ARPC5	actin related protein 2/3 complex, subunit 5, 16kDa	10092	ENSG00000162704
23 ☐	P07737	NA	PFN1	profilin 1	5216	ENSG00000108518
24 ☐	P09972	NA	ALDOC	aldolase C, fructose-bisphosphate	230	ENSG00000109107
25 ☐	O60493	NA	SNX3	sorting nexin 3	8724	ENSG00000112335
26 ☐	O75390	NA	CS	citrate synthase	1431	ENSG00000062485
27 ☐	Q14103	NA	HNRNPD	heterogeneous nuclear ribonucleoprotein D (AU-rich element RNA binding protein 1, 37kDa)	3184	ENSG00000138668
28 ☐	O00299	NA	CLIC1	chloride intracellular channel 1	1192	ENSG00000213719
29 ☐	Q15185	NA	PTGES3	prostaglandin E synthase 3 (cytosolic)	10728	ENSG00000110958
30 ☐	Q15121	NA	PEA15	phosphoprotein enriched in astrocytes 15	8682	ENSG00000162734
31 ☐	Q12905	NA	ILF2	interleukin enhancer binding factor 2, 45kDa	3608	ENSG00000143621
32 ☐	P09382	NA	LGALS1	lectin, galactoside-binding, soluble, 1	3956	ENSG00000100097
33 ☐	P61326	NA	MAGOH	mago-nashi homolog, proliferation-associated (Drosophila)	4116	ENSG00000162385
34 ☐	Q12906	NA	ILF3	interleukin enhancer binding factor 3, 90kDa	3609	ENSG00000129351
35 ☐	P00338	NA	LDHA	lactate dehydrogenase A	3939	ENSG00000134333
36 ☐	Q01469	NA	FABP5	fatty acid binding protein 5 (psoriasis-associated)	2171	ENSG00000164687
37 ☐	Q04760	NA	GLO1	glyoxalase I	2739	ENSG00000124767

Database:cellular component Name:intracellular ID:GO:0005622						
C=12412; O=48; E=36.46; R=1.32; rawP=8.93e-06; adjP=0.0002						
Index	UserID	Value	Gene Symbol	Gene Name	EntrezGene	Ensembl
38 ☐	P19338	NA	NCL	nucleolin	4691	ENSG00000115053
39 ☐	Q14019	NA	COTL1	coactosin-like 1 (Dictyostelium)	23406	ENSG00000103187
40 ☐	Q9Y281	NA	CFL2	cofilin 2 (muscle)	1073	ENSG00000165410
41 ☐	Q15843	NA	NEDD8	neural precursor cell expressed, developmentally down-regulated 8	4738	ENSG00000129559
42 ☐	Q99471	NA	PFDN5	prefoldin subunit 5	5204	ENSG00000123349
43 ☐	Q15717	NA	ELAVL1	ELAV (embryonic lethal, abnormal vision, Drosophila)-like 1 (Hu antigen R)	1994	ENSG00000066044
44 ☐	P29558	NA	RBMS1	RNA binding motif, single stranded interacting protein 1	5937	ENSG00000153250
45 ☐	Q9BTT0	NA	ANP32E	acidic (leucine-rich) nuclear phosphoprotein 32 family, member E	81611	ENSG00000143401
46 ☐	O14979	NA	HNRPDL	heterogeneous nuclear ribonucleoprotein D-like	9987	ENSG00000152795
47 ☐	P61088	NA	UBE2N	ubiquitin-conjugating enzyme E2N	7334	ENSG00000177889
48 ☐	P13693	NA	TPT1	tumor protein, translationally-controlled 1	7178	ENSG00000133112

Database:cellular component Name:intracellular organelle ID:GO:0043229						
C=10521; O=43; E=30.90; R=1.39; rawP=0.0001; adjP=0.0014						
Index	UserID	Value	Gene Symbol	Gene Name	EntrezGene	Ensembl
1 ☐	P20962	NA	PTMS	parathymosin	5763	ENSG00000159335
2 ☐	P10599	NA	TXN	thioredoxin	7295	ENSG00000136810
3 ☐	Q99729	NA	HNRNPAB	heterogeneous nuclear ribonucleoprotein A/B	3182	ENSG00000197451
4 ☐	P61956	NA	SUMO2	SMT3 suppressor of mif two 3 homolog 2 (S. cerevisiae)	6613	ENSG00000188612
5 ☐	P30086	NA	PEBP1	phosphatidylethanolamine binding protein 1	5037	ENSG00000089220
6 ☐	P62633	NA	CNBP	CCHC-type zinc finger, nucleic acid binding protein	7555	ENSG00000169714
7 ☐	P30101	NA	PDIA3		2923	ENSG00000167004

Database:cellular component			Name:intracellular organelle		ID:GO:0043229	
C=10521; O=43; E=30.90; R=1.39; rawP=0.0001; adjP=0.0014						
Index	UserID	Value	Gene Symbol	Gene Name	EntrezGene	Ensembl
				protein disulfide isomerase family A, member 3		
8 ☐	P07741	NA	APRT	adenine phosphoribosyltransferase	353	ENSG00000198931
9 ☐	P48735	NA	IDH2	isocitrate dehydrogenase 2 (NADP+), mitochondrial	3418	ENSG00000182054
10 ☐	P30040	NA	ERP29	endoplasmic reticulum protein 29	10961	ENSG00000089248
11 ☐	P62937	NA	PPIA	peptidylprolyl isomerase A (cyclophilin A)	5478	ENSG00000196262
12 ☐	Q8NBS9	NA	TXNDC5	thioredoxin domain containing 5 (endoplasmic reticulum)	81567	ENSG00000239264
13 ☐	P13667	NA	PDIA4	protein disulfide isomerase family A, member 4	9601	ENSG00000155660
14 ☐	P22626	NA	HNRNPA2B1	heterogeneous nuclear ribonucleoprotein A2/B1	3181	ENSG00000122566
15 ☐	Q96SI9	NA	STRBP	spermatid perinuclear RNA binding protein	55342	ENSG00000165209
16 ☐	P50395	NA	GDI2	GDP dissociation inhibitor 2	2665	ENSG00000057608
17 ☐	P00505	NA	GOT2	glutamic-oxaloacetic transaminase 2, mitochondrial (aspartate aminotransferase 2)	2806	ENSG00000125166
18 ☐	O15511	NA	ARPC5	actin related protein 2/3 complex, subunit 5, 16kDa	10092	ENSG00000162704
19 ☐	Q9NR31	NA	SAR1A	SAR1 homolog A (S. cerevisiae)	56681	ENSG00000079332
20 ☐	P07737	NA	PFN1	profilin 1	5216	ENSG00000108518
21 ☐	P09972	NA	ALDOC	aldolase C, fructose-bisphosphate	230	ENSG00000109107
22 ☐	O60493	NA	SNX3	sorting nexin 3	8724	ENSG00000112335
23 ☐	O75390	NA	CS	citrate synthase	1431	ENSG00000062485
24 ☐	Q14103	NA	HNRNPD	heterogeneous nuclear ribonucleoprotein D (AU-rich element RNA binding protein 1, 37kDa)	3184	ENSG00000138668
25 ☐	O00299	NA	CLIC1	chloride intracellular channel 1	1192	ENSG00000213719
26 ☐	Q15185	NA	PTGES3	prostaglandin E synthase 3 (cytosolic)	10728	ENSG00000110958

Database:cellular component Name:intracellular organelle ID:GO:0043229						
C=10521; O=43; E=30.90; R=1.39; rawP=0.0001; adjP=0.0014						
Index	UserID	Value	Gene Symbol	Gene Name	EntrezGene	Ensembl
27 ☐	Q15121	NA	PEA15	phosphoprotein enriched in astrocytes 15	8682	ENSG00000162734
28 ☐	Q12905	NA	ILF2	interleukin enhancer binding factor 2, 45kDa	3608	ENSG00000143621
29 ☐	P00338	NA	LDHA	lactate dehydrogenase A	3939	ENSG00000134333
30 ☐	Q12906	NA	ILF3	interleukin enhancer binding factor 3, 90kDa	3609	ENSG00000129351
31 ☐	P61326	NA	MAGOH	mago-nashi homolog, proliferation-associated (Drosophila)	4116	ENSG00000162385
32 ☐	P09382	NA	LGALS1	lectin, galactoside-binding, soluble, 1	3956	ENSG00000100097
33 ☐	P19338	NA	NCL	nucleolin	4691	ENSG00000115053
34 ☐	Q14019	NA	COTL1	coactosin-like 1 (Dictyostelium)	23406	ENSG00000103187
35 ☐	Q9Y281	NA	CFL2	cofilin 2 (muscle)	1073	ENSG00000165410
36 ☐	Q15843	NA	NEDD8	neural precursor cell expressed, developmentally down-regulated 8	4738	ENSG00000129559
37 ☐	Q99471	NA	PFDN5	prefoldin subunit 5	5204	ENSG00000123349
38 ☐	Q15717	NA	ELAVL1	ELAV (embryonic lethal, abnormal vision, Drosophila)-like 1 (Hu antigen R)	1994	ENSG00000066044
39 ☐	P29558	NA	RBMS1	RNA binding motif, single stranded interacting protein 1	5937	ENSG00000153250
40 ☐	Q9BTT0	NA	ANP32E	acidic (leucine-rich) nuclear phosphoprotein 32 family, member E	81611	ENSG00000143401
41 ☐	O14979	NA	HNRPDL	heterogeneous nuclear ribonucleoprotein D-like	9987	ENSG00000152795
42 ☐	P61088	NA	UBE2N	ubiquitin-conjugating enzyme E2N	7334	ENSG00000177889
43 ☐	P13693	NA	TPT1	tumor protein, translationally-controlled 1	7178	ENSG00000133112

Database:cellular component Name:organelle ID:GO:0043226						
C=10536; O=43; E=30.95; R=1.39; rawP=0.0001; adjP=0.0014						
Index	UserID	Value	Gene Symbol	Gene Name	EntrezGene	Ensembl
1 ☐	P20962	NA	PTMS	parathymosin	5763	ENSG00000159335

Database:cellular component Name:organelle ID:GO:0043226						
C=10536; O=43; E=30.95; R=1.39; rawP=0.0001; adjP=0.0014						
Index	UserID	Value	Gene Symbol	Gene Name	EntrezGene	Ensembl
2 ☐	P10599	NA	TXN	thioredoxin	7295	ENSG00000136810
3 ☐	Q99729	NA	HNRNPAB	heterogeneous nuclear ribonucleoprotein A/B	3182	ENSG00000197451
4 ☐	P61956	NA	SUMO2	SMT3 suppressor of mif two 3 homolog 2 (S. cerevisiae)	6613	ENSG00000188612
5 ☐	P30086	NA	PEBP1	phosphatidylethanolamine binding protein 1	5037	ENSG00000089220
6 ☐	P62633	NA	CNBP	CCHC-type zinc finger, nucleic acid binding protein	7555	ENSG00000169714
7 ☐	P30101	NA	PDIA3	protein disulfide isomerase family A, member 3	2923	ENSG00000167004
8 ☐	P07741	NA	APRT	adenine phosphoribosyltransferase	353	ENSG00000198931
9 ☐	P48735	NA	IDH2	isocitrate dehydrogenase 2 (NADP+), mitochondrial	3418	ENSG00000182054
10 ☐	P30040	NA	ERP29	endoplasmic reticulum protein 29	10961	ENSG00000089248
11 ☐	P62937	NA	PPIA	peptidylprolyl isomerase A (cyclophilin A)	5478	ENSG00000196262
12 ☐	Q8NBS9	NA	TXNDC5	thioredoxin domain containing 5 (endoplasmic reticulum)	81567	ENSG00000239264
13 ☐	P13667	NA	PDIA4	protein disulfide isomerase family A, member 4	9601	ENSG00000155660
14 ☐	P22626	NA	HNRNPA2B1	heterogeneous nuclear ribonucleoprotein A2/B1	3181	ENSG00000122566
15 ☐	Q96SI9	NA	STRBP	spermatid perinuclear RNA binding protein	55342	ENSG00000165209
16 ☐	P50395	NA	GDI2	GDP dissociation inhibitor 2	2665	ENSG00000057608
17 ☐	P00505	NA	GOT2	glutamic-oxaloacetic transaminase 2, mitochondrial (aspartate aminotransferase 2)	2806	ENSG00000125166
18 ☐	O15511	NA	ARPC5	actin related protein 2/3 complex, subunit 5, 16kDa	10092	ENSG00000162704
19 ☐	Q9NR31	NA	SAR1A	SAR1 homolog A (S. cerevisiae)	56681	ENSG00000079332
20 ☐	P07737	NA	PFN1	profilin 1	5216	ENSG00000108518
21 ☐	P09972	NA	ALDOC	aldolase C, fructose-bisphosphate	230	ENSG00000109107

Database:cellular component Name:organelle ID:GO:0043226						
C=10536; O=43; E=30.95; R=1.39; rawP=0.0001; adjP=0.0014						
Index	UserID	Value	Gene Symbol	Gene Name	EntrezGene	Ensembl
22 ☐	O60493	NA	SNX3	sorting nexin 3	8724	ENSG00000112335
23 ☐	O75390	NA	CS	citrate synthase	1431	ENSG00000062485
24 ☐	Q14103	NA	HNRNPD	heterogeneous nuclear ribonucleoprotein D (AU-rich element RNA binding protein 1, 37kDa)	3184	ENSG00000138668
25 ☐	O00299	NA	CLIC1	chloride intracellular channel 1	1192	ENSG00000213719
26 ☐	Q15185	NA	PTGES3	prostaglandin E synthase 3 (cytosolic)	10728	ENSG00000110958
27 ☐	Q15121	NA	PEA15	phosphoprotein enriched in astrocytes 15	8682	ENSG00000162734
28 ☐	Q12905	NA	ILF2	interleukin enhancer binding factor 2, 45kDa	3608	ENSG00000143621
29 ☐	P00338	NA	LDHA	lactate dehydrogenase A	3939	ENSG00000134333
30 ☐	Q12906	NA	ILF3	interleukin enhancer binding factor 3, 90kDa	3609	ENSG00000129351
31 ☐	P61326	NA	MAGOH	mago-nashi homolog, proliferation-associated (Drosophila)	4116	ENSG00000162385
32 ☐	P09382	NA	LGALS1	lectin, galactoside-binding, soluble, 1	3956	ENSG00000100097
33 ☐	P19338	NA	NCL	nucleolin	4691	ENSG00000115053
34 ☐	Q14019	NA	COTL1	coactosin-like 1 (Dictyostelium)	23406	ENSG00000103187
35 ☐	Q9Y281	NA	CFL2	cofilin 2 (muscle)	1073	ENSG00000165410
36 ☐	Q15843	NA	NEDD8	neural precursor cell expressed, developmentally down-regulated 8	4738	ENSG00000129559
37 ☐	Q99471	NA	PFDN5	prefoldin subunit 5	5204	ENSG00000123349
38 ☐	Q15717	NA	ELAVL1	ELAV (embryonic lethal, abnormal vision, Drosophila)-like 1 (Hu antigen R)	1994	ENSG00000066044
39 ☐	P29558	NA	RBMS1	RNA binding motif, single stranded interacting protein 1	5937	ENSG00000153250
40 ☐	Q9BTT0	NA	ANP32E	acidic (leucine-rich) nuclear phosphoprotein 32 family, member E	81611	ENSG00000143401
41 ☐	O14979	NA	HNRPDL	heterogeneous nuclear ribonucleoprotein D-like	9987	ENSG00000152795
42 ☐	P61088	NA	UBE2N		7334	ENSG00000177889

Database:cellular component Name:organelle ID:GO:0043226						
C=10536; O=43; E=30.95; R=1.39; rawP=0.0001; adjP=0.0014						
Index	UserID	Value	Gene Symbol	Gene Name	EntrezGene	Ensembl
				ubiquitin-conjugating enzyme E2N		
43 ☐	P13693	NA	TPT1	tumor protein, translationally-controlled 1	7178	ENSG00000133112

Database:cellular component Name:membrane-bounded organelle ID:GO:0043227						
C=9495; O=40; E=27.89; R=1.43; rawP=0.0002; adjP=0.0017						
Index	UserID	Value	Gene Symbol	Gene Name	EntrezGene	Ensembl
1 ☐	P20962	NA	PTMS	parathymosin	5763	ENSG00000159335
2 ☐	P10599	NA	TXN	thioredoxin	7295	ENSG00000136810
3 ☐	Q99729	NA	HNRNPAB	heterogeneous nuclear ribonucleoprotein A/B	3182	ENSG00000197451
4 ☐	P61956	NA	SUMO2	SMT3 suppressor of mif two 3 homolog 2 (S. cerevisiae)	6613	ENSG00000188612
5 ☐	P30086	NA	PEBP1	phosphatidylethanolamine binding protein 1	5037	ENSG00000089220
6 ☐	P62633	NA	CNBP	CCHC-type zinc finger, nucleic acid binding protein	7555	ENSG00000169714
7 ☐	P30101	NA	PDIA3	protein disulfide isomerase family A, member 3	2923	ENSG00000167004
8 ☐	P07741	NA	APRT	adenine phosphoribosyltransferase	353	ENSG00000198931
9 ☐	P48735	NA	IDH2	isocitrate dehydrogenase 2 (NADP+), mitochondrial	3418	ENSG00000182054
10 ☐	P30040	NA	ERP29	endoplasmic reticulum protein 29	10961	ENSG00000089248
11 ☐	P62937	NA	PPIA	peptidylprolyl isomerase A (cyclophilin A)	5478	ENSG00000196262
12 ☐	Q8NBS9	NA	TXNDC5	thioredoxin domain containing 5 (endoplasmic reticulum)	81567	ENSG00000239264
13 ☐	P13667	NA	PDIA4	protein disulfide isomerase family A, member 4	9601	ENSG00000155660
14 ☐	P22626	NA	HNRNPA2B1	heterogeneous nuclear ribonucleoprotein A2/B1	3181	ENSG00000122566
15 ☐	Q96SI9	NA	STRBP	spermatid perinuclear RNA binding protein	55342	ENSG00000165209
16 ☐	P50395	NA	GDI2	GDP dissociation inhibitor 2	2665	ENSG00000057608

Database:cellular component			Name:membrane-bounded organelle		ID:GO:0043227	
C=9495; O=40; E=27.89; R=1.43; rawP=0.0002; adjP=0.0017						
Index	UserID	Value	Gene Symbol	Gene Name	EntrezGene	Ensembl
17 ☐	Q9NR31	NA	SAR1A	SAR1 homolog A (S. cerevisiae)	56681	ENSG00000079332
18 ☐	P00505	NA	GOT2	glutamic-oxaloacetic transaminase 2, mitochondrial (aspartate aminotransferase 2)	2806	ENSG00000125166
19 ☐	P07737	NA	PFN1	profilin 1	5216	ENSG00000108518
20 ☐	P09972	NA	ALDOC	aldolase C, fructose-bisphosphate	230	ENSG00000109107
21 ☐	O60493	NA	SNX3	sorting nexin 3	8724	ENSG00000112335
22 ☐	O75390	NA	CS	citrate synthase	1431	ENSG00000062485
23 ☐	Q14103	NA	HNRNPD	heterogeneous nuclear ribonucleoprotein D (AU-rich element RNA binding protein 1, 37kDa)	3184	ENSG00000138668
24 ☐	O00299	NA	CLIC1	chloride intracellular channel 1	1192	ENSG00000213719
25 ☐	Q15185	NA	PTGES3	prostaglandin E synthase 3 (cytosolic)	10728	ENSG00000110958
26 ☐	Q12905	NA	ILF2	interleukin enhancer binding factor 2, 45kDa	3608	ENSG00000143621
27 ☐	P00338	NA	LDHA	lactate dehydrogenase A	3939	ENSG00000134333
28 ☐	Q12906	NA	ILF3	interleukin enhancer binding factor 3, 90kDa	3609	ENSG00000129351
29 ☐	P61326	NA	MAGOH	mago-nashi homolog, proliferation-associated (Drosophila)	4116	ENSG00000162385
30 ☐	P09382	NA	LGALS1	lectin, galactoside-binding, soluble, 1	3956	ENSG00000100097
31 ☐	P19338	NA	NCL	nucleolin	4691	ENSG00000115053
32 ☐	Q9Y281	NA	CFL2	cofilin 2 (muscle)	1073	ENSG00000165410
33 ☐	Q15843	NA	NEDD8	neural precursor cell expressed, developmentally down-regulated 8	4738	ENSG00000129559
34 ☐	Q99471	NA	PFDN5	prefoldin subunit 5	5204	ENSG00000123349
35 ☐	Q15717	NA	ELAVL1	ELAV (embryonic lethal, abnormal vision, Drosophila)-like 1 (Hu antigen R)	1994	ENSG00000066044
36 ☐	P29558	NA	RBMS1	RNA binding motif, single stranded interacting protein 1	5937	ENSG00000153250
37 ☐	Q9BTT0	NA	ANP32E		81611	ENSG00000143401

Database:cellular component			Name:membrane-bounded organelle			ID:GO:0043227
C=9495; O=40; E=27.89; R=1.43; rawP=0.0002; adjP=0.0017						
Index	UserID	Value	Gene Symbol	Gene Name	EntrezGene	Ensembl
				acidic (leucine-rich) nuclear phosphoprotein 32 family, member E		
38 ☐	O14979	NA	HNRPDL	heterogeneous nuclear ribonucleoprotein D-like	9987	ENSG00000152795
39 ☐	P61088	NA	UBE2N	ubiquitin-conjugating enzyme E2N	7334	ENSG00000177889
40 ☐	P13693	NA	TPT1	tumor protein, translationally-controlled 1	7178	ENSG00000133112

Database:cellular component			Name:ribonucleoprotein complex			ID:GO:0030529
C=544; O=8; E=1.60; R=5.01; rawP=0.0002; adjP=0.0017						
Index	UserID	Value	Gene Symbol	Gene Name	EntrezGene	Ensembl
1 ☐	Q12905	NA	ILF2	interleukin enhancer binding factor 2, 45kDa	3608	ENSG00000143621
2 ☐	Q14103	NA	HNRNPD	heterogeneous nuclear ribonucleoprotein D (AU-rich element RNA binding protein 1, 37kDa)	3184	ENSG00000138668
3 ☐	P22626	NA	HNRNPA2B1	heterogeneous nuclear ribonucleoprotein A2/B1	3181	ENSG00000122566
4 ☐	P19338	NA	NCL	nucleolin	4691	ENSG00000115053
5 ☐	Q15185	NA	PTGES3	prostaglandin E synthase 3 (cytosolic)	10728	ENSG00000110958
6 ☐	Q99729	NA	HNRNPAB	heterogeneous nuclear ribonucleoprotein A/B	3182	ENSG00000197451
7 ☐	P61326	NA	MAGOH	mago-nashi homolog, proliferation-associated (Drosophila)	4116	ENSG00000162385
8 ☐	Q12906	NA	ILF3	interleukin enhancer binding factor 3, 90kDa	3609	ENSG00000129351

Database:cellular component organelle			Name:intracellular membrane-bounded ID:GO:0043231			
C=9484; O=40; E=27.86; R=1.44; rawP=0.0002; adjP=0.0017						
Index	UserID	Value	Gene Symbol	Gene Name	EntrezGene	Ensembl
1 ☐	P20962	NA	PTMS	parathymosin	5763	ENSG00000159335
2 ☐	P10599	NA	TXN	thioredoxin	7295	ENSG00000136810
3 ☐	Q99729	NA	HNRNPAB	heterogeneous nuclear ribonucleoprotein A/B	3182	ENSG00000197451
4 ☐	P61956	NA	SUMO2	SMT3 suppressor of mif two 3 homolog 2 (S. cerevisiae)	6613	ENSG00000188612
5 ☐	P30086	NA	PEBP1	phosphatidylethanolamine binding protein 1	5037	ENSG00000089220
6 ☐	P62633	NA	CNBP	CCHC-type zinc finger, nucleic acid binding protein	7555	ENSG00000169714
7 ☐	P30101	NA	PDIA3	protein disulfide isomerase family A, member 3	2923	ENSG00000167004
8 ☐	P07741	NA	APRT	adenine phosphoribosyltransferase	353	ENSG00000198931
9 ☐	P48735	NA	IDH2	isocitrate dehydrogenase 2 (NADP+), mitochondrial	3418	ENSG00000182054
10 ☐	P30040	NA	ERP29	endoplasmic reticulum protein 29	10961	ENSG00000089248
11 ☐	P62937	NA	PPIA	peptidylprolyl isomerase A (cyclophilin A)	5478	ENSG00000196262
12 ☐	Q8NBS9	NA	TXNDC5	thioredoxin domain containing 5 (endoplasmic reticulum)	81567	ENSG00000239264
13 ☐	P13667	NA	PDIA4	protein disulfide isomerase family A, member 4	9601	ENSG00000155660
14 ☐	P22626	NA	HNRNPA2B1	heterogeneous nuclear ribonucleoprotein A2/B1	3181	ENSG00000122566
15 ☐	Q96SI9	NA	STRBP	spermatid perinuclear RNA binding protein	55342	ENSG00000165209
16 ☐	P50395	NA	GDI2	GDP dissociation inhibitor 2	2665	ENSG00000057608
17 ☐	Q9NR31	NA	SAR1A	SAR1 homolog A (S. cerevisiae)	56681	ENSG00000079332
18 ☐	P00505	NA	GOT2	glutamic-oxaloacetic transaminase 2, mitochondrial (aspartate aminotransferase 2)	2806	ENSG00000125166
19 ☐	P07737	NA	PFN1	profilin 1	5216	ENSG00000108518
20 ☐	P09972	NA	ALDOC	aldolase C, fructose-bisphosphate	230	ENSG00000109107

Database:cellular component Name:intracellular membrane-bounded organelle ID:GO:0043231						
C=9484; O=40; E=27.86; R=1.44; rawP=0.0002; adjP=0.0017						
Index	UserID	Value	Gene Symbol	Gene Name	EntrezGene	Ensembl
21 ☐	O60493	NA	SNX3	sorting nexin 3	8724	ENSG00000112335
22 ☐	O75390	NA	CS	citrate synthase	1431	ENSG00000062485
23 ☐	Q14103	NA	HNRNPD	heterogeneous nuclear ribonucleoprotein D (AU-rich element RNA binding protein 1, 37kDa)	3184	ENSG00000138668
24 ☐	O00299	NA	CLIC1	chloride intracellular channel 1	1192	ENSG00000213719
25 ☐	Q15185	NA	PTGES3	prostaglandin E synthase 3 (cytosolic)	10728	ENSG00000110958
26 ☐	Q12905	NA	ILF2	interleukin enhancer binding factor 2, 45kDa	3608	ENSG00000143621
27 ☐	P00338	NA	LDHA	lactate dehydrogenase A	3939	ENSG00000134333
28 ☐	Q12906	NA	ILF3	interleukin enhancer binding factor 3, 90kDa	3609	ENSG00000129351
29 ☐	P61326	NA	MAGOH	mago-nashi homolog, proliferation-associated (Drosophila)	4116	ENSG00000162385
30 ☐	P09382	NA	LGALS1	lectin, galactoside-binding, soluble, 1	3956	ENSG00000100097
31 ☐	P19338	NA	NCL	nucleolin	4691	ENSG00000115053
32 ☐	Q9Y281	NA	CFL2	cofilin 2 (muscle)	1073	ENSG00000165410
33 ☐	Q15843	NA	NEDD8	neural precursor cell expressed, developmentally down-regulated 8	4738	ENSG00000129559
34 ☐	Q99471	NA	PFDN5	prefoldin subunit 5	5204	ENSG00000123349
35 ☐	Q15717	NA	ELAVL1	ELAV (embryonic lethal, abnormal vision, Drosophila)-like 1 (Hu antigen R)	1994	ENSG00000066044
36 ☐	P29558	NA	RBMS1	RNA binding motif, single stranded interacting protein 1	5937	ENSG00000153250
37 ☐	Q9BTT0	NA	ANP32E	acidic (leucine-rich) nuclear phosphoprotein 32 family, member E	81611	ENSG00000143401
38 ☐	O14979	NA	HNRPDL	heterogeneous nuclear ribonucleoprotein D-like	9987	ENSG00000152795
39 ☐	P61088	NA	UBE2N	ubiquitin-conjugating enzyme E2N	7334	ENSG00000177889
40 ☐	P13693	NA	TPT1	tumor protein, translationally-controlled 1	7178	ENSG00000133112

Database:cellular component Name:cell surface ID:GO:0009986						
C=491; O=7; E=1.44; R=4.85; rawP=0.0005; adjP=0.0034						
Index	UserID	Value	Gene Symbol	Gene Name	EntrezGene	Ensembl
1 ☐	P30101	NA	PDIA3	protein disulfide isomerase family A, member 3	2923	ENSG00000167004
2 ☐	P14174	NA	MIF	macrophage migration inhibitory factor (glycosylation-inhibiting factor)	4282	ENSG00000240972
3 ☐	P50395	NA	GDI2	GDP dissociation inhibitor 2	2665	ENSG00000057608
4 ☐	P30040	NA	ERP29	endoplasmic reticulum protein 29	10961	ENSG00000089248
5 ☐	P30086	NA	PEBP1	phosphatidylethanolamine binding protein 1	5037	ENSG00000089220
6 ☐	P09382	NA	LGALS1	lectin, galactoside-binding, soluble, 1	3956	ENSG00000100097
7 ☐	P13667	NA	PDIA4	protein disulfide isomerase family A, member 4	9601	ENSG00000155660

Database:cellular component Name:intracellular organelle lumen ID:GO:0070013						
C=3285; O=20; E=9.65; R=2.07; rawP=0.0005; adjP=0.0034						
Index	UserID	Value	Gene Symbol	Gene Name	EntrezGene	Ensembl
1 ☐	Q12905	NA	ILF2	interleukin enhancer binding factor 2, 45kDa	3608	ENSG00000143621
2 ☐	P10599	NA	TXN	thioredoxin	7295	ENSG00000136810
3 ☐	Q99729	NA	HNRNPAB	heterogeneous nuclear ribonucleoprotein A/B	3182	ENSG00000197451
4 ☐	P61326	NA	MAGOH	mago-nashi homolog, proliferation-associated (Drosophila)	4116	ENSG00000162385
5 ☐	Q12906	NA	ILF3	interleukin enhancer binding factor 3, 90kDa	3609	ENSG00000129351
6 ☐	P30101	NA	PDIA3	protein disulfide isomerase family A, member 3	2923	ENSG00000167004
7 ☐	P19338	NA	NCL	nucleolin	4691	ENSG00000115053
8 ☐	P48735	NA	IDH2	isocitrate dehydrogenase 2 (NADP+), mitochondrial	3418	ENSG00000182054
9 ☐	P07741	NA	APRT	adenine phosphoribosyltransferase	353	ENSG00000198931
10 ☐	P30040	NA	ERP29	endoplasmic reticulum protein 29	10961	ENSG00000089248

Database:cellular component Name:intracellular organelle lumen ID:GO:0070013						
C=3285; O=20; E=9.65; R=2.07; rawP=0.0005; adjP=0.0034						
Index	UserID	Value	Gene Symbol	Gene Name	EntrezGene	Ensembl
11 ☐	Q9Y281	NA	CFL2	cofilin 2 (muscle)	1073	ENSG00000165410
12 ☐	P13667	NA	PDIA4	protein disulfide isomerase family A, member 4	9601	ENSG00000155660
13 ☐	Q8NBS9	NA	TXNDC5	thioredoxin domain containing 5 (endoplasmic reticulum)	81567	ENSG00000239264
14 ☐	P22626	NA	HNRNPA2B1	heterogeneous nuclear ribonucleoprotein A2/B1	3181	ENSG00000122566
15 ☐	Q15717	NA	ELAVL1	ELAV (embryonic lethal, abnormal vision, Drosophila)-like 1 (Hu antigen R)	1994	ENSG00000066044
16 ☐	P00505	NA	GOT2	glutamic-oxaloacetic transaminase 2, mitochondrial (aspartate aminotransferase 2)	2806	ENSG00000125166
17 ☐	O75390	NA	CS	citrate synthase	1431	ENSG00000062485
18 ☐	Q14103	NA	HNRNPD	heterogeneous nuclear ribonucleoprotein D (AU-rich element RNA binding protein 1, 37kDa)	3184	ENSG00000138668
19 ☐	Q15185	NA	PTGES3	prostaglandin E synthase 3 (cytosolic)	10728	ENSG00000110958
20 ☐	P13693	NA	TPT1	tumor protein, translationally-controlled 1	7178	ENSG00000133112

Database:cellular component Name:organelle lumen ID:GO:0043233						
C=3331; O=20; E=9.78; R=2.04; rawP=0.0007; adjP=0.0037						
Index	UserID	Value	Gene Symbol	Gene Name	EntrezGene	Ensembl
1 ☐	Q12905	NA	ILF2	interleukin enhancer binding factor 2, 45kDa	3608	ENSG00000143621
2 ☐	P10599	NA	TXN	thioredoxin	7295	ENSG00000136810
3 ☐	Q99729	NA	HNRNPAB	heterogeneous nuclear ribonucleoprotein A/B	3182	ENSG00000197451
4 ☐	P61326	NA	MAGOH	mago-nashi homolog, proliferation-associated (Drosophila)	4116	ENSG00000162385
5 ☐	Q12906	NA	ILF3	interleukin enhancer binding factor 3, 90kDa	3609	ENSG00000129351
6 ☐	P30101	NA	PDIA3	protein disulfide isomerase family A, member 3	2923	ENSG00000167004
7 ☐	P19338	NA	NCL	nucleolin	4691	ENSG00000115053

Database:cellular component Name:organelle lumen ID:GO:0043233						
C=3331; O=20; E=9.78; R=2.04; rawP=0.0007; adjP=0.0037						
Index	UserID	Value	Gene Symbol	Gene Name	EntrezGene	Ensembl
8 ☐	P48735	NA	IDH2	isocitrate dehydrogenase 2 (NADP+), mitochondrial	3418	ENSG00000182054
9 ☐	P07741	NA	APRT	adenine phosphoribosyltransferase	353	ENSG00000198931
10 ☐	P30040	NA	ERP29	endoplasmic reticulum protein 29	10961	ENSG00000089248
11 ☐	Q9Y281	NA	CFL2	cofilin 2 (muscle)	1073	ENSG00000165410
12 ☐	P13667	NA	PDIA4	protein disulfide isomerase family A, member 4	9601	ENSG00000155660
13 ☐	Q8NBS9	NA	TXNDC5	thioredoxin domain containing 5 (endoplasmic reticulum)	81567	ENSG00000239264
14 ☐	P22626	NA	HNRNPA2B1	heterogeneous nuclear ribonucleoprotein A2/B1	3181	ENSG00000122566
15 ☐	Q15717	NA	ELAVL1	ELAV (embryonic lethal, abnormal vision, Drosophila)-like 1 (Hu antigen R)	1994	ENSG00000066044
16 ☐	P00505	NA	GOT2	glutamic-oxaloacetic transaminase 2, mitochondrial (aspartate aminotransferase 2)	2806	ENSG00000125166
17 ☐	O75390	NA	CS	citrate synthase	1431	ENSG00000062485
18 ☐	Q14103	NA	HNRNPD	heterogeneous nuclear ribonucleoprotein D (AU-rich element RNA binding protein 1, 37kDa)	3184	ENSG00000138668
19 ☐	Q15185	NA	PTGES3	prostaglandin E synthase 3 (cytosolic)	10728	ENSG00000110958
20 ☐	P13693	NA	TPT1	tumor protein, translationally-controlled 1	7178	ENSG00000133112

Database:cellular component Name:cytosol ID:GO:0005829						
C=2367; O=16; E=6.95; R=2.30; rawP=0.0008; adjP=0.0037						
Index	UserID	Value	Gene Symbol	Gene Name	EntrezGene	Ensembl
1 ☐	Q99471	NA	PFDN5	prefoldin subunit 5	5204	ENSG00000123349
2 ☐	P20962	NA	PTMS	parathymosin	5763	ENSG00000159335
3 ☐	P62834	NA	RAP1A	RAP1A, member of RAS oncogene family	5906	ENSG00000116473
4 ☐	P10599	NA	TXN	thioredoxin	7295	ENSG00000136810
5 ☐	P09104	NA	ENO2		2026	ENSG00000111674

Database:cellular component Name:cytosol ID:GO:0005829						
C=2367; O=16; E=6.95; R=2.30; rawP=0.0008; adjP=0.0037						
Index	UserID	Value	Gene Symbol	Gene Name	EntrezGene	Ensembl
				enolase 2 (gamma, neuronal)		
6 ☐	Q15717	NA	ELAVL1	ELAV (embryonic lethal, abnormal vision, Drosophila)-like 1 (Hu antigen R)	1994	ENSG00000066044
7 ☐	P50395	NA	GDI2	GDP dissociation inhibitor 2	2665	ENSG00000057608
8 ☐	P09972	NA	ALDOC	aldolase C, fructose-bisphosphate	230	ENSG00000109107
9 ☐	P61326	NA	MAGOH	mago-nashi homolog, proliferation-associated (Drosophila)	4116	ENSG00000162385
10 ☐	P00338	NA	LDHA	lactate dehydrogenase A	3939	ENSG00000134333
11 ☐	P62633	NA	CNBP	CCHC-type zinc finger, nucleic acid binding protein	7555	ENSG00000169714
12 ☐	Q14103	NA	HNRNPD	heterogeneous nuclear ribonucleoprotein D (AU-rich element RNA binding protein 1, 37kDa)	3184	ENSG00000138668
13 ☐	P07741	NA	APRT	adenine phosphoribosyltransferase	353	ENSG00000198931
14 ☐	Q15185	NA	PTGES3	prostaglandin E synthase 3 (cytosolic)	10728	ENSG00000110958
15 ☐	P61088	NA	UBE2N	ubiquitin-conjugating enzyme E2N	7334	ENSG00000177889
16 ☐	P62937	NA	PPIA	peptidylprolyl isomerase A (cyclophilin A)	5478	ENSG00000196262

Database:cellular component Name:membrane-enclosed lumen ID:GO:0031974						
C=3375; O=20; E=9.91; R=2.02; rawP=0.0008; adjP=0.0037						
Index	UserID	Value	Gene Symbol	Gene Name	EntrezGene	Ensembl
1 ☐	Q12905	NA	ILF2	interleukin enhancer binding factor 2, 45kDa	3608	ENSG00000143621
2 ☐	P10599	NA	TXN	thioredoxin	7295	ENSG00000136810
3 ☐	Q99729	NA	HNRNPAB	heterogeneous nuclear ribonucleoprotein A/B	3182	ENSG00000197451
4 ☐	P61326	NA	MAGOH	mago-nashi homolog, proliferation-associated (Drosophila)	4116	ENSG00000162385
5 ☐	Q12906	NA	ILF3	interleukin enhancer binding factor 3, 90kDa	3609	ENSG00000129351
6 ☐	P30101	NA	PDIA3		2923	ENSG00000167004

Database:cellular component			Name:membrane-enclosed lumen		ID:GO:0031974	
C=3375; O=20; E=9.91; R=2.02; rawP=0.0008; adjP=0.0037						
Index	UserID	Value	Gene Symbol	Gene Name	EntrezGene	Ensembl
				protein disulfide isomerase family A, member 3		
7 ☐	P19338	NA	NCL	nucleolin	4691	ENSG00000115053
8 ☐	P48735	NA	IDH2	isocitrate dehydrogenase 2 (NADP+), mitochondrial	3418	ENSG00000182054
9 ☐	P07741	NA	APRT	adenine phosphoribosyltransferase	353	ENSG00000198931
10 ☐	P30040	NA	ERP29	endoplasmic reticulum protein 29	10961	ENSG00000089248
11 ☐	Q9Y281	NA	CFL2	cofilin 2 (muscle)	1073	ENSG00000165410
12 ☐	P13667	NA	PDIA4	protein disulfide isomerase family A, member 4	9601	ENSG00000155660
13 ☐	Q8NBS9	NA	TXNDC5	thioredoxin domain containing 5 (endoplasmic reticulum)	81567	ENSG00000239264
14 ☐	P22626	NA	HNRNPA2B1	heterogeneous nuclear ribonucleoprotein A2/B1	3181	ENSG00000122566
15 ☐	Q15717	NA	ELAVL1	ELAV (embryonic lethal, abnormal vision, Drosophila)-like 1 (Hu antigen R)	1994	ENSG00000066044
16 ☐	P00505	NA	GOT2	glutamic-oxaloacetic transaminase 2, mitochondrial (aspartate aminotransferase 2)	2806	ENSG00000125166
17 ☐	O75390	NA	CS	citrate synthase	1431	ENSG00000062485
18 ☐	Q14103	NA	HNRNPD	heterogeneous nuclear ribonucleoprotein D (AU-rich element RNA binding protein 1, 37kDa)	3184	ENSG00000138668
19 ☐	Q15185	NA	PTGES3	prostaglandin E synthase 3 (cytosolic)	10728	ENSG00000110958
20 ☐	P13693	NA	TPT1	tumor protein, translationally-controlled 1	7178	ENSG00000133112

Database:cellular component			Name:cell		ID:GO:0005623	
C=14406; O=49; E=42.31; R=1.16; rawP=0.0007; adjP=0.0037						
Index	UserID	Value	Gene Symbol	Gene Name	EntrezGene	Ensembl
1 ☐	P14174	NA	MIF	macrophage migration inhibitory factor (glycosylation-inhibiting factor)	4282	ENSG00000240972

Database:cellular component Name:cell ID:GO:0005623						
C=14406; O=49; E=42.31; R=1.16; rawP=0.0007; adjP=0.0037						
Index	UserID	Value	Gene Symbol	Gene Name	EntrezGene	Ensembl
2 ☐	P20962	NA	PTMS	parathymosin	5763	ENSG00000159335
3 ☐	P10599	NA	TXN	thioredoxin	7295	ENSG00000136810
4 ☐	P09104	NA	ENO2	enolase 2 (gamma, neuronal)	2026	ENSG00000111674
5 ☐	Q99729	NA	HNRNPAB	heterogeneous nuclear ribonucleoprotein A/B	3182	ENSG00000197451
6 ☐	P61956	NA	SUMO2	SMT3 suppressor of mif two 3 homolog 2 (S. cerevisiae)	6613	ENSG00000188612
7 ☐	P30086	NA	PEBP1	phosphatidylethanolamine binding protein 1	5037	ENSG00000089220
8 ☐	P62633	NA	CNBP	CCHC-type zinc finger, nucleic acid binding protein	7555	ENSG00000169714
9 ☐	P30101	NA	PDIA3	protein disulfide isomerase family A, member 3	2923	ENSG00000167004
10 ☐	P07741	NA	APRT	adenine phosphoribosyltransferase	353	ENSG00000198931
11 ☐	P48735	NA	IDH2	isocitrate dehydrogenase 2 (NADP+), mitochondrial	3418	ENSG00000182054
12 ☐	P30040	NA	ERP29	endoplasmic reticulum protein 29	10961	ENSG00000089248
13 ☐	P62937	NA	PPIA	peptidylprolyl isomerase A (cyclophilin A)	5478	ENSG00000196262
14 ☐	Q8NBS9	NA	TXNDC5	thioredoxin domain containing 5 (endoplasmic reticulum)	81567	ENSG00000239264
15 ☐	P13667	NA	PDIA4	protein disulfide isomerase family A, member 4	9601	ENSG00000155660
16 ☐	P22626	NA	HNRNPA2B1	heterogeneous nuclear ribonucleoprotein A2/B1	3181	ENSG00000122566
17 ☐	P62834	NA	RAP1A	RAP1A, member of RAS oncogene family	5906	ENSG00000116473
18 ☐	Q96SI9	NA	STRBP	spermatid perinuclear RNA binding protein	55342	ENSG00000165209
19 ☐	P60903	NA	S100A10	S100 calcium binding protein A10	6281	ENSG00000197747
20 ☐	P50395	NA	GDI2	GDP dissociation inhibitor 2	2665	ENSG00000057608
21 ☐	P00505	NA	GOT2	glutamic-oxaloacetic transaminase 2, mitochondrial (aspartate aminotransferase 2)	2806	ENSG00000125166

Database:cellular component Name:cell ID:GO:0005623						
C=14406; O=49; E=42.31; R=1.16; rawP=0.0007; adjP=0.0037						
Index	UserID	Value	Gene Symbol	Gene Name	EntrezGene	Ensembl
22 ☐	Q9NR31	NA	SAR1A	SAR1 homolog A (S. cerevisiae)	56681	ENSG00000079332
23 ☐	O15511	NA	ARPC5	actin related protein 2/3 complex, subunit 5, 16kDa	10092	ENSG00000162704
24 ☐	P07737	NA	PFN1	profilin 1	5216	ENSG00000108518
25 ☐	P09972	NA	ALDOC	aldolase C, fructose-bisphosphate	230	ENSG00000109107
26 ☐	O60493	NA	SNX3	sorting nexin 3	8724	ENSG00000112335
27 ☐	O75390	NA	CS	citrate synthase	1431	ENSG00000062485
28 ☐	Q14103	NA	HNRNPD	heterogeneous nuclear ribonucleoprotein D (AU-rich element RNA binding protein 1, 37kDa)	3184	ENSG00000138668
29 ☐	O00299	NA	CLIC1	chloride intracellular channel 1	1192	ENSG00000213719
30 ☐	Q15185	NA	PTGES3	prostaglandin E synthase 3 (cytosolic)	10728	ENSG00000110958
31 ☐	Q15121	NA	PEA15	phosphoprotein enriched in astrocytes 15	8682	ENSG00000162734
32 ☐	Q12905	NA	ILF2	interleukin enhancer binding factor 2, 45kDa	3608	ENSG00000143621
33 ☐	P09382	NA	LGALS1	lectin, galactoside-binding, soluble, 1	3956	ENSG00000100097
34 ☐	P61326	NA	MAGOH	mago-nashi homolog, proliferation-associated (Drosophila)	4116	ENSG00000162385
35 ☐	Q12906	NA	ILF3	interleukin enhancer binding factor 3, 90kDa	3609	ENSG00000129351
36 ☐	P00338	NA	LDHA	lactate dehydrogenase A	3939	ENSG00000134333
37 ☐	Q01469	NA	FABP5	fatty acid binding protein 5 (psoriasis-associated)	2171	ENSG00000164687
38 ☐	Q04760	NA	GLO1	glyoxalase I	2739	ENSG00000124767
39 ☐	P19338	NA	NCL	nucleolin	4691	ENSG00000115053
40 ☐	Q14019	NA	COTL1	coactosin-like 1 (Dictyostelium)	23406	ENSG00000103187
41 ☐	Q9Y281	NA	CFL2	cofilin 2 (muscle)	1073	ENSG00000165410
42 ☐	Q15843	NA	NEDD8	neural precursor cell expressed, developmentally down-regulated 8	4738	ENSG00000129559
43 ☐	Q99471	NA	PFDN5	prefoldin subunit 5	5204	ENSG00000123349

Database:cellular component Name:cell ID:GO:0005623						
C=14406; O=49; E=42.31; R=1.16; rawP=0.0007; adjP=0.0037						
Index	UserID	Value	Gene Symbol	Gene Name	EntrezGene	Ensembl
44 ☐	Q15717	NA	ELAVL1	ELAV (embryonic lethal, abnormal vision, Drosophila)-like 1 (Hu antigen R)	1994	ENSG00000066044
45 ☐	P29558	NA	RBMS1	RNA binding motif, single stranded interacting protein 1	5937	ENSG00000153250
46 ☐	Q9BTT0	NA	ANP32E	acidic (leucine-rich) nuclear phosphoprotein 32 family, member E	81611	ENSG00000143401
47 ☐	O14979	NA	HNRPDL	heterogeneous nuclear ribonucleoprotein D-like	9987	ENSG00000152795
48 ☐	P61088	NA	UBE2N	ubiquitin-conjugating enzyme E2N	7334	ENSG00000177889
49 ☐	P13693	NA	TPT1	tumor protein, translationally-controlled 1	7178	ENSG00000133112

Database:cellular component Name:cell part ID:GO:0044464						
C=14405; O=49; E=42.31; R=1.16; rawP=0.0007; adjP=0.0037						
Index	UserID	Value	Gene Symbol	Gene Name	EntrezGene	Ensembl
1 ☐	P14174	NA	MIF	macrophage migration inhibitory factor (glycosylation-inhibiting factor)	4282	ENSG00000240972
2 ☐	P20962	NA	PTMS	parathymosin	5763	ENSG00000159335
3 ☐	P10599	NA	TXN	thioredoxin	7295	ENSG00000136810
4 ☐	P09104	NA	ENO2	enolase 2 (gamma, neuronal)	2026	ENSG00000111674
5 ☐	Q99729	NA	HNRNPAB	heterogeneous nuclear ribonucleoprotein A/B	3182	ENSG00000197451
6 ☐	P61956	NA	SUMO2	SMT3 suppressor of mif two 3 homolog 2 (S. cerevisiae)	6613	ENSG00000188612
7 ☐	P30086	NA	PEBP1	phosphatidylethanolamine binding protein 1	5037	ENSG00000089220
8 ☐	P62633	NA	CNBP	CCHC-type zinc finger, nucleic acid binding protein	7555	ENSG00000169714
9 ☐	P30101	NA	PDIA3	protein disulfide isomerase family A, member 3	2923	ENSG00000167004
10 ☐	P07741	NA	APRT	adenine phosphoribosyltransferase	353	ENSG00000198931
11 ☐	P48735	NA	IDH2		3418	ENSG00000182054

Database:cellular component Name:cell part ID:GO:0044464						
C=14405; O=49; E=42.31; R=1.16; rawP=0.0007; adjP=0.0037						
Index	UserID	Value	Gene Symbol	Gene Name	EntrezGene	Ensembl
				isocitrate dehydrogenase 2 (NADP+), mitochondrial		
12 ☐	P30040	NA	ERP29	endoplasmic reticulum protein 29	10961	ENSG00000089248
13 ☐	P62937	NA	PPIA	peptidylprolyl isomerase A (cyclophilin A)	5478	ENSG00000196262
14 ☐	Q8NBS9	NA	TXNDC5	thioredoxin domain containing 5 (endoplasmic reticulum)	81567	ENSG00000239264
15 ☐	P13667	NA	PDIA4	protein disulfide isomerase family A, member 4	9601	ENSG00000155660
16 ☐	P22626	NA	HNRNPA2B1	heterogeneous nuclear ribonucleoprotein A2/B1	3181	ENSG00000122566
17 ☐	P62834	NA	RAP1A	RAP1A, member of RAS oncogene family	5906	ENSG00000116473
18 ☐	Q96SI9	NA	STRBP	spermatid perinuclear RNA binding protein	55342	ENSG00000165209
19 ☐	P60903	NA	S100A10	S100 calcium binding protein A10	6281	ENSG00000197747
20 ☐	P50395	NA	GDI2	GDP dissociation inhibitor 2	2665	ENSG00000057608
21 ☐	P00505	NA	GOT2	glutamic-oxaloacetic transaminase 2, mitochondrial (aspartate aminotransferase 2)	2806	ENSG00000125166
22 ☐	Q9NR31	NA	SAR1A	SAR1 homolog A (S. cerevisiae)	56681	ENSG00000079332
23 ☐	O15511	NA	ARPC5	actin related protein 2/3 complex, subunit 5, 16kDa	10092	ENSG00000162704
24 ☐	P07737	NA	PFN1	profilin 1	5216	ENSG00000108518
25 ☐	P09972	NA	ALDOC	aldolase C, fructose-bisphosphate	230	ENSG00000109107
26 ☐	O60493	NA	SNX3	sorting nexin 3	8724	ENSG00000112335
27 ☐	O75390	NA	CS	citrate synthase	1431	ENSG00000062485
28 ☐	Q14103	NA	HNRNPD	heterogeneous nuclear ribonucleoprotein D (AU-rich element RNA binding protein 1, 37kDa)	3184	ENSG00000138668
29 ☐	O00299	NA	CLIC1	chloride intracellular channel 1	1192	ENSG00000213719
30 ☐	Q15185	NA	PTGES3	prostaglandin E synthase 3 (cytosolic)	10728	ENSG00000110958
31 ☐	Q15121	NA	PEA15		8682	ENSG00000162734

Database:cellular component Name:cell part ID:GO:0044464						
C=14405; O=49; E=42.31; R=1.16; rawP=0.0007; adjP=0.0037						
Index	UserID	Value	Gene Symbol	Gene Name	EntrezGene	Ensembl
				phosphoprotein enriched in astrocytes 15		
32 ☐	Q12905	NA	ILF2	interleukin enhancer binding factor 2, 45kDa	3608	ENSG00000143621
33 ☐	P09382	NA	LGALS1	lectin, galactoside-binding, soluble, 1	3956	ENSG00000100097
34 ☐	P61326	NA	MAGOH	mago-nashi homolog, proliferation-associated (Drosophila)	4116	ENSG00000162385
35 ☐	Q12906	NA	ILF3	interleukin enhancer binding factor 3, 90kDa	3609	ENSG00000129351
36 ☐	P00338	NA	LDHA	lactate dehydrogenase A	3939	ENSG00000134333
37 ☐	Q01469	NA	FABP5	fatty acid binding protein 5 (psoriasis-associated)	2171	ENSG00000164687
38 ☐	Q04760	NA	GLO1	glyoxalase I	2739	ENSG00000124767
39 ☐	P19338	NA	NCL	nucleolin	4691	ENSG00000115053
40 ☐	Q14019	NA	COTL1	coactosin-like 1 (Dictyostelium)	23406	ENSG00000103187
41 ☐	Q9Y281	NA	CFL2	cofilin 2 (muscle)	1073	ENSG00000165410
42 ☐	Q15843	NA	NEDD8	neural precursor cell expressed, developmentally down-regulated 8	4738	ENSG00000129559
43 ☐	Q99471	NA	PFDN5	prefoldin subunit 5	5204	ENSG00000123349
44 ☐	Q15717	NA	ELAVL1	ELAV (embryonic lethal, abnormal vision, Drosophila)-like 1 (Hu antigen R)	1994	ENSG00000066044
45 ☐	P29558	NA	RBMS1	RNA binding motif, single stranded interacting protein 1	5937	ENSG00000153250
46 ☐	Q9BTT0	NA	ANP32E	acidic (leucine-rich) nuclear phosphoprotein 32 family, member E	81611	ENSG00000143401
47 ☐	O14979	NA	HNRPDL	heterogeneous nuclear ribonucleoprotein D-like	9987	ENSG00000152795
48 ☐	P61088	NA	UBE2N	ubiquitin-conjugating enzyme E2N	7334	ENSG00000177889
49 ☐	P13693	NA	TPT1	tumor protein, translationally-controlled 1	7178	ENSG00000133112

Database:cellular component Name:endoplasmic reticulum lumen ID:GO:0005788						
C=170; O=4; E=0.50; R=8.01; rawP=0.0015; adjP=0.0065						
Index	UserID	Value	Gene Symbol	Gene Name	EntrezGene	Ensembl
1 ☐	P30101	NA	PDIA3	protein disulfide isomerase family A, member 3	2923	ENSG00000167004
2 ☐	P30040	NA	ERP29	endoplasmic reticulum protein 29	10961	ENSG00000089248
3 ☐	Q8NBS9	NA	TXNDC5	thioredoxin domain containing 5 (endoplasmic reticulum)	81567	ENSG00000239264
4 ☐	P13667	NA	PDIA4	protein disulfide isomerase family A, member 4	9601	ENSG00000155660

Database:cellular component Name:cytoplasmic part ID:GO:0044444						
C=6728; O=30; E=19.76; R=1.52; rawP=0.0025; adjP=0.0094						
Index	UserID	Value	Gene Symbol	Gene Name	EntrezGene	Ensembl
1 ☐	P20962	NA	PTMS	parathymosin	5763	ENSG00000159335
2 ☐	P10599	NA	TXN	thioredoxin	7295	ENSG00000136810
3 ☐	P09104	NA	ENO2	enolase 2 (gamma, neuronal)	2026	ENSG00000111674
4 ☐	P30086	NA	PEBP1	phosphatidylethanolamine binding protein 1	5037	ENSG00000089220
5 ☐	P61326	NA	MAGOH	mago-nashi homolog, proliferation-associated (Drosophila)	4116	ENSG00000162385
6 ☐	Q12906	NA	ILF3	interleukin enhancer binding factor 3, 90kDa	3609	ENSG00000129351
7 ☐	P00338	NA	LDHA	lactate dehydrogenase A	3939	ENSG00000134333
8 ☐	P62633	NA	CNBP	CCHC-type zinc finger, nucleic acid binding protein	7555	ENSG00000169714
9 ☐	P30101	NA	PDIA3	protein disulfide isomerase family A, member 3	2923	ENSG00000167004
10 ☐	P19338	NA	NCL	nucleolin	4691	ENSG00000115053
11 ☐	P48735	NA	IDH2	isocitrate dehydrogenase 2 (NADP+), mitochondrial	3418	ENSG00000182054
12 ☐	P07741	NA	APRT	adenine phosphoribosyltransferase	353	ENSG00000198931
13 ☐	P30040	NA	ERP29	endoplasmic reticulum protein 29	10961	ENSG00000089248

Database:cellular component Name:cytoplasmic part ID:GO:0044444						
C=6728; O=30; E=19.76; R=1.52; rawP=0.0025; adjP=0.0094						
Index	UserID	Value	Gene Symbol	Gene Name	EntrezGene	Ensembl
14 ☐	P62937	NA	PPIA	peptidylprolyl isomerase A (cyclophilin A)	5478	ENSG00000196262
15 ☐	Q8NBS9	NA	TXNDC5	thioredoxin domain containing 5 (endoplasmic reticulum)	81567	ENSG00000239264
16 ☐	P13667	NA	PDIA4	protein disulfide isomerase family A, member 4	9601	ENSG00000155660
17 ☐	Q99471	NA	PFDN5	prefoldin subunit 5	5204	ENSG00000123349
18 ☐	P62834	NA	RAP1A	RAP1A, member of RAS oncogene family	5906	ENSG00000116473
19 ☐	Q15717	NA	ELAVL1	ELAV (embryonic lethal, abnormal vision, Drosophila)-like 1 (Hu antigen R)	1994	ENSG00000066044
20 ☐	Q9NR31	NA	SAR1A	SAR1 homolog A (S. cerevisiae)	56681	ENSG00000079332
21 ☐	P00505	NA	GOT2	glutamic-oxaloacetic transaminase 2, mitochondrial (aspartate aminotransferase 2)	2806	ENSG00000125166
22 ☐	P50395	NA	GDI2	GDP dissociation inhibitor 2	2665	ENSG00000057608
23 ☐	P09972	NA	ALDOC	aldolase C, fructose-bisphosphate	230	ENSG00000109107
24 ☐	O60493	NA	SNX3	sorting nexin 3	8724	ENSG00000112335
25 ☐	O75390	NA	CS	citrate synthase	1431	ENSG00000062485
26 ☐	Q14103	NA	HNRNPD	heterogeneous nuclear ribonucleoprotein D (AU-rich element RNA binding protein 1, 37kDa)	3184	ENSG00000138668
27 ☐	Q9BTT0	NA	ANP32E	acidic (leucine-rich) nuclear phosphoprotein 32 family, member E	81611	ENSG00000143401
28 ☐	Q15185	NA	PTGES3	prostaglandin E synthase 3 (cytosolic)	10728	ENSG00000110958
29 ☐	P61088	NA	UBE2N	ubiquitin-conjugating enzyme E2N	7334	ENSG00000177889
30 ☐	P13693	NA	TPT1	tumor protein, translationally-controlled 1	7178	ENSG00000133112

Database:cellular component Name:melanosome ID:GO:0042470						
C=93; O=3; E=0.27; R=10.98; rawP=0.0026; adjP=0.0094						
Index	UserID	Value	Gene Symbol	Gene Name	EntrezGene	Ensembl
1 ☐	P30101	NA	PDIA3	protein disulfide isomerase family A, member 3	2923	ENSG00000167004
2 ☐	P30040	NA	ERP29	endoplasmic reticulum protein 29	10961	ENSG00000089248
3 ☐	P13667	NA	PDIA4	protein disulfide isomerase family A, member 4	9601	ENSG00000155660

Database:cellular component Name:pigment granule ID:GO:0048770						
C=93; O=3; E=0.27; R=10.98; rawP=0.0026; adjP=0.0094						
Index	UserID	Value	Gene Symbol	Gene Name	EntrezGene	Ensembl
1 ☐	P30101	NA	PDIA3	protein disulfide isomerase family A, member 3	2923	ENSG00000167004
2 ☐	P30040	NA	ERP29	endoplasmic reticulum protein 29	10961	ENSG00000089248
3 ☐	P13667	NA	PDIA4	protein disulfide isomerase family A, member 4	9601	ENSG00000155660

Database:cellular component Name:nucleus ID:GO:0005634						
C=5883; O=27; E=17.28; R=1.56; rawP=0.0035; adjP=0.0121						
Index	UserID	Value	Gene Symbol	Gene Name	EntrezGene	Ensembl
1 ☐	Q12905	NA	ILF2	interleukin enhancer binding factor 2, 45kDa	3608	ENSG00000143621
2 ☐	P20962	NA	PTMS	parathymosin	5763	ENSG00000159335
3 ☐	P10599	NA	TXN	thioredoxin	7295	ENSG00000136810
4 ☐	Q99729	NA	HNRNPAB	heterogeneous nuclear ribonucleoprotein A/B	3182	ENSG00000197451
5 ☐	P61956	NA	SUMO2	SMT3 suppressor of mif two 3 homolog 2 (S. cerevisiae)	6613	ENSG00000188612
6 ☐	P09382	NA	LGALS1	lectin, galactoside-binding, soluble, 1	3956	ENSG00000100097
7 ☐	P61326	NA	MAGOH	mago-nashi homolog, proliferation-associated (Drosophila)	4116	ENSG00000162385
8 ☐	Q12906	NA	ILF3	interleukin enhancer binding factor 3, 90kDa	3609	ENSG00000129351
9 ☐	P62633	NA	CNBP		7555	ENSG00000169714

Database:cellular component Name:nucleus ID:GO:0005634						
C=5883; O=27; E=17.28; R=1.56; rawP=0.0035; adjP=0.0121						
Index	UserID	Value	Gene Symbol	Gene Name	EntrezGene	Ensembl
				CCHC-type zinc finger, nucleic acid binding protein		
10 ☐	P19338	NA	NCL	nucleolin	4691	ENSG00000115053
11 ☐	P07741	NA	APRT	adenine phosphoribosyltransferase	353	ENSG00000198931
12 ☐	Q9Y281	NA	CFL2	cofilin 2 (muscle)	1073	ENSG00000165410
13 ☐	P62937	NA	PPIA	peptidylprolyl isomerase A (cyclophilin A)	5478	ENSG00000196262
14 ☐	Q15843	NA	NEDD8	neural precursor cell expressed, developmentally down-regulated 8	4738	ENSG00000129559
15 ☐	P22626	NA	HNRNPA2B1	heterogeneous nuclear ribonucleoprotein A2/B1	3181	ENSG00000122566
16 ☐	Q99471	NA	PFDN5	prefoldin subunit 5	5204	ENSG00000123349
17 ☐	Q96SI9	NA	STRBP	spermatid perinuclear RNA binding protein	55342	ENSG00000165209
18 ☐	Q15717	NA	ELAVL1	ELAV (embryonic lethal, abnormal vision, Drosophila)-like 1 (Hu antigen R)	1994	ENSG00000066044
19 ☐	P07737	NA	PFN1	profilin 1	5216	ENSG00000108518
20 ☐	P29558	NA	RBMS1	RNA binding motif, single stranded interacting protein 1	5937	ENSG00000153250
21 ☐	Q14103	NA	HNRNPD	heterogeneous nuclear ribonucleoprotein D (AU-rich element RNA binding protein 1, 37kDa)	3184	ENSG00000138668
22 ☐	Q9BTT0	NA	ANP32E	acidic (leucine-rich) nuclear phosphoprotein 32 family, member E	81611	ENSG00000143401
23 ☐	O00299	NA	CLIC1	chloride intracellular channel 1	1192	ENSG00000213719
24 ☐	Q15185	NA	PTGES3	prostaglandin E synthase 3 (cytosolic)	10728	ENSG00000110958
25 ☐	O14979	NA	HNRPDL	heterogeneous nuclear ribonucleoprotein D-like	9987	ENSG00000152795
26 ☐	P61088	NA	UBE2N	ubiquitin-conjugating enzyme E2N	7334	ENSG00000177889
27 ☐	P13693	NA	TPT1	tumor protein, translationally-controlled 1	7178	ENSG00000133112

**Database:cellular component Name:catalytic step 2 spliceosome ID:GO:0071013**						
C=80; O=2; E=0.23; R=8.51; rawP=0.0231; adjP=0.0759						
Index	UserID	Value	Gene Symbol	Gene Name	EntrezGene	Ensembl
1 ☐	P22626	NA	HNRNPA2B1	heterogeneous nuclear ribonucleoprotein A2/B1	3181	ENSG00000122566
2 ☐	P61326	NA	MAGOH	mago-nashi homolog, proliferation-associated (Drosophila)	4116	ENSG00000162385

WebGestalt is currently developed and maintained by Jing Wang and Bing Zhang at the Zhang Lab. Other people who have made significant contribution to the project include Dexter Duncan, Stefan Kirov, Zhiao Shi, and Jay Snoddy.

Funding credits: NIH/NIAAA (U01 AA016662, U01 AA013512); NIH/NIDA (P01 DA015027); NIH/NIMH (P50 MH078028, P50 MH096972); NIH/NCI (U24 CA159988); NIH/NIGMS (R01 GM088822).
