# Supplementary material for: Expression of leukemia inhibitory factor in Müller glia cells is regulated by a redox-dependent mRNA stability mechanism
Source: BMC Biol. 2015 Apr 25;13:30. doi: 10.1186/s12915-015-0137-1 (PMC4462110; doi:10.1186/s12915-015-0137-1)
Supplement: Additional file 7: File S2. — Analysis of gene sets using the WEB-based Gene Set Analysis Toolkit. Genes encoding for proteins identified using R36 are listed according to their biological processes, molecular function, and cellular component. Links are provided to EntrezGene and Ensembl data bases for each gene. [file 12915_2015_137_MOESM7_ESM.pdf]

WEB-based GEne SeT AnaLysis Toolkit

WebGestalt *Translating gene lists into biological insights...*

User data and parameters: User data: textAreaUpload.txt, Organism: hsapiens, Id Type: uniprot_swissprot_accession, Ref Set: entrezgene_protein-coding, Significance Level: .1, Statistics Test: Hypergeometric, MTC: BH, Minimum: 2

The results for the enriched GO category are listed in this table. For each GO category, the first row lists its sub-root (biological process, molecular function, or cellular component), category name, and corresponding GO ID.
The second row lists the following statistics:

- C: the number of reference genes in the category
- O: the number of genes in the gene set and also in the category
- E: the expected number in the category
- R: ratio of enrichment
- rawP: p value from hypergeometric test
- adjP: p value adjusted by the multiple test adjustment

Finally, genes in the category are listed. For each gene, the table lists the user uploaded ID and value (optional), Entrez ID, Ensembl Gene Stable ID, Gene symbol, and description. Ensembl Gene Stable ID and Entrez Gene ID are linked to the Ensembl and Entrez Gene databases, respectively.

**Database:biological process**			**Name:protein targeting to ER**		**ID:GO:0045047**	
C=106; O=9; E=0.36; R=24.99; rawP=7.13e-11; adjP=1.06e-08						
Index	UserID	Value	Gene Symbol	Gene Name	EntrezGene	Ensembl
1 ☐	P39023	NA	RPL3	ribosomal protein L3	6122	ENSG00000100316
2 ☐	P42766	NA	RPL35	ribosomal protein L35	11224	ENSG00000136942
3 ☐	P27635	NA	RPL10	ribosomal protein L10	6134	ENSG00000147403
4 ☐	P60059	NA	SEC61G	Sec61 gamma subunit	23480	ENSG00000132432
5 ☐	P84098	NA	RPL19	ribosomal protein L19	6143	ENSG00000108298
6 ☐	Q02543	NA	RPL18A	ribosomal protein L18a	6142	ENSG00000105640
7 ☐	P40429	NA	RPL13A	ribosomal protein L13a	23521	ENSG00000142541
8 ☐	Q9Y3U8	NA	RPL36	ribosomal protein L36	25873	ENSG00000130255
9 ☐	P49207	NA	RPL34	ribosomal protein L34	6164	ENSG00000109475

**Database:biological process**			**Name:establishment of protein localization to endoplasmic reticulum**		**ID:GO:0072599**	
C=106; O=9; E=0.36; R=24.99; rawP=7.13e-11; adjP=1.06e-08						
Index	UserID	Value	Gene Symbol	Gene Name	EntrezGene	Ensembl
1 ☐	P39023	NA	RPL3	ribosomal protein L3	6122	ENSG00000100316
2 ☐	P42766	NA	RPL35		11224	ENSG00000136942

Database:biological process Name:establishment of protein localization to endoplasmic reticulum ID:GO:0072599						
C=106; O=9; E=0.36; R=24.99; rawP=7.13e-11; adjP=1.06e-08						
Index	UserID	Value	Gene Symbol	Gene Name	EntrezGene	Ensembl
				ribosomal protein L35		
3 ☐	P27635	NA	RPL10	ribosomal protein L10	6134	ENSG00000147403
4 ☐	P60059	NA	SEC61G	Sec61 gamma subunit	23480	ENSG00000132432
5 ☐	P84098	NA	RPL19	ribosomal protein L19	6143	ENSG00000108298
6 ☐	Q02543	NA	RPL18A	ribosomal protein L18a	6142	ENSG00000105640
7 ☐	P40429	NA	RPL13A	ribosomal protein L13a	23521	ENSG00000142541
8 ☐	Q9Y3U8	NA	RPL36	ribosomal protein L36	25873	ENSG00000130255
9 ☐	P49207	NA	RPL34	ribosomal protein L34	6164	ENSG00000109475

Database:biological process Name:cotranslational protein targeting to membrane ID:GO:0006613						
C=107; O=9; E=0.36; R=24.76; rawP=7.77e-11; adjP=1.06e-08						
Index	UserID	Value	Gene Symbol	Gene Name	EntrezGene	Ensembl
1 ☐	P39023	NA	RPL3	ribosomal protein L3	6122	ENSG00000100316
2 ☐	P42766	NA	RPL35	ribosomal protein L35	11224	ENSG00000136942
3 ☐	P27635	NA	RPL10	ribosomal protein L10	6134	ENSG00000147403
4 ☐	P60059	NA	SEC61G	Sec61 gamma subunit	23480	ENSG00000132432
5 ☐	P84098	NA	RPL19	ribosomal protein L19	6143	ENSG00000108298
6 ☐	Q02543	NA	RPL18A	ribosomal protein L18a	6142	ENSG00000105640
7 ☐	P40429	NA	RPL13A	ribosomal protein L13a	23521	ENSG00000142541
8 ☐	Q9Y3U8	NA	RPL36	ribosomal protein L36	25873	ENSG00000130255
9 ☐	P49207	NA	RPL34	ribosomal protein L34	6164	ENSG00000109475

**Database:biological process Name:SRP-dependent cotranslational protein targeting to membrane ID:GO:0006614**						
C=105; O=9; E=0.36; R=25.23; rawP=6.54e-11; adjP=1.06e-08						
Index	UserID	Value	Gene Symbol	Gene Name	EntrezGene	Ensembl
1 ☐	P39023	NA	RPL3	ribosomal protein L3	6122	ENSG00000100316
2 ☐	P42766	NA	RPL35	ribosomal protein L35	11224	ENSG00000136942
3 ☐	P27635	NA	RPL10	ribosomal protein L10	6134	ENSG00000147403
4 ☐	P60059	NA	SEC61G	Sec61 gamma subunit	23480	ENSG00000132432
5 ☐	P84098	NA	RPL19	ribosomal protein L19	6143	ENSG00000108298
6 ☐	Q02543	NA	RPL18A	ribosomal protein L18a	6142	ENSG00000105640
7 ☐	P40429	NA	RPL13A	ribosomal protein L13a	23521	ENSG00000142541
8 ☐	Q9Y3U8	NA	RPL36	ribosomal protein L36	25873	ENSG00000130255
9 ☐	P49207	NA	RPL34	ribosomal protein L34	6164	ENSG00000109475

**Database:biological process Name:protein localization to endoplasmic reticulum ID:GO:0070972**						
C=119; O=9; E=0.40; R=22.26; rawP=2.03e-10; adjP=2.21e-08						
Index	UserID	Value	Gene Symbol	Gene Name	EntrezGene	Ensembl
1 ☐	P39023	NA	RPL3	ribosomal protein L3	6122	ENSG00000100316
2 ☐	P42766	NA	RPL35	ribosomal protein L35	11224	ENSG00000136942
3 ☐	P27635	NA	RPL10	ribosomal protein L10	6134	ENSG00000147403
4 ☐	P60059	NA	SEC61G	Sec61 gamma subunit	23480	ENSG00000132432
5 ☐	P84098	NA	RPL19	ribosomal protein L19	6143	ENSG00000108298
6 ☐	Q02543	NA	RPL18A	ribosomal protein L18a	6142	ENSG00000105640
7 ☐	P40429	NA	RPL13A	ribosomal protein L13a	23521	ENSG00000142541
8 ☐	Q9Y3U8	NA	RPL36	ribosomal protein L36	25873	ENSG00000130255
9 ☐	P49207	NA	RPL34	ribosomal protein L34	6164	ENSG00000109475

Database:biological process Name:translational termination ID:GO:0006415						
C=91; O=8; E=0.31; R=25.87; rawP=6.70e-10; adjP=6.07e-08						
Index	UserID	Value	Gene Symbol	Gene Name	EntrezGene	Ensembl
1 ☐	P39023	NA	RPL3	ribosomal protein L3	6122	ENSG00000100316
2 ☐	P42766	NA	RPL35	ribosomal protein L35	11224	ENSG00000136942
3 ☐	P27635	NA	RPL10	ribosomal protein L10	6134	ENSG00000147403
4 ☐	P84098	NA	RPL19	ribosomal protein L19	6143	ENSG00000108298
5 ☐	Q02543	NA	RPL18A	ribosomal protein L18a	6142	ENSG00000105640
6 ☐	P40429	NA	RPL13A	ribosomal protein L13a	23521	ENSG00000142541
7 ☐	P49207	NA	RPL34	ribosomal protein L34	6164	ENSG00000109475
8 ☐	Q9Y3U8	NA	RPL36	ribosomal protein L36	25873	ENSG00000130255

Database:biological process Name:protein targeting to membrane ID:GO:0006612						
C=147; O=9; E=0.50; R=18.02; rawP=1.35e-09; adjP=1.05e-07						
Index	UserID	Value	Gene Symbol	Gene Name	EntrezGene	Ensembl
1 ☐	P39023	NA	RPL3	ribosomal protein L3	6122	ENSG00000100316
2 ☐	P42766	NA	RPL35	ribosomal protein L35	11224	ENSG00000136942
3 ☐	P27635	NA	RPL10	ribosomal protein L10	6134	ENSG00000147403
4 ☐	P60059	NA	SEC61G	Sec61 gamma subunit	23480	ENSG00000132432
5 ☐	P84098	NA	RPL19	ribosomal protein L19	6143	ENSG00000108298
6 ☐	Q02543	NA	RPL18A	ribosomal protein L18a	6142	ENSG00000105640
7 ☐	P40429	NA	RPL13A	ribosomal protein L13a	23521	ENSG00000142541
8 ☐	Q9Y3U8	NA	RPL36	ribosomal protein L36	25873	ENSG00000130255
9 ☐	P49207	NA	RPL34	ribosomal protein L34	6164	ENSG00000109475

**Database:biological process Name:establishment of protein localization to organelle ID:GO:0072594**						
C=208; O=10; E=0.71; R=14.15; rawP=1.58e-09; adjP=1.07e-07						
Index	UserID	Value	Gene Symbol	Gene Name	EntrezGene	Ensembl
1 ☐	P39023	NA	RPL3	ribosomal protein L3	6122	ENSG00000100316
2 ☐	P42766	NA	RPL35	ribosomal protein L35	11224	ENSG00000136942
3 ☐	P27635	NA	RPL10	ribosomal protein L10	6134	ENSG00000147403
4 ☐	Q9HAV7	NA	GRPEL1	GrpE-like 1, mitochondrial (E. coli)	80273	ENSG00000109519
5 ☐	P60059	NA	SEC61G	Sec61 gamma subunit	23480	ENSG00000132432
6 ☐	Q02543	NA	RPL18A	ribosomal protein L18a	6142	ENSG00000105640
7 ☐	P84098	NA	RPL19	ribosomal protein L19	6143	ENSG00000108298
8 ☐	P40429	NA	RPL13A	ribosomal protein L13a	23521	ENSG00000142541
9 ☐	Q9Y3U8	NA	RPL36	ribosomal protein L36	25873	ENSG00000130255
10 ☐	P49207	NA	RPL34	ribosomal protein L34	6164	ENSG00000109475

**Database:biological process Name:translational initiation ID:GO:0006413**						
C=152; O=9; E=0.52; R=17.43; rawP=1.81e-09; adjP=1.09e-07						
Index	UserID	Value	Gene Symbol	Gene Name	EntrezGene	Ensembl
1 ☐	P39023	NA	RPL3	ribosomal protein L3	6122	ENSG00000100316
2 ☐	P42766	NA	RPL35	ribosomal protein L35	11224	ENSG00000136942
3 ☐	P27635	NA	RPL10	ribosomal protein L10	6134	ENSG00000147403
4 ☐	P41567	NA	EIF1	eukaryotic translation initiation factor 1	10209	ENSG00000173812
5 ☐	P84098	NA	RPL19	ribosomal protein L19	6143	ENSG00000108298
6 ☐	Q02543	NA	RPL18A	ribosomal protein L18a	6142	ENSG00000105640
7 ☐	P40429	NA	RPL13A	ribosomal protein L13a	23521	ENSG00000142541
8 ☐	Q9Y3U8	NA	RPL36	ribosomal protein L36	25873	ENSG00000130255

Database:biological process Name:translational initiation ID:GO:0006413						
C=152; O=9; E=0.52; R=17.43; rawP=1.81e-09; adjP=1.09e-07						
Index	UserID	Value	Gene Symbol	Gene Name	EntrezGene	Ensembl
9 ☐	P49207	NA	RPL34	ribosomal protein L34	6164	ENSG00000109475

Database:biological process Name:translational elongation ID:GO:0006414						
C=108; O=8; E=0.37; R=21.80; rawP=2.66e-09; adjP=1.45e-07						
Index	UserID	Value	Gene Symbol	Gene Name	EntrezGene	Ensembl
1 ☐	P39023	NA	RPL3	ribosomal protein L3	6122	ENSG00000100316
2 ☐	P42766	NA	RPL35	ribosomal protein L35	11224	ENSG00000136942
3 ☐	P27635	NA	RPL10	ribosomal protein L10	6134	ENSG00000147403
4 ☐	P84098	NA	RPL19	ribosomal protein L19	6143	ENSG00000108298
5 ☐	Q02543	NA	RPL18A	ribosomal protein L18a	6142	ENSG00000105640
6 ☐	P40429	NA	RPL13A	ribosomal protein L13a	23521	ENSG00000142541
7 ☐	P49207	NA	RPL34	ribosomal protein L34	6164	ENSG00000109475
8 ☐	Q9Y3U8	NA	RPL36	ribosomal protein L36	25873	ENSG00000130255

Database:biological process Name:nuclear-transcribed mRNA catabolic process, nonsense-mediated decay ID:GO:0000184						
C=119; O=8; E=0.40; R=19.79; rawP=5.76e-09; adjP=2.85e-07						
Index	UserID	Value	Gene Symbol	Gene Name	EntrezGene	Ensembl
1 ☐	P39023	NA	RPL3	ribosomal protein L3	6122	ENSG00000100316
2 ☐	P42766	NA	RPL35	ribosomal protein L35	11224	ENSG00000136942
3 ☐	P27635	NA	RPL10	ribosomal protein L10	6134	ENSG00000147403
4 ☐	P84098	NA	RPL19	ribosomal protein L19	6143	ENSG00000108298
5 ☐	Q02543	NA	RPL18A	ribosomal protein L18a	6142	ENSG00000105640
6 ☐	P40429	NA	RPL13A	ribosomal protein L13a	23521	ENSG00000142541
7 ☐	P49207	NA	RPL34	ribosomal protein L34	6164	ENSG00000109475

Database:biological process Name:nuclear-transcribed mRNA catabolic process, nonsense-mediated decay ID:GO:0000184						
C=119; O=8; E=0.40; R=19.79; rawP=5.76e-09; adjP=2.85e-07						
Index	UserID	Value	Gene Symbol	Gene Name	EntrezGene	Ensembl
8 ☐	Q9Y3U8	NA	RPL36	ribosomal protein L36	25873	ENSG00000130255

Database:biological process Name:viral genome expression ID:GO:0019080						
C=152; O=8; E=0.52; R=15.49; rawP=3.96e-08; adjP=1.66e-06						
Index	UserID	Value	Gene Symbol	Gene Name	EntrezGene	Ensembl
1 ☐	P39023	NA	RPL3	ribosomal protein L3	6122	ENSG00000100316
2 ☐	P42766	NA	RPL35	ribosomal protein L35	11224	ENSG00000136942
3 ☐	P27635	NA	RPL10	ribosomal protein L10	6134	ENSG00000147403
4 ☐	P84098	NA	RPL19	ribosomal protein L19	6143	ENSG00000108298
5 ☐	Q02543	NA	RPL18A	ribosomal protein L18a	6142	ENSG00000105640
6 ☐	P40429	NA	RPL13A	ribosomal protein L13a	23521	ENSG00000142541
7 ☐	P49207	NA	RPL34	ribosomal protein L34	6164	ENSG00000109475
8 ☐	Q9Y3U8	NA	RPL36	ribosomal protein L36	25873	ENSG00000130255

Database:biological process Name:viral transcription ID:GO:0019083						
C=152; O=8; E=0.52; R=15.49; rawP=3.96e-08; adjP=1.66e-06						
Index	UserID	Value	Gene Symbol	Gene Name	EntrezGene	Ensembl
1 ☐	P39023	NA	RPL3	ribosomal protein L3	6122	ENSG00000100316
2 ☐	P42766	NA	RPL35	ribosomal protein L35	11224	ENSG00000136942
3 ☐	P27635	NA	RPL10	ribosomal protein L10	6134	ENSG00000147403
4 ☐	P84098	NA	RPL19	ribosomal protein L19	6143	ENSG00000108298
5 ☐	Q02543	NA	RPL18A	ribosomal protein L18a	6142	ENSG00000105640
6 ☐	P40429	NA	RPL13A	ribosomal protein L13a	23521	ENSG00000142541
7 ☐	P49207	NA	RPL34	ribosomal protein L34	6164	ENSG00000109475

Database:biological process Name:viral transcription ID:GO:0019083						
C=152; O=8; E=0.52; R=15.49; rawP=3.96e-08; adjP=1.66e-06						
Index	UserID	Value	Gene Symbol	Gene Name	EntrezGene	Ensembl
8 ☐	Q9Y3U8	NA	RPL36	ribosomal protein L36	25873	ENSG00000130255

Database:biological process Name:cellular protein complex disassembly ID:GO:0043624						
C=156; O=8; E=0.53; R=15.09; rawP=4.85e-08; adjP=1.88e-06						
Index	UserID	Value	Gene Symbol	Gene Name	EntrezGene	Ensembl
1 ☐	P39023	NA	RPL3	ribosomal protein L3	6122	ENSG00000100316
2 ☐	P42766	NA	RPL35	ribosomal protein L35	11224	ENSG00000136942
3 ☐	P27635	NA	RPL10	ribosomal protein L10	6134	ENSG00000147403
4 ☐	P84098	NA	RPL19	ribosomal protein L19	6143	ENSG00000108298
5 ☐	Q02543	NA	RPL18A	ribosomal protein L18a	6142	ENSG00000105640
6 ☐	P40429	NA	RPL13A	ribosomal protein L13a	23521	ENSG00000142541
7 ☐	P49207	NA	RPL34	ribosomal protein L34	6164	ENSG00000109475
8 ☐	Q9Y3U8	NA	RPL36	ribosomal protein L36	25873	ENSG00000130255

Database:biological process Name:protein complex disassembly ID:GO:0043241						
C=161; O=8; E=0.55; R=14.62; rawP=6.20e-08; adjP=2.25e-06						
Index	UserID	Value	Gene Symbol	Gene Name	EntrezGene	Ensembl
1 ☐	P39023	NA	RPL3	ribosomal protein L3	6122	ENSG00000100316
2 ☐	P42766	NA	RPL35	ribosomal protein L35	11224	ENSG00000136942
3 ☐	P27635	NA	RPL10	ribosomal protein L10	6134	ENSG00000147403
4 ☐	P84098	NA	RPL19	ribosomal protein L19	6143	ENSG00000108298
5 ☐	Q02543	NA	RPL18A	ribosomal protein L18a	6142	ENSG00000105640
6 ☐	P40429	NA	RPL13A	ribosomal protein L13a	23521	ENSG00000142541
7 ☐	P49207	NA	RPL34	ribosomal protein L34	6164	ENSG00000109475

Database:biological process Name:protein complex disassembly ID:GO:0043241						
C=161; O=8; E=0.55; R=14.62; rawP=6.20e-08; adjP=2.25e-06						
Index	UserID	Value	Gene Symbol	Gene Name	EntrezGene	Ensembl
8 ☐	Q9Y3U8	NA	RPL36	ribosomal protein L36	25873	ENSG00000130255

Database:biological process Name:intracellular transport ID:GO:0046907						
C=1127; O=17; E=3.83; R=4.44; rawP=7.89e-08; adjP=2.68e-06						
Index	UserID	Value	Gene Symbol	Gene Name	EntrezGene	Ensembl
1 ☐	P39023	NA	RPL3	ribosomal protein L3	6122	ENSG00000100316
2 ☐	P61106	NA	RAB14	RAB14, member RAS oncogene family	51552	ENSG00000119396
3 ☐	P42766	NA	RPL35	ribosomal protein L35	11224	ENSG00000136942
4 ☐	Q9HAV7	NA	GRPEL1	GrpE-like 1, mitochondrial (E. coli)	80273	ENSG00000109519
5 ☐	P60059	NA	SEC61G	Sec61 gamma subunit	23480	ENSG00000132432
6 ☐	P06748	NA	NPM1	nucleophosmin (nucleolar phosphoprotein B23, numatrin)	4869	ENSG00000181163
7 ☐	P61019	NA	RAB2A	RAB2A, member RAS oncogene family	5862	ENSG00000104388
8 ☐	Q02543	NA	RPL18A	ribosomal protein L18a	6142	ENSG00000105640
9 ☐	P30040	NA	ERP29	endoplasmic reticulum protein 29	10961	ENSG00000089248
10 ☐	P40429	NA	RPL13A	ribosomal protein L13a	23521	ENSG00000142541
11 ☐	Q9Y3U8	NA	RPL36	ribosomal protein L36	25873	ENSG00000130255
12 ☐	P49207	NA	RPL34	ribosomal protein L34	6164	ENSG00000109475
13 ☐	Q01130	NA	SRSF2	serine/arginine-rich splicing factor 2	6427	ENSG00000161547
14 ☐	P27635	NA	RPL10	ribosomal protein L10	6134	ENSG00000147403
15 ☐	P40616	NA	ARL1	ADP-ribosylation factor-like 1	400	ENSG00000120805
16 ☐	P84098	NA	RPL19		6143	ENSG00000108298

Database:biological process Name:intracellular transport ID:GO:0046907						
C=1127; O=17; E=3.83; R=4.44; rawP=7.89e-08; adjP=2.68e-06						
Index	UserID	Value	Gene Symbol	Gene Name	EntrezGene	Ensembl
				ribosomal protein L19		
17 ☐	O43747	NA	AP1G1	adaptor-related protein complex 1, gamma 1 subunit	164	ENSG00000166747

Database:biological process Name:nuclear-transcribed mRNA catabolic process ID:GO:0000956						
C=173; O=8; E=0.59; R=13.61; rawP=1.08e-07; adjP=3.45e-06						
Index	UserID	Value	Gene Symbol	Gene Name	EntrezGene	Ensembl
1 ☐	P39023	NA	RPL3	ribosomal protein L3	6122	ENSG00000100316
2 ☐	P42766	NA	RPL35	ribosomal protein L35	11224	ENSG00000136942
3 ☐	P27635	NA	RPL10	ribosomal protein L10	6134	ENSG00000147403
4 ☐	P84098	NA	RPL19	ribosomal protein L19	6143	ENSG00000108298
5 ☐	Q02543	NA	RPL18A	ribosomal protein L18a	6142	ENSG00000105640
6 ☐	P40429	NA	RPL13A	ribosomal protein L13a	23521	ENSG00000142541
7 ☐	P49207	NA	RPL34	ribosomal protein L34	6164	ENSG00000109475
8 ☐	Q9Y3U8	NA	RPL36	ribosomal protein L36	25873	ENSG00000130255

Database:biological process Name:nucleobase-containing compound catabolic process ID:GO:0034655						
C=749; O=14; E=2.54; R=5.50; rawP=1.14e-07; adjP=3.45e-06						
Index	UserID	Value	Gene Symbol	Gene Name	EntrezGene	Ensembl
1 ☐	P39023	NA	RPL3	ribosomal protein L3	6122	ENSG00000100316
2 ☐	P09429	NA	HMGB1	high mobility group box 1	3146	ENSG00000189403
3 ☐	P61106	NA	RAB14	RAB14, member RAS oncogene family	51552	ENSG00000119396
4 ☐	P42766	NA	RPL35	ribosomal protein L35	11224	ENSG00000136942
5 ☐	P27635	NA	RPL10	ribosomal protein L10	6134	ENSG00000147403

Database:biological process Name:nucleobase-containing compound catabolic process ID:GO:0034655						
C=749; O=14; E=2.54; R=5.50; rawP=1.14e-07; adjP=3.45e-06						
Index	UserID	Value	Gene Symbol	Gene Name	EntrezGene	Ensembl
6 ☐	P40616	NA	ARL1	ADP-ribosylation factor-like 1	400	ENSG00000120805
7 ☐	P61019	NA	RAB2A	RAB2A, member RAS oncogene family	5862	ENSG00000104388
8 ☐	Q02543	NA	RPL18A	ribosomal protein L18a	6142	ENSG00000105640
9 ☐	P84098	NA	RPL19	ribosomal protein L19	6143	ENSG00000108298
10 ☐	P09493	NA	TPM1	tropomyosin 1 (alpha)	7168	ENSG00000140416
11 ☐	P40429	NA	RPL13A	ribosomal protein L13a	23521	ENSG00000142541
12 ☐	Q13126	NA	MTAP	methylthioadenosine phosphorylase	4507	ENSG00000099810
13 ☐	Q9Y3U8	NA	RPL36	ribosomal protein L36	25873	ENSG00000130255
14 ☐	P49207	NA	RPL34	ribosomal protein L34	6164	ENSG00000109475

Database:biological process Name:cellular macromolecular complex disassembly ID:GO:0034623						
C=177; O=8; E=0.60; R=13.30; rawP=1.29e-07; adjP=3.69e-06						
Index	UserID	Value	Gene Symbol	Gene Name	EntrezGene	Ensembl
1 ☐	P39023	NA	RPL3	ribosomal protein L3	6122	ENSG00000100316
2 ☐	P42766	NA	RPL35	ribosomal protein L35	11224	ENSG00000136942
3 ☐	P27635	NA	RPL10	ribosomal protein L10	6134	ENSG00000147403
4 ☐	P84098	NA	RPL19	ribosomal protein L19	6143	ENSG00000108298
5 ☐	Q02543	NA	RPL18A	ribosomal protein L18a	6142	ENSG00000105640
6 ☐	P40429	NA	RPL13A	ribosomal protein L13a	23521	ENSG00000142541
7 ☐	P49207	NA	RPL34	ribosomal protein L34	6164	ENSG00000109475
8 ☐	Q9Y3U8	NA	RPL36	ribosomal protein L36	25873	ENSG00000130255

Database:biological process disassembly				Name:macromolecular complex ID:GO:0032984		
C=182; O=8; E=0.62; R=12.94; rawP=1.60e-07; adjP=4.35e-06						
Index	UserID	Value	Gene Symbol	Gene Name	EntrezGene	Ensembl
1 ☐	P39023	NA	RPL3	ribosomal protein L3	6122	ENSG00000100316
2 ☐	P42766	NA	RPL35	ribosomal protein L35	11224	ENSG00000136942
3 ☐	P27635	NA	RPL10	ribosomal protein L10	6134	ENSG00000147403
4 ☐	P84098	NA	RPL19	ribosomal protein L19	6143	ENSG00000108298
5 ☐	Q02543	NA	RPL18A	ribosomal protein L18a	6142	ENSG00000105640
6 ☐	P40429	NA	RPL13A	ribosomal protein L13a	23521	ENSG00000142541
7 ☐	P49207	NA	RPL34	ribosomal protein L34	6164	ENSG00000109475
8 ☐	Q9Y3U8	NA	RPL36	ribosomal protein L36	25873	ENSG00000130255

Database:biological process				Name:mRNA catabolic process		ID:GO:0006402
C=184; O=8; E=0.63; R=12.80; rawP=1.74e-07; adjP=4.51e-06						
Index	UserID	Value	Gene Symbol	Gene Name	EntrezGene	Ensembl
1 ☐	P39023	NA	RPL3	ribosomal protein L3	6122	ENSG00000100316
2 ☐	P42766	NA	RPL35	ribosomal protein L35	11224	ENSG00000136942
3 ☐	P27635	NA	RPL10	ribosomal protein L10	6134	ENSG00000147403
4 ☐	P84098	NA	RPL19	ribosomal protein L19	6143	ENSG00000108298
5 ☐	Q02543	NA	RPL18A	ribosomal protein L18a	6142	ENSG00000105640
6 ☐	P40429	NA	RPL13A	ribosomal protein L13a	23521	ENSG00000142541
7 ☐	P49207	NA	RPL34	ribosomal protein L34	6164	ENSG00000109475
8 ☐	Q9Y3U8	NA	RPL36	ribosomal protein L36	25873	ENSG00000130255

Database:biological process				Name:heterocycle catabolic process		ID:GO:0046700
C=791; O=14; E=2.69; R=5.21; rawP=2.23e-07; adjP=5.28e-06						
Index	UserID	Value	Gene Symbol	Gene Name	EntrezGene	Ensembl

Database:biological process			Name:heterocycle catabolic process			ID:GO:0046700
C=791; O=14; E=2.69; R=5.21; rawP=2.23e-07; adjP=5.28e-06						
Index	UserID	Value	Gene Symbol	Gene Name	EntrezGene	Ensembl
1 ☐	P39023	NA	RPL3	ribosomal protein L3	6122	ENSG00000100316
2 ☐	P09429	NA	HMGB1	high mobility group box 1	3146	ENSG00000189403
3 ☐	P61106	NA	RAB14	RAB14, member RAS oncogene family	51552	ENSG00000119396
4 ☐	P42766	NA	RPL35	ribosomal protein L35	11224	ENSG00000136942
5 ☐	P27635	NA	RPL10	ribosomal protein L10	6134	ENSG00000147403
6 ☐	P40616	NA	ARL1	ADP-ribosylation factor-like 1	400	ENSG00000120805
7 ☐	P61019	NA	RAB2A	RAB2A, member RAS oncogene family	5862	ENSG00000104388
8 ☐	Q02543	NA	RPL18A	ribosomal protein L18a	6142	ENSG00000105640
9 ☐	P84098	NA	RPL19	ribosomal protein L19	6143	ENSG00000108298
10 ☐	P09493	NA	TPM1	tropomyosin 1 (alpha)	7168	ENSG00000140416
11 ☐	P40429	NA	RPL13A	ribosomal protein L13a	23521	ENSG00000142541
12 ☐	Q13126	NA	MTAP	methylthioadenosine phosphorylase	4507	ENSG00000099810
13 ☐	Q9Y3U8	NA	RPL36	ribosomal protein L36	25873	ENSG00000130255
14 ☐	P49207	NA	RPL34	ribosomal protein L34	6164	ENSG00000109475

Database:biological process			Name:aromatic compound catabolic process			ID:GO:0019439
C=794; O=14; E=2.70; R=5.19; rawP=2.33e-07; adjP=5.28e-06						
Index	UserID	Value	Gene Symbol	Gene Name	EntrezGene	Ensembl
1 ☐	P39023	NA	RPL3	ribosomal protein L3	6122	ENSG00000100316
2 ☐	P09429	NA	HMGB1	high mobility group box 1	3146	ENSG00000189403
3 ☐	P61106	NA	RAB14	RAB14, member RAS oncogene family	51552	ENSG00000119396
4 ☐	P42766	NA	RPL35	ribosomal protein L35	11224	ENSG00000136942
5 ☐	P27635	NA	RPL10		6134	ENSG00000147403

Database:biological process	Name:aromatic compound catabolic process ID:GO:0019439					
C=794; O=14; E=2.70; R=5.19; rawP=2.33e-07; adjP=5.28e-06						
Index	UserID	Value	Gene Symbol	Gene Name	EntrezGene	Ensembl
				ribosomal protein L10		
6 ☐	P40616	NA	ARL1	ADP-ribosylation factor-like 1	400	ENSG00000120805
7 ☐	P61019	NA	RAB2A	RAB2A, member RAS oncogene family	5862	ENSG00000104388
8 ☐	Q02543	NA	RPL18A	ribosomal protein L18a	6142	ENSG00000105640
9 ☐	P84098	NA	RPL19	ribosomal protein L19	6143	ENSG00000108298
10 ☐	P09493	NA	TPM1	tropomyosin 1 (alpha)	7168	ENSG00000140416
11 ☐	P40429	NA	RPL13A	ribosomal protein L13a	23521	ENSG00000142541
12 ☐	Q13126	NA	MTAP	methylthioadenosine phosphorylase	4507	ENSG00000099810
13 ☐	Q9Y3U8	NA	RPL36	ribosomal protein L36	25873	ENSG00000130255
14 ☐	P49207	NA	RPL34	ribosomal protein L34	6164	ENSG00000109475

Database:biological process	Name:cellular nitrogen compound catabolic process ID:GO:0044270					
C=793; O=14; E=2.69; R=5.20; rawP=2.30e-07; adjP=5.28e-06						
Index	UserID	Value	Gene Symbol	Gene Name	EntrezGene	Ensembl
1 ☐	P39023	NA	RPL3	ribosomal protein L3	6122	ENSG00000100316
2 ☐	P09429	NA	HMGB1	high mobility group box 1	3146	ENSG00000189403
3 ☐	P61106	NA	RAB14	RAB14, member RAS oncogene family	51552	ENSG00000119396
4 ☐	P42766	NA	RPL35	ribosomal protein L35	11224	ENSG00000136942
5 ☐	P27635	NA	RPL10	ribosomal protein L10	6134	ENSG00000147403
6 ☐	P40616	NA	ARL1	ADP-ribosylation factor-like 1	400	ENSG00000120805
7 ☐	P61019	NA	RAB2A	RAB2A, member RAS oncogene family	5862	ENSG00000104388
8 ☐	Q02543	NA	RPL18A	ribosomal protein L18a	6142	ENSG00000105640

Database:biological process Name:cellular nitrogen compound catabolic process ID:GO:0044270						
C=793; O=14; E=2.69; R=5.20; rawP=2.30e-07; adjP=5.28e-06						
Index	UserID	Value	Gene Symbol	Gene Name	EntrezGene	Ensembl
9 ☐	P84098	NA	RPL19	ribosomal protein L19	6143	ENSG00000108298
10 ☐	P09493	NA	TPM1	tropomyosin 1 (alpha)	7168	ENSG00000140416
11 ☐	P40429	NA	RPL13A	ribosomal protein L13a	23521	ENSG00000142541
12 ☐	Q13126	NA	MTAP	methylthioadenosine phosphorylase	4507	ENSG00000099810
13 ☐	Q9Y3U8	NA	RPL36	ribosomal protein L36	25873	ENSG00000130255
14 ☐	P49207	NA	RPL34	ribosomal protein L34	6164	ENSG00000109475

Database:biological process Name:intracellular protein transport ID:GO:0006886						
C=691; O=13; E=2.35; R=5.54; rawP=3.33e-07; adjP=7.25e-06						
Index	UserID	Value	Gene Symbol	Gene Name	EntrezGene	Ensembl
1 ☐	P39023	NA	RPL3	ribosomal protein L3	6122	ENSG00000100316
2 ☐	P42766	NA	RPL35	ribosomal protein L35	11224	ENSG00000136942
3 ☐	P27635	NA	RPL10	ribosomal protein L10	6134	ENSG00000147403
4 ☐	Q9HAV7	NA	GRPEL1	GrpE-like 1, mitochondrial (E. coli)	80273	ENSG00000109519
5 ☐	P60059	NA	SEC61G	Sec61 gamma subunit	23480	ENSG00000132432
6 ☐	P06748	NA	NPM1	nucleophosmin (nucleolar phosphoprotein B23, numatrin)	4869	ENSG00000181163
7 ☐	Q02543	NA	RPL18A	ribosomal protein L18a	6142	ENSG00000105640
8 ☐	P84098	NA	RPL19	ribosomal protein L19	6143	ENSG00000108298
9 ☐	O43747	NA	AP1G1	adaptor-related protein complex 1, gamma 1 subunit	164	ENSG00000166747
10 ☐	P30040	NA	ERP29	endoplasmic reticulum protein 29	10961	ENSG00000089248

Index	UserID	Value	Gene Symbol	Gene Name	EntrezGene	Ensembl
11 ☐	P40429	NA	RPL13A	ribosomal protein L13a	23521	ENSG00000142541
12 ☐	Q9Y3U8	NA	RPL36	ribosomal protein L36	25873	ENSG00000130255
13 ☐	P49207	NA	RPL34	ribosomal protein L34	6164	ENSG00000109475

Database:biological process Name:organic cyclic compound catabolic process ID:GO:1901361

C=822; O=14; E=2.79; R=5.01; rawP=3.56e-07; adjP=7.45e-06

Index	UserID	Value	Gene Symbol	Gene Name	EntrezGene	Ensembl
1 ☐	P39023	NA	RPL3	ribosomal protein L3	6122	ENSG00000100316
2 ☐	P09429	NA	HMGB1	high mobility group box 1	3146	ENSG00000189403
3 ☐	P61106	NA	RAB14	RAB14, member RAS oncogene family	51552	ENSG00000119396
4 ☐	P42766	NA	RPL35	ribosomal protein L35	11224	ENSG00000136942
5 ☐	P27635	NA	RPL10	ribosomal protein L10	6134	ENSG00000147403
6 ☐	P40616	NA	ARL1	ADP-ribosylation factor-like 1	400	ENSG00000120805
7 ☐	P61019	NA	RAB2A	RAB2A, member RAS oncogene family	5862	ENSG00000104388
8 ☐	Q02543	NA	RPL18A	ribosomal protein L18a	6142	ENSG00000105640
9 ☐	P84098	NA	RPL19	ribosomal protein L19	6143	ENSG00000108298
10 ☐	P09493	NA	TPM1	tropomyosin 1 (alpha)	7168	ENSG00000140416
11 ☐	P40429	NA	RPL13A	ribosomal protein L13a	23521	ENSG00000142541
12 ☐	Q13126	NA	MTAP	methylthioadenosine phosphorylase	4507	ENSG00000099810
13 ☐	Q9Y3U8	NA	RPL36	ribosomal protein L36	25873	ENSG00000130255
14 ☐	P49207	NA	RPL34	ribosomal protein L34	6164	ENSG00000109475

Database:biological process Name:RNA catabolic process ID:GO:0006401						
C=210; O=8; E=0.71; R=11.21; rawP=4.78e-07; adjP=9.29e-06						
Index	UserID	Value	Gene Symbol	Gene Name	EntrezGene	Ensembl
1 ☐	P39023	NA	RPL3	ribosomal protein L3	6122	ENSG00000100316
2 ☐	P42766	NA	RPL35	ribosomal protein L35	11224	ENSG00000136942
3 ☐	P27635	NA	RPL10	ribosomal protein L10	6134	ENSG00000147403
4 ☐	P84098	NA	RPL19	ribosomal protein L19	6143	ENSG00000108298
5 ☐	Q02543	NA	RPL18A	ribosomal protein L18a	6142	ENSG00000105640
6 ☐	P40429	NA	RPL13A	ribosomal protein L13a	23521	ENSG00000142541
7 ☐	P49207	NA	RPL34	ribosomal protein L34	6164	ENSG00000109475
8 ☐	Q9Y3U8	NA	RPL36	ribosomal protein L36	25873	ENSG00000130255

Database:biological process Name:cellular component disassembly at cellular level ID:GO:0071845						
C=289; O=9; E=0.98; R=9.17; rawP=4.68e-07; adjP=9.29e-06						
Index	UserID	Value	Gene Symbol	Gene Name	EntrezGene	Ensembl
1 ☐	P39023	NA	RPL3	ribosomal protein L3	6122	ENSG00000100316
2 ☐	P09429	NA	HMGB1	high mobility group box 1	3146	ENSG00000189403
3 ☐	P42766	NA	RPL35	ribosomal protein L35	11224	ENSG00000136942
4 ☐	P27635	NA	RPL10	ribosomal protein L10	6134	ENSG00000147403
5 ☐	P84098	NA	RPL19	ribosomal protein L19	6143	ENSG00000108298
6 ☐	Q02543	NA	RPL18A	ribosomal protein L18a	6142	ENSG00000105640
7 ☐	P40429	NA	RPL13A	ribosomal protein L13a	23521	ENSG00000142541
8 ☐	Q9Y3U8	NA	RPL36	ribosomal protein L36	25873	ENSG00000130255
9 ☐	P49207	NA	RPL34	ribosomal protein L34	6164	ENSG00000109475

Database:biological process disassembly	Name:cellular component	ID:GO:0022411				
C=293; O=9; E=1.00; R=9.04; rawP=5.25e-07; adjP=9.85e-06						
Index	UserID	Value	Gene Symbol	Gene Name	EntrezGene	Ensembl
1 ☐	P39023	NA	RPL3	ribosomal protein L3	6122	ENSG00000100316
2 ☐	P09429	NA	HMGB1	high mobility group box 1	3146	ENSG00000189403
3 ☐	P42766	NA	RPL35	ribosomal protein L35	11224	ENSG00000136942
4 ☐	P27635	NA	RPL10	ribosomal protein L10	6134	ENSG00000147403
5 ☐	P84098	NA	RPL19	ribosomal protein L19	6143	ENSG00000108298
6 ☐	Q02543	NA	RPL18A	ribosomal protein L18a	6142	ENSG00000105640
7 ☐	P40429	NA	RPL13A	ribosomal protein L13a	23521	ENSG00000142541
8 ☐	Q9Y3U8	NA	RPL36	ribosomal protein L36	25873	ENSG00000130255
9 ☐	P49207	NA	RPL34	ribosomal protein L34	6164	ENSG00000109475

Database:biological process	Name:catabolic process	ID:GO:0009056				
C=1991; O=21; E=6.76; R=3.10; rawP=6.29e-07; adjP=1.14e-05						
Index	UserID	Value	Gene Symbol	Gene Name	EntrezGene	Ensembl
1 ☐	P39023	NA	RPL3	ribosomal protein L3	6122	ENSG00000100316
2 ☐	P09429	NA	HMGB1	high mobility group box 1	3146	ENSG00000189403
3 ☐	P61106	NA	RAB14	RAB14, member RAS oncogene family	51552	ENSG00000119396
4 ☐	P42766	NA	RPL35	ribosomal protein L35	11224	ENSG00000136942
5 ☐	P30041	NA	PRDX6	peroxiredoxin 6	9588	ENSG00000117592
6 ☐	P68036	NA	UBE2L3	ubiquitin-conjugating enzyme E2L 3	7332	ENSG00000185651
7 ☐	P61019	NA	RAB2A	RAB2A, member RAS oncogene family	5862	ENSG00000104388
8 ☐	Q02543	NA	RPL18A	ribosomal protein L18a	6142	ENSG00000105640
9 ☐	Q99497	NA	PARK7	parkinson protein 7	11315	ENSG00000116288
10 ☐	P40429	NA	RPL13A		23521	ENSG00000142541

Database:biological process Name:catabolic process ID:GO:0009056						
C=1991; O=21; E=6.76; R=3.10; rawP=6.29e-07; adjP=1.14e-05						
Index	UserID	Value	Gene Symbol	Gene Name	EntrezGene	Ensembl
				ribosomal protein L13a		
11 ☐	Q13126	NA	MTAP	methylthioadenosine phosphorylase	4507	ENSG00000099810
12 ☐	Q9Y3U8	NA	RPL36	ribosomal protein L36	25873	ENSG00000130255
13 ☐	P49207	NA	RPL34	ribosomal protein L34	6164	ENSG00000109475
14 ☐	P62942	NA	FKBP1A	FK506 binding protein 1A, 12kDa	2280	ENSG00000088832
15 ☐	P27635	NA	RPL10	ribosomal protein L10	6134	ENSG00000147403
16 ☐	P00558	NA	PGK1	phosphoglycerate kinase 1	5230	ENSG00000102144
17 ☐	P09972	NA	ALDOC	aldolase C, fructose-bisphosphate	230	ENSG00000109107
18 ☐	P40616	NA	ARL1	ADP-ribosylation factor-like 1	400	ENSG00000120805
19 ☐	O75390	NA	CS	citrate synthase	1431	ENSG00000062485
20 ☐	P84098	NA	RPL19	ribosomal protein L19	6143	ENSG00000108298
21 ☐	P09493	NA	TPM1	tropomyosin 1 (alpha)	7168	ENSG00000140416

Database:biological process Name:protein localization to organelle ID:GO:0033365						
C=499; O=11; E=1.70; R=6.49; rawP=6.75e-07; adjP=1.18e-05						
Index	UserID	Value	Gene Symbol	Gene Name	EntrezGene	Ensembl
1 ☐	P39023	NA	RPL3	ribosomal protein L3	6122	ENSG00000100316
2 ☐	P42766	NA	RPL35	ribosomal protein L35	11224	ENSG00000136942
3 ☐	P27635	NA	RPL10	ribosomal protein L10	6134	ENSG00000147403
4 ☐	Q9HAV7	NA	GRPEL1	GrpE-like 1, mitochondrial (E. coli)	80273	ENSG00000109519
5 ☐	P60059	NA	SEC61G	Sec61 gamma subunit	23480	ENSG00000132432
6 ☐	P40616	NA	ARL1	ADP-ribosylation factor-like 1	400	ENSG00000120805
7 ☐	Q02543	NA	RPL18A	ribosomal protein L18a	6142	ENSG00000105640

Database:biological process Name:protein localization to organelle ID:GO:0033365						
C=499; O=11; E=1.70; R=6.49; rawP=6.75e-07; adjP=1.18e-05						
Index	UserID	Value	Gene Symbol	Gene Name	EntrezGene	Ensembl
8 ☐	P84098	NA	RPL19	ribosomal protein L19	6143	ENSG00000108298
9 ☐	P40429	NA	RPL13A	ribosomal protein L13a	23521	ENSG00000142541
10 ☐	Q9Y3U8	NA	RPL36	ribosomal protein L36	25873	ENSG00000130255
11 ☐	P49207	NA	RPL34	ribosomal protein L34	6164	ENSG00000109475

Database:biological process Name:viral infectious cycle ID:GO:0019058						
C=221; O=8; E=0.75; R=10.65; rawP=7.04e-07; adjP=1.20e-05						
Index	UserID	Value	Gene Symbol	Gene Name	EntrezGene	Ensembl
1 ☐	P39023	NA	RPL3	ribosomal protein L3	6122	ENSG00000100316
2 ☐	P42766	NA	RPL35	ribosomal protein L35	11224	ENSG00000136942
3 ☐	P27635	NA	RPL10	ribosomal protein L10	6134	ENSG00000147403
4 ☐	P84098	NA	RPL19	ribosomal protein L19	6143	ENSG00000108298
5 ☐	Q02543	NA	RPL18A	ribosomal protein L18a	6142	ENSG00000105640
6 ☐	P40429	NA	RPL13A	ribosomal protein L13a	23521	ENSG00000142541
7 ☐	P49207	NA	RPL34	ribosomal protein L34	6164	ENSG00000109475
8 ☐	Q9Y3U8	NA	RPL36	ribosomal protein L36	25873	ENSG00000130255

Database:biological process Name:organic substance catabolic process ID:GO:1901575						
C=1851; O=20; E=6.29; R=3.18; rawP=9.15e-07; adjP=1.51e-05						
Index	UserID	Value	Gene Symbol	Gene Name	EntrezGene	Ensembl
1 ☐	P39023	NA	RPL3	ribosomal protein L3	6122	ENSG00000100316
2 ☐	P09429	NA	HMGB1	high mobility group box 1	3146	ENSG00000189403
3 ☐	P61106	NA	RAB14	RAB14, member RAS oncogene family	51552	ENSG00000119396
4 ☐	P42766	NA	RPL35		11224	ENSG00000136942

Database:biological process Name:organic substance catabolic process ID:GO:1901575						
C=1851; O=20; E=6.29; R=3.18; rawP=9.15e-07; adjP=1.51e-05						
Index	UserID	Value	Gene Symbol	Gene Name	EntrezGene	Ensembl
				ribosomal protein L35		
5 ☐	P30041	NA	PRDX6	peroxiredoxin 6	9588	ENSG00000117592
6 ☐	P68036	NA	UBE2L3	ubiquitin-conjugating enzyme E2L 3	7332	ENSG00000185651
7 ☐	P61019	NA	RAB2A	RAB2A, member RAS oncogene family	5862	ENSG00000104388
8 ☐	Q02543	NA	RPL18A	ribosomal protein L18a	6142	ENSG00000105640
9 ☐	P40429	NA	RPL13A	ribosomal protein L13a	23521	ENSG00000142541
10 ☐	Q13126	NA	MTAP	methylthioadenosine phosphorylase	4507	ENSG00000099810
11 ☐	Q9Y3U8	NA	RPL36	ribosomal protein L36	25873	ENSG00000130255
12 ☐	P49207	NA	RPL34	ribosomal protein L34	6164	ENSG00000109475
13 ☐	P62942	NA	FKBP1A	FK506 binding protein 1A, 12kDa	2280	ENSG00000088832
14 ☐	P27635	NA	RPL10	ribosomal protein L10	6134	ENSG00000147403
15 ☐	P00558	NA	PGK1	phosphoglycerate kinase 1	5230	ENSG00000102144
16 ☐	P09972	NA	ALDOC	aldolase C, fructose-bisphosphate	230	ENSG00000109107
17 ☐	P40616	NA	ARL1	ADP-ribosylation factor-like 1	400	ENSG00000120805
18 ☐	O75390	NA	CS	citrate synthase	1431	ENSG00000062485
19 ☐	P84098	NA	RPL19	ribosomal protein L19	6143	ENSG00000108298
20 ☐	P09493	NA	TPM1	tropomyosin 1 (alpha)	7168	ENSG00000140416

Database:biological process Name:cellular protein localization ID:GO:0034613						
C=931; O=14; E=3.16; R=4.43; rawP=1.59e-06; adjP=2.50e-05						
Index	UserID	Value	Gene Symbol	Gene Name	EntrezGene	Ensembl
1 ☐	P39023	NA	RPL3	ribosomal protein L3	6122	ENSG00000100316
2 ☐	P42766	NA	RPL35	ribosomal protein L35	11224	ENSG00000136942

Database:biological process Name:cellular protein localization ID:GO:0034613						
C=931; O=14; E=3.16; R=4.43; rawP=1.59e-06; adjP=2.50e-05						
Index	UserID	Value	Gene Symbol	Gene Name	EntrezGene	Ensembl
3 ☐	P27635	NA	RPL10	ribosomal protein L10	6134	ENSG00000147403
4 ☐	Q9HAV7	NA	GRPEL1	GrpE-like 1, mitochondrial (E. coli)	80273	ENSG00000109519
5 ☐	P60059	NA	SEC61G	Sec61 gamma subunit	23480	ENSG00000132432
6 ☐	P40616	NA	ARL1	ADP-ribosylation factor-like 1	400	ENSG00000120805
7 ☐	P06748	NA	NPM1	nucleophosmin (nucleolar phosphoprotein B23, numatrin)	4869	ENSG00000181163
8 ☐	Q02543	NA	RPL18A	ribosomal protein L18a	6142	ENSG00000105640
9 ☐	P84098	NA	RPL19	ribosomal protein L19	6143	ENSG00000108298
10 ☐	O43747	NA	AP1G1	adaptor-related protein complex 1, gamma 1 subunit	164	ENSG00000166747
11 ☐	P30040	NA	ERP29	endoplasmic reticulum protein 29	10961	ENSG00000089248
12 ☐	P40429	NA	RPL13A	ribosomal protein L13a	23521	ENSG00000142541
13 ☐	Q9Y3U8	NA	RPL36	ribosomal protein L36	25873	ENSG00000130255
14 ☐	P49207	NA	RPL34	ribosomal protein L34	6164	ENSG00000109475

Database:biological process Name:protein transport ID:GO:0015031						
C=1231; O=16; E=4.18; R=3.83; rawP=1.61e-06; adjP=2.50e-05						
Index	UserID	Value	Gene Symbol	Gene Name	EntrezGene	Ensembl
1 ☐	P39023	NA	RPL3	ribosomal protein L3	6122	ENSG00000100316
2 ☐	P61106	NA	RAB14	RAB14, member RAS oncogene family	51552	ENSG00000119396
3 ☐	P42766	NA	RPL35	ribosomal protein L35	11224	ENSG00000136942
4 ☐	Q9HAV7	NA	GRPEL1	GrpE-like 1, mitochondrial (E. coli)	80273	ENSG00000109519

Database:biological process Name:protein transport ID:GO:0015031						
C=1231; O=16; E=4.18; R=3.83; rawP=1.61e-06; adjP=2.50e-05						
Index	UserID	Value	Gene Symbol	Gene Name	EntrezGene	Ensembl
5 ☐	P60059	NA	SEC61G	Sec61 gamma subunit	23480	ENSG00000132432
6 ☐	P06748	NA	NPM1	nucleophosmin (nucleolar phosphoprotein B23, numatrin)	4869	ENSG00000181163
7 ☐	P61019	NA	RAB2A	RAB2A, member RAS oncogene family	5862	ENSG00000104388
8 ☐	Q02543	NA	RPL18A	ribosomal protein L18a	6142	ENSG00000105640
9 ☐	P30040	NA	ERP29	endoplasmic reticulum protein 29	10961	ENSG00000089248
10 ☐	P40429	NA	RPL13A	ribosomal protein L13a	23521	ENSG00000142541
11 ☐	Q9Y3U8	NA	RPL36	ribosomal protein L36	25873	ENSG00000130255
12 ☐	P49207	NA	RPL34	ribosomal protein L34	6164	ENSG00000109475
13 ☐	P27635	NA	RPL10	ribosomal protein L10	6134	ENSG00000147403
14 ☐	Q9H0U4	NA	RAB1B	RAB1B, member RAS oncogene family	81876	ENSG00000174903
15 ☐	P84098	NA	RPL19	ribosomal protein L19	6143	ENSG00000108298
16 ☐	O43747	NA	AP1G1	adaptor-related protein complex 1, gamma 1 subunit	164	ENSG00000166747

Database:biological process Name:cellular macromolecule localization ID:GO:0070727						
C=935; O=14; E=3.18; R=4.41; rawP=1.67e-06; adjP=2.52e-05						
Index	UserID	Value	Gene Symbol	Gene Name	EntrezGene	Ensembl
1 ☐	P39023	NA	RPL3	ribosomal protein L3	6122	ENSG00000100316
2 ☐	P42766	NA	RPL35	ribosomal protein L35	11224	ENSG00000136942
3 ☐	P27635	NA	RPL10	ribosomal protein L10	6134	ENSG00000147403
4 ☐	Q9HAV7	NA	GRPEL1	GrpE-like 1, mitochondrial (E. coli)	80273	ENSG00000109519

Database:biological process localization			Name:cellular macromolecule ID:GO:0070727			
C=935; O=14; E=3.18; R=4.41; rawP=1.67e-06; adjP=2.52e-05						
Index	UserID	Value	Gene Symbol	Gene Name	EntrezGene	Ensembl
5 ☐	P60059	NA	SEC61G	Sec61 gamma subunit	23480	ENSG00000132432
6 ☐	P40616	NA	ARL1	ADP-ribosylation factor-like 1	400	ENSG00000120805
7 ☐	P06748	NA	NPM1	nucleophosmin (nucleolar phosphoprotein B23, numatrin)	4869	ENSG00000181163
8 ☐	Q02543	NA	RPL18A	ribosomal protein L18a	6142	ENSG00000105640
9 ☐	P84098	NA	RPL19	ribosomal protein L19	6143	ENSG00000108298
10 ☐	O43747	NA	AP1G1	adaptor-related protein complex 1, gamma 1 subunit	164	ENSG00000166747
11 ☐	P30040	NA	ERP29	endoplasmic reticulum protein 29	10961	ENSG00000089248
12 ☐	P40429	NA	RPL13A	ribosomal protein L13a	23521	ENSG00000142541
13 ☐	Q9Y3U8	NA	RPL36	ribosomal protein L36	25873	ENSG00000130255
14 ☐	P49207	NA	RPL34	ribosomal protein L34	6164	ENSG00000109475

Database:biological process localization			Name:establishment of protein ID:GO:0045184			
C=1269; O=16; E=4.31; R=3.71; rawP=2.40e-06; adjP=3.53e-05						
Index	UserID	Value	Gene Symbol	Gene Name	EntrezGene	Ensembl
1 ☐	P39023	NA	RPL3	ribosomal protein L3	6122	ENSG00000100316
2 ☐	P61106	NA	RAB14	RAB14, member RAS oncogene family	51552	ENSG00000119396
3 ☐	P42766	NA	RPL35	ribosomal protein L35	11224	ENSG00000136942
4 ☐	Q9HAV7	NA	GRPEL1	GrpE-like 1, mitochondrial (E. coli)	80273	ENSG00000109519
5 ☐	P60059	NA	SEC61G	Sec61 gamma subunit	23480	ENSG00000132432
6 ☐	P06748	NA	NPM1	nucleophosmin (nucleolar	4869	ENSG00000181163

Database:biological process				Name:establishment of protein localization ID:GO:0045184		
C=1269; O=16; E=4.31; R=3.71; rawP=2.40e-06; adjP=3.53e-05						
Index	UserID	Value	Gene Symbol	Gene Name	EntrezGene	Ensembl
				phosphoprotein B23, numatrin)		
7 ☐	P61019	NA	RAB2A	RAB2A, member RAS oncogene family	5862	ENSG00000104388
8 ☐	Q02543	NA	RPL18A	ribosomal protein L18a	6142	ENSG00000105640
9 ☐	P30040	NA	ERP29	endoplasmic reticulum protein 29	10961	ENSG00000089248
10 ☐	P40429	NA	RPL13A	ribosomal protein L13a	23521	ENSG00000142541
11 ☐	Q9Y3U8	NA	RPL36	ribosomal protein L36	25873	ENSG00000130255
12 ☐	P49207	NA	RPL34	ribosomal protein L34	6164	ENSG00000109475
13 ☐	P27635	NA	RPL10	ribosomal protein L10	6134	ENSG00000147403
14 ☐	Q9H0U4	NA	RAB1B	RAB1B, member RAS oncogene family	81876	ENSG00000174903
15 ☐	P84098	NA	RPL19	ribosomal protein L19	6143	ENSG00000108298
16 ☐	O43747	NA	AP1G1	adaptor-related protein complex 1, gamma 1 subunit	164	ENSG00000166747

Database:biological process				Name:protein targeting	ID:GO:0006605	
C=469; O=10; E=1.59; R=6.28; rawP=3.16e-06; adjP=4.52e-05						
Index	UserID	Value	Gene Symbol	Gene Name	EntrezGene	Ensembl
1 ☐	P39023	NA	RPL3	ribosomal protein L3	6122	ENSG00000100316
2 ☐	P42766	NA	RPL35	ribosomal protein L35	11224	ENSG00000136942
3 ☐	P27635	NA	RPL10	ribosomal protein L10	6134	ENSG00000147403
4 ☐	Q9HAV7	NA	GRPEL1	GrpE-like 1, mitochondrial (E. coli)	80273	ENSG00000109519
5 ☐	P60059	NA	SEC61G	Sec61 gamma subunit	23480	ENSG00000132432
6 ☐	Q02543	NA	RPL18A	ribosomal protein L18a	6142	ENSG00000105640

Database:biological process			Name:protein targeting		ID:GO:0006605	
C=469; O=10; E=1.59; R=6.28; rawP=3.16e-06; adjP=4.52e-05						
Index	UserID	Value	Gene Symbol	Gene Name	EntrezGene	Ensembl
7 ☐	P84098	NA	RPL19	ribosomal protein L19	6143	ENSG00000108298
8 ☐	P40429	NA	RPL13A	ribosomal protein L13a	23521	ENSG00000142541
9 ☐	Q9Y3U8	NA	RPL36	ribosomal protein L36	25873	ENSG00000130255
10 ☐	P49207	NA	RPL34	ribosomal protein L34	6164	ENSG00000109475

Database:biological process			Name:cellular catabolic process		ID:GO:0044248	
C=1665; O=18; E=5.66; R=3.18; rawP=4.12e-06; adjP=5.75e-05						
Index	UserID	Value	Gene Symbol	Gene Name	EntrezGene	Ensembl
1 ☐	P39023	NA	RPL3	ribosomal protein L3	6122	ENSG00000100316
2 ☐	P09429	NA	HMGB1	high mobility group box 1	3146	ENSG00000189403
3 ☐	P61106	NA	RAB14	RAB14, member RAS oncogene family	51552	ENSG00000119396
4 ☐	P42766	NA	RPL35	ribosomal protein L35	11224	ENSG00000136942
5 ☐	P30041	NA	PRDX6	peroxiredoxin 6	9588	ENSG00000117592
6 ☐	P68036	NA	UBE2L3	ubiquitin-conjugating enzyme E2L 3	7332	ENSG00000185651
7 ☐	P61019	NA	RAB2A	RAB2A, member RAS oncogene family	5862	ENSG00000104388
8 ☐	Q02543	NA	RPL18A	ribosomal protein L18a	6142	ENSG00000105640
9 ☐	Q99497	NA	PARK7	parkinson protein 7	11315	ENSG00000116288
10 ☐	P40429	NA	RPL13A	ribosomal protein L13a	23521	ENSG00000142541
11 ☐	Q13126	NA	MTAP	methylthioadenosine phosphorylase	4507	ENSG00000099810
12 ☐	Q9Y3U8	NA	RPL36	ribosomal protein L36	25873	ENSG00000130255
13 ☐	P49207	NA	RPL34	ribosomal protein L34	6164	ENSG00000109475
14 ☐	P27635	NA	RPL10	ribosomal protein L10	6134	ENSG00000147403
15 ☐	P40616	NA	ARL1	ADP-ribosylation factor-like 1	400	ENSG00000120805

Database:biological process			Name:cellular catabolic process		ID:GO:0044248	
C=1665; O=18; E=5.66; R=3.18; rawP=4.12e-06; adjP=5.75e-05						
Index	UserID	Value	Gene Symbol	Gene Name	EntrezGene	Ensembl
16 ☐	O75390	NA	CS	citrate synthase	1431	ENSG00000062485
17 ☐	P84098	NA	RPL19	ribosomal protein L19	6143	ENSG00000108298
18 ☐	P09493	NA	TPM1	tropomyosin 1 (alpha)	7168	ENSG00000140416

Database:biological process			Name:protein localization		ID:GO:0008104	
C=1559; O=17; E=5.30; R=3.21; rawP=7.60e-06; adjP=0.0001						
Index	UserID	Value	Gene Symbol	Gene Name	EntrezGene	Ensembl
1 ☐	P39023	NA	RPL3	ribosomal protein L3	6122	ENSG00000100316
2 ☐	P61106	NA	RAB14	RAB14, member RAS oncogene family	51552	ENSG00000119396
3 ☐	P42766	NA	RPL35	ribosomal protein L35	11224	ENSG00000136942
4 ☐	Q9HAV7	NA	GRPEL1	GrpE-like 1, mitochondrial (E. coli)	80273	ENSG00000109519
5 ☐	P60059	NA	SEC61G	Sec61 gamma subunit	23480	ENSG00000132432
6 ☐	P06748	NA	NPM1	nucleophosmin (nucleolar phosphoprotein B23, numatrin)	4869	ENSG00000181163
7 ☐	P61019	NA	RAB2A	RAB2A, member RAS oncogene family	5862	ENSG00000104388
8 ☐	Q02543	NA	RPL18A	ribosomal protein L18a	6142	ENSG00000105640
9 ☐	P30040	NA	ERP29	endoplasmic reticulum protein 29	10961	ENSG00000089248
10 ☐	P40429	NA	RPL13A	ribosomal protein L13a	23521	ENSG00000142541
11 ☐	Q9Y3U8	NA	RPL36	ribosomal protein L36	25873	ENSG00000130255
12 ☐	P49207	NA	RPL34	ribosomal protein L34	6164	ENSG00000109475
13 ☐	P27635	NA	RPL10	ribosomal protein L10	6134	ENSG00000147403
14 ☐	P40616	NA	ARL1	ADP-ribosylation factor-like 1	400	ENSG00000120805
15 ☐	Q9H0U4	NA	RAB1B		81876	ENSG00000174903

Database:biological process Name:protein localization ID:GO:0008104						
C=1559; O=17; E=5.30; R=3.21; rawP=7.60e-06; adjP=0.0001						
Index	UserID	Value	Gene Symbol	Gene Name	EntrezGene	Ensembl
				RAB1B, member RAS oncogene family		
16 ☐	P84098	NA	RPL19	ribosomal protein L19	6143	ENSG00000108298
17 ☐	O43747	NA	AP1G1	adaptor-related protein complex 1, gamma 1 subunit	164	ENSG00000166747

Database:molecular function Name:structural constituent of ribosome ID:GO:0003735						
C=152; O=8; E=0.51; R=15.77; rawP=3.50e-08; adjP=3.29e-06						
Index	UserID	Value	Gene Symbol	Gene Name	EntrezGene	Ensembl
1 ☐	P39023	NA	RPL3	ribosomal protein L3	6122	ENSG00000100316
2 ☐	P42766	NA	RPL35	ribosomal protein L35	11224	ENSG00000136942
3 ☐	P27635	NA	RPL10	ribosomal protein L10	6134	ENSG00000147403
4 ☐	P84098	NA	RPL19	ribosomal protein L19	6143	ENSG00000108298
5 ☐	Q02543	NA	RPL18A	ribosomal protein L18a	6142	ENSG00000105640
6 ☐	P40429	NA	RPL13A	ribosomal protein L13a	23521	ENSG00000142541
7 ☐	P49207	NA	RPL34	ribosomal protein L34	6164	ENSG00000109475
8 ☐	Q9Y3U8	NA	RPL36	ribosomal protein L36	25873	ENSG00000130255

Database:molecular function Name:RNA binding ID:GO:0003723						
C=843; O=14; E=2.81; R=4.97; rawP=4.04e-07; adjP=1.90e-05						
Index	UserID	Value	Gene Symbol	Gene Name	EntrezGene	Ensembl
1 ☐	P39023	NA	RPL3	ribosomal protein L3	6122	ENSG00000100316
2 ☐	P42766	NA	RPL35	ribosomal protein L35	11224	ENSG00000136942
3 ☐	Q01130	NA	SRSF2	serine/arginine-rich splicing factor 2	6427	ENSG00000161547
4 ☐	P55795	NA	HNRNPH2	heterogeneous nuclear	3188	ENSG00000126945

Database:molecular function			Name:RNA binding		ID:GO:0003723	
C=843; O=14; E=2.81; R=4.97; rawP=4.04e-07; adjP=1.90e-05						
Index	UserID	Value	Gene Symbol	Gene Name	EntrezGene	Ensembl
				ribonucleoprotein H2 (H')		
5 ☐	Q15020	NA	SART3	squamous cell carcinoma antigen recognized by T cells 3	9733	ENSG00000075856
6 ☐	P41567	NA	EIF1	eukaryotic translation initiation factor 1	10209	ENSG00000173812
7 ☐	P06748	NA	NPM1	nucleophosmin (nucleolar phosphoprotein B23, numatrin)	4869	ENSG00000181163
8 ☐	Q02543	NA	RPL18A	ribosomal protein L18a	6142	ENSG00000105640
9 ☐	P84098	NA	RPL19	ribosomal protein L19	6143	ENSG00000108298
10 ☐	P16989	NA	CSDA	cold shock domain protein A	8531	ENSG00000060138
11 ☐	P49588	NA	AARS	alanyl-tRNA synthetase	16	ENSG00000090861
12 ☐	Q99497	NA	PARK7	parkinson protein 7	11315	ENSG00000116288
13 ☐	Q96AE4	NA	FUBP1	far upstream element (FUSE) binding protein 1	8880	ENSG00000162613
14 ☐	P49207	NA	RPL34	ribosomal protein L34	6164	ENSG00000109475

Database:molecular function			Name:structural molecule activity		ID:GO:0005198	
C=608; O=10; E=2.03; R=4.93; rawP=2.67e-05; adjP=0.0008						
Index	UserID	Value	Gene Symbol	Gene Name	EntrezGene	Ensembl
1 ☐	P39023	NA	RPL3	ribosomal protein L3	6122	ENSG00000100316
2 ☐	P42766	NA	RPL35	ribosomal protein L35	11224	ENSG00000136942
3 ☐	P27635	NA	RPL10	ribosomal protein L10	6134	ENSG00000147403
4 ☐	P63261	NA	ACTG1	actin, gamma 1	71	ENSG00000184009
5 ☐	P84098	NA	RPL19	ribosomal protein L19	6143	ENSG00000108298
6 ☐	Q02543	NA	RPL18A	ribosomal protein L18a	6142	ENSG00000105640

Database:molecular function Name:structural molecule activity ID:GO:0005198						
C=608; O=10; E=2.03; R=4.93; rawP=2.67e-05; adjP=0.0008						
Index	UserID	Value	Gene Symbol	Gene Name	EntrezGene	Ensembl
7 ☐	P09493	NA	TPM1	tropomyosin 1 (alpha)	7168	ENSG00000140416
8 ☐	P40429	NA	RPL13A	ribosomal protein L13a	23521	ENSG00000142541
9 ☐	Q9Y3U8	NA	RPL36	ribosomal protein L36	25873	ENSG00000130255
10 ☐	P49207	NA	RPL34	ribosomal protein L34	6164	ENSG00000109475

Database:molecular function Name: ID:GO:0003697						
C=61; O=4; E=0.20; R=19.64; rawP=4.99e-05; adjP=0.0012						
Index	UserID	Value	Gene Symbol	Gene Name	EntrezGene	Ensembl
1 ☐	P09429	NA	HMGB1	high mobility group box 1	3146	ENSG00000189403
2 ☐	P16989	NA	CSDA	cold shock domain protein A	8531	ENSG00000060138
3 ☐	Q00577	NA	PURA	purine-rich element binding protein A	5813	ENSG00000185129
4 ☐	Q96AE4	NA	FUBP1	far upstream element (FUSE) binding protein 1	8880	ENSG00000162613

Database:molecular function Name:transferase activity, transferring acyl groups, acyl groups converted into alkyl on transfer ID:GO:0046912						
C=5; O=2; E=0.02; R=119.82; rawP=0.0001; adjP=0.0019						
Index	UserID	Value	Gene Symbol	Gene Name	EntrezGene	Ensembl
1 ☐	Q01581	NA	HMGCS1	3-hydroxy-3-methylglutaryl-CoA synthase 1 (soluble)	3157	ENSG00000112972
2 ☐	O75390	NA	CS	citrate synthase	1431	ENSG00000062485

Database:molecular function Name:peroxiredoxin activity ID:GO:0051920						
C=7; O=2; E=0.02; R=85.58; rawP=0.0002; adjP=0.0031						
Index	UserID	Value	Gene Symbol	Gene Name	EntrezGene	Ensembl
1 ☐	P30041	NA	PRDX6	peroxiredoxin 6	9588	ENSG00000117592
2 ☐	Q99497	NA	PARK7	parkinson protein 7	11315	ENSG00000116288

Database:molecular function			Name:heterocyclic compound binding			ID:GO:1901363
C=5290; O=30; E=17.66; R=1.70; rawP=0.0003; adjP=0.0040						
Index	UserID	Value	Gene Symbol	Gene Name	EntrezGene	Ensembl
1 ☐	P39023	NA	RPL3	ribosomal protein L3	6122	ENSG00000100316
2 ☐	P09429	NA	HMGB1	high mobility group box 1	3146	ENSG00000189403
3 ☐	P61106	NA	RAB14	RAB14, member RAS oncogene family	51552	ENSG00000119396
4 ☐	P42766	NA	RPL35	ribosomal protein L35	11224	ENSG00000136942
5 ☐	P63261	NA	ACTG1	actin, gamma 1	71	ENSG00000184009
6 ☐	P22314	NA	UBA1	ubiquitin-like modifier activating enzyme 1	7317	ENSG00000130985
7 ☐	Q9HAV7	NA	GRPEL1	GrpE-like 1, mitochondrial (E. coli)	80273	ENSG00000109519
8 ☐	Q15020	NA	SART3	squamous cell carcinoma antigen recognized by T cells 3	9733	ENSG00000075856
9 ☐	P68036	NA	UBE2L3	ubiquitin-conjugating enzyme E2L 3	7332	ENSG00000185651
10 ☐	P30086	NA	PEBP1	phosphatidylethanolamine binding protein 1	5037	ENSG00000089220
11 ☐	P06748	NA	NPM1	nucleophosmin (nucleolar phosphoprotein B23, numatrin)	4869	ENSG00000181163
12 ☐	P61019	NA	RAB2A	RAB2A, member RAS oncogene family	5862	ENSG00000104388
13 ☐	Q02543	NA	RPL18A	ribosomal protein L18a	6142	ENSG00000105640
14 ☐	Q99497	NA	PARK7	parkinson protein 7	11315	ENSG00000116288
15 ☐	P49588	NA	AARS	alanyl-tRNA synthetase	16	ENSG00000090861
16 ☐	P26639	NA	TARS	threonyl-tRNA synthetase	6897	ENSG00000113407
17 ☐	P49207	NA	RPL34	ribosomal protein L34	6164	ENSG00000109475
18 ☐	P62942	NA	FKBP1A	FK506 binding protein 1A, 12kDa	2280	ENSG00000088832
19 ☐	Q9UQE7	NA	SMC3	structural maintenance of chromosomes 3	9126	ENSG00000108055
20 ☐	Q01130	NA	SRSF2	serine/arginine-rich splicing factor 2	6427	ENSG00000161547
21 ☐	P00558	NA	PGK1	phosphoglycerate kinase 1	5230	ENSG00000102144
22 ☐	P55795	NA	HNRNPH2	heterogeneous nuclear ribonucleoprotein H2 (H')	3188	ENSG00000126945
23 ☐	P40616	NA	ARL1	ADP-ribosylation factor-like 1	400	ENSG00000120805
24 ☐	P41567	NA	EIF1	eukaryotic translation initiation factor 1	10209	ENSG00000173812

Database:molecular function			Name:heterocyclic compound binding			ID:GO:1901363
C=5290; O=30; E=17.66; R=1.70; rawP=0.0003; adjP=0.0040						
Index	UserID	Value	Gene Symbol	Gene Name	EntrezGene	Ensembl
25 ☐	Q9H0U4	NA	RAB1B	RAB1B, member RAS oncogene family	81876	ENSG00000174903
26 ☐	Q99536	NA	VAT1	vesicle amine transport protein 1 homolog (T. californica)	10493	ENSG00000108828
27 ☐	P84098	NA	RPL19	ribosomal protein L19	6143	ENSG00000108298
28 ☐	P16989	NA	CSDA	cold shock domain protein A	8531	ENSG00000060138
29 ☐	Q00577	NA	PURA	purine-rich element binding protein A	5813	ENSG00000185129
30 ☐	Q96AE4	NA	FUBP1	far upstream element (FUSE) binding protein 1	8880	ENSG00000162613

Database:molecular function			Name:RAGE receptor binding			ID:GO:0050786
C=9; O=2; E=0.03; R=66.56; rawP=0.0004; adjP=0.0042						
Index	UserID	Value	Gene Symbol	Gene Name	EntrezGene	Ensembl
1 ☐	P09429	NA	HMGB1	high mobility group box 1	3146	ENSG00000189403
2 ☐	P26447	NA	S100A4	S100 calcium binding protein A4	6275	ENSG00000196154

Database:molecular function			Name:organic cyclic compound binding			ID:GO:0097159
C=5342; O=30; E=17.83; R=1.68; rawP=0.0004; adjP=0.0042						
Index	UserID	Value	Gene Symbol	Gene Name	EntrezGene	Ensembl
1 ☐	P39023	NA	RPL3	ribosomal protein L3	6122	ENSG00000100316
2 ☐	P09429	NA	HMGB1	high mobility group box 1	3146	ENSG00000189403
3 ☐	P61106	NA	RAB14	RAB14, member RAS oncogene family	51552	ENSG00000119396
4 ☐	P42766	NA	RPL35	ribosomal protein L35	11224	ENSG00000136942
5 ☐	P63261	NA	ACTG1	actin, gamma 1	71	ENSG00000184009
6 ☐	P22314	NA	UBA1	ubiquitin-like modifier activating enzyme 1	7317	ENSG00000130985
7 ☐	Q9HAV7	NA	GRPEL1	GrpE-like 1, mitochondrial (E. coli)	80273	ENSG00000109519
8 ☐	Q15020	NA	SART3	squamous cell carcinoma antigen recognized by T cells 3	9733	ENSG00000075856
9 ☐	P68036	NA	UBE2L3	ubiquitin-conjugating enzyme E2L 3	7332	ENSG00000185651

Database:molecular function			Name:organic cyclic compound binding			ID:GO:0097159
C=5342; O=30; E=17.83; R=1.68; rawP=0.0004; adjP=0.0042						
Index	UserID	Value	Gene Symbol	Gene Name	EntrezGene	Ensembl
10 ☐	P30086	NA	PEBP1	phosphatidylethanolamine binding protein 1	5037	ENSG00000089220
11 ☐	P06748	NA	NPM1	nucleophosmin (nucleolar phosphoprotein B23, numatrin)	4869	ENSG00000181163
12 ☐	P61019	NA	RAB2A	RAB2A, member RAS oncogene family	5862	ENSG00000104388
13 ☐	Q02543	NA	RPL18A	ribosomal protein L18a	6142	ENSG00000105640
14 ☐	Q99497	NA	PARK7	parkinson protein 7	11315	ENSG00000116288
15 ☐	P49588	NA	AARS	alanyl-tRNA synthetase	16	ENSG00000090861
16 ☐	P26639	NA	TARS	threonyl-tRNA synthetase	6897	ENSG00000113407
17 ☐	P49207	NA	RPL34	ribosomal protein L34	6164	ENSG00000109475
18 ☐	P62942	NA	FKBP1A	FK506 binding protein 1A, 12kDa	2280	ENSG00000088832
19 ☐	Q9UQE7	NA	SMC3	structural maintenance of chromosomes 3	9126	ENSG00000108055
20 ☐	Q01130	NA	SRSF2	serine/arginine-rich splicing factor 2	6427	ENSG00000161547
21 ☐	P00558	NA	PGK1	phosphoglycerate kinase 1	5230	ENSG00000102144
22 ☐	P55795	NA	HNRNPH2	heterogeneous nuclear ribonucleoprotein H2 (H')	3188	ENSG00000126945
23 ☐	P40616	NA	ARL1	ADP-ribosylation factor-like 1	400	ENSG00000120805
24 ☐	P41567	NA	EIF1	eukaryotic translation initiation factor 1	10209	ENSG00000173812
25 ☐	Q9H0U4	NA	RAB1B	RAB1B, member RAS oncogene family	81876	ENSG00000174903
26 ☐	Q99536	NA	VAT1	vesicle amine transport protein 1 homolog (T. californica)	10493	ENSG00000108828
27 ☐	P84098	NA	RPL19	ribosomal protein L19	6143	ENSG00000108298
28 ☐	P16989	NA	CSDA	cold shock domain protein A	8531	ENSG00000060138
29 ☐	Q00577	NA	PURA	purine-rich element binding protein A	5813	ENSG00000185129
30 ☐	Q96AE4	NA	FUBP1	far upstream element (FUSE) binding protein 1	8880	ENSG00000162613

Database:molecular function				Name:nucleoside phosphate binding		ID:GO:1901265
C=2404; O=17; E=8.03; R=2.12; rawP=0.0014; adjP=0.0110						
Index	UserID	Value	Gene Symbol	Gene Name	EntrezGene	Ensembl
1 ☐	P61106	NA	RAB14	RAB14, member RAS oncogene family	51552	ENSG00000119396
2 ☐	P63261	NA	ACTG1	actin, gamma 1	71	ENSG00000184009
3 ☐	P22314	NA	UBA1	ubiquitin-like modifier activating enzyme 1	7317	ENSG00000130985
4 ☐	Q9HAV7	NA	GRPEL1	GrpE-like 1, mitochondrial (E. coli)	80273	ENSG00000109519
5 ☐	Q15020	NA	SART3	squamous cell carcinoma antigen recognized by T cells 3	9733	ENSG00000075856
6 ☐	P68036	NA	UBE2L3	ubiquitin-conjugating enzyme E2L 3	7332	ENSG00000185651
7 ☐	P30086	NA	PEBP1	phosphatidylethanolamine binding protein 1	5037	ENSG00000089220
8 ☐	P61019	NA	RAB2A	RAB2A, member RAS oncogene family	5862	ENSG00000104388
9 ☐	P49588	NA	AARS	alanyl-tRNA synthetase	16	ENSG00000090861
10 ☐	P26639	NA	TARS	threonyl-tRNA synthetase	6897	ENSG00000113407
11 ☐	Q9UQE7	NA	SMC3	structural maintenance of chromosomes 3	9126	ENSG00000108055
12 ☐	Q01130	NA	SRSF2	serine/arginine-rich splicing factor 2	6427	ENSG00000161547
13 ☐	P00558	NA	PGK1	phosphoglycerate kinase 1	5230	ENSG00000102144
14 ☐	P55795	NA	HNRNPH2	heterogeneous nuclear ribonucleoprotein H2 (H')	3188	ENSG00000126945
15 ☐	P40616	NA	ARL1	ADP-ribosylation factor-like 1	400	ENSG00000120805
16 ☐	Q9H0U4	NA	RAB1B	RAB1B, member RAS oncogene family	81876	ENSG00000174903
17 ☐	Q99536	NA	VAT1	vesicle amine transport protein 1 homolog (T. californica)	10493	ENSG00000108828

Database:molecular function				Name:small molecule binding		ID:GO:0036094
C=2595; O=18; E=8.66; R=2.08; rawP=0.0012; adjP=0.0110						
Index	UserID	Value	Gene Symbol	Gene Name	EntrezGene	Ensembl
1 ☐	P61106	NA	RAB14	RAB14, member RAS oncogene family	51552	ENSG00000119396
2 ☐	P63261	NA	ACTG1	actin, gamma 1	71	ENSG00000184009
3 ☐	P22314	NA	UBA1		7317	ENSG00000130985

Database:molecular function			Name:small molecule binding		ID:GO:0036094	
C=2595; O=18; E=8.66; R=2.08; rawP=0.0012; adjP=0.0110						
Index	UserID	Value	Gene Symbol	Gene Name	EntrezGene	Ensembl
				ubiquitin-like modifier activating enzyme 1		
4 ☐	Q9HAV7	NA	GRPEL1	GrpE-like 1, mitochondrial (E. coli)	80273	ENSG00000109519
5 ☐	Q15020	NA	SART3	squamous cell carcinoma antigen recognized by T cells 3	9733	ENSG00000075856
6 ☐	P68036	NA	UBE2L3	ubiquitin-conjugating enzyme E2L 3	7332	ENSG00000185651
7 ☐	P30086	NA	PEBP1	phosphatidylethanolamine binding protein 1	5037	ENSG00000089220
8 ☐	P61019	NA	RAB2A	RAB2A, member RAS oncogene family	5862	ENSG00000104388
9 ☐	P49588	NA	AARS	alanyl-tRNA synthetase	16	ENSG00000090861
10 ☐	P26639	NA	TARS	threonyl-tRNA synthetase	6897	ENSG00000113407
11 ☐	Q9UQE7	NA	SMC3	structural maintenance of chromosomes 3	9126	ENSG00000108055
12 ☐	Q01581	NA	HMGCS1	3-hydroxy-3-methylglutaryl-CoA synthase 1 (soluble)	3157	ENSG00000112972
13 ☐	Q01130	NA	SRSF2	serine/arginine-rich splicing factor 2	6427	ENSG00000161547
14 ☐	P00558	NA	PGK1	phosphoglycerate kinase 1	5230	ENSG00000102144
15 ☐	P55795	NA	HNRNPH2	heterogeneous nuclear ribonucleoprotein H2 (H')	3188	ENSG00000126945
16 ☐	P40616	NA	ARL1	ADP-ribosylation factor-like 1	400	ENSG00000120805
17 ☐	Q9H0U4	NA	RAB1B	RAB1B, member RAS oncogene family	81876	ENSG00000174903
18 ☐	Q99536	NA	VAT1	vesicle amine transport protein 1 homolog (T. californica)	10493	ENSG00000108828

Database:molecular function			Name:nucleotide binding		ID:GO:0000166	
C=2403; O=17; E=8.02; R=2.12; rawP=0.0014; adjP=0.0110						
Index	UserID	Value	Gene Symbol	Gene Name	EntrezGene	Ensembl
1 ☐	P61106	NA	RAB14	RAB14, member RAS oncogene family	51552	ENSG00000119396
2 ☐	P63261	NA	ACTG1	actin, gamma 1	71	ENSG00000184009
3 ☐	P22314	NA	UBA1	ubiquitin-like modifier activating enzyme 1	7317	ENSG00000130985

Database:molecular function			Name:nucleotide binding		ID:GO:0000166	
C=2403; O=17; E=8.02; R=2.12; rawP=0.0014; adjP=0.0110						
Index	UserID	Value	Gene Symbol	Gene Name	EntrezGene	Ensembl
4 ☐	Q9HAV7	NA	GRPEL1	GrpE-like 1, mitochondrial (E. coli)	80273	ENSG00000109519
5 ☐	Q15020	NA	SART3	squamous cell carcinoma antigen recognized by T cells 3	9733	ENSG00000075856
6 ☐	P68036	NA	UBE2L3	ubiquitin-conjugating enzyme E2L 3	7332	ENSG00000185651
7 ☐	P30086	NA	PEBP1	phosphatidylethanolamine binding protein 1	5037	ENSG00000089220
8 ☐	P61019	NA	RAB2A	RAB2A, member RAS oncogene family	5862	ENSG00000104388
9 ☐	P49588	NA	AARS	alanyl-tRNA synthetase	16	ENSG00000090861
10 ☐	P26639	NA	TARS	threonyl-tRNA synthetase	6897	ENSG00000113407
11 ☐	Q9UQE7	NA	SMC3	structural maintenance of chromosomes 3	9126	ENSG00000108055
12 ☐	Q01130	NA	SRSF2	serine/arginine-rich splicing factor 2	6427	ENSG00000161547
13 ☐	P00558	NA	PGK1	phosphoglycerate kinase 1	5230	ENSG00000102144
14 ☐	P55795	NA	HNRNPH2	heterogeneous nuclear ribonucleoprotein H2 (H')	3188	ENSG00000126945
15 ☐	P40616	NA	ARL1	ADP-ribosylation factor-like 1	400	ENSG00000120805
16 ☐	Q9H0U4	NA	RAB1B	RAB1B, member RAS oncogene family	81876	ENSG00000174903
17 ☐	Q99536	NA	VAT1	vesicle amine transport protein 1 homolog (T. californica)	10493	ENSG00000108828

Database:molecular function			Name:mRNA binding		ID:GO:0003729	
C=90; O=3; E=0.30; R=9.98; rawP=0.0034; adjP=0.0246						
Index	UserID	Value	Gene Symbol	Gene Name	EntrezGene	Ensembl
1 ☐	P42766	NA	RPL35	ribosomal protein L35	11224	ENSG00000136942
2 ☐	P16989	NA	CSDA	cold shock domain protein A	8531	ENSG00000060138
3 ☐	Q99497	NA	PARK7	parkinson protein 7	11315	ENSG00000116288

Database:molecular function Name:GDP binding ID:GO:0019003						
C=29; O=2; E=0.10; R=20.66; rawP=0.0042; adjP=0.0282						
Index	UserID	Value	Gene Symbol	Gene Name	EntrezGene	Ensembl
1 ☐	P61019	NA	RAB2A	RAB2A, member RAS oncogene family	5862	ENSG00000104388
2 ☐	P61106	NA	RAB14	RAB14, member RAS oncogene family	51552	ENSG00000119396

Database:molecular function Name:purine nucleotide binding ID:GO:0017076						
C=1842; O=13; E=6.15; R=2.11; rawP=0.0062; adjP=0.0376						
Index	UserID	Value	Gene Symbol	Gene Name	EntrezGene	Ensembl
1 ☐	P61106	NA	RAB14	RAB14, member RAS oncogene family	51552	ENSG00000119396
2 ☐	Q9UQE7	NA	SMC3	structural maintenance of chromosomes 3	9126	ENSG00000108055
3 ☐	P63261	NA	ACTG1	actin, gamma 1	71	ENSG00000184009
4 ☐	P22314	NA	UBA1	ubiquitin-like modifier activating enzyme 1	7317	ENSG00000130985
5 ☐	P00558	NA	PGK1	phosphoglycerate kinase 1	5230	ENSG00000102144
6 ☐	Q9HAV7	NA	GRPEL1	GrpE-like 1, mitochondrial (E. coli)	80273	ENSG00000109519
7 ☐	P40616	NA	ARL1	ADP-ribosylation factor-like 1	400	ENSG00000120805
8 ☐	P68036	NA	UBE2L3	ubiquitin-conjugating enzyme E2L 3	7332	ENSG00000185651
9 ☐	Q9H0U4	NA	RAB1B	RAB1B, member RAS oncogene family	81876	ENSG00000174903
10 ☐	P30086	NA	PEBP1	phosphatidylethanolamine binding protein 1	5037	ENSG00000089220
11 ☐	P61019	NA	RAB2A	RAB2A, member RAS oncogene family	5862	ENSG00000104388
12 ☐	P49588	NA	AARS	alanyl-tRNA synthetase	16	ENSG00000090861
13 ☐	P26639	NA	TARS	threonyl-tRNA synthetase	6897	ENSG00000113407

Database:molecular function Name:peroxidase activity ID:GO:0004601						
C=37; O=2; E=0.12; R=16.19; rawP=0.0068; adjP=0.0376						
Index	UserID	Value	Gene Symbol	Gene Name	EntrezGene	Ensembl
1 ☐	P30041	NA	PRDX6	peroxiredoxin 6	9588	ENSG00000117592
2 ☐	Q99497	NA	PARK7	parkinson protein 7	11315	ENSG00000116288

**Database:molecular function Name:oxidoreductase activity, acting on peroxide as acceptor ID:GO:0016684**						
C=37; O=2; E=0.12; R=16.19; rawP=0.0068; adjP=0.0376						
Index	UserID	Value	Gene Symbol	Gene Name	EntrezGene	Ensembl
1 ☐	P30041	NA	PRDX6	peroxiredoxin 6	9588	ENSG00000117592
2 ☐	Q99497	NA	PARK7	parkinson protein 7	11315	ENSG00000116288

**Database:molecular function Name:ligase activity, forming carbon-oxygen bonds ID:GO:0016875**						
C=44; O=2; E=0.15; R=13.62; rawP=0.0094; adjP=0.0442						
Index	UserID	Value	Gene Symbol	Gene Name	EntrezGene	Ensembl
1 ☐	P49588	NA	AARS	alanyl-tRNA synthetase	16	ENSG00000090861
2 ☐	P26639	NA	TARS	threonyl-tRNA synthetase	6897	ENSG00000113407

**Database:molecular function Name:aminoacyl-tRNA ligase activity ID:GO:0004812**						
C=44; O=2; E=0.15; R=13.62; rawP=0.0094; adjP=0.0442						
Index	UserID	Value	Gene Symbol	Gene Name	EntrezGene	Ensembl
1 ☐	P49588	NA	AARS	alanyl-tRNA synthetase	16	ENSG00000090861
2 ☐	P26639	NA	TARS	threonyl-tRNA synthetase	6897	ENSG00000113407

**Database:molecular function Name:ligase activity, forming aminoacyl-tRNA and related compounds ID:GO:0016876**						
C=44; O=2; E=0.15; R=13.62; rawP=0.0094; adjP=0.0442						
Index	UserID	Value	Gene Symbol	Gene Name	EntrezGene	Ensembl
1 ☐	P49588	NA	AARS	alanyl-tRNA synthetase	16	ENSG00000090861
2 ☐	P26639	NA	TARS	threonyl-tRNA synthetase	6897	ENSG00000113407

**Database:molecular function Name:purine nucleoside binding ID:GO:0001883**						
C=1812; O=12; E=6.05; R=1.98; rawP=0.0142; adjP=0.0517						
Index	UserID	Value	Gene Symbol	Gene Name	EntrezGene	Ensembl
1 ☐	P61106	NA	RAB14	RAB14, member RAS oncogene family	51552	ENSG00000119396
2 ☐	Q9UQE7	NA	SMC3		9126	ENSG00000108055

Database:molecular function			Name:purine nucleoside binding		ID:GO:0001883	
C=1812; O=12; E=6.05; R=1.98; rawP=0.0142; adjP=0.0517						
Index	UserID	Value	Gene Symbol	Gene Name	EntrezGene	Ensembl
				structural maintenance of chromosomes 3		
3 ☐	P63261	NA	ACTG1	actin, gamma 1	71	ENSG00000184009
4 ☐	P22314	NA	UBA1	ubiquitin-like modifier activating enzyme 1	7317	ENSG00000130985
5 ☐	P00558	NA	PGK1	phosphoglycerate kinase 1	5230	ENSG00000102144
6 ☐	P40616	NA	ARL1	ADP-ribosylation factor-like 1	400	ENSG00000120805
7 ☐	P68036	NA	UBE2L3	ubiquitin-conjugating enzyme E2L 3	7332	ENSG00000185651
8 ☐	Q9H0U4	NA	RAB1B	RAB1B, member RAS oncogene family	81876	ENSG00000174903
9 ☐	P30086	NA	PEBP1	phosphatidylethanolamine binding protein 1	5037	ENSG00000089220
10 ☐	P61019	NA	RAB2A	RAB2A, member RAS oncogene family	5862	ENSG00000104388
11 ☐	P49588	NA	AARS	alanyl-tRNA synthetase	16	ENSG00000090861
12 ☐	P26639	NA	TARS	threonyl-tRNA synthetase	6897	ENSG00000113407

Database:molecular function			Name:ribonucleoside binding		ID:GO:0032549	
C=1813; O=12; E=6.05; R=1.98; rawP=0.0143; adjP=0.0517						
Index	UserID	Value	Gene Symbol	Gene Name	EntrezGene	Ensembl
1 ☐	P61106	NA	RAB14	RAB14, member RAS oncogene family	51552	ENSG00000119396
2 ☐	Q9UQE7	NA	SMC3	structural maintenance of chromosomes 3	9126	ENSG00000108055
3 ☐	P63261	NA	ACTG1	actin, gamma 1	71	ENSG00000184009
4 ☐	P22314	NA	UBA1	ubiquitin-like modifier activating enzyme 1	7317	ENSG00000130985
5 ☐	P00558	NA	PGK1	phosphoglycerate kinase 1	5230	ENSG00000102144
6 ☐	P40616	NA	ARL1	ADP-ribosylation factor-like 1	400	ENSG00000120805
7 ☐	P68036	NA	UBE2L3	ubiquitin-conjugating enzyme E2L 3	7332	ENSG00000185651
8 ☐	Q9H0U4	NA	RAB1B	RAB1B, member RAS oncogene family	81876	ENSG00000174903
9 ☐	P30086	NA	PEBP1	phosphatidylethanolamine binding protein 1	5037	ENSG00000089220
10 ☐	P61019	NA	RAB2A		5862	ENSG00000104388

Database:molecular function			Name:ribonucleoside binding			ID:GO:0032549
C=1813; O=12; E=6.05; R=1.98; rawP=0.0143; adjP=0.0517						
Index	UserID	Value	Gene Symbol	Gene Name	EntrezGene	Ensembl
				RAB2A, member RAS oncogene family		
11 ☐	P49588	NA	AARS	alanyl-tRNA synthetase	16	ENSG00000090861
12 ☐	P26639	NA	TARS	threonyl-tRNA synthetase	6897	ENSG00000113407

Database:molecular function			Name:purine ribonucleoside binding			ID:GO:0032550
C=1809; O=12; E=6.04; R=1.99; rawP=0.0140; adjP=0.0517						
Index	UserID	Value	Gene Symbol	Gene Name	EntrezGene	Ensembl
1 ☐	P61106	NA	RAB14	RAB14, member RAS oncogene family	51552	ENSG00000119396
2 ☐	Q9UQE7	NA	SMC3	structural maintenance of chromosomes 3	9126	ENSG00000108055
3 ☐	P63261	NA	ACTG1	actin, gamma 1	71	ENSG00000184009
4 ☐	P22314	NA	UBA1	ubiquitin-like modifier activating enzyme 1	7317	ENSG00000130985
5 ☐	P00558	NA	PGK1	phosphoglycerate kinase 1	5230	ENSG00000102144
6 ☐	P40616	NA	ARL1	ADP-ribosylation factor-like 1	400	ENSG00000120805
7 ☐	P68036	NA	UBE2L3	ubiquitin-conjugating enzyme E2L 3	7332	ENSG00000185651
8 ☐	Q9H0U4	NA	RAB1B	RAB1B, member RAS oncogene family	81876	ENSG00000174903
9 ☐	P30086	NA	PEBP1	phosphatidylethanolamine binding protein 1	5037	ENSG00000089220
10 ☐	P61019	NA	RAB2A	RAB2A, member RAS oncogene family	5862	ENSG00000104388
11 ☐	P49588	NA	AARS	alanyl-tRNA synthetase	16	ENSG00000090861
12 ☐	P26639	NA	TARS	threonyl-tRNA synthetase	6897	ENSG00000113407

Database:molecular function			Name:isomerase activity			ID:GO:0016853
C=142; O=3; E=0.47; R=6.33; rawP=0.0118; adjP=0.0517						
Index	UserID	Value	Gene Symbol	Gene Name	EntrezGene	Ensembl
1 ☐	P62942	NA	FKBP1A	FK506 binding protein 1A, 12kDa	2280	ENSG00000088832
2 ☐	Q01581	NA	HMGCS1	3-hydroxy-3-methylglutaryl-CoA synthase 1 (soluble)	3157	ENSG00000112972
3 ☐	P30040	NA	ERP29		10961	ENSG00000089248

Database:molecular function Name:isomerase activity ID:GO:0016853						
C=142; O=3; E=0.47; R=6.33; rawP=0.0118; adjP=0.0517						
Index	UserID	Value	Gene Symbol	Gene Name	EntrezGene	Ensembl
				endoplasmic reticulum protein 29		

Database:molecular function Name:double-stranded DNA binding ID:GO:0003690						
C=151; O=3; E=0.50; R=5.95; rawP=0.0139; adjP=0.0517						
Index	UserID	Value	Gene Symbol	Gene Name	EntrezGene	Ensembl
1 ☐	P09429	NA	HMGB1	high mobility group box 1	3146	ENSG00000189403
2 ☐	P16989	NA	CSDA	cold shock domain protein A	8531	ENSG00000060138
3 ☐	Q00577	NA	PURA	purine-rich element binding protein A	5813	ENSG00000185129

Database:molecular function Name:purine ribonucleoside triphosphate binding ID:GO:0035639						
C=1800; O=12; E=6.01; R=2.00; rawP=0.0135; adjP=0.0517						
Index	UserID	Value	Gene Symbol	Gene Name	EntrezGene	Ensembl
1 ☐	P61106	NA	RAB14	RAB14, member RAS oncogene family	51552	ENSG00000119396
2 ☐	Q9UQE7	NA	SMC3	structural maintenance of chromosomes 3	9126	ENSG00000108055
3 ☐	P63261	NA	ACTG1	actin, gamma 1	71	ENSG00000184009
4 ☐	P22314	NA	UBA1	ubiquitin-like modifier activating enzyme 1	7317	ENSG00000130985
5 ☐	P00558	NA	PGK1	phosphoglycerate kinase 1	5230	ENSG00000102144
6 ☐	P40616	NA	ARL1	ADP-ribosylation factor-like 1	400	ENSG00000120805
7 ☐	P68036	NA	UBE2L3	ubiquitin-conjugating enzyme E2L 3	7332	ENSG00000185651
8 ☐	Q9H0U4	NA	RAB1B	RAB1B, member RAS oncogene family	81876	ENSG00000174903
9 ☐	P30086	NA	PEBP1	phosphatidylethanolamine binding protein 1	5037	ENSG00000089220
10 ☐	P61019	NA	RAB2A	RAB2A, member RAS oncogene family	5862	ENSG00000104388
11 ☐	P49588	NA	AARS	alanyl-tRNA synthetase	16	ENSG00000090861
12 ☐	P26639	NA	TARS	threonyl-tRNA synthetase	6897	ENSG00000113407

Database:molecular function			Name:nucleoside binding		ID:GO:0001882	
C=1823; O=12; E=6.09; R=1.97; rawP=0.0149; adjP=0.0519						
Index	UserID	Value	Gene Symbol	Gene Name	EntrezGene	Ensembl
1 ☐	P61106	NA	RAB14	RAB14, member RAS oncogene family	51552	ENSG00000119396
2 ☐	Q9UQE7	NA	SMC3	structural maintenance of chromosomes 3	9126	ENSG00000108055
3 ☐	P63261	NA	ACTG1	actin, gamma 1	71	ENSG00000184009
4 ☐	P22314	NA	UBA1	ubiquitin-like modifier activating enzyme 1	7317	ENSG00000130985
5 ☐	P00558	NA	PGK1	phosphoglycerate kinase 1	5230	ENSG00000102144
6 ☐	P40616	NA	ARL1	ADP-ribosylation factor-like 1	400	ENSG00000120805
7 ☐	P68036	NA	UBE2L3	ubiquitin-conjugating enzyme E2L 3	7332	ENSG00000185651
8 ☐	Q9H0U4	NA	RAB1B	RAB1B, member RAS oncogene family	81876	ENSG00000174903
9 ☐	P30086	NA	PEBP1	phosphatidylethanolamine binding protein 1	5037	ENSG00000089220
10 ☐	P61019	NA	RAB2A	RAB2A, member RAS oncogene family	5862	ENSG00000104388
11 ☐	P49588	NA	AARS	alanyl-tRNA synthetase	16	ENSG00000090861
12 ☐	P26639	NA	TARS	threonyl-tRNA synthetase	6897	ENSG00000113407

Database:molecular function			Name:purine ribonucleotide binding		ID:GO:0032555	
C=1835; O=12; E=6.13; R=1.96; rawP=0.0156; adjP=0.0524						
Index	UserID	Value	Gene Symbol	Gene Name	EntrezGene	Ensembl
1 ☐	P61106	NA	RAB14	RAB14, member RAS oncogene family	51552	ENSG00000119396
2 ☐	Q9UQE7	NA	SMC3	structural maintenance of chromosomes 3	9126	ENSG00000108055
3 ☐	P63261	NA	ACTG1	actin, gamma 1	71	ENSG00000184009
4 ☐	P22314	NA	UBA1	ubiquitin-like modifier activating enzyme 1	7317	ENSG00000130985
5 ☐	P00558	NA	PGK1	phosphoglycerate kinase 1	5230	ENSG00000102144
6 ☐	P40616	NA	ARL1	ADP-ribosylation factor-like 1	400	ENSG00000120805
7 ☐	P68036	NA	UBE2L3	ubiquitin-conjugating enzyme E2L 3	7332	ENSG00000185651
8 ☐	Q9H0U4	NA	RAB1B		81876	ENSG00000174903

Database:molecular function			Name:purine ribonucleotide binding			ID:GO:0032555
C=1835; O=12; E=6.13; R=1.96; rawP=0.0156; adjP=0.0524						
Index	UserID	Value	Gene Symbol	Gene Name	EntrezGene	Ensembl
				RAB1B, member RAS oncogene family		
9 ☐	P30086	NA	PEBP1	phosphatidylethanolamine binding protein 1	5037	ENSG00000089220
10 ☐	P61019	NA	RAB2A	RAB2A, member RAS oncogene family	5862	ENSG00000104388
11 ☐	P49588	NA	AARS	alanyl-tRNA synthetase	16	ENSG00000090861
12 ☐	P26639	NA	TARS	threonyl-tRNA synthetase	6897	ENSG00000113407

Database:molecular function			Name:ribonucleotide binding			ID:GO:0032553
C=1850; O=12; E=6.18; R=1.94; rawP=0.0166; adjP=0.0538						
Index	UserID	Value	Gene Symbol	Gene Name	EntrezGene	Ensembl
1 ☐	P61106	NA	RAB14	RAB14, member RAS oncogene family	51552	ENSG00000119396
2 ☐	Q9UQE7	NA	SMC3	structural maintenance of chromosomes 3	9126	ENSG00000108055
3 ☐	P63261	NA	ACTG1	actin, gamma 1	71	ENSG00000184009
4 ☐	P22314	NA	UBA1	ubiquitin-like modifier activating enzyme 1	7317	ENSG00000130985
5 ☐	P00558	NA	PGK1	phosphoglycerate kinase 1	5230	ENSG00000102144
6 ☐	P40616	NA	ARL1	ADP-ribosylation factor-like 1	400	ENSG00000120805
7 ☐	P68036	NA	UBE2L3	ubiquitin-conjugating enzyme E2L 3	7332	ENSG00000185651
8 ☐	Q9H0U4	NA	RAB1B	RAB1B, member RAS oncogene family	81876	ENSG00000174903
9 ☐	P30086	NA	PEBP1	phosphatidylethanolamine binding protein 1	5037	ENSG00000089220
10 ☐	P61019	NA	RAB2A	RAB2A, member RAS oncogene family	5862	ENSG00000104388
11 ☐	P49588	NA	AARS	alanyl-tRNA synthetase	16	ENSG00000090861
12 ☐	P26639	NA	TARS	threonyl-tRNA synthetase	6897	ENSG00000113407

Database:molecular function			Name:antioxidant activity			ID:GO:0016209
C=61; O=2; E=0.20; R=9.82; rawP=0.0176; adjP=0.0551						
Index	UserID	Value	Gene Symbol	Gene Name	EntrezGene	Ensembl
1 ☐	P30041	NA	PRDX6	peroxiredoxin 6	9588	ENSG00000117592
2 ☐	Q99497	NA	PARK7		11315	ENSG00000116288

Database:molecular function Name:antioxidant activity ID:GO:0016209						
C=61; O=2; E=0.20; R=9.82; rawP=0.0176; adjP=0.0551						
Index	UserID	Value	Gene Symbol	Gene Name	EntrezGene	Ensembl
				parkinson protein 7		

Database:molecular function Name:SMAD binding ID:GO:0046332						
C=63; O=2; E=0.21; R=9.51; rawP=0.0187; adjP=0.0567						
Index	UserID	Value	Gene Symbol	Gene Name	EntrezGene	Ensembl
1 ☐	P62942	NA	FKBP1A	FK506 binding protein 1A, 12kDa	2280	ENSG00000088832
2 ☐	Q00577	NA	PURA	purine-rich element binding protein A	5813	ENSG00000185129

Database:molecular function Name:identical protein binding ID:GO:0042802						
C=860; O=7; E=2.87; R=2.44; rawP=0.0232; adjP=0.0682						
Index	UserID	Value	Gene Symbol	Gene Name	EntrezGene	Ensembl
1 ☐	P06748	NA	NPM1	nucleophosmin (nucleolar phosphoprotein B23, numatrin)	4869	ENSG00000181163
2 ☐	Q01581	NA	HMGCS1	3-hydroxy-3-methylglutaryl-CoA synthase 1 (soluble)	3157	ENSG00000112972
3 ☐	P63261	NA	ACTG1	actin, gamma 1	71	ENSG00000184009
4 ☐	Q9HAV7	NA	GRPEL1	GrpE-like 1, mitochondrial (E. coli)	80273	ENSG00000109519
5 ☐	P26447	NA	S100A4	S100 calcium binding protein A4	6275	ENSG00000196154
6 ☐	Q99497	NA	PARK7	parkinson protein 7	11315	ENSG00000116288
7 ☐	P26639	NA	TARS	threonyl-tRNA synthetase	6897	ENSG00000113407

Database:molecular function Name:structure-specific DNA binding ID:GO:0043566						
C=192; O=3; E=0.64; R=4.68; rawP=0.0262; adjP=0.0735						
Index	UserID	Value	Gene Symbol	Gene Name	EntrezGene	Ensembl
1 ☐	P09429	NA	HMGB1	high mobility group box 1	3146	ENSG00000189403
2 ☐	P16989	NA	CSDA		8531	ENSG00000060138

Database:molecular function Name:structure-specific DNA binding ID:GO:0043566						
C=192; O=3; E=0.64; R=4.68; rawP=0.0262; adjP=0.0735						
Index	UserID	Value	Gene Symbol	Gene Name	EntrezGene	Ensembl
				cold shock domain protein A		
3 ☐	Q00577	NA	PURA	purine-rich element binding protein A	5813	ENSG00000185129

Database:molecular function Name:structural constituent of cytoskeleton ID:GO:0005200						
C=76; O=2; E=0.25; R=7.88; rawP=0.0266; adjP=0.0735						
Index	UserID	Value	Gene Symbol	Gene Name	EntrezGene	Ensembl
1 ☐	P09493	NA	TPM1	tropomyosin 1 (alpha)	7168	ENSG00000140416
2 ☐	P63261	NA	ACTG1	actin, gamma 1	71	ENSG00000184009

Database:molecular function Name:guanyl ribonucleotide binding ID:GO:0032561						
C=383; O=4; E=1.28; R=3.13; rawP=0.0386; adjP=0.0919						
Index	UserID	Value	Gene Symbol	Gene Name	EntrezGene	Ensembl
1 ☐	P61019	NA	RAB2A	RAB2A, member RAS oncogene family	5862	ENSG00000104388
2 ☐	P61106	NA	RAB14	RAB14, member RAS oncogene family	51552	ENSG00000119396
3 ☐	P40616	NA	ARL1	ADP-ribosylation factor-like 1	400	ENSG00000120805
4 ☐	Q9H0U4	NA	RAB1B	RAB1B, member RAS oncogene family	81876	ENSG00000174903

Database:molecular function Name:GTP binding ID:GO:0005525						
C=370; O=4; E=1.24; R=3.24; rawP=0.0346; adjP=0.0919						
Index	UserID	Value	Gene Symbol	Gene Name	EntrezGene	Ensembl
1 ☐	P61019	NA	RAB2A	RAB2A, member RAS oncogene family	5862	ENSG00000104388
2 ☐	P61106	NA	RAB14	RAB14, member RAS oncogene family	51552	ENSG00000119396
3 ☐	P40616	NA	ARL1	ADP-ribosylation factor-like 1	400	ENSG00000120805

Database:molecular function Name:GTP binding ID:GO:0005525						
C=370; O=4; E=1.24; R=3.24; rawP=0.0346; adjP=0.0919						
Index	UserID	Value	Gene Symbol	Gene Name	EntrezGene	Ensembl
4 ☐	Q9H0U4	NA	RAB1B	RAB1B, member RAS oncogene family	81876	ENSG00000174903

Database:molecular function Name:GTPase activity ID:GO:0003924						
C=225; O=3; E=0.75; R=3.99; rawP=0.0391; adjP=0.0919						
Index	UserID	Value	Gene Symbol	Gene Name	EntrezGene	Ensembl
1 ☐	P61019	NA	RAB2A	RAB2A, member RAS oncogene family	5862	ENSG00000104388
2 ☐	P61106	NA	RAB14	RAB14, member RAS oncogene family	51552	ENSG00000119396
3 ☐	P40616	NA	ARL1	ADP-ribosylation factor-like 1	400	ENSG00000120805

Database:molecular function Name:drug binding ID:GO:0008144						
C=89; O=2; E=0.30; R=6.73; rawP=0.0356; adjP=0.0919						
Index	UserID	Value	Gene Symbol	Gene Name	EntrezGene	Ensembl
1 ☐	P62942	NA	FKBP1A	FK506 binding protein 1A, 12kDa	2280	ENSG00000088832
2 ☐	Q01581	NA	HMGCS1	3-hydroxy-3-methylglutaryl-CoA synthase 1 (soluble)	3157	ENSG00000112972

Database:molecular function Name:protein transporter activity ID:GO:0008565						
C=91; O=2; E=0.30; R=6.58; rawP=0.0370; adjP=0.0919						
Index	UserID	Value	Gene Symbol	Gene Name	EntrezGene	Ensembl
1 ☐	O43747	NA	AP1G1	adaptor-related protein complex 1, gamma 1 subunit	164	ENSG00000166747
2 ☐	P60059	NA	SEC61G	Sec61 gamma subunit	23480	ENSG00000132432

Database:molecular function Name:guanyl nucleotide binding ID:GO:0019001						
C=383; O=4; E=1.28; R=3.13; rawP=0.0386; adjP=0.0919						
Index	UserID	Value	Gene Symbol	Gene Name	EntrezGene	Ensembl
1 ☐	P61019	NA	RAB2A	RAB2A, member RAS oncogene family	5862	ENSG00000104388

Database:molecular function			Name:guanyl nucleotide binding			ID:GO:0019001
C=383; O=4; E=1.28; R=3.13; rawP=0.0386; adjP=0.0919						
Index	UserID	Value	Gene Symbol	Gene Name	EntrezGene	Ensembl
2 ☐	P61106	NA	RAB14	RAB14, member RAS oncogene family	51552	ENSG00000119396
3 ☐	P40616	NA	ARL1	ADP-ribosylation factor-like 1	400	ENSG00000120805
4 ☐	Q9H0U4	NA	RAB1B	RAB1B, member RAS oncogene family	81876	ENSG00000174903

Database:cellular component			Name:cytosolic large ribosomal subunit			ID:GO:0022625
C=52; O=8; E=0.16; R=50.32; rawP=2.92e-12; adjP=3.07e-10						
Index	UserID	Value	Gene Symbol	Gene Name	EntrezGene	Ensembl
1 ☐	P39023	NA	RPL3	ribosomal protein L3	6122	ENSG00000100316
2 ☐	P42766	NA	RPL35	ribosomal protein L35	11224	ENSG00000136942
3 ☐	P27635	NA	RPL10	ribosomal protein L10	6134	ENSG00000147403
4 ☐	P84098	NA	RPL19	ribosomal protein L19	6143	ENSG00000108298
5 ☐	Q02543	NA	RPL18A	ribosomal protein L18a	6142	ENSG00000105640
6 ☐	P40429	NA	RPL13A	ribosomal protein L13a	23521	ENSG00000142541
7 ☐	P49207	NA	RPL34	ribosomal protein L34	6164	ENSG00000109475
8 ☐	Q9Y3U8	NA	RPL36	ribosomal protein L36	25873	ENSG00000130255

Database:cellular component			Name:cytoplasm			ID:GO:0005737
C=9051; O=49; E=27.67; R=1.77; rawP=2.58e-11; adjP=1.35e-09						
Index	UserID	Value	Gene Symbol	Gene Name	EntrezGene	Ensembl
1 ☐	P39023	NA	RPL3	ribosomal protein L3	6122	ENSG00000100316
2 ☐	P09429	NA	HMGB1	high mobility group box 1	3146	ENSG00000189403
3 ☐	P61106	NA	RAB14	RAB14, member RAS oncogene family	51552	ENSG00000119396
4 ☐	P30041	NA	PRDX6	peroxiredoxin 6	9588	ENSG00000117592
5 ☐	P63261	NA	ACTG1	actin, gamma 1	71	ENSG00000184009
6 ☐	P60059	NA	SEC61G	Sec61 gamma subunit	23480	ENSG00000132432

Database:cellular component Name:cytoplasm ID:GO:0005737						
C=9051; O=49; E=27.67; R=1.77; rawP=2.58e-11; adjP=1.35e-09						
Index	UserID	Value	Gene Symbol	Gene Name	EntrezGene	Ensembl
7 ☐	P30086	NA	PEBP1	phosphatidylethanolamine binding protein 1	5037	ENSG00000089220
8 ☐	P61019	NA	RAB2A	RAB2A, member RAS oncogene family	5862	ENSG00000104388
9 ☐	P06748	NA	NPM1	nucleophosmin (nucleolar phosphoprotein B23, numatrin)	4869	ENSG00000181163
10 ☐	P30040	NA	ERP29	endoplasmic reticulum protein 29	10961	ENSG00000089248
11 ☐	Q99497	NA	PARK7	parkinson protein 7	11315	ENSG00000116288
12 ☐	P49588	NA	AARS	alanyl-tRNA synthetase	16	ENSG00000090861
13 ☐	Q13126	NA	MTAP	methylthioadenosine phosphorylase	4507	ENSG00000099810
14 ☐	P40429	NA	RPL13A	ribosomal protein L13a	23521	ENSG00000142541
15 ☐	P49207	NA	RPL34	ribosomal protein L34	6164	ENSG00000109475
16 ☐	P62942	NA	FKBP1A	FK506 binding protein 1A, 12kDa	2280	ENSG00000088832
17 ☐	Q01581	NA	HMGCS1	3-hydroxy-3-methylglutaryl-CoA synthase 1 (soluble)	3157	ENSG00000112972
18 ☐	Q9UQE7	NA	SMC3	structural maintenance of chromosomes 3	9126	ENSG00000108055
19 ☐	P27635	NA	RPL10	ribosomal protein L10	6134	ENSG00000147403
20 ☐	P40616	NA	ARL1	ADP-ribosylation factor-like 1	400	ENSG00000120805
21 ☐	P09972	NA	ALDOC	aldolase C, fructose-bisphosphate	230	ENSG00000109107
22 ☐	P41567	NA	EIF1	eukaryotic translation initiation factor 1	10209	ENSG00000173812
23 ☐	Q9H0U4	NA	RAB1B	RAB1B, member RAS oncogene family	81876	ENSG00000174903
24 ☐	O75390	NA	CS	citrate synthase	1431	ENSG00000062485
25 ☐	P15121	NA	AKR1B1	aldo-keto reductase family 1, member B1 (aldose reductase)	231	ENSG00000085662
26 ☐	Q99536	NA	VAT1	vesicle amine transport protein 1 homolog (T. californica)	10493	ENSG00000108828
27 ☐	P84098	NA	RPL19	ribosomal protein L19	6143	ENSG00000108298
28 ☐	P16989	NA	CSDA	cold shock domain protein A	8531	ENSG00000060138
29 ☐	Q96AG4	NA	LRRC59		55379	ENSG00000108829

Database:cellular component Name:cytoplasm ID:GO:0005737						
C=9051; O=49; E=27.67; R=1.77; rawP=2.58e-11; adjP=1.35e-09						
Index	UserID	Value	Gene Symbol	Gene Name	EntrezGene	Ensembl
				leucine rich repeat containing 59		
30 ☐	Q9Y266	NA	NUDC	nuclear distribution C homolog (A. nidulans)	10726	ENSG00000090273
31 ☐	O43396	NA	TXNL1	thioredoxin-like 1	9352	ENSG00000091164
32 ☐	P42766	NA	RPL35	ribosomal protein L35	11224	ENSG00000136942
33 ☐	Q9HAV7	NA	GRPEL1	GrpE-like 1, mitochondrial (E. coli)	80273	ENSG00000109519
34 ☐	P26447	NA	S100A4	S100 calcium binding protein A4	6275	ENSG00000196154
35 ☐	Q15020	NA	SART3	squamous cell carcinoma antigen recognized by T cells 3	9733	ENSG00000075856
36 ☐	P68036	NA	UBE2L3	ubiquitin-conjugating enzyme E2L 3	7332	ENSG00000185651
37 ☐	Q02543	NA	RPL18A	ribosomal protein L18a	6142	ENSG00000105640
38 ☐	Q14019	NA	COTL1	coactosin-like 1 (Dictyostelium)	23406	ENSG00000103187
39 ☐	P26639	NA	TARS	threonyl-tRNA synthetase	6897	ENSG00000113407
40 ☐	Q9Y281	NA	CFL2	cofilin 2 (muscle)	1073	ENSG00000165410
41 ☐	Q9Y3U8	NA	RPL36	ribosomal protein L36	25873	ENSG00000130255
42 ☐	P00558	NA	PGK1	phosphoglycerate kinase 1	5230	ENSG00000102144
43 ☐	O75915	NA	ARL6IP5	ADP-ribosylation-like factor 6 interacting protein 5	10550	ENSG00000144746
44 ☐	P55795	NA	HNRNPH2	heterogeneous nuclear ribonucleoprotein H2 (H')	3188	ENSG00000126945
45 ☐	Q9BTT0	NA	ANP32E	acidic (leucine-rich) nuclear phosphoprotein 32 family, member E	81611	ENSG00000143401
46 ☐	P09493	NA	TPM1	tropomyosin 1 (alpha)	7168	ENSG00000140416
47 ☐	O43747	NA	AP1G1	adaptor-related protein complex 1, gamma 1 subunit	164	ENSG00000166747
48 ☐	Q00577	NA	PURA	purine-rich element binding protein A	5813	ENSG00000185129
49 ☐	P13693	NA	TPT1	tumor protein, translationally-controlled 1	7178	ENSG00000133112

Database:cellular component			Name:large ribosomal subunit		ID:GO:0015934	
C=74; O=8; E=0.23; R=35.36; rawP=5.55e-11; adjP=1.94e-09						
Index	UserID	Value	Gene Symbol	Gene Name	EntrezGene	Ensembl
1 ☐	P39023	NA	RPL3	ribosomal protein L3	6122	ENSG00000100316
2 ☐	P42766	NA	RPL35	ribosomal protein L35	11224	ENSG00000136942
3 ☐	P27635	NA	RPL10	ribosomal protein L10	6134	ENSG00000147403
4 ☐	P84098	NA	RPL19	ribosomal protein L19	6143	ENSG00000108298
5 ☐	Q02543	NA	RPL18A	ribosomal protein L18a	6142	ENSG00000105640
6 ☐	P40429	NA	RPL13A	ribosomal protein L13a	23521	ENSG00000142541
7 ☐	P49207	NA	RPL34	ribosomal protein L34	6164	ENSG00000109475
8 ☐	Q9Y3U8	NA	RPL36	ribosomal protein L36	25873	ENSG00000130255

Database:cellular component			Name:cytosolic ribosome		ID:GO:0022626	
C=91; O=8; E=0.28; R=28.76; rawP=3.01e-10; adjP=7.90e-09						
Index	UserID	Value	Gene Symbol	Gene Name	EntrezGene	Ensembl
1 ☐	P39023	NA	RPL3	ribosomal protein L3	6122	ENSG00000100316
2 ☐	P42766	NA	RPL35	ribosomal protein L35	11224	ENSG00000136942
3 ☐	P27635	NA	RPL10	ribosomal protein L10	6134	ENSG00000147403
4 ☐	P84098	NA	RPL19	ribosomal protein L19	6143	ENSG00000108298
5 ☐	Q02543	NA	RPL18A	ribosomal protein L18a	6142	ENSG00000105640
6 ☐	P40429	NA	RPL13A	ribosomal protein L13a	23521	ENSG00000142541
7 ☐	P49207	NA	RPL34	ribosomal protein L34	6164	ENSG00000109475
8 ☐	Q9Y3U8	NA	RPL36	ribosomal protein L36	25873	ENSG00000130255

Database:cellular component			Name:ribosomal subunit		ID:GO:0044391	
C=133; O=8; E=0.41; R=19.68; rawP=6.30e-09; adjP=1.32e-07						
Index	UserID	Value	Gene Symbol	Gene Name	EntrezGene	Ensembl

Database:cellular component Name:ribosomal subunit ID:GO:0044391						
C=133; O=8; E=0.41; R=19.68; rawP=6.30e-09; adjP=1.32e-07						
Index	UserID	Value	Gene Symbol	Gene Name	EntrezGene	Ensembl
1 ☐	P39023	NA	RPL3	ribosomal protein L3	6122	ENSG00000100316
2 ☐	P42766	NA	RPL35	ribosomal protein L35	11224	ENSG00000136942
3 ☐	P27635	NA	RPL10	ribosomal protein L10	6134	ENSG00000147403
4 ☐	P84098	NA	RPL19	ribosomal protein L19	6143	ENSG00000108298
5 ☐	Q02543	NA	RPL18A	ribosomal protein L18a	6142	ENSG00000105640
6 ☐	P40429	NA	RPL13A	ribosomal protein L13a	23521	ENSG00000142541
7 ☐	P49207	NA	RPL34	ribosomal protein L34	6164	ENSG00000109475
8 ☐	Q9Y3U8	NA	RPL36	ribosomal protein L36	25873	ENSG00000130255

Database:cellular component Name:ribonucleoprotein complex ID:GO:0030529						
C=544; O=12; E=1.66; R=7.22; rawP=6.37e-08; adjP=1.11e-06						
Index	UserID	Value	Gene Symbol	Gene Name	EntrezGene	Ensembl
1 ☐	P39023	NA	RPL3	ribosomal protein L3	6122	ENSG00000100316
2 ☐	P42766	NA	RPL35	ribosomal protein L35	11224	ENSG00000136942
3 ☐	Q01130	NA	SRSF2	serine/arginine-rich splicing factor 2	6427	ENSG00000161547
4 ☐	P27635	NA	RPL10	ribosomal protein L10	6134	ENSG00000147403
5 ☐	P55795	NA	HNRNPH2	heterogeneous nuclear ribonucleoprotein H2 (H')	3188	ENSG00000126945
6 ☐	P06748	NA	NPM1	nucleophosmin (nucleolar phosphoprotein B23, numatrin)	4869	ENSG00000181163
7 ☐	Q02543	NA	RPL18A	ribosomal protein L18a	6142	ENSG00000105640
8 ☐	P84098	NA	RPL19	ribosomal protein L19	6143	ENSG00000108298
9 ☐	P16989	NA	CSDA	cold shock domain protein A	8531	ENSG00000060138
10 ☐	P40429	NA	RPL13A		23521	ENSG00000142541

Database:cellular component			Name:ribonucleoprotein complex			ID:GO:0030529
C=544; O=12; E=1.66; R=7.22; rawP=6.37e-08; adjP=1.11e-06						
Index	UserID	Value	Gene Symbol	Gene Name	EntrezGene	Ensembl
				ribosomal protein L13a		
11 ☐	Q9Y3U8	NA	RPL36	ribosomal protein L36	25873	ENSG00000130255
12 ☐	P49207	NA	RPL34	ribosomal protein L34	6164	ENSG00000109475

Database:cellular component			Name:intracellular part			ID:GO:0044424
C=12096; O=51; E=36.98; R=1.38; rawP=7.37e-08; adjP=1.11e-06						
Index	UserID	Value	Gene Symbol	Gene Name	EntrezGene	Ensembl
1 ☐	P39023	NA	RPL3	ribosomal protein L3	6122	ENSG00000100316
2 ☐	P09429	NA	HMGB1	high mobility group box 1	3146	ENSG00000189403
3 ☐	P61106	NA	RAB14	RAB14, member RAS oncogene family	51552	ENSG00000119396
4 ☐	P30041	NA	PRDX6	peroxiredoxin 6	9588	ENSG00000117592
5 ☐	P63261	NA	ACTG1	actin, gamma 1	71	ENSG00000184009
6 ☐	P60059	NA	SEC61G	Sec61 gamma subunit	23480	ENSG00000132432
7 ☐	P30086	NA	PEBP1	phosphatidylethanolamine binding protein 1	5037	ENSG00000089220
8 ☐	P61019	NA	RAB2A	RAB2A, member RAS oncogene family	5862	ENSG00000104388
9 ☐	P06748	NA	NPM1	nucleophosmin (nucleolar phosphoprotein B23, numatrin)	4869	ENSG00000181163
10 ☐	P30040	NA	ERP29	endoplasmic reticulum protein 29	10961	ENSG00000089248
11 ☐	Q99497	NA	PARK7	parkinson protein 7	11315	ENSG00000116288
12 ☐	P49588	NA	AARS	alanyl-tRNA synthetase	16	ENSG00000090861
13 ☐	Q13126	NA	MTAP	methylthioadenosine phosphorylase	4507	ENSG00000099810
14 ☐	P40429	NA	RPL13A	ribosomal protein L13a	23521	ENSG00000142541
15 ☐	P49207	NA	RPL34	ribosomal protein L34	6164	ENSG00000109475
16 ☐	P62942	NA	FKBP1A	FK506 binding protein 1A, 12kDa	2280	ENSG00000088832
17 ☐	Q01130	NA	SRSF2	serine/arginine-rich splicing factor 2	6427	ENSG00000161547
18 ☐	Q01581	NA	HMGCS1	3-hydroxy-3-methylglutaryl-CoA synthase 1 (soluble)	3157	ENSG00000112972
19 ☐	Q9UQE7	NA	SMC3		9126	ENSG00000108055

Database:cellular component Name:intracellular part ID:GO:0044424						
C=12096; O=51; E=36.98; R=1.38; rawP=7.37e-08; adjP=1.11e-06						
Index	UserID	Value	Gene Symbol	Gene Name	EntrezGene	Ensembl
				structural maintenance of chromosomes 3		
20 ☐	P27635	NA	RPL10	ribosomal protein L10	6134	ENSG00000147403
21 ☐	P40616	NA	ARL1	ADP-ribosylation factor-like 1	400	ENSG00000120805
22 ☐	P09972	NA	ALDOC	aldolase C, fructose-bisphosphate	230	ENSG00000109107
23 ☐	P41567	NA	EIF1	eukaryotic translation initiation factor 1	10209	ENSG00000173812
24 ☐	Q9H0U4	NA	RAB1B	RAB1B, member RAS oncogene family	81876	ENSG00000174903
25 ☐	O75390	NA	CS	citrate synthase	1431	ENSG00000062485
26 ☐	P15121	NA	AKR1B1	aldo-keto reductase family 1, member B1 (aldose reductase)	231	ENSG00000085662
27 ☐	Q99536	NA	VAT1	vesicle amine transport protein 1 homolog (T. californica)	10493	ENSG00000108828
28 ☐	P84098	NA	RPL19	ribosomal protein L19	6143	ENSG00000108298
29 ☐	P16989	NA	CSDA	cold shock domain protein A	8531	ENSG00000060138
30 ☐	Q96AG4	NA	LRRC59	leucine rich repeat containing 59	55379	ENSG00000108829
31 ☐	Q9Y266	NA	NUDC	nuclear distribution C homolog (A. nidulans)	10726	ENSG00000090273
32 ☐	O43396	NA	TXNL1	thioredoxin-like 1	9352	ENSG00000091164
33 ☐	P42766	NA	RPL35	ribosomal protein L35	11224	ENSG00000136942
34 ☐	Q9HAV7	NA	GRPEL1	GrpE-like 1, mitochondrial (E. coli)	80273	ENSG00000109519
35 ☐	P26447	NA	S100A4	S100 calcium binding protein A4	6275	ENSG00000196154
36 ☐	Q15020	NA	SART3	squamous cell carcinoma antigen recognized by T cells 3	9733	ENSG00000075856
37 ☐	P68036	NA	UBE2L3	ubiquitin-conjugating enzyme E2L 3	7332	ENSG00000185651
38 ☐	Q02543	NA	RPL18A	ribosomal protein L18a	6142	ENSG00000105640
39 ☐	Q14019	NA	COTL1	coactosin-like 1 (Dictyostelium)	23406	ENSG00000103187
40 ☐	P26639	NA	TARS	threonyl-tRNA synthetase	6897	ENSG00000113407
41 ☐	Q9Y281	NA	CFL2	cofilin 2 (muscle)	1073	ENSG00000165410

Database:cellular component Name:intracellular part ID:GO:0044424						
C=12096; O=51; E=36.98; R=1.38; rawP=7.37e-08; adjP=1.11e-06						
Index	UserID	Value	Gene Symbol	Gene Name	EntrezGene	Ensembl
42 ☐	Q9Y3U8	NA	RPL36	ribosomal protein L36	25873	ENSG00000130255
43 ☐	P00558	NA	PGK1	phosphoglycerate kinase 1	5230	ENSG00000102144
44 ☐	O75915	NA	ARL6IP5	ADP-ribosylation-like factor 6 interacting protein 5	10550	ENSG00000144746
45 ☐	P55795	NA	HNRNPH2	heterogeneous nuclear ribonucleoprotein H2 (H')	3188	ENSG00000126945
46 ☐	Q9BTT0	NA	ANP32E	acidic (leucine-rich) nuclear phosphoprotein 32 family, member E	81611	ENSG00000143401
47 ☐	P09493	NA	TPM1	tropomyosin 1 (alpha)	7168	ENSG00000140416
48 ☐	O43747	NA	AP1G1	adaptor-related protein complex 1, gamma 1 subunit	164	ENSG00000166747
49 ☐	Q00577	NA	PURA	purine-rich element binding protein A	5813	ENSG00000185129
50 ☐	Q96AE4	NA	FUBP1	far upstream element (FUSE) binding protein 1	8880	ENSG00000162613
51 ☐	P13693	NA	TPT1	tumor protein, translationally-controlled 1	7178	ENSG00000133112

Database:cellular component Name:cytosol ID:GO:0005829						
C=2367; O=23; E=7.24; R=3.18; rawP=9.86e-08; adjP=1.15e-06						
Index	UserID	Value	Gene Symbol	Gene Name	EntrezGene	Ensembl
1 ☐	P39023	NA	RPL3	ribosomal protein L3	6122	ENSG00000100316
2 ☐	P61106	NA	RAB14	RAB14, member RAS oncogene family	51552	ENSG00000119396
3 ☐	P42766	NA	RPL35	ribosomal protein L35	11224	ENSG00000136942
4 ☐	P30041	NA	PRDX6	peroxiredoxin 6	9588	ENSG00000117592
5 ☐	P63261	NA	ACTG1	actin, gamma 1	71	ENSG00000184009
6 ☐	Q02543	NA	RPL18A	ribosomal protein L18a	6142	ENSG00000105640
7 ☐	P49588	NA	AARS	alanyl-tRNA synthetase	16	ENSG00000090861
8 ☐	Q99497	NA	PARK7	parkinson protein 7	11315	ENSG00000116288
9 ☐	P26639	NA	TARS	threonyl-tRNA synthetase	6897	ENSG00000113407
10 ☐	P40429	NA	RPL13A		23521	ENSG00000142541

Database:cellular component Name:cytosol ID:GO:0005829						
C=2367; O=23; E=7.24; R=3.18; rawP=9.86e-08; adjP=1.15e-06						
Index	UserID	Value	Gene Symbol	Gene Name	EntrezGene	Ensembl
				ribosomal protein L13a		
11 ☐	Q13126	NA	MTAP	methylthioadenosine phosphorylase	4507	ENSG00000099810
12 ☐	Q9Y3U8	NA	RPL36	ribosomal protein L36	25873	ENSG00000130255
13 ☐	P49207	NA	RPL34	ribosomal protein L34	6164	ENSG00000109475
14 ☐	P62942	NA	FKBP1A	FK506 binding protein 1A, 12kDa	2280	ENSG00000088832
15 ☐	Q01581	NA	HMGCS1	3-hydroxy-3-methylglutaryl-CoA synthase 1 (soluble)	3157	ENSG00000112972
16 ☐	P27635	NA	RPL10	ribosomal protein L10	6134	ENSG00000147403
17 ☐	P00558	NA	PGK1	phosphoglycerate kinase 1	5230	ENSG00000102144
18 ☐	P09972	NA	ALDOC	aldolase C, fructose-bisphosphate	230	ENSG00000109107
19 ☐	P15121	NA	AKR1B1	aldo-keto reductase family 1, member B1 (aldose reductase)	231	ENSG00000085662
20 ☐	P84098	NA	RPL19	ribosomal protein L19	6143	ENSG00000108298
21 ☐	P09493	NA	TPM1	tropomyosin 1 (alpha)	7168	ENSG00000140416
22 ☐	O43747	NA	AP1G1	adaptor-related protein complex 1, gamma 1 subunit	164	ENSG00000166747
23 ☐	Q9Y266	NA	NUDC	nuclear distribution C homolog (A. nidulans)	10726	ENSG00000090273

Database:cellular component Name:cytosolic part ID:GO:0044445						
C=187; O=8; E=0.57; R=13.99; rawP=9.05e-08; adjP=1.15e-06						
Index	UserID	Value	Gene Symbol	Gene Name	EntrezGene	Ensembl
1 ☐	P39023	NA	RPL3	ribosomal protein L3	6122	ENSG00000100316
2 ☐	P42766	NA	RPL35	ribosomal protein L35	11224	ENSG00000136942
3 ☐	P27635	NA	RPL10	ribosomal protein L10	6134	ENSG00000147403
4 ☐	P84098	NA	RPL19		6143	ENSG00000108298

Database:cellular component Name:cytosolic part ID:GO:0044445						
C=187; O=8; E=0.57; R=13.99; rawP=9.05e-08; adjP=1.15e-06						
Index	UserID	Value	Gene Symbol	Gene Name	EntrezGene	Ensembl
				ribosomal protein L19		
5 ☐	Q02543	NA	RPL18A	ribosomal protein L18a	6142	ENSG00000105640
6 ☐	P40429	NA	RPL13A	ribosomal protein L13a	23521	ENSG00000142541
7 ☐	P49207	NA	RPL34	ribosomal protein L34	6164	ENSG00000109475
8 ☐	Q9Y3U8	NA	RPL36	ribosomal protein L36	25873	ENSG00000130255

Database:cellular component Name:ribosome ID:GO:0005840						
C=198; O=8; E=0.61; R=13.22; rawP=1.41e-07; adjP=1.48e-06						
Index	UserID	Value	Gene Symbol	Gene Name	EntrezGene	Ensembl
1 ☐	P39023	NA	RPL3	ribosomal protein L3	6122	ENSG00000100316
2 ☐	P42766	NA	RPL35	ribosomal protein L35	11224	ENSG00000136942
3 ☐	P27635	NA	RPL10	ribosomal protein L10	6134	ENSG00000147403
4 ☐	P84098	NA	RPL19	ribosomal protein L19	6143	ENSG00000108298
5 ☐	Q02543	NA	RPL18A	ribosomal protein L18a	6142	ENSG00000105640
6 ☐	P40429	NA	RPL13A	ribosomal protein L13a	23521	ENSG00000142541
7 ☐	P49207	NA	RPL34	ribosomal protein L34	6164	ENSG00000109475
8 ☐	Q9Y3U8	NA	RPL36	ribosomal protein L36	25873	ENSG00000130255

Database:cellular component Name:intracellular organelle ID:GO:0043229						
C=10521; O=48; E=32.16; R=1.49; rawP=2.78e-07; adjP=2.39e-06						
Index	UserID	Value	Gene Symbol	Gene Name	EntrezGene	Ensembl
1 ☐	P39023	NA	RPL3	ribosomal protein L3	6122	ENSG00000100316
2 ☐	P09429	NA	HMGB1	high mobility group box 1	3146	ENSG00000189403
3 ☐	P61106	NA	RAB14	RAB14, member RAS oncogene family	51552	ENSG00000119396
4 ☐	P30041	NA	PRDX6	peroxiredoxin 6	9588	ENSG00000117592
5 ☐	P63261	NA	ACTG1	actin, gamma 1	71	ENSG00000184009

Database:cellular component			Name:intracellular organelle			ID:GO:0043229
C=10521; O=48; E=32.16; R=1.49; rawP=2.78e-07; adjP=2.39e-06						
Index	UserID	Value	Gene Symbol	Gene Name	EntrezGene	Ensembl
6 ☐	P60059	NA	SEC61G	Sec61 gamma subunit	23480	ENSG00000132432
7 ☐	P30086	NA	PEBP1	phosphatidylethanolamine binding protein 1	5037	ENSG00000089220
8 ☐	P61019	NA	RAB2A	RAB2A, member RAS oncogene family	5862	ENSG00000104388
9 ☐	P06748	NA	NPM1	nucleophosmin (nucleolar phosphoprotein B23, numatrin)	4869	ENSG00000181163
10 ☐	P30040	NA	ERP29	endoplasmic reticulum protein 29	10961	ENSG00000089248
11 ☐	Q99497	NA	PARK7	parkinson protein 7	11315	ENSG00000116288
12 ☐	Q13126	NA	MTAP	methylthioadenosine phosphorylase	4507	ENSG00000099810
13 ☐	P40429	NA	RPL13A	ribosomal protein L13a	23521	ENSG00000142541
14 ☐	P49207	NA	RPL34	ribosomal protein L34	6164	ENSG00000109475
15 ☐	P62942	NA	FKBP1A	FK506 binding protein 1A, 12kDa	2280	ENSG00000088832
16 ☐	Q01130	NA	SRSF2	serine/arginine-rich splicing factor 2	6427	ENSG00000161547
17 ☐	Q9UQE7	NA	SMC3	structural maintenance of chromosomes 3	9126	ENSG00000108055
18 ☐	Q01581	NA	HMGCS1	3-hydroxy-3-methylglutaryl-CoA synthase 1 (soluble)	3157	ENSG00000112972
19 ☐	P27635	NA	RPL10	ribosomal protein L10	6134	ENSG00000147403
20 ☐	P40616	NA	ARL1	ADP-ribosylation factor-like 1	400	ENSG00000120805
21 ☐	P09972	NA	ALDOC	aldolase C, fructose-bisphosphate	230	ENSG00000109107
22 ☐	Q9H0U4	NA	RAB1B	RAB1B, member RAS oncogene family	81876	ENSG00000174903
23 ☐	O75390	NA	CS	citrate synthase	1431	ENSG00000062485
24 ☐	P15121	NA	AKR1B1	aldo-keto reductase family 1, member B1 (aldose reductase)	231	ENSG00000085662
25 ☐	Q99536	NA	VAT1	vesicle amine transport protein 1 homolog (T. californica)	10493	ENSG00000108828
26 ☐	P84098	NA	RPL19	ribosomal protein L19	6143	ENSG00000108298
27 ☐	P16989	NA	CSDA	cold shock domain protein A	8531	ENSG00000060138
28 ☐	Q96AG4	NA	LRRC59		55379	ENSG00000108829

Database:cellular component			Name:intracellular organelle		ID:GO:0043229	
C=10521; O=48; E=32.16; R=1.49; rawP=2.78e-07; adjP=2.39e-06						
Index	UserID	Value	Gene Symbol	Gene Name	EntrezGene	Ensembl
				leucine rich repeat containing 59		
29 ☐	Q9Y266	NA	NUDC	nuclear distribution C homolog (A. nidulans)	10726	ENSG00000090273
30 ☐	O43396	NA	TXNL1	thioredoxin-like 1	9352	ENSG00000091164
31 ☐	P42766	NA	RPL35	ribosomal protein L35	11224	ENSG00000136942
32 ☐	Q9HAV7	NA	GRPEL1	GrpE-like 1, mitochondrial (E. coli)	80273	ENSG00000109519
33 ☐	P26447	NA	S100A4	S100 calcium binding protein A4	6275	ENSG00000196154
34 ☐	Q15020	NA	SART3	squamous cell carcinoma antigen recognized by T cells 3	9733	ENSG00000075856
35 ☐	P68036	NA	UBE2L3	ubiquitin-conjugating enzyme E2L 3	7332	ENSG00000185651
36 ☐	Q02543	NA	RPL18A	ribosomal protein L18a	6142	ENSG00000105640
37 ☐	Q14019	NA	COTL1	coactosin-like 1 (Dictyostelium)	23406	ENSG00000103187
38 ☐	P26639	NA	TARS	threonyl-tRNA synthetase	6897	ENSG00000113407
39 ☐	Q9Y281	NA	CFL2	cofilin 2 (muscle)	1073	ENSG00000165410
40 ☐	Q9Y3U8	NA	RPL36	ribosomal protein L36	25873	ENSG00000130255
41 ☐	O75915	NA	ARL6IP5	ADP-ribosylation-like factor 6 interacting protein 5	10550	ENSG00000144746
42 ☐	P55795	NA	HNRNPH2	heterogeneous nuclear ribonucleoprotein H2 (H')	3188	ENSG00000126945
43 ☐	Q9BTT0	NA	ANP32E	acidic (leucine-rich) nuclear phosphoprotein 32 family, member E	81611	ENSG00000143401
44 ☐	P09493	NA	TPM1	tropomyosin 1 (alpha)	7168	ENSG00000140416
45 ☐	O43747	NA	AP1G1	adaptor-related protein complex 1, gamma 1 subunit	164	ENSG00000166747
46 ☐	Q00577	NA	PURA	purine-rich element binding protein A	5813	ENSG00000185129
47 ☐	Q96AE4	NA	FUBP1	far upstream element (FUSE) binding protein 1	8880	ENSG00000162613
48 ☐	P13693	NA	TPT1	tumor protein, translationally-controlled 1	7178	ENSG00000133112

Database:cellular component Name:intracellular ID:GO:0005622						
C=12412; O=51; E=37.95; R=1.34; rawP=2.75e-07; adjP=2.39e-06						
Index	UserID	Value	Gene Symbol	Gene Name	EntrezGene	Ensembl
1 ☐	P39023	NA	RPL3	ribosomal protein L3	6122	ENSG00000100316
2 ☐	P09429	NA	HMGB1	high mobility group box 1	3146	ENSG00000189403
3 ☐	P61106	NA	RAB14	RAB14, member RAS oncogene family	51552	ENSG00000119396
4 ☐	P30041	NA	PRDX6	peroxiredoxin 6	9588	ENSG00000117592
5 ☐	P63261	NA	ACTG1	actin, gamma 1	71	ENSG00000184009
6 ☐	P60059	NA	SEC61G	Sec61 gamma subunit	23480	ENSG00000132432
7 ☐	P30086	NA	PEBP1	phosphatidylethanolamine binding protein 1	5037	ENSG00000089220
8 ☐	P61019	NA	RAB2A	RAB2A, member RAS oncogene family	5862	ENSG00000104388
9 ☐	P06748	NA	NPM1	nucleophosmin (nucleolar phosphoprotein B23, numatrin)	4869	ENSG00000181163
10 ☐	P30040	NA	ERP29	endoplasmic reticulum protein 29	10961	ENSG00000089248
11 ☐	Q99497	NA	PARK7	parkinson protein 7	11315	ENSG00000116288
12 ☐	P49588	NA	AARS	alanyl-tRNA synthetase	16	ENSG00000090861
13 ☐	Q13126	NA	MTAP	methylthioadenosine phosphorylase	4507	ENSG00000099810
14 ☐	P40429	NA	RPL13A	ribosomal protein L13a	23521	ENSG00000142541
15 ☐	P49207	NA	RPL34	ribosomal protein L34	6164	ENSG00000109475
16 ☐	P62942	NA	FKBP1A	FK506 binding protein 1A, 12kDa	2280	ENSG00000088832
17 ☐	Q01130	NA	SRSF2	serine/arginine-rich splicing factor 2	6427	ENSG00000161547
18 ☐	Q01581	NA	HMGCS1	3-hydroxy-3-methylglutaryl-CoA synthase 1 (soluble)	3157	ENSG00000112972
19 ☐	Q9UQE7	NA	SMC3	structural maintenance of chromosomes 3	9126	ENSG00000108055
20 ☐	P27635	NA	RPL10	ribosomal protein L10	6134	ENSG00000147403
21 ☐	P40616	NA	ARL1	ADP-ribosylation factor-like 1	400	ENSG00000120805
22 ☐	P09972	NA	ALDOC	aldolase C, fructose-bisphosphate	230	ENSG00000109107
23 ☐	P41567	NA	EIF1	eukaryotic translation initiation factor 1	10209	ENSG00000173812
24 ☐	Q9H0U4	NA	RAB1B	RAB1B, member RAS oncogene family	81876	ENSG00000174903

Database:cellular component Name:intracellular ID:GO:0005622						
C=12412; O=51; E=37.95; R=1.34; rawP=2.75e-07; adjP=2.39e-06						
Index	UserID	Value	Gene Symbol	Gene Name	EntrezGene	Ensembl
25 ☐	O75390	NA	CS	citrate synthase	1431	ENSG00000062485
26 ☐	P15121	NA	AKR1B1	aldo-keto reductase family 1, member B1 (aldose reductase)	231	ENSG00000085662
27 ☐	Q99536	NA	VAT1	vesicle amine transport protein 1 homolog (T. californica)	10493	ENSG00000108828
28 ☐	P84098	NA	RPL19	ribosomal protein L19	6143	ENSG00000108298
29 ☐	P16989	NA	CSDA	cold shock domain protein A	8531	ENSG00000060138
30 ☐	Q96AG4	NA	LRRC59	leucine rich repeat containing 59	55379	ENSG00000108829
31 ☐	Q9Y266	NA	NUDC	nuclear distribution C homolog (A. nidulans)	10726	ENSG00000090273
32 ☐	O43396	NA	TXNL1	thioredoxin-like 1	9352	ENSG00000091164
33 ☐	P42766	NA	RPL35	ribosomal protein L35	11224	ENSG00000136942
34 ☐	Q9HAV7	NA	GRPEL1	GrpE-like 1, mitochondrial (E. coli)	80273	ENSG00000109519
35 ☐	P26447	NA	S100A4	S100 calcium binding protein A4	6275	ENSG00000196154
36 ☐	Q15020	NA	SART3	squamous cell carcinoma antigen recognized by T cells 3	9733	ENSG00000075856
37 ☐	P68036	NA	UBE2L3	ubiquitin-conjugating enzyme E2L 3	7332	ENSG00000185651
38 ☐	Q02543	NA	RPL18A	ribosomal protein L18a	6142	ENSG00000105640
39 ☐	Q14019	NA	COTL1	coactosin-like 1 (Dictyostelium)	23406	ENSG00000103187
40 ☐	P26639	NA	TARS	threonyl-tRNA synthetase	6897	ENSG00000113407
41 ☐	Q9Y281	NA	CFL2	cofilin 2 (muscle)	1073	ENSG00000165410
42 ☐	Q9Y3U8	NA	RPL36	ribosomal protein L36	25873	ENSG00000130255
43 ☐	P00558	NA	PGK1	phosphoglycerate kinase 1	5230	ENSG00000102144
44 ☐	O75915	NA	ARL6IP5	ADP-ribosylation-like factor 6 interacting protein 5	10550	ENSG00000144746
45 ☐	P55795	NA	HNRNPH2	heterogeneous nuclear ribonucleoprotein H2 (H')	3188	ENSG00000126945
46 ☐	Q9BTT0	NA	ANP32E	acidic (leucine-rich) nuclear phosphoprotein 32 family, member E	81611	ENSG00000143401
47 ☐	P09493	NA	TPM1	tropomyosin 1 (alpha)	7168	ENSG00000140416

Database:cellular component Name:intracellular ID:GO:0005622						
C=12412; O=51; E=37.95; R=1.34; rawP=2.75e-07; adjP=2.39e-06						
Index	UserID	Value	Gene Symbol	Gene Name	EntrezGene	Ensembl
48 ☐	O43747	NA	AP1G1	adaptor-related protein complex 1, gamma 1 subunit	164	ENSG00000166747
49 ☐	Q00577	NA	PURA	purine-rich element binding protein A	5813	ENSG00000185129
50 ☐	Q96AE4	NA	FUBP1	far upstream element (FUSE) binding protein 1	8880	ENSG00000162613
51 ☐	P13693	NA	TPT1	tumor protein, translationally-controlled 1	7178	ENSG00000133112

Database:cellular component Name:organelle ID:GO:0043226						
C=10536; O=48; E=32.21; R=1.49; rawP=2.96e-07; adjP=2.39e-06						
Index	UserID	Value	Gene Symbol	Gene Name	EntrezGene	Ensembl
1 ☐	P39023	NA	RPL3	ribosomal protein L3	6122	ENSG00000100316
2 ☐	P09429	NA	HMGB1	high mobility group box 1	3146	ENSG00000189403
3 ☐	P61106	NA	RAB14	RAB14, member RAS oncogene family	51552	ENSG00000119396
4 ☐	P30041	NA	PRDX6	peroxiredoxin 6	9588	ENSG00000117592
5 ☐	P63261	NA	ACTG1	actin, gamma 1	71	ENSG00000184009
6 ☐	P60059	NA	SEC61G	Sec61 gamma subunit	23480	ENSG00000132432
7 ☐	P30086	NA	PEBP1	phosphatidylethanolamine binding protein 1	5037	ENSG00000089220
8 ☐	P61019	NA	RAB2A	RAB2A, member RAS oncogene family	5862	ENSG00000104388
9 ☐	P06748	NA	NPM1	nucleophosmin (nucleolar phosphoprotein B23, numatrin)	4869	ENSG00000181163
10 ☐	P30040	NA	ERP29	endoplasmic reticulum protein 29	10961	ENSG00000089248
11 ☐	Q99497	NA	PARK7	parkinson protein 7	11315	ENSG00000116288
12 ☐	Q13126	NA	MTAP	methylthioadenosine phosphorylase	4507	ENSG00000099810
13 ☐	P40429	NA	RPL13A	ribosomal protein L13a	23521	ENSG00000142541
14 ☐	P49207	NA	RPL34	ribosomal protein L34	6164	ENSG00000109475
15 ☐	P62942	NA	FKBP1A	FK506 binding protein 1A, 12kDa	2280	ENSG00000088832
16 ☐	Q01130	NA	SRSF2	serine/arginine-rich splicing factor 2	6427	ENSG00000161547
17 ☐	Q9UQE7	NA	SMC3	structural maintenance of chromosomes 3	9126	ENSG00000108055

Database:cellular component Name:organelle ID:GO:0043226						
C=10536; O=48; E=32.21; R=1.49; rawP=2.96e-07; adjP=2.39e-06						
Index	UserID	Value	Gene Symbol	Gene Name	EntrezGene	Ensembl
18 ☐	Q01581	NA	HMGCS1	3-hydroxy-3-methylglutaryl-CoA synthase 1 (soluble)	3157	ENSG00000112972
19 ☐	P27635	NA	RPL10	ribosomal protein L10	6134	ENSG00000147403
20 ☐	P40616	NA	ARL1	ADP-ribosylation factor-like 1	400	ENSG00000120805
21 ☐	P09972	NA	ALDOC	aldolase C, fructose-bisphosphate	230	ENSG00000109107
22 ☐	Q9H0U4	NA	RAB1B	RAB1B, member RAS oncogene family	81876	ENSG00000174903
23 ☐	O75390	NA	CS	citrate synthase	1431	ENSG00000062485
24 ☐	P15121	NA	AKR1B1	aldo-keto reductase family 1, member B1 (aldose reductase)	231	ENSG00000085662
25 ☐	Q99536	NA	VAT1	vesicle amine transport protein 1 homolog (T. californica)	10493	ENSG00000108828
26 ☐	P84098	NA	RPL19	ribosomal protein L19	6143	ENSG00000108298
27 ☐	P16989	NA	CSDA	cold shock domain protein A	8531	ENSG00000060138
28 ☐	Q96AG4	NA	LRRC59	leucine rich repeat containing 59	55379	ENSG00000108829
29 ☐	Q9Y266	NA	NUDC	nuclear distribution C homolog (A. nidulans)	10726	ENSG00000090273
30 ☐	O43396	NA	TXNL1	thioredoxin-like 1	9352	ENSG00000091164
31 ☐	P42766	NA	RPL35	ribosomal protein L35	11224	ENSG00000136942
32 ☐	Q9HAV7	NA	GRPEL1	GrpE-like 1, mitochondrial (E. coli)	80273	ENSG00000109519
33 ☐	P26447	NA	S100A4	S100 calcium binding protein A4	6275	ENSG00000196154
34 ☐	Q15020	NA	SART3	squamous cell carcinoma antigen recognized by T cells 3	9733	ENSG00000075856
35 ☐	P68036	NA	UBE2L3	ubiquitin-conjugating enzyme E2L 3	7332	ENSG00000185651
36 ☐	Q02543	NA	RPL18A	ribosomal protein L18a	6142	ENSG00000105640
37 ☐	Q14019	NA	COTL1	coactosin-like 1 (Dictyostelium)	23406	ENSG00000103187
38 ☐	P26639	NA	TARS	threonyl-tRNA synthetase	6897	ENSG00000113407
39 ☐	Q9Y281	NA	CFL2	cofilin 2 (muscle)	1073	ENSG00000165410
40 ☐	Q9Y3U8	NA	RPL36	ribosomal protein L36	25873	ENSG00000130255

Database:cellular component Name:organelle ID:GO:0043226						
C=10536; O=48; E=32.21; R=1.49; rawP=2.96e-07; adjP=2.39e-06						
Index	UserID	Value	Gene Symbol	Gene Name	EntrezGene	Ensembl
41 ☐	O75915	NA	ARL6IP5	ADP-ribosylation-like factor 6 interacting protein 5	10550	ENSG00000144746
42 ☐	P55795	NA	HNRNPH2	heterogeneous nuclear ribonucleoprotein H2 (H')	3188	ENSG00000126945
43 ☐	Q9BTT0	NA	ANP32E	acidic (leucine-rich) nuclear phosphoprotein 32 family, member E	81611	ENSG00000143401
44 ☐	P09493	NA	TPM1	tropomyosin 1 (alpha)	7168	ENSG00000140416
45 ☐	O43747	NA	AP1G1	adaptor-related protein complex 1, gamma 1 subunit	164	ENSG00000166747
46 ☐	Q00577	NA	PURA	purine-rich element binding protein A	5813	ENSG00000185129
47 ☐	Q96AE4	NA	FUBP1	far upstream element (FUSE) binding protein 1	8880	ENSG00000162613
48 ☐	P13693	NA	TPT1	tumor protein, translationally-controlled 1	7178	ENSG00000133112

Database:cellular component Name:cytoplasmic part ID:GO:0044444						
C=6728; O=38; E=20.57; R=1.85; rawP=7.38e-07; adjP=5.54e-06						
Index	UserID	Value	Gene Symbol	Gene Name	EntrezGene	Ensembl
1 ☐	P39023	NA	RPL3	ribosomal protein L3	6122	ENSG00000100316
2 ☐	P61106	NA	RAB14	RAB14, member RAS oncogene family	51552	ENSG00000119396
3 ☐	P30041	NA	PRDX6	peroxiredoxin 6	9588	ENSG00000117592
4 ☐	P63261	NA	ACTG1	actin, gamma 1	71	ENSG00000184009
5 ☐	P60059	NA	SEC61G	Sec61 gamma subunit	23480	ENSG00000132432
6 ☐	P30086	NA	PEBP1	phosphatidylethanolamine binding protein 1	5037	ENSG00000089220
7 ☐	P06748	NA	NPM1	nucleophosmin (nucleolar phosphoprotein B23, numatrin)	4869	ENSG00000181163
8 ☐	P61019	NA	RAB2A	RAB2A, member RAS oncogene family	5862	ENSG00000104388
9 ☐	P30040	NA	ERP29	endoplasmic reticulum protein 29	10961	ENSG00000089248
10 ☐	Q99497	NA	PARK7	parkinson protein 7	11315	ENSG00000116288
11 ☐	P49588	NA	AARS	alanyl-tRNA synthetase	16	ENSG00000090861
12 ☐	Q13126	NA	MTAP	methylthioadenosine phosphorylase	4507	ENSG00000099810

Database:cellular component Name:cytoplasmic part ID:GO:0044444						
C=6728; O=38; E=20.57; R=1.85; rawP=7.38e-07; adjP=5.54e-06						
Index	UserID	Value	Gene Symbol	Gene Name	EntrezGene	Ensembl
13 ☐	P40429	NA	RPL13A	ribosomal protein L13a	23521	ENSG00000142541
14 ☐	P49207	NA	RPL34	ribosomal protein L34	6164	ENSG00000109475
15 ☐	P62942	NA	FKBP1A	FK506 binding protein 1A, 12kDa	2280	ENSG00000088832
16 ☐	Q01581	NA	HMGCS1	3-hydroxy-3-methylglutaryl-CoA synthase 1 (soluble)	3157	ENSG00000112972
17 ☐	P27635	NA	RPL10	ribosomal protein L10	6134	ENSG00000147403
18 ☐	P40616	NA	ARL1	ADP-ribosylation factor-like 1	400	ENSG00000120805
19 ☐	P09972	NA	ALDOC	aldolase C, fructose-bisphosphate	230	ENSG00000109107
20 ☐	Q9H0U4	NA	RAB1B	RAB1B, member RAS oncogene family	81876	ENSG00000174903
21 ☐	O75390	NA	CS	citrate synthase	1431	ENSG00000062485
22 ☐	P15121	NA	AKR1B1	aldo-keto reductase family 1, member B1 (aldose reductase)	231	ENSG00000085662
23 ☐	Q99536	NA	VAT1	vesicle amine transport protein 1 homolog (T. californica)	10493	ENSG00000108828
24 ☐	P84098	NA	RPL19	ribosomal protein L19	6143	ENSG00000108298
25 ☐	Q96AG4	NA	LRRC59	leucine rich repeat containing 59	55379	ENSG00000108829
26 ☐	Q9Y266	NA	NUDC	nuclear distribution C homolog (A. nidulans)	10726	ENSG00000090273
27 ☐	P42766	NA	RPL35	ribosomal protein L35	11224	ENSG00000136942
28 ☐	Q9HAV7	NA	GRPEL1	GrpE-like 1, mitochondrial (E. coli)	80273	ENSG00000109519
29 ☐	P26447	NA	S100A4	S100 calcium binding protein A4	6275	ENSG00000196154
30 ☐	Q02543	NA	RPL18A	ribosomal protein L18a	6142	ENSG00000105640
31 ☐	P26639	NA	TARS	threonyl-tRNA synthetase	6897	ENSG00000113407
32 ☐	Q9Y3U8	NA	RPL36	ribosomal protein L36	25873	ENSG00000130255
33 ☐	P00558	NA	PGK1	phosphoglycerate kinase 1	5230	ENSG00000102144
34 ☐	O75915	NA	ARL6IP5	ADP-ribosylation-like factor 6 interacting protein 5	10550	ENSG00000144746
35 ☐	Q9BTT0	NA	ANP32E	acidic (leucine-rich) nuclear phosphoprotein 32 family, member E	81611	ENSG00000143401

Database:cellular component Name:cytoplasmic part ID:GO:0044444						
C=6728; O=38; E=20.57; R=1.85; rawP=7.38e-07; adjP=5.54e-06						
Index	UserID	Value	Gene Symbol	Gene Name	EntrezGene	Ensembl
36 ☐	P09493	NA	TPM1	tropomyosin 1 (alpha)	7168	ENSG00000140416
37 ☐	O43747	NA	AP1G1	adaptor-related protein complex 1, gamma 1 subunit	164	ENSG00000166747
38 ☐	P13693	NA	TPT1	tumor protein, translationally-controlled 1	7178	ENSG00000133112

Database:cellular component Name:intracellular organelle part ID:GO:0044446						
C=6690; O=36; E=20.45; R=1.76; rawP=9.97e-06; adjP=6.98e-05						
Index	UserID	Value	Gene Symbol	Gene Name	EntrezGene	Ensembl
1 ☐	P39023	NA	RPL3	ribosomal protein L3	6122	ENSG00000100316
2 ☐	P09429	NA	HMGB1	high mobility group box 1	3146	ENSG00000189403
3 ☐	P61106	NA	RAB14	RAB14, member RAS oncogene family	51552	ENSG00000119396
4 ☐	P63261	NA	ACTG1	actin, gamma 1	71	ENSG00000184009
5 ☐	P60059	NA	SEC61G	Sec61 gamma subunit	23480	ENSG00000132432
6 ☐	P30086	NA	PEBP1	phosphatidylethanolamine binding protein 1	5037	ENSG00000089220
7 ☐	P06748	NA	NPM1	nucleophosmin (nucleolar phosphoprotein B23, numatrin)	4869	ENSG00000181163
8 ☐	P61019	NA	RAB2A	RAB2A, member RAS oncogene family	5862	ENSG00000104388
9 ☐	P30040	NA	ERP29	endoplasmic reticulum protein 29	10961	ENSG00000089248
10 ☐	P40429	NA	RPL13A	ribosomal protein L13a	23521	ENSG00000142541
11 ☐	P49207	NA	RPL34	ribosomal protein L34	6164	ENSG00000109475
12 ☐	P62942	NA	FKBP1A	FK506 binding protein 1A, 12kDa	2280	ENSG00000088832
13 ☐	Q9UQE7	NA	SMC3	structural maintenance of chromosomes 3	9126	ENSG00000108055
14 ☐	Q01581	NA	HMGCS1	3-hydroxy-3-methylglutaryl-CoA synthase 1 (soluble)	3157	ENSG00000112972
15 ☐	Q01130	NA	SRSF2	serine/arginine-rich splicing factor 2	6427	ENSG00000161547
16 ☐	P27635	NA	RPL10	ribosomal protein L10	6134	ENSG00000147403
17 ☐	P40616	NA	ARL1	ADP-ribosylation factor-like 1	400	ENSG00000120805

Database:cellular component			Name:intracellular organelle part			ID:GO:0044446
C=6690; O=36; E=20.45; R=1.76; rawP=9.97e-06; adjP=6.98e-05						
Index	UserID	Value	Gene Symbol	Gene Name	EntrezGene	Ensembl
18 ☐	O75390	NA	CS	citrate synthase	1431	ENSG00000062485
19 ☐	P15121	NA	AKR1B1	aldo-keto reductase family 1, member B1 (aldose reductase)	231	ENSG00000085662
20 ☐	Q99536	NA	VAT1	vesicle amine transport protein 1 homolog (T. californica)	10493	ENSG00000108828
21 ☐	P84098	NA	RPL19	ribosomal protein L19	6143	ENSG00000108298
22 ☐	Q96AG4	NA	LRRC59	leucine rich repeat containing 59	55379	ENSG00000108829
23 ☐	Q9Y266	NA	NUDC	nuclear distribution C homolog (A. nidulans)	10726	ENSG00000090273
24 ☐	P42766	NA	RPL35	ribosomal protein L35	11224	ENSG00000136942
25 ☐	Q9HAV7	NA	GRPEL1	GrpE-like 1, mitochondrial (E. coli)	80273	ENSG00000109519
26 ☐	Q15020	NA	SART3	squamous cell carcinoma antigen recognized by T cells 3	9733	ENSG00000075856
27 ☐	Q02543	NA	RPL18A	ribosomal protein L18a	6142	ENSG00000105640
28 ☐	Q9Y281	NA	CFL2	cofilin 2 (muscle)	1073	ENSG00000165410
29 ☐	Q9Y3U8	NA	RPL36	ribosomal protein L36	25873	ENSG00000130255
30 ☐	P55795	NA	HNRNPH2	heterogeneous nuclear ribonucleoprotein H2 (H')	3188	ENSG00000126945
31 ☐	O75915	NA	ARL6IP5	ADP-ribosylation-like factor 6 interacting protein 5	10550	ENSG00000144746
32 ☐	P09493	NA	TPM1	tropomyosin 1 (alpha)	7168	ENSG00000140416
33 ☐	O43747	NA	AP1G1	adaptor-related protein complex 1, gamma 1 subunit	164	ENSG00000166747
34 ☐	Q00577	NA	PURA	purine-rich element binding protein A	5813	ENSG00000185129
35 ☐	Q96AE4	NA	FUBP1	far upstream element (FUSE) binding protein 1	8880	ENSG00000162613
36 ☐	P13693	NA	TPT1	tumor protein, translationally-controlled 1	7178	ENSG00000133112

Database:cellular component			Name:organelle part			ID:GO:0044422
C=6777; O=36; E=20.72; R=1.74; rawP=1.40e-05; adjP=9.19e-05						
Index	UserID	Value	Gene Symbol	Gene Name	EntrezGene	Ensembl
1 ☐	P39023	NA	RPL3	ribosomal protein L3	6122	ENSG00000100316

Database:cellular component Name:organelle part ID:GO:0044422						
C=6777; O=36; E=20.72; R=1.74; rawP=1.40e-05; adjP=9.19e-05						
Index	UserID	Value	Gene Symbol	Gene Name	EntrezGene	Ensembl
2 ☐	P09429	NA	HMGB1	high mobility group box 1	3146	ENSG00000189403
3 ☐	P61106	NA	RAB14	RAB14, member RAS oncogene family	51552	ENSG00000119396
4 ☐	P63261	NA	ACTG1	actin, gamma 1	71	ENSG00000184009
5 ☐	P60059	NA	SEC61G	Sec61 gamma subunit	23480	ENSG00000132432
6 ☐	P30086	NA	PEBP1	phosphatidylethanolamine binding protein 1	5037	ENSG00000089220
7 ☐	P06748	NA	NPM1	nucleophosmin (nucleolar phosphoprotein B23, numatrin)	4869	ENSG00000181163
8 ☐	P61019	NA	RAB2A	RAB2A, member RAS oncogene family	5862	ENSG00000104388
9 ☐	P30040	NA	ERP29	endoplasmic reticulum protein 29	10961	ENSG00000089248
10 ☐	P40429	NA	RPL13A	ribosomal protein L13a	23521	ENSG00000142541
11 ☐	P49207	NA	RPL34	ribosomal protein L34	6164	ENSG00000109475
12 ☐	P62942	NA	FKBP1A	FK506 binding protein 1A, 12kDa	2280	ENSG00000088832
13 ☐	Q9UQE7	NA	SMC3	structural maintenance of chromosomes 3	9126	ENSG00000108055
14 ☐	Q01581	NA	HMGCS1	3-hydroxy-3-methylglutaryl-CoA synthase 1 (soluble)	3157	ENSG00000112972
15 ☐	Q01130	NA	SRSF2	serine/arginine-rich splicing factor 2	6427	ENSG00000161547
16 ☐	P27635	NA	RPL10	ribosomal protein L10	6134	ENSG00000147403
17 ☐	P40616	NA	ARL1	ADP-ribosylation factor-like 1	400	ENSG00000120805
18 ☐	O75390	NA	CS	citrate synthase	1431	ENSG00000062485
19 ☐	P15121	NA	AKR1B1	aldo-keto reductase family 1, member B1 (aldose reductase)	231	ENSG00000085662
20 ☐	Q99536	NA	VAT1	vesicle amine transport protein 1 homolog (T. californica)	10493	ENSG00000108828
21 ☐	P84098	NA	RPL19	ribosomal protein L19	6143	ENSG00000108298
22 ☐	Q96AG4	NA	LRRC59	leucine rich repeat containing 59	55379	ENSG00000108829
23 ☐	Q9Y266	NA	NUDC	nuclear distribution C homolog (A. nidulans)	10726	ENSG00000090273
24 ☐	P42766	NA	RPL35	ribosomal protein L35	11224	ENSG00000136942

Database:cellular component Name:organelle part ID:GO:0044422						
C=6777; O=36; E=20.72; R=1.74; rawP=1.40e-05; adjP=9.19e-05						
Index	UserID	Value	Gene Symbol	Gene Name	EntrezGene	Ensembl
25 ☐	Q9HAV7	NA	GRPEL1	GrpE-like 1, mitochondrial (E. coli)	80273	ENSG00000109519
26 ☐	Q15020	NA	SART3	squamous cell carcinoma antigen recognized by T cells 3	9733	ENSG00000075856
27 ☐	Q02543	NA	RPL18A	ribosomal protein L18a	6142	ENSG00000105640
28 ☐	Q9Y281	NA	CFL2	cofilin 2 (muscle)	1073	ENSG00000165410
29 ☐	Q9Y3U8	NA	RPL36	ribosomal protein L36	25873	ENSG00000130255
30 ☐	P55795	NA	HNRNPH2	heterogeneous nuclear ribonucleoprotein H2 (H')	3188	ENSG00000126945
31 ☐	O75915	NA	ARL6IP5	ADP-ribosylation-like factor 6 interacting protein 5	10550	ENSG00000144746
32 ☐	P09493	NA	TPM1	tropomyosin 1 (alpha)	7168	ENSG00000140416
33 ☐	O43747	NA	AP1G1	adaptor-related protein complex 1, gamma 1 subunit	164	ENSG00000166747
34 ☐	Q00577	NA	PURA	purine-rich element binding protein A	5813	ENSG00000185129
35 ☐	Q96AE4	NA	FUBP1	far upstream element (FUSE) binding protein 1	8880	ENSG00000162613
36 ☐	P13693	NA	TPT1	tumor protein, translationally-controlled 1	7178	ENSG00000133112

Database:cellular component Name:intracellular non-membrane-bounded organelle ID:GO:0043232						
C=3778; O=25; E=11.55; R=2.16; rawP=3.18e-05; adjP=0.0002						
Index	UserID	Value	Gene Symbol	Gene Name	EntrezGene	Ensembl
1 ☐	P39023	NA	RPL3	ribosomal protein L3	6122	ENSG00000100316
2 ☐	P09429	NA	HMGB1	high mobility group box 1	3146	ENSG00000189403
3 ☐	P42766	NA	RPL35	ribosomal protein L35	11224	ENSG00000136942
4 ☐	P63261	NA	ACTG1	actin, gamma 1	71	ENSG00000184009
5 ☐	P06748	NA	NPM1	nucleophosmin (nucleolar phosphoprotein B23, numatrin)	4869	ENSG00000181163
6 ☐	Q02543	NA	RPL18A	ribosomal protein L18a	6142	ENSG00000105640
7 ☐	Q14019	NA	COTL1		23406	ENSG00000103187

Database:cellular component Name:intracellular non-membrane-bounded organelle ID:GO:0043232						
C=3778; O=25; E=11.55; R=2.16; rawP=3.18e-05; adjP=0.0002						
Index	UserID	Value	Gene Symbol	Gene Name	EntrezGene	Ensembl
				coactosin-like 1 (Dictyostelium)		
8 ☐	P26639	NA	TARS	threonyl-tRNA synthetase	6897	ENSG00000113407
9 ☐	Q9Y281	NA	CFL2	cofilin 2 (muscle)	1073	ENSG00000165410
10 ☐	P40429	NA	RPL13A	ribosomal protein L13a	23521	ENSG00000142541
11 ☐	Q9Y3U8	NA	RPL36	ribosomal protein L36	25873	ENSG00000130255
12 ☐	P49207	NA	RPL34	ribosomal protein L34	6164	ENSG00000109475
13 ☐	Q9UQE7	NA	SMC3	structural maintenance of chromosomes 3	9126	ENSG00000108055
14 ☐	Q01581	NA	HMGCS1	3-hydroxy-3-methylglutaryl-CoA synthase 1 (soluble)	3157	ENSG00000112972
15 ☐	P27635	NA	RPL10	ribosomal protein L10	6134	ENSG00000147403
16 ☐	P55795	NA	HNRNPH2	heterogeneous nuclear ribonucleoprotein H2 (H')	3188	ENSG00000126945
17 ☐	P09972	NA	ALDOC	aldolase C, fructose-bisphosphate	230	ENSG00000109107
18 ☐	P15121	NA	AKR1B1	aldo-keto reductase family 1, member B1 (aldose reductase)	231	ENSG00000085662
19 ☐	P84098	NA	RPL19	ribosomal protein L19	6143	ENSG00000108298
20 ☐	P09493	NA	TPM1	tropomyosin 1 (alpha)	7168	ENSG00000140416
21 ☐	Q96AG4	NA	LRRC59	leucine rich repeat containing 59	55379	ENSG00000108829
22 ☐	Q00577	NA	PURA	purine-rich element binding protein A	5813	ENSG00000185129
23 ☐	Q96AE4	NA	FUBP1	far upstream element (FUSE) binding protein 1	8880	ENSG00000162613
24 ☐	P13693	NA	TPT1		7178	ENSG00000133112

Database:cellular component Name:intracellular non-membrane-bounded organelle ID:GO:0043232						
C=3778; O=25; E=11.55; R=2.16; rawP=3.18e-05; adjP=0.0002						
Index	UserID	Value	Gene Symbol	Gene Name	EntrezGene	Ensembl
				tumor protein, translationally-controlled 1		
25 ☐	Q9Y266	NA	NUDC	nuclear distribution C homolog (A. nidulans)	10726	ENSG00000090273

Database:cellular component Name:non-membrane-bounded organelle ID:GO:0043228						
C=3778; O=25; E=11.55; R=2.16; rawP=3.18e-05; adjP=0.0002						
Index	UserID	Value	Gene Symbol	Gene Name	EntrezGene	Ensembl
1 ☐	P39023	NA	RPL3	ribosomal protein L3	6122	ENSG00000100316
2 ☐	P09429	NA	HMGB1	high mobility group box 1	3146	ENSG00000189403
3 ☐	P42766	NA	RPL35	ribosomal protein L35	11224	ENSG00000136942
4 ☐	P63261	NA	ACTG1	actin, gamma 1	71	ENSG00000184009
5 ☐	P06748	NA	NPM1	nucleophosmin (nucleolar phosphoprotein B23, numatrin)	4869	ENSG00000181163
6 ☐	Q02543	NA	RPL18A	ribosomal protein L18a	6142	ENSG00000105640
7 ☐	Q14019	NA	COTL1	coactosin-like 1 (Dictyostelium)	23406	ENSG00000103187
8 ☐	P26639	NA	TARS	threonyl-tRNA synthetase	6897	ENSG00000113407
9 ☐	Q9Y281	NA	CFL2	cofilin 2 (muscle)	1073	ENSG00000165410
10 ☐	P40429	NA	RPL13A	ribosomal protein L13a	23521	ENSG00000142541
11 ☐	Q9Y3U8	NA	RPL36	ribosomal protein L36	25873	ENSG00000130255
12 ☐	P49207	NA	RPL34	ribosomal protein L34	6164	ENSG00000109475
13 ☐	Q9UQE7	NA	SMC3	structural maintenance of chromosomes 3	9126	ENSG00000108055
14 ☐	Q01581	NA	HMGCS1	3-hydroxy-3-methylglutaryl-CoA synthase 1 (soluble)	3157	ENSG00000112972
15 ☐	P27635	NA	RPL10		6134	ENSG00000147403

Database:cellular component Name:non-membrane-bounded organelle ID:GO:0043228						
C=3778; O=25; E=11.55; R=2.16; rawP=3.18e-05; adjP=0.0002						
Index	UserID	Value	Gene Symbol	Gene Name	EntrezGene	Ensembl
				ribosomal protein L10		
16 ☐	P55795	NA	HNRNPH2	heterogeneous nuclear ribonucleoprotein H2 (H')	3188	ENSG00000126945
17 ☐	P09972	NA	ALDOC	aldolase C, fructose-bisphosphate	230	ENSG00000109107
18 ☐	P15121	NA	AKR1B1	aldo-keto reductase family 1, member B1 (aldose reductase)	231	ENSG00000085662
19 ☐	P84098	NA	RPL19	ribosomal protein L19	6143	ENSG00000108298
20 ☐	P09493	NA	TPM1	tropomyosin 1 (alpha)	7168	ENSG00000140416
21 ☐	Q96AG4	NA	LRRC59	leucine rich repeat containing 59	55379	ENSG00000108829
22 ☐	Q00577	NA	PURA	purine-rich element binding protein A	5813	ENSG00000185129
23 ☐	Q96AE4	NA	FUBP1	far upstream element (FUSE) binding protein 1	8880	ENSG00000162613
24 ☐	P13693	NA	TPT1	tumor protein, translationally-controlled 1	7178	ENSG00000133112
25 ☐	Q9Y266	NA	NUDC	nuclear distribution C homolog (A. nidulans)	10726	ENSG00000090273

Database:cellular component Name:cell ID:GO:0005623						
C=14406; O=51; E=44.04; R=1.16; rawP=0.0006; adjP=0.0032						
Index	UserID	Value	Gene Symbol	Gene Name	EntrezGene	Ensembl
1 ☐	P39023	NA	RPL3	ribosomal protein L3	6122	ENSG00000100316
2 ☐	P09429	NA	HMGB1	high mobility group box 1	3146	ENSG00000189403
3 ☐	P61106	NA	RAB14	RAB14, member RAS oncogene family	51552	ENSG00000119396
4 ☐	P30041	NA	PRDX6	peroxiredoxin 6	9588	ENSG00000117592
5 ☐	P63261	NA	ACTG1	actin, gamma 1	71	ENSG00000184009

Database:cellular component Name:cell ID:GO:0005623						
C=14406; O=51; E=44.04; R=1.16; rawP=0.0006; adjP=0.0032						
Index	UserID	Value	Gene Symbol	Gene Name	EntrezGene	Ensembl
6 ☐	P60059	NA	SEC61G	Sec61 gamma subunit	23480	ENSG00000132432
7 ☐	P30086	NA	PEBP1	phosphatidylethanolamine binding protein 1	5037	ENSG00000089220
8 ☐	P61019	NA	RAB2A	RAB2A, member RAS oncogene family	5862	ENSG00000104388
9 ☐	P06748	NA	NPM1	nucleophosmin (nucleolar phosphoprotein B23, numatrin)	4869	ENSG00000181163
10 ☐	P30040	NA	ERP29	endoplasmic reticulum protein 29	10961	ENSG00000089248
11 ☐	Q99497	NA	PARK7	parkinson protein 7	11315	ENSG00000116288
12 ☐	P49588	NA	AARS	alanyl-tRNA synthetase	16	ENSG00000090861
13 ☐	Q13126	NA	MTAP	methylthioadenosine phosphorylase	4507	ENSG00000099810
14 ☐	P40429	NA	RPL13A	ribosomal protein L13a	23521	ENSG00000142541
15 ☐	P49207	NA	RPL34	ribosomal protein L34	6164	ENSG00000109475
16 ☐	P62942	NA	FKBP1A	FK506 binding protein 1A, 12kDa	2280	ENSG00000088832
17 ☐	Q01130	NA	SRSF2	serine/arginine-rich splicing factor 2	6427	ENSG00000161547
18 ☐	Q01581	NA	HMGCS1	3-hydroxy-3-methylglutaryl-CoA synthase 1 (soluble)	3157	ENSG00000112972
19 ☐	Q9UQE7	NA	SMC3	structural maintenance of chromosomes 3	9126	ENSG00000108055
20 ☐	P27635	NA	RPL10	ribosomal protein L10	6134	ENSG00000147403
21 ☐	P40616	NA	ARL1	ADP-ribosylation factor-like 1	400	ENSG00000120805
22 ☐	P09972	NA	ALDOC	aldolase C, fructose-bisphosphate	230	ENSG00000109107
23 ☐	P41567	NA	EIF1	eukaryotic translation initiation factor 1	10209	ENSG00000173812
24 ☐	Q9H0U4	NA	RAB1B	RAB1B, member RAS oncogene family	81876	ENSG00000174903
25 ☐	O75390	NA	CS	citrate synthase	1431	ENSG00000062485
26 ☐	P15121	NA	AKR1B1	aldo-keto reductase family 1, member B1 (aldose reductase)	231	ENSG00000085662
27 ☐	Q99536	NA	VAT1	vesicle amine transport protein 1 homolog (T. californica)	10493	ENSG00000108828
28 ☐	P84098	NA	RPL19	ribosomal protein L19	6143	ENSG00000108298

Database:cellular component Name:cell ID:GO:0005623						
C=14406; O=51; E=44.04; R=1.16; rawP=0.0006; adjP=0.0032						
Index	UserID	Value	Gene Symbol	Gene Name	EntrezGene	Ensembl
29 ☐	P16989	NA	CSDA	cold shock domain protein A	8531	ENSG00000060138
30 ☐	Q96AG4	NA	LRRC59	leucine rich repeat containing 59	55379	ENSG00000108829
31 ☐	Q9Y266	NA	NUDC	nuclear distribution C homolog (A. nidulans)	10726	ENSG00000090273
32 ☐	O43396	NA	TXNL1	thioredoxin-like 1	9352	ENSG00000091164
33 ☐	P42766	NA	RPL35	ribosomal protein L35	11224	ENSG00000136942
34 ☐	Q9HAV7	NA	GRPEL1	GrpE-like 1, mitochondrial (E. coli)	80273	ENSG00000109519
35 ☐	P26447	NA	S100A4	S100 calcium binding protein A4	6275	ENSG00000196154
36 ☐	Q15020	NA	SART3	squamous cell carcinoma antigen recognized by T cells 3	9733	ENSG00000075856
37 ☐	P68036	NA	UBE2L3	ubiquitin-conjugating enzyme E2L 3	7332	ENSG00000185651
38 ☐	Q02543	NA	RPL18A	ribosomal protein L18a	6142	ENSG00000105640
39 ☐	Q14019	NA	COTL1	coactosin-like 1 (Dictyostelium)	23406	ENSG00000103187
40 ☐	P26639	NA	TARS	threonyl-tRNA synthetase	6897	ENSG00000113407
41 ☐	Q9Y281	NA	CFL2	cofilin 2 (muscle)	1073	ENSG00000165410
42 ☐	Q9Y3U8	NA	RPL36	ribosomal protein L36	25873	ENSG00000130255
43 ☐	P00558	NA	PGK1	phosphoglycerate kinase 1	5230	ENSG00000102144
44 ☐	O75915	NA	ARL6IP5	ADP-ribosylation-like factor 6 interacting protein 5	10550	ENSG00000144746
45 ☐	P55795	NA	HNRNPH2	heterogeneous nuclear ribonucleoprotein H2 (H')	3188	ENSG00000126945
46 ☐	Q9BTT0	NA	ANP32E	acidic (leucine-rich) nuclear phosphoprotein 32 family, member E	81611	ENSG00000143401
47 ☐	P09493	NA	TPM1	tropomyosin 1 (alpha)	7168	ENSG00000140416
48 ☐	O43747	NA	AP1G1	adaptor-related protein complex 1, gamma 1 subunit	164	ENSG00000166747
49 ☐	Q00577	NA	PURA	purine-rich element binding protein A	5813	ENSG00000185129
50 ☐	Q96AE4	NA	FUBP1	far upstream element (FUSE) binding protein 1	8880	ENSG00000162613
51 ☐	P13693	NA	TPT1		7178	ENSG00000133112

Database:cellular component Name:cell ID:GO:0005623						
C=14406; O=51; E=44.04; R=1.16; rawP=0.0006; adjP=0.0032						
Index	UserID	Value	Gene Symbol	Gene Name	EntrezGene	Ensembl
				tumor protein, translationally-controlled 1		

Database:cellular component Name:cell part ID:GO:0044464						
C=14405; O=51; E=44.04; R=1.16; rawP=0.0006; adjP=0.0032						
Index	UserID	Value	Gene Symbol	Gene Name	EntrezGene	Ensembl
1 ☐	P39023	NA	RPL3	ribosomal protein L3	6122	ENSG00000100316
2 ☐	P09429	NA	HMGB1	high mobility group box 1	3146	ENSG00000189403
3 ☐	P61106	NA	RAB14	RAB14, member RAS oncogene family	51552	ENSG00000119396
4 ☐	P30041	NA	PRDX6	peroxiredoxin 6	9588	ENSG00000117592
5 ☐	P63261	NA	ACTG1	actin, gamma 1	71	ENSG00000184009
6 ☐	P60059	NA	SEC61G	Sec61 gamma subunit	23480	ENSG00000132432
7 ☐	P30086	NA	PEBP1	phosphatidylethanolamine binding protein 1	5037	ENSG00000089220
8 ☐	P61019	NA	RAB2A	RAB2A, member RAS oncogene family	5862	ENSG00000104388
9 ☐	P06748	NA	NPM1	nucleophosmin (nucleolar phosphoprotein B23, numatrin)	4869	ENSG00000181163
10 ☐	P30040	NA	ERP29	endoplasmic reticulum protein 29	10961	ENSG00000089248
11 ☐	Q99497	NA	PARK7	parkinson protein 7	11315	ENSG00000116288
12 ☐	P49588	NA	AARS	alanyl-tRNA synthetase	16	ENSG00000090861
13 ☐	Q13126	NA	MTAP	methylthioadenosine phosphorylase	4507	ENSG00000099810
14 ☐	P40429	NA	RPL13A	ribosomal protein L13a	23521	ENSG00000142541
15 ☐	P49207	NA	RPL34	ribosomal protein L34	6164	ENSG00000109475
16 ☐	P62942	NA	FKBP1A	FK506 binding protein 1A, 12kDa	2280	ENSG00000088832
17 ☐	Q01130	NA	SRSF2	serine/arginine-rich splicing factor 2	6427	ENSG00000161547
18 ☐	Q01581	NA	HMGCS1	3-hydroxy-3-methylglutaryl-CoA synthase 1 (soluble)	3157	ENSG00000112972
19 ☐	Q9UQE7	NA	SMC3	structural maintenance of chromosomes 3	9126	ENSG00000108055
20 ☐	P27635	NA	RPL10	ribosomal protein L10	6134	ENSG00000147403
21 ☐	P40616	NA	ARL1		400	ENSG00000120805

Database:cellular component Name:cell part ID:GO:0044464						
C=14405; O=51; E=44.04; R=1.16; rawP=0.0006; adjP=0.0032						
Index	UserID	Value	Gene Symbol	Gene Name	EntrezGene	Ensembl
				ADP-ribosylation factor-like 1		
22 ☐	P09972	NA	ALDOC	aldolase C, fructose-bisphosphate	230	ENSG00000109107
23 ☐	P41567	NA	EIF1	eukaryotic translation initiation factor 1	10209	ENSG00000173812
24 ☐	Q9H0U4	NA	RAB1B	RAB1B, member RAS oncogene family	81876	ENSG00000174903
25 ☐	O75390	NA	CS	citrate synthase	1431	ENSG00000062485
26 ☐	P15121	NA	AKR1B1	aldo-keto reductase family 1, member B1 (aldose reductase)	231	ENSG00000085662
27 ☐	Q99536	NA	VAT1	vesicle amine transport protein 1 homolog (T. californica)	10493	ENSG00000108828
28 ☐	P84098	NA	RPL19	ribosomal protein L19	6143	ENSG00000108298
29 ☐	P16989	NA	CSDA	cold shock domain protein A	8531	ENSG00000060138
30 ☐	Q96AG4	NA	LRRC59	leucine rich repeat containing 59	55379	ENSG00000108829
31 ☐	Q9Y266	NA	NUDC	nuclear distribution C homolog (A. nidulans)	10726	ENSG00000090273
32 ☐	O43396	NA	TXNL1	thioredoxin-like 1	9352	ENSG00000091164
33 ☐	P42766	NA	RPL35	ribosomal protein L35	11224	ENSG00000136942
34 ☐	Q9HAV7	NA	GRPEL1	GrpE-like 1, mitochondrial (E. coli)	80273	ENSG00000109519
35 ☐	P26447	NA	S100A4	S100 calcium binding protein A4	6275	ENSG00000196154
36 ☐	Q15020	NA	SART3	squamous cell carcinoma antigen recognized by T cells 3	9733	ENSG00000075856
37 ☐	P68036	NA	UBE2L3	ubiquitin-conjugating enzyme E2L 3	7332	ENSG00000185651
38 ☐	Q02543	NA	RPL18A	ribosomal protein L18a	6142	ENSG00000105640
39 ☐	Q14019	NA	COTL1	coactosin-like 1 (Dictyostelium)	23406	ENSG00000103187
40 ☐	P26639	NA	TARS	threonyl-tRNA synthetase	6897	ENSG00000113407
41 ☐	Q9Y281	NA	CFL2	cofilin 2 (muscle)	1073	ENSG00000165410
42 ☐	Q9Y3U8	NA	RPL36	ribosomal protein L36	25873	ENSG00000130255
43 ☐	P00558	NA	PGK1	phosphoglycerate kinase 1	5230	ENSG00000102144

Database:cellular component Name:cell part ID:GO:0044464						
C=14405; O=51; E=44.04; R=1.16; rawP=0.0006; adjP=0.0032						
Index	UserID	Value	Gene Symbol	Gene Name	EntrezGene	Ensembl
44 ☐	O75915	NA	ARL6IP5	ADP-ribosylation-like factor 6 interacting protein 5	10550	ENSG00000144746
45 ☐	P55795	NA	HNRNPH2	heterogeneous nuclear ribonucleoprotein H2 (H')	3188	ENSG00000126945
46 ☐	Q9BTT0	NA	ANP32E	acidic (leucine-rich) nuclear phosphoprotein 32 family, member E	81611	ENSG00000143401
47 ☐	P09493	NA	TPM1	tropomyosin 1 (alpha)	7168	ENSG00000140416
48 ☐	O43747	NA	AP1G1	adaptor-related protein complex 1, gamma 1 subunit	164	ENSG00000166747
49 ☐	Q00577	NA	PURA	purine-rich element binding protein A	5813	ENSG00000185129
50 ☐	Q96AE4	NA	FUBP1	far upstream element (FUSE) binding protein 1	8880	ENSG00000162613
51 ☐	P13693	NA	TPT1	tumor protein, translationally-controlled 1	7178	ENSG00000133112

Database:cellular component Name:neuron projection ID:GO:0043005						
C=651; O=8; E=1.99; R=4.02; rawP=0.0007; adjP=0.0035						
Index	UserID	Value	Gene Symbol	Gene Name	EntrezGene	Ensembl
1 ☐	P62942	NA	FKBP1A	FK506 binding protein 1A, 12kDa	2280	ENSG00000088832
2 ☐	P09429	NA	HMGB1	high mobility group box 1	3146	ENSG00000189403
3 ☐	P63261	NA	ACTG1	actin, gamma 1	71	ENSG00000184009
4 ☐	P26447	NA	S100A4	S100 calcium binding protein A4	6275	ENSG00000196154
5 ☐	P09972	NA	ALDOC	aldolase C, fructose-bisphosphate	230	ENSG00000109107
6 ☐	P30086	NA	PEBP1	phosphatidylethanolamine binding protein 1	5037	ENSG00000089220
7 ☐	Q99497	NA	PARK7	parkinson protein 7	11315	ENSG00000116288
8 ☐	Q00577	NA	PURA	purine-rich element binding protein A	5813	ENSG00000185129

Database:cellular component Name:intracellular organelle lumen ID:GO:0070013						
C=3285; O=20; E=10.04; R=1.99; rawP=0.0010; adjP=0.0048						
Index	UserID	Value	Gene Symbol	Gene Name	EntrezGene	Ensembl

Database:cellular component Name:intracellular organelle lumen ID:GO:0070013						
C=3285; O=20; E=10.04; R=1.99; rawP=0.0010; adjP=0.0048						
Index	UserID	Value	Gene Symbol	Gene Name	EntrezGene	Ensembl
1 ☐	P39023	NA	RPL3	ribosomal protein L3	6122	ENSG00000100316
2 ☐	P09429	NA	HMGB1	high mobility group box 1	3146	ENSG00000189403
3 ☐	P42766	NA	RPL35	ribosomal protein L35	11224	ENSG00000136942
4 ☐	Q9HAV7	NA	GRPEL1	GrpE-like 1, mitochondrial (E. coli)	80273	ENSG00000109519
5 ☐	Q15020	NA	SART3	squamous cell carcinoma antigen recognized by T cells 3	9733	ENSG00000075856
6 ☐	P06748	NA	NPM1	nucleophosmin (nucleolar phosphoprotein B23, numatrin)	4869	ENSG00000181163
7 ☐	P30040	NA	ERP29	endoplasmic reticulum protein 29	10961	ENSG00000089248
8 ☐	Q9Y281	NA	CFL2	cofilin 2 (muscle)	1073	ENSG00000165410
9 ☐	Q9Y3U8	NA	RPL36	ribosomal protein L36	25873	ENSG00000130255
10 ☐	Q9UQE7	NA	SMC3	structural maintenance of chromosomes 3	9126	ENSG00000108055
11 ☐	Q01581	NA	HMGCS1	3-hydroxy-3-methylglutaryl-CoA synthase 1 (soluble)	3157	ENSG00000112972
12 ☐	Q01130	NA	SRSF2	serine/arginine-rich splicing factor 2	6427	ENSG00000161547
13 ☐	P55795	NA	HNRNPH2	heterogeneous nuclear ribonucleoprotein H2 (H')	3188	ENSG00000126945
14 ☐	O75390	NA	CS	citrate synthase	1431	ENSG00000062485
15 ☐	P15121	NA	AKR1B1	aldo-keto reductase family 1, member B1 (aldose reductase)	231	ENSG00000085662
16 ☐	Q96AG4	NA	LRRC59	leucine rich repeat containing 59	55379	ENSG00000108829
17 ☐	Q00577	NA	PURA		5813	ENSG00000185129

Database:cellular component Name:intracellular organelle lumen ID:GO:0070013						
C=3285; O=20; E=10.04; R=1.99; rawP=0.0010; adjP=0.0048						
Index	UserID	Value	Gene Symbol	Gene Name	EntrezGene	Ensembl
				purine-rich element binding protein A		
18 ☐	Q96AE4	NA	FUBP1	far upstream element (FUSE) binding protein 1	8880	ENSG00000162613
19 ☐	P13693	NA	TPT1	tumor protein, translationally-controlled 1	7178	ENSG00000133112
20 ☐	Q9Y266	NA	NUDC	nuclear distribution C homolog (A. nidulans)	10726	ENSG00000090273

Database:cellular component Name:intracellular membrane-bounded organelle ID:GO:0043231						
C=9484; O=40; E=28.99; R=1.38; rawP=0.0011; adjP=0.0048						
Index	UserID	Value	Gene Symbol	Gene Name	EntrezGene	Ensembl
1 ☐	P39023	NA	RPL3	ribosomal protein L3	6122	ENSG00000100316
2 ☐	P09429	NA	HMGB1	high mobility group box 1	3146	ENSG00000189403
3 ☐	P61106	NA	RAB14	RAB14, member RAS oncogene family	51552	ENSG00000119396
4 ☐	P30041	NA	PRDX6	peroxiredoxin 6	9588	ENSG00000117592
5 ☐	P60059	NA	SEC61G	Sec61 gamma subunit	23480	ENSG00000132432
6 ☐	P30086	NA	PEBP1	phosphatidylethanolamine binding protein 1	5037	ENSG00000089220
7 ☐	P06748	NA	NPM1	nucleophosmin (nucleolar phosphoprotein B23, numatrin)	4869	ENSG00000181163
8 ☐	P61019	NA	RAB2A	RAB2A, member RAS oncogene family	5862	ENSG00000104388
9 ☐	P30040	NA	ERP29	endoplasmic reticulum protein 29	10961	ENSG00000089248
10 ☐	Q99497	NA	PARK7	parkinson protein 7	11315	ENSG00000116288
11 ☐	Q13126	NA	MTAP	methylthioadenosine phosphorylase	4507	ENSG00000099810
12 ☐	P62942	NA	FKBP1A	FK506 binding protein 1A, 12kDa	2280	ENSG00000088832
13 ☐	Q9UQE7	NA	SMC3	structural maintenance of chromosomes 3	9126	ENSG00000108055
14 ☐	Q01581	NA	HMGCS1	3-hydroxy-3-methylglutaryl-CoA synthase 1 (soluble)	3157	ENSG00000112972

Database:cellular component organelle			Name:intracellular membrane-bounded ID:GO:0043231			
C=9484; O=40; E=28.99; R=1.38; rawP=0.0011; adjP=0.0048						
Index	UserID	Value	Gene Symbol	Gene Name	EntrezGene	Ensembl
15 ☐	Q01130	NA	SRSF2	serine/arginine-rich splicing factor 2	6427	ENSG00000161547
16 ☐	P27635	NA	RPL10	ribosomal protein L10	6134	ENSG00000147403
17 ☐	P40616	NA	ARL1	ADP-ribosylation factor-like 1	400	ENSG00000120805
18 ☐	P09972	NA	ALDOC	aldolase C, fructose-bisphosphate	230	ENSG00000109107
19 ☐	Q9H0U4	NA	RAB1B	RAB1B, member RAS oncogene family	81876	ENSG00000174903
20 ☐	O75390	NA	CS	citrate synthase	1431	ENSG00000062485
21 ☐	P15121	NA	AKR1B1	aldo-keto reductase family 1, member B1 (aldose reductase)	231	ENSG00000085662
22 ☐	Q99536	NA	VAT1	vesicle amine transport protein 1 homolog (T. californica)	10493	ENSG00000108828
23 ☐	P16989	NA	CSDA	cold shock domain protein A	8531	ENSG00000060138
24 ☐	Q96AG4	NA	LRRC59	leucine rich repeat containing 59	55379	ENSG00000108829
25 ☐	Q9Y266	NA	NUDC	nuclear distribution C homolog (A. nidulans)	10726	ENSG00000090273
26 ☐	O43396	NA	TXNL1	thioredoxin-like 1	9352	ENSG00000091164
27 ☐	P42766	NA	RPL35	ribosomal protein L35	11224	ENSG00000136942
28 ☐	Q9HAV7	NA	GRPEL1	GrpE-like 1, mitochondrial (E. coli)	80273	ENSG00000109519
29 ☐	Q15020	NA	SART3	squamous cell carcinoma antigen recognized by T cells 3	9733	ENSG00000075856
30 ☐	P26447	NA	S100A4	S100 calcium binding protein A4	6275	ENSG00000196154
31 ☐	P68036	NA	UBE2L3	ubiquitin-conjugating enzyme E2L 3	7332	ENSG00000185651
32 ☐	Q9Y281	NA	CFL2	cofilin 2 (muscle)	1073	ENSG00000165410
33 ☐	Q9Y3U8	NA	RPL36	ribosomal protein L36	25873	ENSG00000130255
34 ☐	O75915	NA	ARL6IP5	ADP-ribosylation-like factor 6 interacting protein 5	10550	ENSG00000144746
35 ☐	P55795	NA	HNRNPH2	heterogeneous nuclear ribonucleoprotein H2 (H')	3188	ENSG00000126945
36 ☐	Q9BTT0	NA	ANP32E		81611	ENSG00000143401

Database:cellular component Name:intracellular membrane-bounded organelle ID:GO:0043231						
C=9484; O=40; E=28.99; R=1.38; rawP=0.0011; adjP=0.0048						
Index	UserID	Value	Gene Symbol	Gene Name	EntrezGene	Ensembl
				acidic (leucine-rich) nuclear phosphoprotein 32 family, member E		
37 ☐	O43747	NA	AP1G1	adaptor-related protein complex 1, gamma 1 subunit	164	ENSG00000166747
38 ☐	Q00577	NA	PURA	purine-rich element binding protein A	5813	ENSG00000185129
39 ☐	Q96AE4	NA	FUBP1	far upstream element (FUSE) binding protein 1	8880	ENSG00000162613
40 ☐	P13693	NA	TPT1	tumor protein, translationally-controlled 1	7178	ENSG00000133112

Database:cellular component Name:membrane-bounded organelle ID:GO:0043227						
C=9495; O=40; E=29.03; R=1.38; rawP=0.0011; adjP=0.0048						
Index	UserID	Value	Gene Symbol	Gene Name	EntrezGene	Ensembl
1 ☐	P39023	NA	RPL3	ribosomal protein L3	6122	ENSG00000100316
2 ☐	P09429	NA	HMGB1	high mobility group box 1	3146	ENSG00000189403
3 ☐	P61106	NA	RAB14	RAB14, member RAS oncogene family	51552	ENSG00000119396
4 ☐	P30041	NA	PRDX6	peroxiredoxin 6	9588	ENSG00000117592
5 ☐	P60059	NA	SEC61G	Sec61 gamma subunit	23480	ENSG00000132432
6 ☐	P30086	NA	PEBP1	phosphatidylethanolamine binding protein 1	5037	ENSG00000089220
7 ☐	P06748	NA	NPM1	nucleophosmin (nucleolar phosphoprotein B23, numatrin)	4869	ENSG00000181163
8 ☐	P61019	NA	RAB2A	RAB2A, member RAS oncogene family	5862	ENSG00000104388
9 ☐	P30040	NA	ERP29	endoplasmic reticulum protein 29	10961	ENSG00000089248
10 ☐	Q99497	NA	PARK7	parkinson protein 7	11315	ENSG00000116288
11 ☐	Q13126	NA	MTAP	methylthioadenosine phosphorylase	4507	ENSG00000099810
12 ☐	P62942	NA	FKBP1A	FK506 binding protein 1A, 12kDa	2280	ENSG00000088832
13 ☐	Q9UQE7	NA	SMC3	structural maintenance of chromosomes 3	9126	ENSG00000108055
14 ☐	Q01581	NA	HMGCS1	3-hydroxy-3-methylglutaryl-CoA synthase 1 (soluble)	3157	ENSG00000112972

Database:cellular component			Name:membrane-bounded organelle			ID:GO:0043227
C=9495; O=40; E=29.03; R=1.38; rawP=0.0011; adjP=0.0048						
Index	UserID	Value	Gene Symbol	Gene Name	EntrezGene	Ensembl
15 ☐	Q01130	NA	SRSF2	serine/arginine-rich splicing factor 2	6427	ENSG00000161547
16 ☐	P27635	NA	RPL10	ribosomal protein L10	6134	ENSG00000147403
17 ☐	P40616	NA	ARL1	ADP-ribosylation factor-like 1	400	ENSG00000120805
18 ☐	P09972	NA	ALDOC	aldolase C, fructose-bisphosphate	230	ENSG00000109107
19 ☐	Q9H0U4	NA	RAB1B	RAB1B, member RAS oncogene family	81876	ENSG00000174903
20 ☐	O75390	NA	CS	citrate synthase	1431	ENSG00000062485
21 ☐	P15121	NA	AKR1B1	aldo-keto reductase family 1, member B1 (aldose reductase)	231	ENSG00000085662
22 ☐	Q99536	NA	VAT1	vesicle amine transport protein 1 homolog (T. californica)	10493	ENSG00000108828
23 ☐	P16989	NA	CSDA	cold shock domain protein A	8531	ENSG00000060138
24 ☐	Q96AG4	NA	LRRC59	leucine rich repeat containing 59	55379	ENSG00000108829
25 ☐	Q9Y266	NA	NUDC	nuclear distribution C homolog (A. nidulans)	10726	ENSG00000090273
26 ☐	O43396	NA	TXNL1	thioredoxin-like 1	9352	ENSG00000091164
27 ☐	P42766	NA	RPL35	ribosomal protein L35	11224	ENSG00000136942
28 ☐	Q9HAV7	NA	GRPEL1	GrpE-like 1, mitochondrial (E. coli)	80273	ENSG00000109519
29 ☐	Q15020	NA	SART3	squamous cell carcinoma antigen recognized by T cells 3	9733	ENSG00000075856
30 ☐	P26447	NA	S100A4	S100 calcium binding protein A4	6275	ENSG00000196154
31 ☐	P68036	NA	UBE2L3	ubiquitin-conjugating enzyme E2L 3	7332	ENSG00000185651
32 ☐	Q9Y281	NA	CFL2	cofilin 2 (muscle)	1073	ENSG00000165410
33 ☐	Q9Y3U8	NA	RPL36	ribosomal protein L36	25873	ENSG00000130255
34 ☐	O75915	NA	ARL6IP5	ADP-ribosylation-like factor 6 interacting protein 5	10550	ENSG00000144746
35 ☐	P55795	NA	HNRNPH2	heterogeneous nuclear ribonucleoprotein H2 (H')	3188	ENSG00000126945
36 ☐	Q9BTT0	NA	ANP32E	acidic (leucine-rich) nuclear phosphoprotein 32 family, member E	81611	ENSG00000143401

Database:cellular component			Name:membrane-bounded organelle			ID:GO:0043227
C=9495; O=40; E=29.03; R=1.38; rawP=0.0011; adjP=0.0048						
Index	UserID	Value	Gene Symbol	Gene Name	EntrezGene	Ensembl
37 ☐	O43747	NA	AP1G1	adaptor-related protein complex 1, gamma 1 subunit	164	ENSG00000166747
38 ☐	Q00577	NA	PURA	purine-rich element binding protein A	5813	ENSG00000185129
39 ☐	Q96AE4	NA	FUBP1	far upstream element (FUSE) binding protein 1	8880	ENSG00000162613
40 ☐	P13693	NA	TPT1	tumor protein, translationally-controlled 1	7178	ENSG00000133112

Database:cellular component			Name:organelle lumen			ID:GO:0043233
C=3331; O=20; E=10.18; R=1.96; rawP=0.0012; adjP=0.0050						
Index	UserID	Value	Gene Symbol	Gene Name	EntrezGene	Ensembl
1 ☐	P39023	NA	RPL3	ribosomal protein L3	6122	ENSG00000100316
2 ☐	P09429	NA	HMGB1	high mobility group box 1	3146	ENSG00000189403
3 ☐	P42766	NA	RPL35	ribosomal protein L35	11224	ENSG00000136942
4 ☐	Q9HAV7	NA	GRPEL1	GrpE-like 1, mitochondrial (E. coli)	80273	ENSG00000109519
5 ☐	Q15020	NA	SART3	squamous cell carcinoma antigen recognized by T cells 3	9733	ENSG00000075856
6 ☐	P06748	NA	NPM1	nucleophosmin (nucleolar phosphoprotein B23, numatrin)	4869	ENSG00000181163
7 ☐	P30040	NA	ERP29	endoplasmic reticulum protein 29	10961	ENSG00000089248
8 ☐	Q9Y281	NA	CFL2	cofilin 2 (muscle)	1073	ENSG00000165410
9 ☐	Q9Y3U8	NA	RPL36	ribosomal protein L36	25873	ENSG00000130255
10 ☐	Q9UQE7	NA	SMC3	structural maintenance of chromosomes 3	9126	ENSG00000108055
11 ☐	Q01581	NA	HMGCS1	3-hydroxy-3-methylglutaryl-CoA synthase 1 (soluble)	3157	ENSG00000112972

Database:cellular component			Name:organelle lumen		ID:GO:0043233	
C=3331; O=20; E=10.18; R=1.96; rawP=0.0012; adjP=0.0050						
Index	UserID	Value	Gene Symbol	Gene Name	EntrezGene	Ensembl
12 ☐	Q01130	NA	SRSF2	serine/arginine-rich splicing factor 2	6427	ENSG00000161547
13 ☐	P55795	NA	HNRNPH2	heterogeneous nuclear ribonucleoprotein H2 (H')	3188	ENSG00000126945
14 ☐	O75390	NA	CS	citrate synthase	1431	ENSG00000062485
15 ☐	P15121	NA	AKR1B1	aldo-keto reductase family 1, member B1 (aldose reductase)	231	ENSG00000085662
16 ☐	Q96AG4	NA	LRRC59	leucine rich repeat containing 59	55379	ENSG00000108829
17 ☐	Q00577	NA	PURA	purine-rich element binding protein A	5813	ENSG00000185129
18 ☐	Q96AE4	NA	FUBP1	far upstream element (FUSE) binding protein 1	8880	ENSG00000162613
19 ☐	P13693	NA	TPT1	tumor protein, translationally-controlled 1	7178	ENSG00000133112
20 ☐	Q9Y266	NA	NUDC	nuclear distribution C homolog (A. nidulans)	10726	ENSG00000090273

Database:cellular component			Name:membrane-enclosed lumen		ID:GO:0031974	
C=3375; O=20; E=10.32; R=1.94; rawP=0.0014; adjP=0.0057						
Index	UserID	Value	Gene Symbol	Gene Name	EntrezGene	Ensembl
1 ☐	P39023	NA	RPL3	ribosomal protein L3	6122	ENSG00000100316
2 ☐	P09429	NA	HMGB1	high mobility group box 1	3146	ENSG00000189403
3 ☐	P42766	NA	RPL35	ribosomal protein L35	11224	ENSG00000136942
4 ☐	Q9HAV7	NA	GRPEL1	GrpE-like 1, mitochondrial (E. coli)	80273	ENSG00000109519
5 ☐	Q15020	NA	SART3	squamous cell carcinoma antigen recognized by T cells 3	9733	ENSG00000075856

Database:cellular component			Name:membrane-enclosed lumen			ID:GO:0031974
C=3375; O=20; E=10.32; R=1.94; rawP=0.0014; adjP=0.0057						
Index	UserID	Value	Gene Symbol	Gene Name	EntrezGene	Ensembl
6 ☐	P06748	NA	NPM1	nucleophosmin (nucleolar phosphoprotein B23, numatrin)	4869	ENSG00000181163
7 ☐	P30040	NA	ERP29	endoplasmic reticulum protein 29	10961	ENSG00000089248
8 ☐	Q9Y281	NA	CFL2	cofilin 2 (muscle)	1073	ENSG00000165410
9 ☐	Q9Y3U8	NA	RPL36	ribosomal protein L36	25873	ENSG00000130255
10 ☐	Q9UQE7	NA	SMC3	structural maintenance of chromosomes 3	9126	ENSG00000108055
11 ☐	Q01581	NA	HMGCS1	3-hydroxy-3-methylglutaryl-CoA synthase 1 (soluble)	3157	ENSG00000112972
12 ☐	Q01130	NA	SRSF2	serine/arginine-rich splicing factor 2	6427	ENSG00000161547
13 ☐	P55795	NA	HNRNPH2	heterogeneous nuclear ribonucleoprotein H2 (H')	3188	ENSG00000126945
14 ☐	O75390	NA	CS	citrate synthase	1431	ENSG00000062485
15 ☐	P15121	NA	AKR1B1	aldo-keto reductase family 1, member B1 (aldose reductase)	231	ENSG00000085662
16 ☐	Q96AG4	NA	LRRC59	leucine rich repeat containing 59	55379	ENSG00000108829
17 ☐	Q00577	NA	PURA	purine-rich element binding protein A	5813	ENSG00000185129
18 ☐	Q96AE4	NA	FUBP1	far upstream element (FUSE) binding protein 1	8880	ENSG00000162613
19 ☐	P13693	NA	TPT1	tumor protein, translationally-controlled 1	7178	ENSG00000133112
20 ☐	Q9Y266	NA	NUDC	nuclear distribution C homolog (A. nidulans)	10726	ENSG00000090273

Database:cellular component Name:axon ID:GO:0030424						
C=286; O=5; E=0.87; R=5.72; rawP=0.0018; adjP=0.0070						
Index	UserID	Value	Gene Symbol	Gene Name	EntrezGene	Ensembl
1 ☐	P62942	NA	FKBP1A	FK506 binding protein 1A, 12kDa	2280	ENSG00000088832
2 ☐	P63261	NA	ACTG1	actin, gamma 1	71	ENSG00000184009
3 ☐	P09972	NA	ALDOC	aldolase C, fructose-bisphosphate	230	ENSG00000109107
4 ☐	Q99497	NA	PARK7	parkinson protein 7	11315	ENSG00000116288
5 ☐	P30086	NA	PEBP1	phosphatidylethanolamine binding protein 1	5037	ENSG00000089220

Database:cellular component Name:macromolecular complex ID:GO:0032991						
C=3836; O=21; E=11.73; R=1.79; rawP=0.0029; adjP=0.0109						
Index	UserID	Value	Gene Symbol	Gene Name	EntrezGene	Ensembl
1 ☐	O43396	NA	TXNL1	thioredoxin-like 1	9352	ENSG00000091164
2 ☐	P39023	NA	RPL3	ribosomal protein L3	6122	ENSG00000100316
3 ☐	P09429	NA	HMGB1	high mobility group box 1	3146	ENSG00000189403
4 ☐	P42766	NA	RPL35	ribosomal protein L35	11224	ENSG00000136942
5 ☐	P63261	NA	ACTG1	actin, gamma 1	71	ENSG00000184009
6 ☐	P68036	NA	UBE2L3	ubiquitin-conjugating enzyme E2L 3	7332	ENSG00000185651
7 ☐	P06748	NA	NPM1	nucleophosmin (nucleolar phosphoprotein B23, numatrin)	4869	ENSG00000181163
8 ☐	Q02543	NA	RPL18A	ribosomal protein L18a	6142	ENSG00000105640
9 ☐	P40429	NA	RPL13A	ribosomal protein L13a	23521	ENSG00000142541
10 ☐	Q9Y3U8	NA	RPL36	ribosomal protein L36	25873	ENSG00000130255
11 ☐	P49207	NA	RPL34	ribosomal protein L34	6164	ENSG00000109475
12 ☐	Q9UQE7	NA	SMC3	structural maintenance of chromosomes 3	9126	ENSG00000108055
13 ☐	Q01130	NA	SRSF2	serine/arginine-rich splicing factor 2	6427	ENSG00000161547
14 ☐	P27635	NA	RPL10		6134	ENSG00000147403

Database:cellular component			Name:macromolecular complex			ID:GO:0032991
C=3836; O=21; E=11.73; R=1.79; rawP=0.0029; adjP=0.0109						
Index	UserID	Value	Gene Symbol	Gene Name	EntrezGene	Ensembl
				ribosomal protein L10		
15 ☐	P55795	NA	HNRNPH2	heterogeneous nuclear ribonucleoprotein H2 (H')	3188	ENSG00000126945
16 ☐	P84098	NA	RPL19	ribosomal protein L19	6143	ENSG00000108298
17 ☐	P16989	NA	CSDA	cold shock domain protein A	8531	ENSG00000060138
18 ☐	O43747	NA	AP1G1	adaptor-related protein complex 1, gamma 1 subunit	164	ENSG00000166747
19 ☐	Q00577	NA	PURA	purine-rich element binding protein A	5813	ENSG00000185129
20 ☐	P13693	NA	TPT1	tumor protein, translationally-controlled 1	7178	ENSG00000133112
21 ☐	Q9Y266	NA	NUDC	nuclear distribution C homolog (A. nidulans)	10726	ENSG00000090273

Database:cellular component			Name:nuclear lumen			ID:GO:0031981
C=2728; O=16; E=8.34; R=1.92; rawP=0.0058; adjP=0.0210						
Index	UserID	Value	Gene Symbol	Gene Name	EntrezGene	Ensembl
1 ☐	P39023	NA	RPL3	ribosomal protein L3	6122	ENSG00000100316
2 ☐	P09429	NA	HMGB1	high mobility group box 1	3146	ENSG00000189403
3 ☐	P42766	NA	RPL35	ribosomal protein L35	11224	ENSG00000136942
4 ☐	Q15020	NA	SART3	squamous cell carcinoma antigen recognized by T cells 3	9733	ENSG00000075856
5 ☐	P06748	NA	NPM1	nucleophosmin (nucleolar phosphoprotein B23, numatrin)	4869	ENSG00000181163
6 ☐	Q9Y281	NA	CFL2	cofilin 2 (muscle)	1073	ENSG00000165410
7 ☐	Q9Y3U8	NA	RPL36	ribosomal protein L36	25873	ENSG00000130255

Database:cellular component			Name:nuclear lumen		ID:GO:0031981	
C=2728; O=16; E=8.34; R=1.92; rawP=0.0058; adjP=0.0210						
Index	UserID	Value	Gene Symbol	Gene Name	EntrezGene	Ensembl
8 ☐	Q9UQE7	NA	SMC3	structural maintenance of chromosomes 3	9126	ENSG00000108055
9 ☐	Q01581	NA	HMGCS1	3-hydroxy-3-methylglutaryl-CoA synthase 1 (soluble)	3157	ENSG00000112972
10 ☐	Q01130	NA	SRSF2	serine/arginine-rich splicing factor 2	6427	ENSG00000161547
11 ☐	P55795	NA	HNRNPH2	heterogeneous nuclear ribonucleoprotein H2 (H')	3188	ENSG00000126945
12 ☐	P15121	NA	AKR1B1	aldo-keto reductase family 1, member B1 (aldose reductase)	231	ENSG00000085662
13 ☐	Q00577	NA	PURA	purine-rich element binding protein A	5813	ENSG00000185129
14 ☐	Q9Y266	NA	NUDC	nuclear distribution C homolog (A. nidulans)	10726	ENSG00000090273
15 ☐	P13693	NA	TPT1	tumor protein, translationally-controlled 1	7178	ENSG00000133112
16 ☐	Q96AE4	NA	FUBP1	far upstream element (FUSE) binding protein 1	8880	ENSG00000162613

Database:cellular component			Name:rough endoplasmic reticulum		ID:GO:0005791	
C=47; O=2; E=0.14; R=13.92; rawP=0.0091; adjP=0.0318						
Index	UserID	Value	Gene Symbol	Gene Name	EntrezGene	Ensembl
1 ☐	P61106	NA	RAB14	RAB14, member RAS oncogene family	51552	ENSG00000119396
2 ☐	P30086	NA	PEBP1	phosphatidylethanolamine binding protein 1	5037	ENSG00000089220

Database:cellular component			Name:cell projection		ID:GO:0042995	
C=1229; O=9; E=3.76; R=2.40; rawP=0.0113; adjP=0.0383						
Index	UserID	Value	Gene Symbol	Gene Name	EntrezGene	Ensembl
1 ☐	P62942	NA	FKBP1A	FK506 binding protein 1A, 12kDa	2280	ENSG00000088832

Database:cellular component Name:cell projection ID:GO:0042995						
C=1229; O=9; E=3.76; R=2.40; rawP=0.0113; adjP=0.0383						
Index	UserID	Value	Gene Symbol	Gene Name	EntrezGene	Ensembl
2 ☐	P09429	NA	HMGB1	high mobility group box 1	3146	ENSG00000189403
3 ☐	P63261	NA	ACTG1	actin, gamma 1	71	ENSG00000184009
4 ☐	P26447	NA	S100A4	S100 calcium binding protein A4	6275	ENSG00000196154
5 ☐	P09972	NA	ALDOC	aldolase C, fructose-bisphosphate	230	ENSG00000109107
6 ☐	P30086	NA	PEBP1	phosphatidylethanolamine binding protein 1	5037	ENSG00000089220
7 ☐	P09493	NA	TPM1	tropomyosin 1 (alpha)	7168	ENSG00000140416
8 ☐	Q00577	NA	PURA	purine-rich element binding protein A	5813	ENSG00000185129
9 ☐	Q99497	NA	PARK7	parkinson protein 7	11315	ENSG00000116288

Database:cellular component Name:endoplasmic reticulum ID:GO:0005783						
C=1277; O=9; E=3.90; R=2.31; rawP=0.0143; adjP=0.0469						
Index	UserID	Value	Gene Symbol	Gene Name	EntrezGene	Ensembl
1 ☐	P62942	NA	FKBP1A	FK506 binding protein 1A, 12kDa	2280	ENSG00000088832
2 ☐	P61106	NA	RAB14	RAB14, member RAS oncogene family	51552	ENSG00000119396
3 ☐	P27635	NA	RPL10	ribosomal protein L10	6134	ENSG00000147403
4 ☐	P60059	NA	SEC61G	Sec61 gamma subunit	23480	ENSG00000132432
5 ☐	O75915	NA	ARL6IP5	ADP-ribosylation-like factor 6 interacting protein 5	10550	ENSG00000144746
6 ☐	P30086	NA	PEBP1	phosphatidylethanolamine binding protein 1	5037	ENSG00000089220
7 ☐	P61019	NA	RAB2A	RAB2A, member RAS oncogene family	5862	ENSG00000104388
8 ☐	P30040	NA	ERP29	endoplasmic reticulum protein 29	10961	ENSG00000089248
9 ☐	Q96AG4	NA	LRRC59	leucine rich repeat containing 59	55379	ENSG00000108829

Database:cellular component Name:cytoplasmic membrane-bounded vesicle ID:GO:0016023						
C=861; O=7; E=2.63; R=2.66; rawP=0.0152; adjP=0.0484						
Index	UserID	Value	Gene Symbol	Gene Name	EntrezGene	Ensembl
1 ☐	P61019	NA	RAB2A	RAB2A, member RAS oncogene family	5862	ENSG00000104388

**Database:cellular component**				**Name:cytoplasmic membrane-bounded vesicle**		**ID:GO:0016023**
C=861; O=7; E=2.63; R=2.66; rawP=0.0152; adjP=0.0484						
Index	UserID	Value	Gene Symbol	Gene Name	EntrezGene	Ensembl
2 ☐	Q9BTT0	NA	ANP32E	acidic (leucine-rich) nuclear phosphoprotein 32 family, member E	81611	ENSG00000143401
3 ☐	P61106	NA	RAB14	RAB14, member RAS oncogene family	51552	ENSG00000119396
4 ☐	P30041	NA	PRDX6	peroxiredoxin 6	9588	ENSG00000117592
5 ☐	O43747	NA	AP1G1	adaptor-related protein complex 1, gamma 1 subunit	164	ENSG00000166747
6 ☐	P30040	NA	ERP29	endoplasmic reticulum protein 29	10961	ENSG00000089248
7 ☐	P30086	NA	PEBP1	phosphatidylethanolamine binding protein 1	5037	ENSG00000089220

**Database:cellular component**				**Name:cytoskeleton**		**ID:GO:0005856**
C=1783; O=11; E=5.45; R=2.02; rawP=0.0172; adjP=0.0502						
Index	UserID	Value	Gene Symbol	Gene Name	EntrezGene	Ensembl
1 ☐	Q9UQE7	NA	SMC3	structural maintenance of chromosomes 3	9126	ENSG00000108055
2 ☐	P63261	NA	ACTG1	actin, gamma 1	71	ENSG00000184009
3 ☐	P55795	NA	HNRNPH2	heterogeneous nuclear ribonucleoprotein H2 (H')	3188	ENSG00000126945
4 ☐	P09972	NA	ALDOC	aldolase C, fructose-bisphosphate	230	ENSG00000109107
5 ☐	P06748	NA	NPM1	nucleophosmin (nucleolar phosphoprotein B23, numatrin)	4869	ENSG00000181163
6 ☐	Q14019	NA	COTL1	coactosin-like 1 (Dictyostelium)	23406	ENSG00000103187
7 ☐	P09493	NA	TPM1	tropomyosin 1 (alpha)	7168	ENSG00000140416
8 ☐	Q9Y281	NA	CFL2	cofilin 2 (muscle)	1073	ENSG00000165410
9 ☐	P26639	NA	TARS	threonyl-tRNA synthetase	6897	ENSG00000113407
10 ☐	P13693	NA	TPT1	tumor protein, translationally-controlled 1	7178	ENSG00000133112
11 ☐	Q9Y266	NA	NUDC		10726	ENSG00000090273

Database:cellular component Name:cytoskeleton ID:GO:0005856						
C=1783; O=11; E=5.45; R=2.02; rawP=0.0172; adjP=0.0502						
Index	UserID	Value	Gene Symbol	Gene Name	EntrezGene	Ensembl
				nuclear distribution C homolog (A. nidulans)		

Database:cellular component Name:membrane-bounded vesicle ID:GO:0031988						
C=882; O=7; E=2.70; R=2.60; rawP=0.0171; adjP=0.0502						
Index	UserID	Value	Gene Symbol	Gene Name	EntrezGene	Ensembl
1 ☐	P61019	NA	RAB2A	RAB2A, member RAS oncogene family	5862	ENSG00000104388
2 ☐	Q9BTT0	NA	ANP32E	acidic (leucine-rich) nuclear phosphoprotein 32 family, member E	81611	ENSG00000143401
3 ☐	P61106	NA	RAB14	RAB14, member RAS oncogene family	51552	ENSG00000119396
4 ☐	P30041	NA	PRDX6	peroxiredoxin 6	9588	ENSG00000117592
5 ☐	O43747	NA	AP1G1	adaptor-related protein complex 1, gamma 1 subunit	164	ENSG00000166747
6 ☐	P30040	NA	ERP29	endoplasmic reticulum protein 29	10961	ENSG00000089248
7 ☐	P30086	NA	PEBP1	phosphatidylethanolamine binding protein 1	5037	ENSG00000089220

Database:cellular component Name:nuclear part ID:GO:0044428						
C=3057; O=16; E=9.35; R=1.71; rawP=0.0171; adjP=0.0502						
Index	UserID	Value	Gene Symbol	Gene Name	EntrezGene	Ensembl
1 ☐	P39023	NA	RPL3	ribosomal protein L3	6122	ENSG00000100316
2 ☐	P09429	NA	HMGB1	high mobility group box 1	3146	ENSG00000189403
3 ☐	P42766	NA	RPL35	ribosomal protein L35	11224	ENSG00000136942
4 ☐	Q15020	NA	SART3	squamous cell carcinoma antigen recognized by T cells 3	9733	ENSG00000075856
5 ☐	P06748	NA	NPM1	nucleophosmin (nucleolar phosphoprotein B23, numatrin)	4869	ENSG00000181163
6 ☐	Q9Y281	NA	CFL2	cofilin 2 (muscle)	1073	ENSG00000165410

Database:cellular component Name:nuclear part ID:GO:0044428						
C=3057; O=16; E=9.35; R=1.71; rawP=0.0171; adjP=0.0502						
Index	UserID	Value	Gene Symbol	Gene Name	EntrezGene	Ensembl
7 ☐	Q9Y3U8	NA	RPL36	ribosomal protein L36	25873	ENSG00000130255
8 ☐	Q9UQE7	NA	SMC3	structural maintenance of chromosomes 3	9126	ENSG00000108055
9 ☐	Q01581	NA	HMGCS1	3-hydroxy-3-methylglutaryl-CoA synthase 1 (soluble)	3157	ENSG00000112972
10 ☐	Q01130	NA	SRSF2	serine/arginine-rich splicing factor 2	6427	ENSG00000161547
11 ☐	P55795	NA	HNRNPH2	heterogeneous nuclear ribonucleoprotein H2 (H')	3188	ENSG00000126945
12 ☐	P15121	NA	AKR1B1	aldo-keto reductase family 1, member B1 (aldose reductase)	231	ENSG00000085662
13 ☐	Q00577	NA	PURA	purine-rich element binding protein A	5813	ENSG00000185129
14 ☐	Q9Y266	NA	NUDC	nuclear distribution C homolog (A. nidulans)	10726	ENSG00000090273
15 ☐	P13693	NA	TPT1	tumor protein, translationally-controlled 1	7178	ENSG00000133112
16 ☐	Q96AE4	NA	FUBP1	far upstream element (FUSE) binding protein 1	8880	ENSG00000162613

Database:cellular component Name:recycling endosome ID:GO:0055037						
C=73; O=2; E=0.22; R=8.96; rawP=0.0210; adjP=0.0596						
Index	UserID	Value	Gene Symbol	Gene Name	EntrezGene	Ensembl
1 ☐	P61106	NA	RAB14	RAB14, member RAS oncogene family	51552	ENSG00000119396
2 ☐	O43747	NA	AP1G1	adaptor-related protein complex 1, gamma 1 subunit	164	ENSG00000166747

Database:cellular component Name:cytoplasmic vesicle ID:GO:0031410						
C=927; O=7; E=2.83; R=2.47; rawP=0.0219; adjP=0.0605						
Index	UserID	Value	Gene Symbol	Gene Name	EntrezGene	Ensembl
1 ☐	P61019	NA	RAB2A	RAB2A, member RAS oncogene family	5862	ENSG00000104388
2 ☐	Q9BTT0	NA	ANP32E	acidic (leucine-rich) nuclear phosphoprotein 32 family, member E	81611	ENSG00000143401
3 ☐	P61106	NA	RAB14	RAB14, member RAS oncogene family	51552	ENSG00000119396
4 ☐	P30041	NA	PRDX6	peroxiredoxin 6	9588	ENSG00000117592
5 ☐	O43747	NA	AP1G1	adaptor-related protein complex 1, gamma 1 subunit	164	ENSG00000166747
6 ☐	P30040	NA	ERP29	endoplasmic reticulum protein 29	10961	ENSG00000089248
7 ☐	P30086	NA	PEBP1	phosphatidylethanolamine binding protein 1	5037	ENSG00000089220

Database:cellular component Name:clathrin-coated vesicle ID:GO:0030136						
C=207; O=3; E=0.63; R=4.74; rawP=0.0254; adjP=0.0667						
Index	UserID	Value	Gene Symbol	Gene Name	EntrezGene	Ensembl
1 ☐	P61106	NA	RAB14	RAB14, member RAS oncogene family	51552	ENSG00000119396
2 ☐	O43747	NA	AP1G1	adaptor-related protein complex 1, gamma 1 subunit	164	ENSG00000166747
3 ☐	P30086	NA	PEBP1	phosphatidylethanolamine binding protein 1	5037	ENSG00000089220

Database:cellular component Name:actin cytoskeleton ID:GO:0015629						
C=365; O=4; E=1.12; R=3.58; rawP=0.0251; adjP=0.0667						
Index	UserID	Value	Gene Symbol	Gene Name	EntrezGene	Ensembl
1 ☐	P09493	NA	TPM1	tropomyosin 1 (alpha)	7168	ENSG00000140416
2 ☐	P63261	NA	ACTG1	actin, gamma 1	71	ENSG00000184009
3 ☐	P55795	NA	HNRNPH2	heterogeneous nuclear ribonucleoprotein H2 (H')	3188	ENSG00000126945
4 ☐	P26639	NA	TARS	threonyl-tRNA synthetase	6897	ENSG00000113407

WebGestalt is currently developed and maintained by Jing Wang and Bing Zhang at the Zhang Lab. Other people who have made significant contribution to the project include Dexter Duncan, Stefan Kirov, Zhiao Shi, and Jay Snoddy.

Funding credits: NIH/NIAAA (U01 AA016662, U01 AA013512); NIH/NIDA (P01 DA015027); NIH/NIMH (P50 MH078028, P50 MH096972); NIH/NCI (U24 CA159988); NIH/NIGMS (R01 GM088822).
