# Supplementary material for: Expression of leukemia inhibitory factor in Müller glia cells is regulated by a redox-dependent mRNA stability mechanism
Source: BMC Biol. 2015 Apr 25;13:30. doi: 10.1186/s12915-015-0137-1 (PMC4462110; doi:10.1186/s12915-015-0137-1)
Supplement: Additional file 8: Table S1. — Real-time PCR primer sequences. Sequences of primers used for real-time PCR. Primers used for both rat and mouse samples are marked (Rn/Mm). Rn, Rattus norvegicus; Mm, Mus musculus. [file 12915_2015_137_MOESM8_ESM.pptx]

## Slide 1
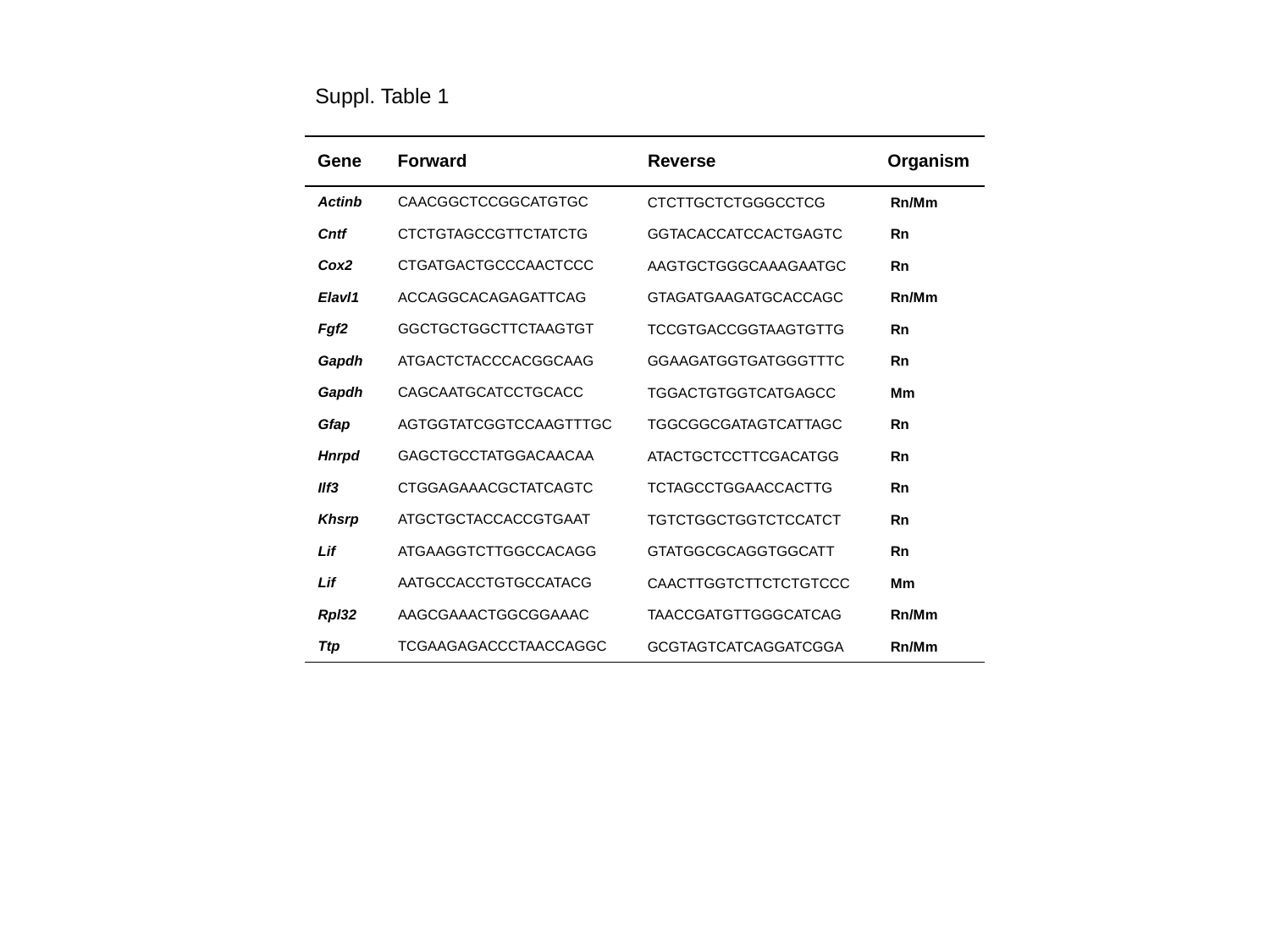

Suppl. Table 1
| Gene | Forward | Reverse | Organism |
| --- | --- | --- | --- |
| Actinb | CAACGGCTCCGGCATGTGC | CTCTTGCTCTGGGCCTCG | Rn/Mm |
| Cntf | CTCTGTAGCCGTTCTATCTG | GGTACACCATCCACTGAGTC | Rn |
| Cox2 | CTGATGACTGCCCAACTCCC | AAGTGCTGGGCAAAGAATGC | Rn |
| Elavl1 | ACCAGGCACAGAGATTCAG | GTAGATGAAGATGCACCAGC | Rn/Mm |
| Fgf2 | GGCTGCTGGCTTCTAAGTGT | TCCGTGACCGGTAAGTGTTG | Rn |
| Gapdh | ATGACTCTACCCACGGCAAG | GGAAGATGGTGATGGGTTTC | Rn |
| Gapdh | CAGCAATGCATCCTGCACC | TGGACTGTGGTCATGAGCC | Mm |
| Gfap | AGTGGTATCGGTCCAAGTTTGC | TGGCGGCGATAGTCATTAGC | Rn |
| Hnrpd | GAGCTGCCTATGGACAACAA | ATACTGCTCCTTCGACATGG | Rn |
| Ilf3 | CTGGAGAAACGCTATCAGTC | TCTAGCCTGGAACCACTTG | Rn |
| Khsrp | ATGCTGCTACCACCGTGAAT | TGTCTGGCTGGTCTCCATCT | Rn |
| Lif | ATGAAGGTCTTGGCCACAGG | GTATGGCGCAGGTGGCATT | Rn |
| Lif | AATGCCACCTGTGCCATACG | CAACTTGGTCTTCTCTGTCCC | Mm |
| Rpl32 | AAGCGAAACTGGCGGAAAC | TAACCGATGTTGGGCATCAG | Rn/Mm |
| Ttp | TCGAAGAGACCCTAACCAGGC | GCGTAGTCATCAGGATCGGA | Rn/Mm |
