# Supplementary material for: Expression of leukemia inhibitory factor in Müller glia cells is regulated by a redox-dependent mRNA stability mechanism
Source: BMC Biol. 2015 Apr 25;13:30. doi: 10.1186/s12915-015-0137-1 (PMC4462110; doi:10.1186/s12915-015-0137-1)
Supplement: Additional file 10: Table S2 — Primer sequences used for cloning and site-directed mutagenesis. Sequences of primers used for cloning, site-directed mutagenesis and templates for in vitro transcription. [file 12915_2015_137_MOESM10_ESM.pptx]

## Slide 1
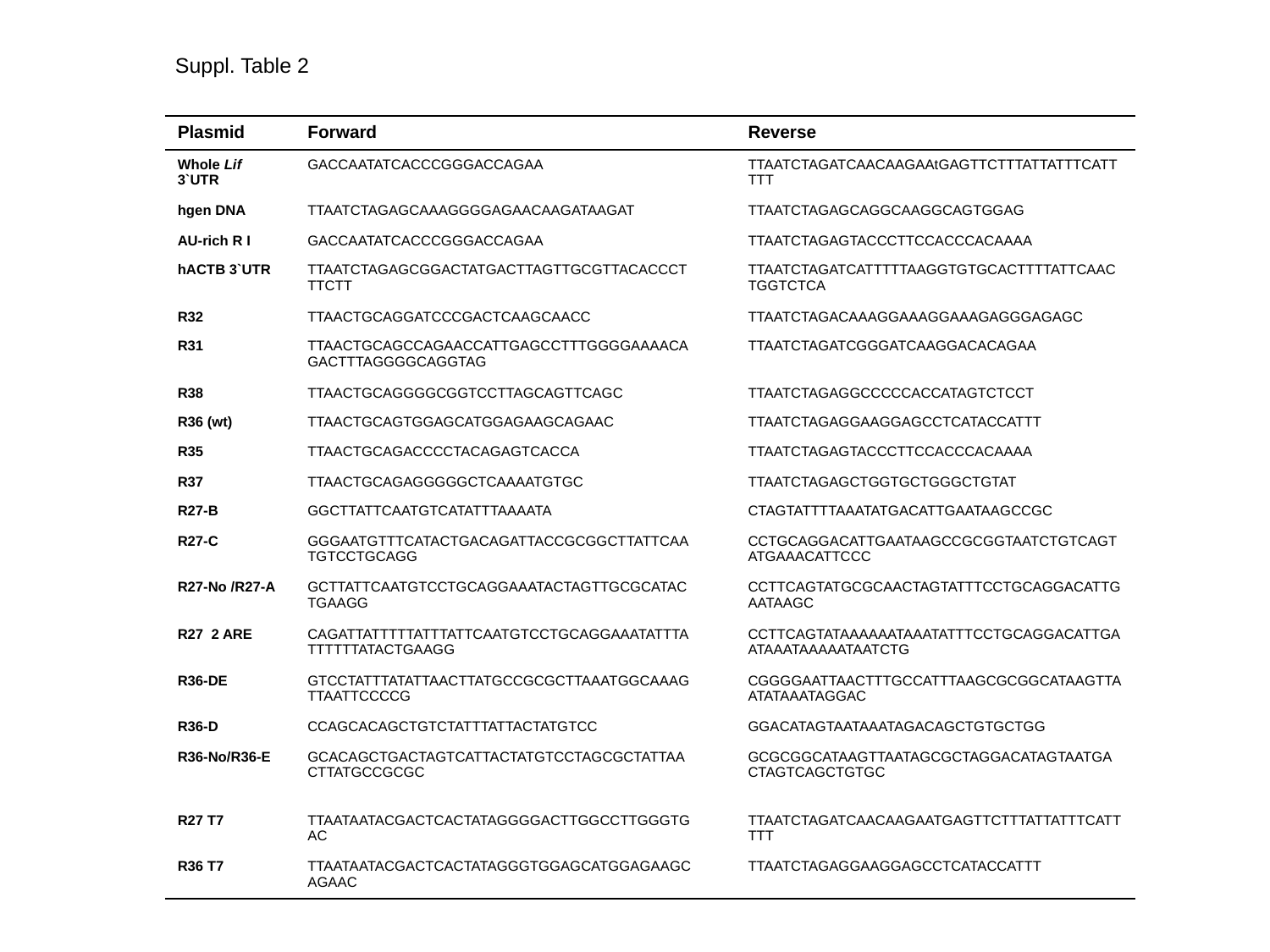

Suppl. Table 2
| Plasmid | Forward | | Reverse |
| --- | --- | --- | --- |
| Whole Lif 3`UTR | GACCAATATCACCCGGGACCAGAA | | TTAATCTAGATCAACAAGAAtGAGTTCTTTATTATTTCATTTTT |
| hgen DNA | TTAATCTAGAGCAAAGGGGAGAACAAGATAAGAT | | TTAATCTAGAGCAGGCAAGGCAGTGGAG |
| AU-rich R I | GACCAATATCACCCGGGACCAGAA | | TTAATCTAGAGTACCCTTCCACCCACAAAA |
| hACTB 3`UTR | TTAATCTAGAGCGGACTATGACTTAGTTGCGTTACACCCTTTCTT | | TTAATCTAGATCATTTTTAAGGTGTGCACTTTTATTCAACTGGTCTCA |
| R32 | TTAACTGCAGGATCCCGACTCAAGCAACC | | TTAATCTAGACAAAGGAAAGGAAAGAGGGAGAGC |
| R31 | TTAACTGCAGCCAGAACCATTGAGCCTTTGGGGAAAACAGACTTTAGGGGCAGGTAG | | TTAATCTAGATCGGGATCAAGGACACAGAA |
| R38 | TTAACTGCAGGGGCGGTCCTTAGCAGTTCAGC | | TTAATCTAGAGGCCCCCACCATAGTCTCCT |
| R36 (wt) | TTAACTGCAGTGGAGCATGGAGAAGCAGAAC | | TTAATCTAGAGGAAGGAGCCTCATACCATTT |
| R35 | TTAACTGCAGACCCCTACAGAGTCACCA | | TTAATCTAGAGTACCCTTCCACCCACAAAA |
| R37 | TTAACTGCAGAGGGGGCTCAAAATGTGC | | TTAATCTAGAGCTGGTGCTGGGCTGTAT |
| R27-B | GGCTTATTCAATGTCATATTTAAAATA | | CTAGTATTTTAAATATGACATTGAATAAGCCGC |
| R27-C | GGGAATGTTTCATACTGACAGATTACCGCGGCTTATTCAATGTCCTGCAGG | | CCTGCAGGACATTGAATAAGCCGCGGTAATCTGTCAGTATGAAACATTCCC |
| R27-No /R27-A | GCTTATTCAATGTCCTGCAGGAAATACTAGTTGCGCATACTGAAGG | | CCTTCAGTATGCGCAACTAGTATTTCCTGCAGGACATTGAATAAGC |
| R27 2 ARE | CAGATTATTTTTATTTATTCAATGTCCTGCAGGAAATATTTATTTTTTATACTGAAGG | | CCTTCAGTATAAAAAATAAATATTTCCTGCAGGACATTGAATAAATAAAAATAATCTG |
| R36-DE | GTCCTATTTATATTAACTTATGCCGCGCTTAAATGGCAAAGTTAATTCCCCG | | CGGGGAATTAACTTTGCCATTTAAGCGCGGCATAAGTTAATATAAATAGGAC |
| R36-D | CCAGCACAGCTGTCTATTTATTACTATGTCC | | GGACATAGTAATAAATAGACAGCTGTGCTGG |
| R36-No/R36-E | GCACAGCTGACTAGTCATTACTATGTCCTAGCGCTATTAACTTATGCCGCGC | | GCGCGGCATAAGTTAATAGCGCTAGGACATAGTAATGACTAGTCAGCTGTGC |
| R27 T7 | TTAATAATACGACTCACTATAGGGGACTTGGCCTTGGGTGAC | | TTAATCTAGATCAACAAGAATGAGTTCTTTATTATTTCATTTTT |
| R36 T7 | TTAATAATACGACTCACTATAGGGTGGAGCATGGAGAAGCAGAAC | | TTAATCTAGAGGAAGGAGCCTCATACCATTT |
