# Supplementary material for: Expression of leukemia inhibitory factor in Müller glia cells is regulated by a redox-dependent mRNA stability mechanism
Source: BMC Biol. 2015 Apr 25;13:30. doi: 10.1186/s12915-015-0137-1 (PMC4462110; doi:10.1186/s12915-015-0137-1)
Supplement: Additional file 11: Table S3. — siRNA sequences. Target sequences of siRNAs used to knockdown respective genes in rMC-1 cells. [file 12915_2015_137_MOESM11_ESM.pptx]

## Slide 1
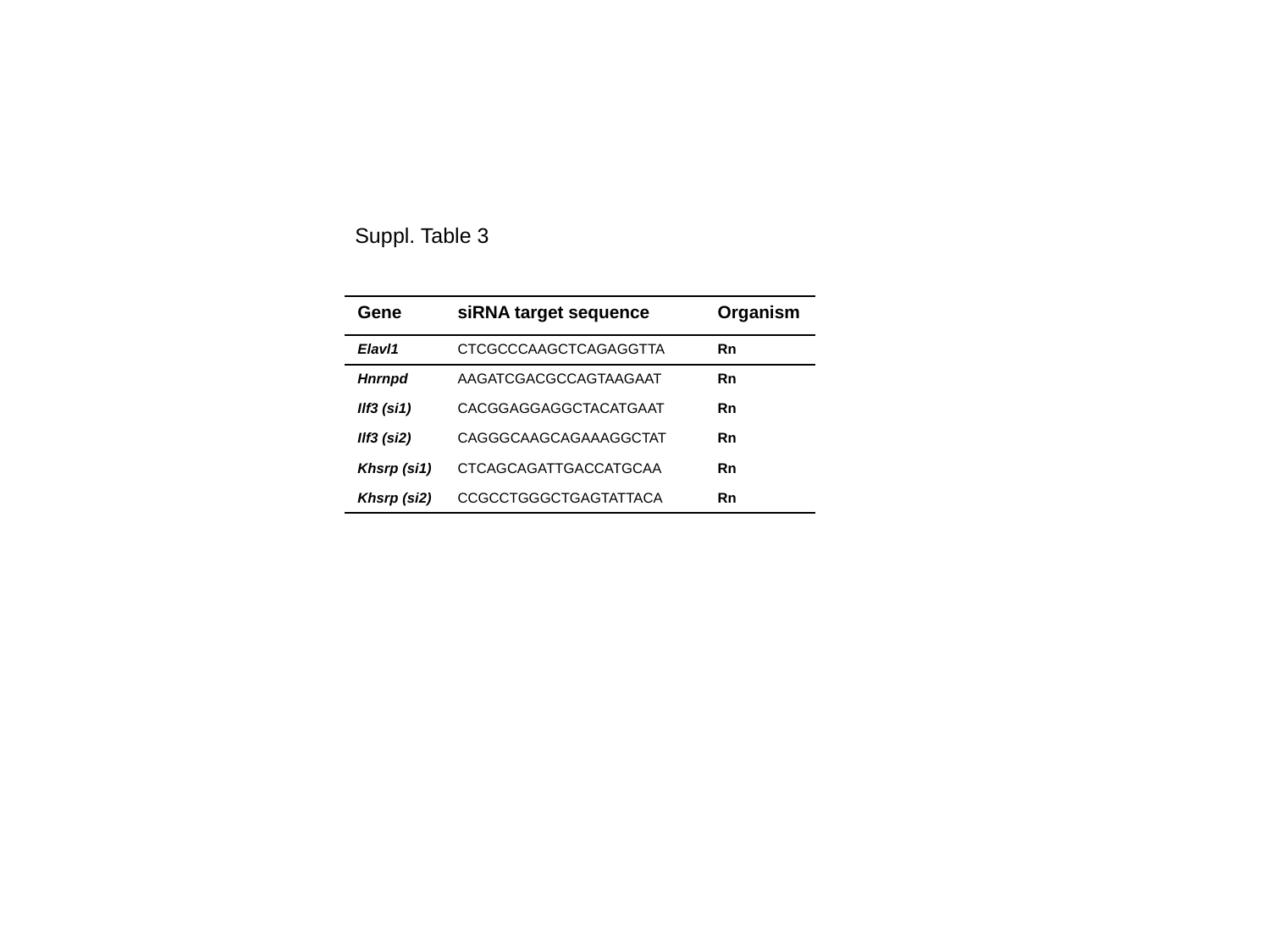

Suppl. Table 3
| Gene | siRNA target sequence | Organism |
| --- | --- | --- |
| Elavl1 | CTCGCCCAAGCTCAGAGGTTA | Rn |
| Hnrnpd | AAGATCGACGCCAGTAAGAAT | Rn |
| Ilf3 (si1) | CACGGAGGAGGCTACATGAAT | Rn |
| Ilf3 (si2) | CAGGGCAAGCAGAAAGGCTAT | Rn |
| Khsrp (si1) | CTCAGCAGATTGACCATGCAA | Rn |
| Khsrp (si2) | CCGCCTGGGCTGAGTATTACA | Rn |
